# Supplementary material for: The forecasted prevalence of comorbidities and multimorbidity in people with HIV in the United States through the year 2030: A modeling study
Source: PLoS Med. 2024 Jan 12;21(1):e1004325. doi: 10.1371/journal.pmed.1004325 (PMC10833859; doi:10.1371/journal.pmed.1004325)
Supplement: S1 Fig — (DOCX) [file pmed.1004325.s001.docx]

**S1 Fig:** Comorbidity incidence validation plots, by subgroup.

**
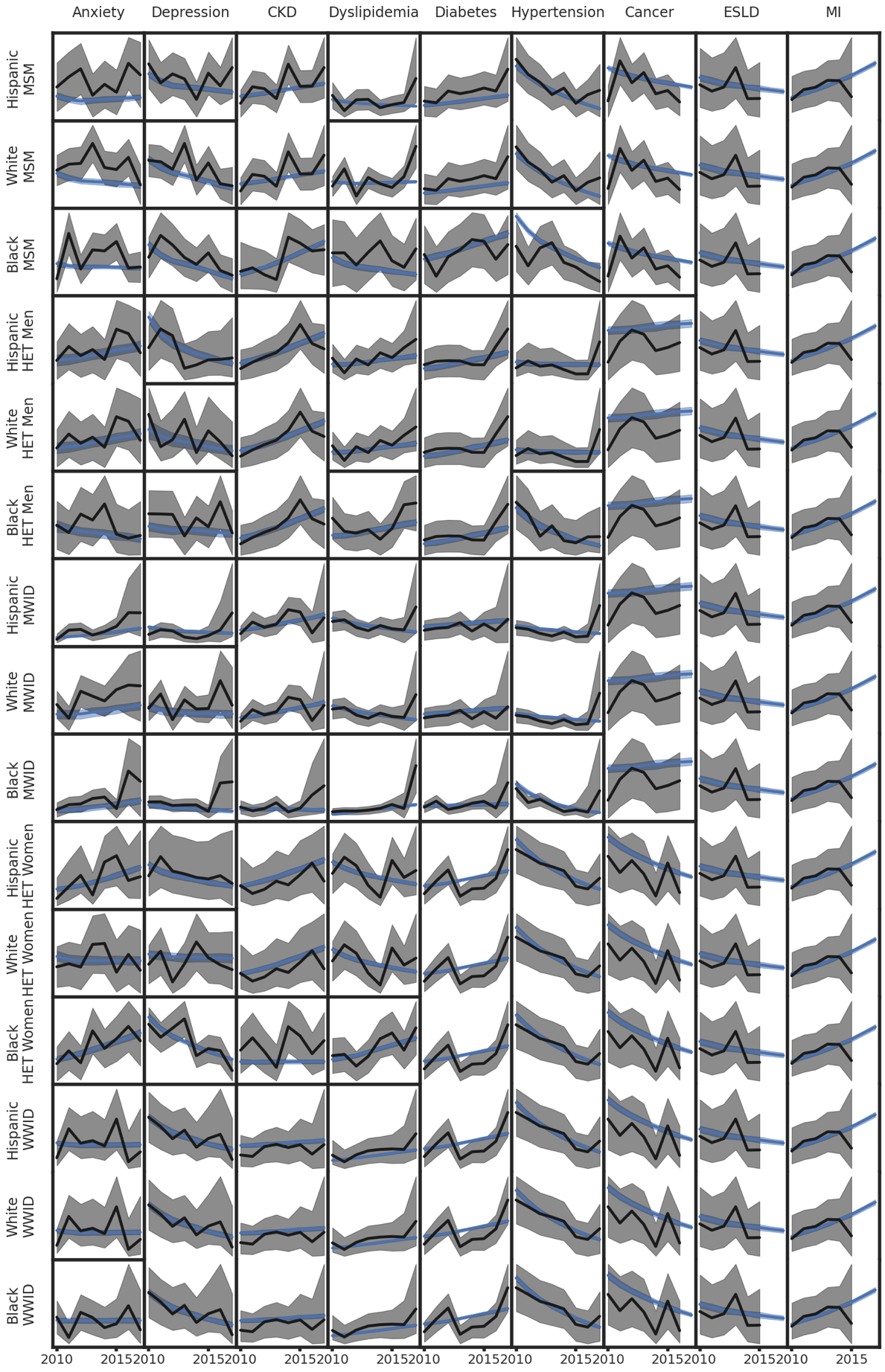
**

Footnotes:

Black line=observed annual incidence from the NA-ACCORD.

Gray shading = the 95% confidence intervals of the observed annual incidence from the NA-ACCORD data.

Blue line=forecasted annual incidence from PEARL.

Orange plot shading signals <75% PEARL estimates [total of 7 observed incidence (2009-2015) for MI and ESLD and 9 observed incidence (2009-2017) for all other comorbidities] are within +/-5% of the observed incidence or the 95% confidence interval of the observed incidence [whichever is larger].

The y axis is unlabeled and is not the same scale in each plot.

For subgroups with small observed sample size in the NA-ACCORD, the ordered collapsing strategy is: 1) by race/ethnicity, 2) by gender.
