# Supplementary material for: The forecasted prevalence of comorbidities and multimorbidity in people with HIV in the United States through the year 2030: A modeling study
Source: PLoS Med. 2024 Jan 12;21(1):e1004325. doi: 10.1371/journal.pmed.1004325 (PMC10833859; doi:10.1371/journal.pmed.1004325)
Supplement: S2 Fig — (DOCX) [file pmed.1004325.s002.docx]

**S2 Fig:** Comorbidity prevalence validation plots, by subgroup

**
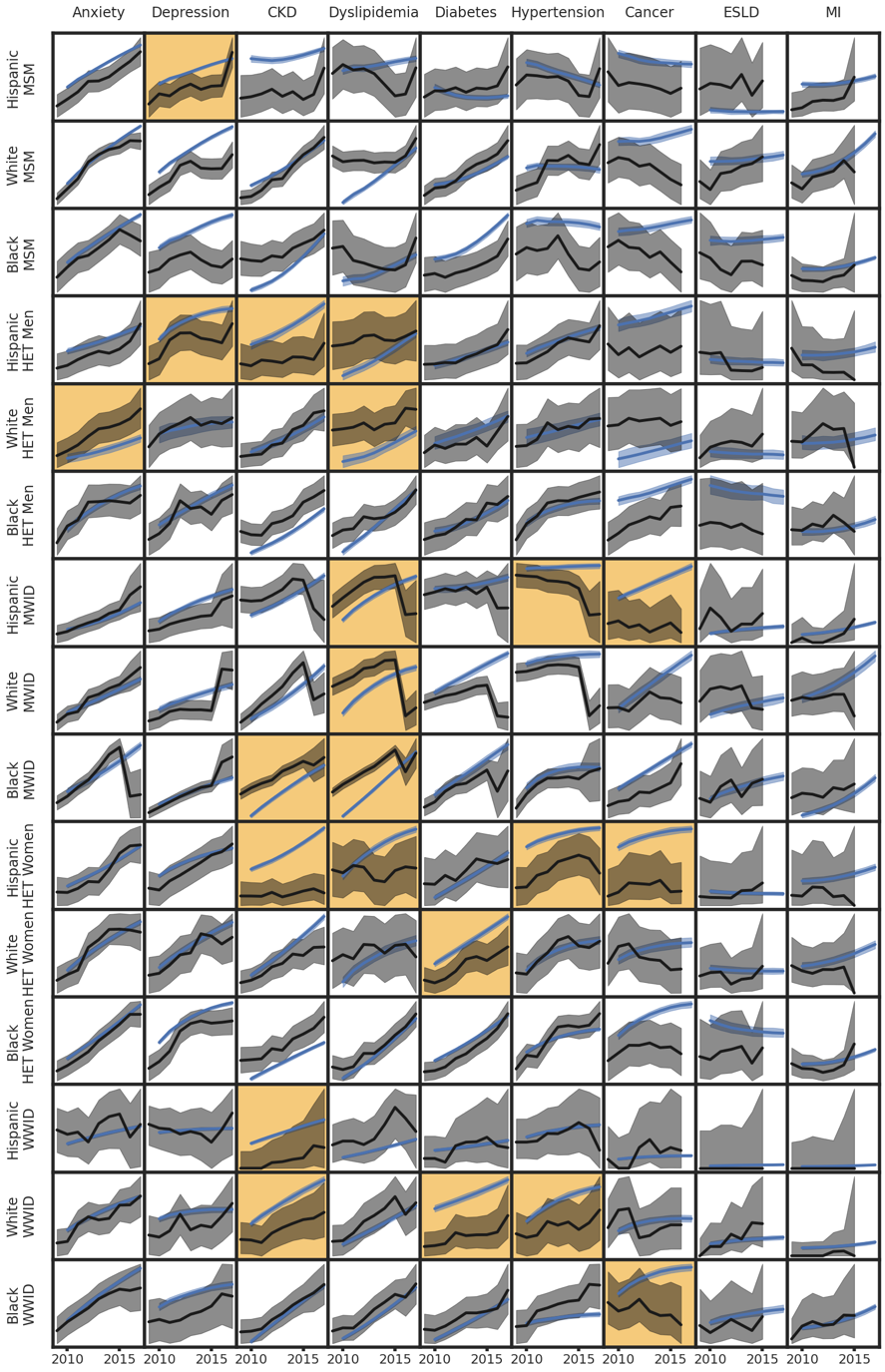
**

Footnotes:

Black line=observed annual prevalence from the NA-ACCORD.

Gray shading = the 95% confidence intervals of the observed annual prevalence from the NA-ACCORD data.

Blue line=forecasted annual prevalence from PEARL

Orange plot = <75% PEARL estimates [total of 7 observed prevalence (2009-2015) for MI and ESLD and 9 observed prevalence (2009-2017) for all other comorbidities] are within +/-5% of the observed prevalence or the 95% confidence interval of the observed prevalence [whichever is larger].

Please note that the orange plots occur among Hispanic sub-groups, white heterosexual men, and Black/AA and White women who injected drugs; these groups have smaller observed sample sizes and were combined with other subgroups resulting in differences between the NA-ACCORD observed estimates and PEARL estimates.

The y axis is unlabeled and is not the same scale in each plot.

For subgroups with small observed sample size in the NA-ACCORD, the ordered collapsing strategy is: 1) by race/ethnicity, 2) by gender.
