# Supplementary material for: The forecasted prevalence of comorbidities and multimorbidity in people with HIV in the United States through the year 2030: A modeling study
Source: PLoS Med. 2024 Jan 12;21(1):e1004325. doi: 10.1371/journal.pmed.1004325 (PMC10833859; doi:10.1371/journal.pmed.1004325)
Supplement: S3 Fig — (DOCX) [file pmed.1004325.s003.docx]

**S3 Fig:** Comparing the age distributions of ART-users in PEARL to the observed data from NA-ACCORD, 2010, 2013, and 2017


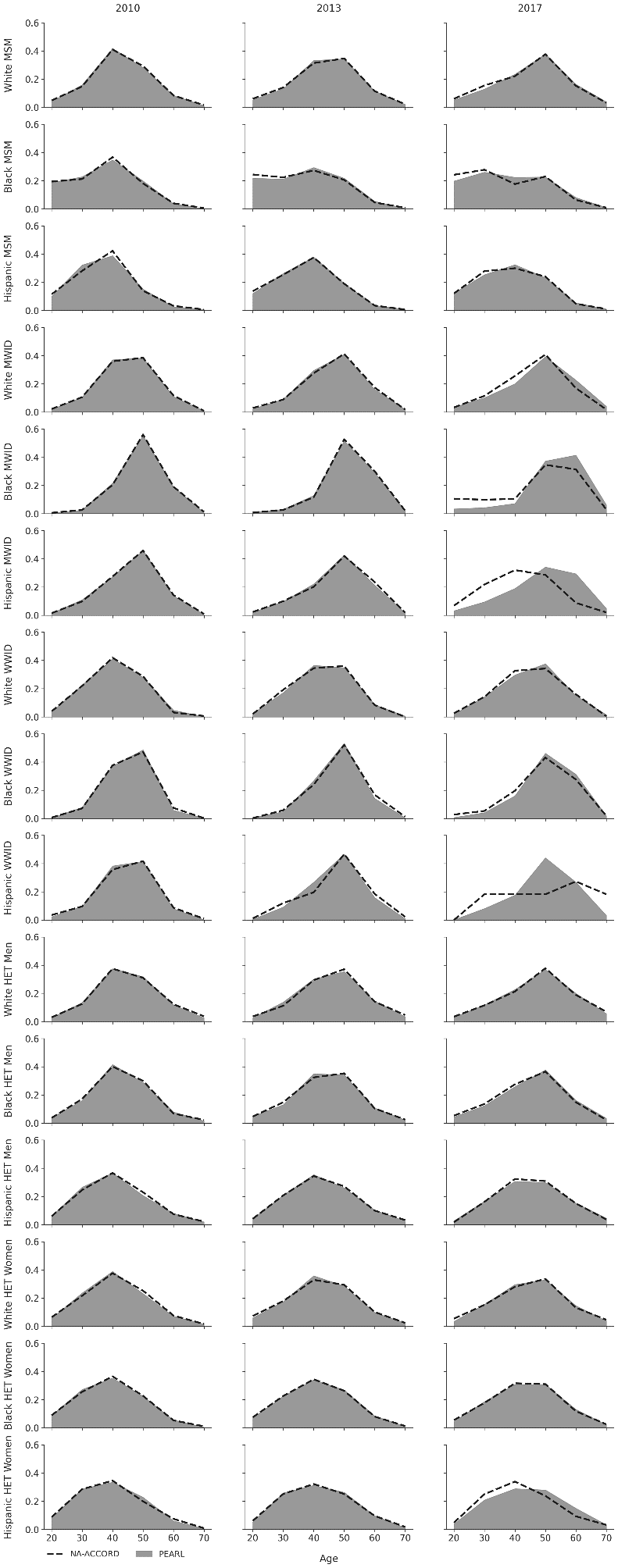


Black dashed line is observed age distribution in the NA-ACCORD.

Gray shading is PEARL estimated age distribution.
