# Supplementary material for: The forecasted prevalence of comorbidities and multimorbidity in people with HIV in the United States through the year 2030: A modeling study
Source: PLoS Med. 2024 Jan 12;21(1):e1004325. doi: 10.1371/journal.pmed.1004325 (PMC10833859; doi:10.1371/journal.pmed.1004325)
Supplement: S4 Fig — Trends in multimorbidity distribution by age groups, overall and within the 15 subgroups of people with HIV, (a) overall; (b) White, (c) Black/African American, and (d) Hispanic men who have sex with men; (e) White, (f) Black/African American, and (g) Hispanic men with injection drug use as their HIV acquisition risk factor; (h) White, (i) Black/African American, and (j) Hispanic women with injection drug use as their HIV acquisition risk factor; (k) White, (l) Black/African American, and (m) Hispanic heterosexual men; (n) White, (o) Black/African American, and (p) Hispanic heterosexual women. (DOCX) [file pmed.1004325.s004.docx]

## **S4 Fig:** Trends in multimorbidity distribution by age groups, overall and within the 15 subgroups of people with HIV, a) overall; b) White, c) Black/African American, and d) Hispanic men who have sex with men; e) White, f) Black/African American, and g) Hispanic men with injection drug use as their HIV acquisition risk factor; h) White, i) Black/African American, and j) Hispanic women with injection drug use as their HIV acquisition risk factor; k) White, l) Black/African American, and m) Hispanic heterosexual men; n) White, o) Black/African American, and p) Hispanic heterosexual women

S3a) Overall


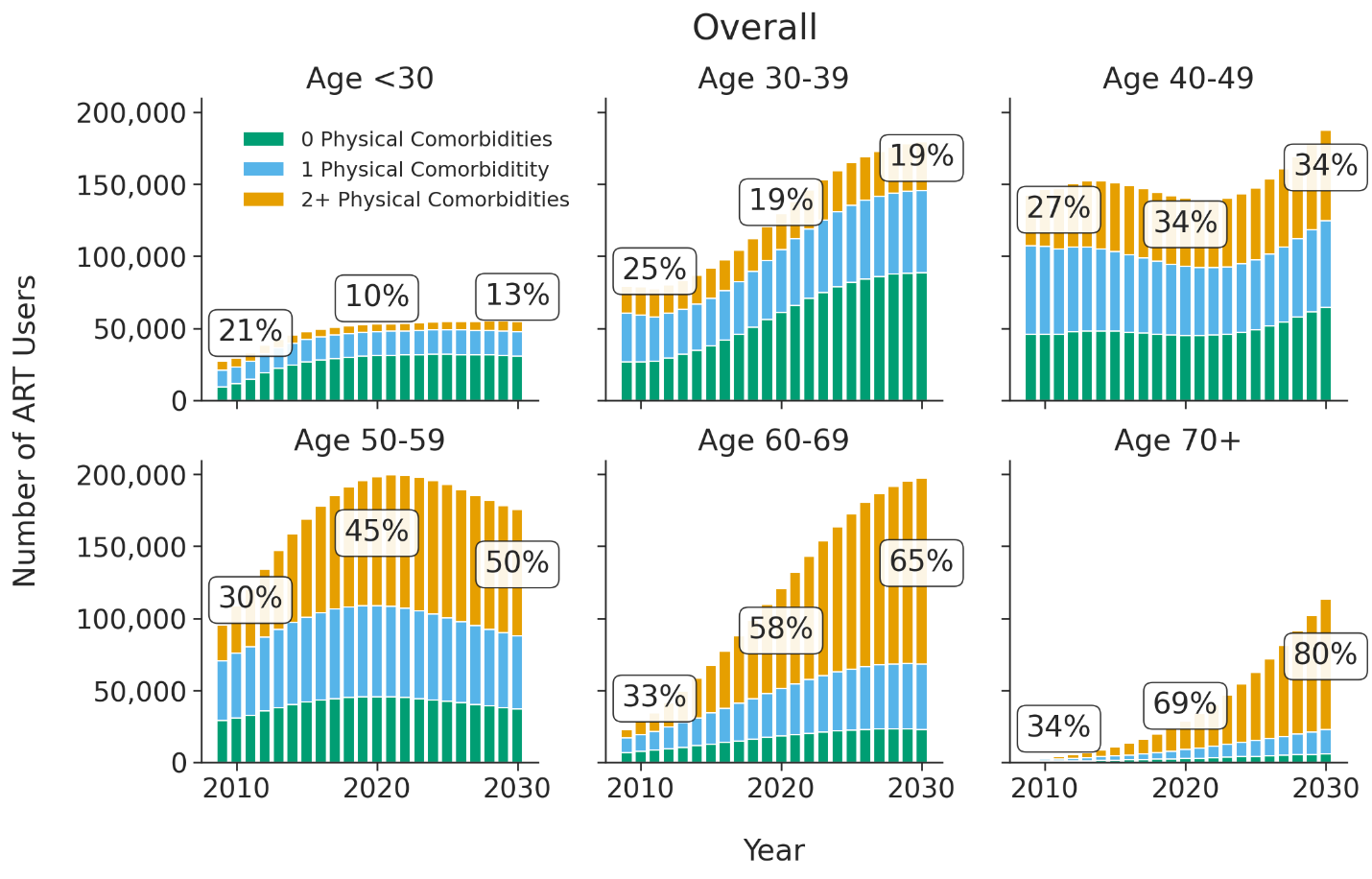


S3b) White men who have sex with men


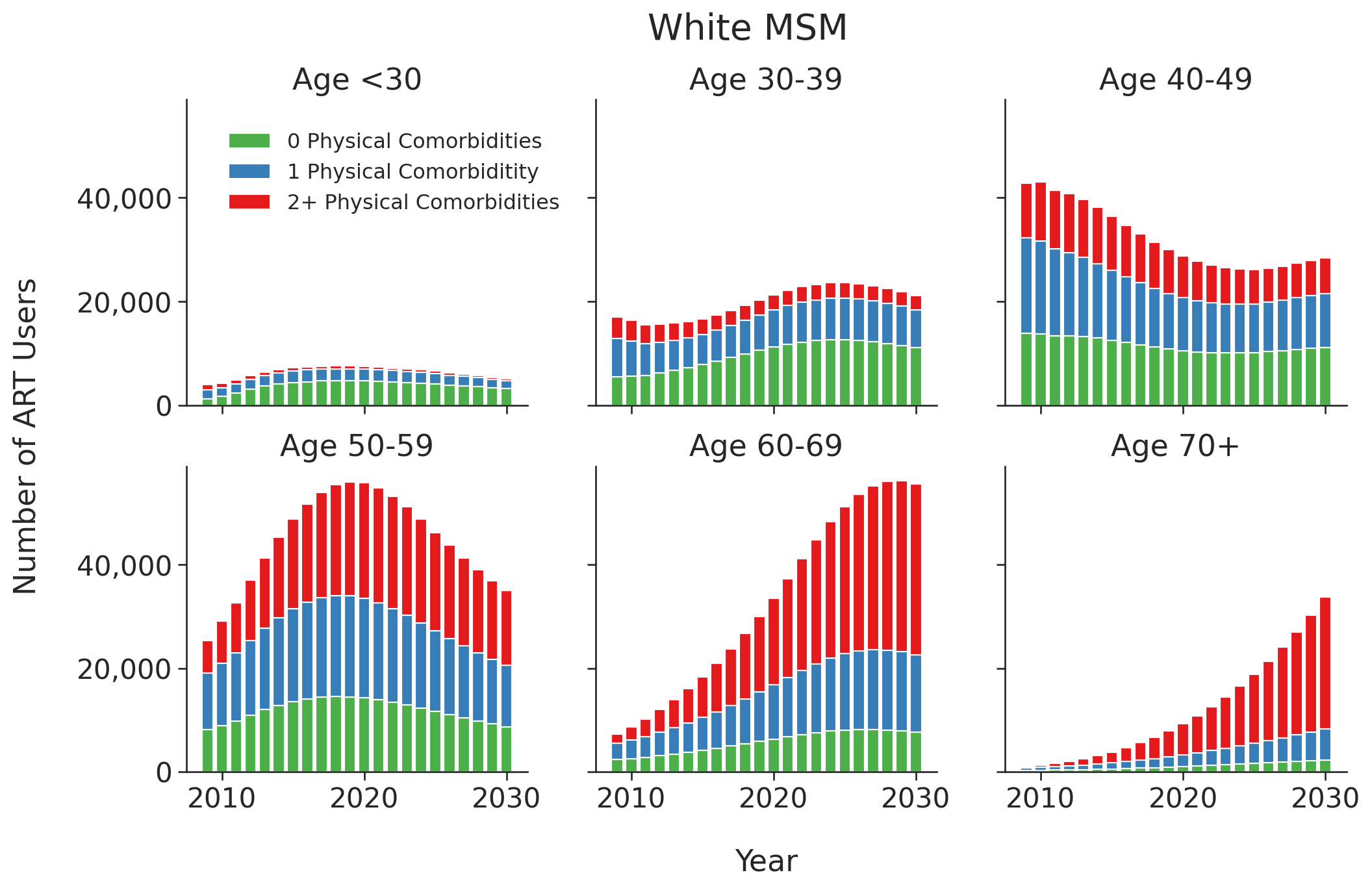


S3c) Black/African American men who have sex with men


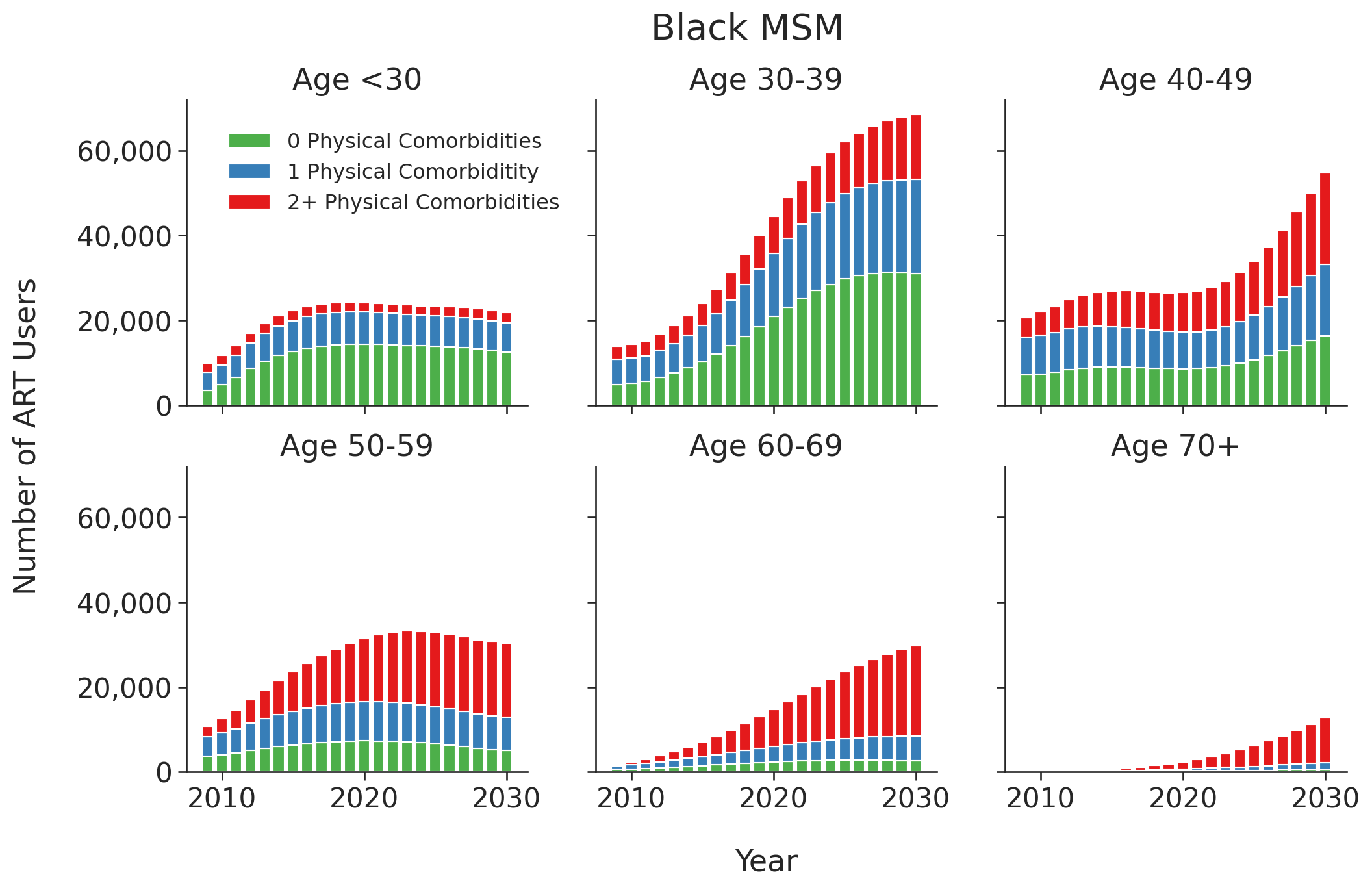


S3d) Hispanic men who have sex with men


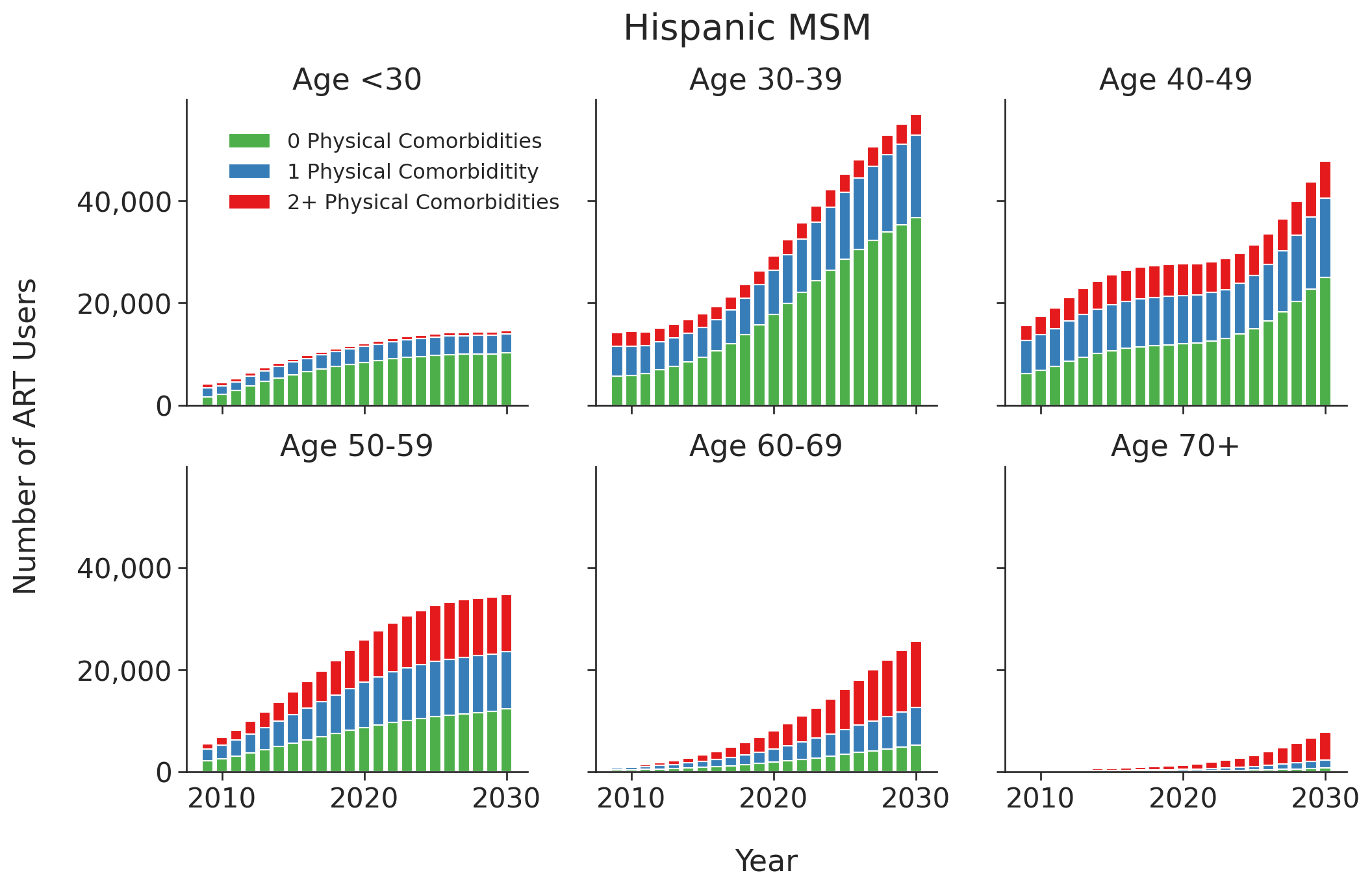


S3e) White men with injection drug use as their HIV acquisition risk factor


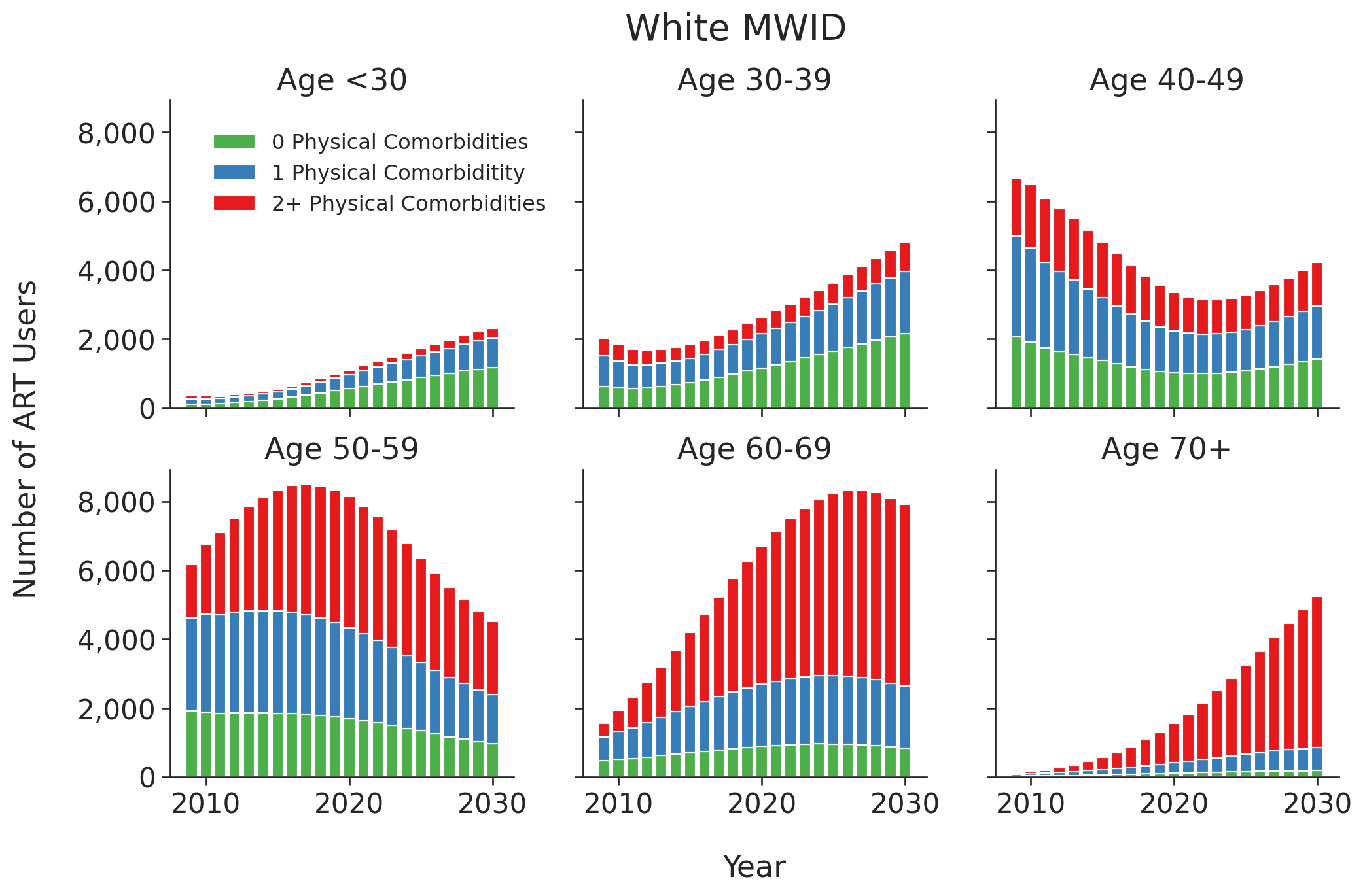


S3f) Black/African American men with injection drug use as their HIV acquisition risk factor


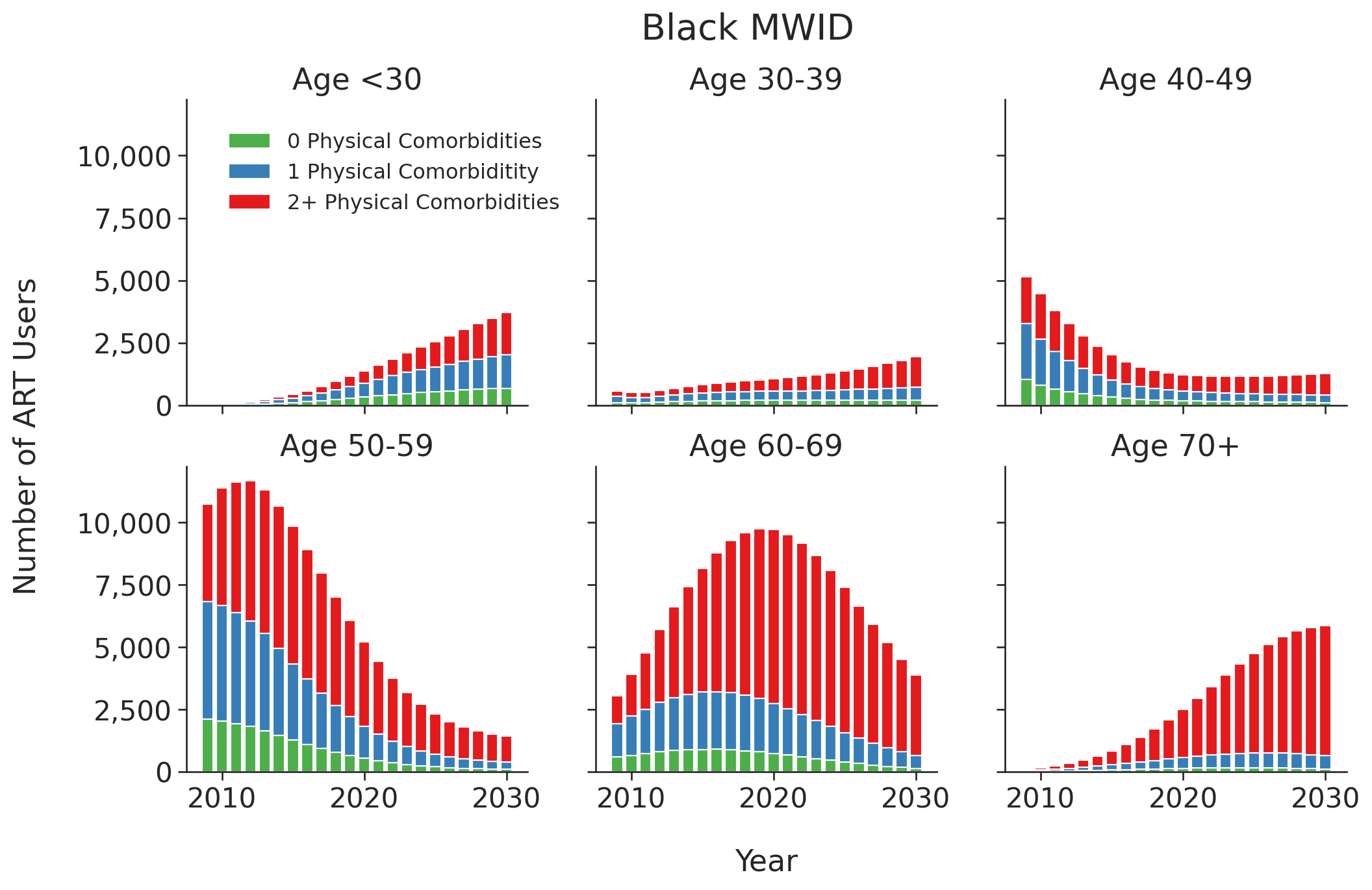


S3g) Hispanic men with injection drug use as their HIV acquisition risk factor


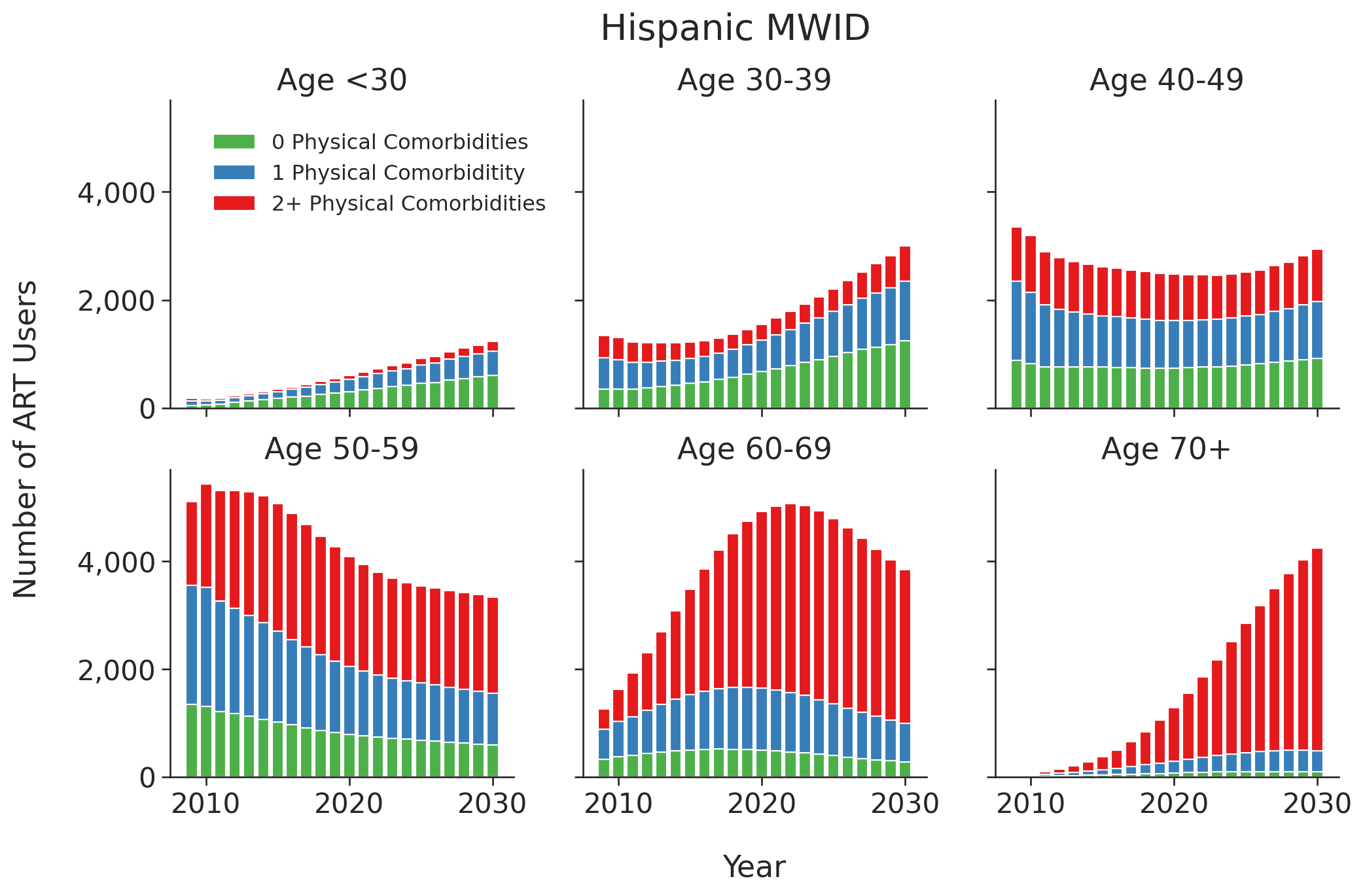


S3h) White women with injection drug use as their HIV acquisition risk factor


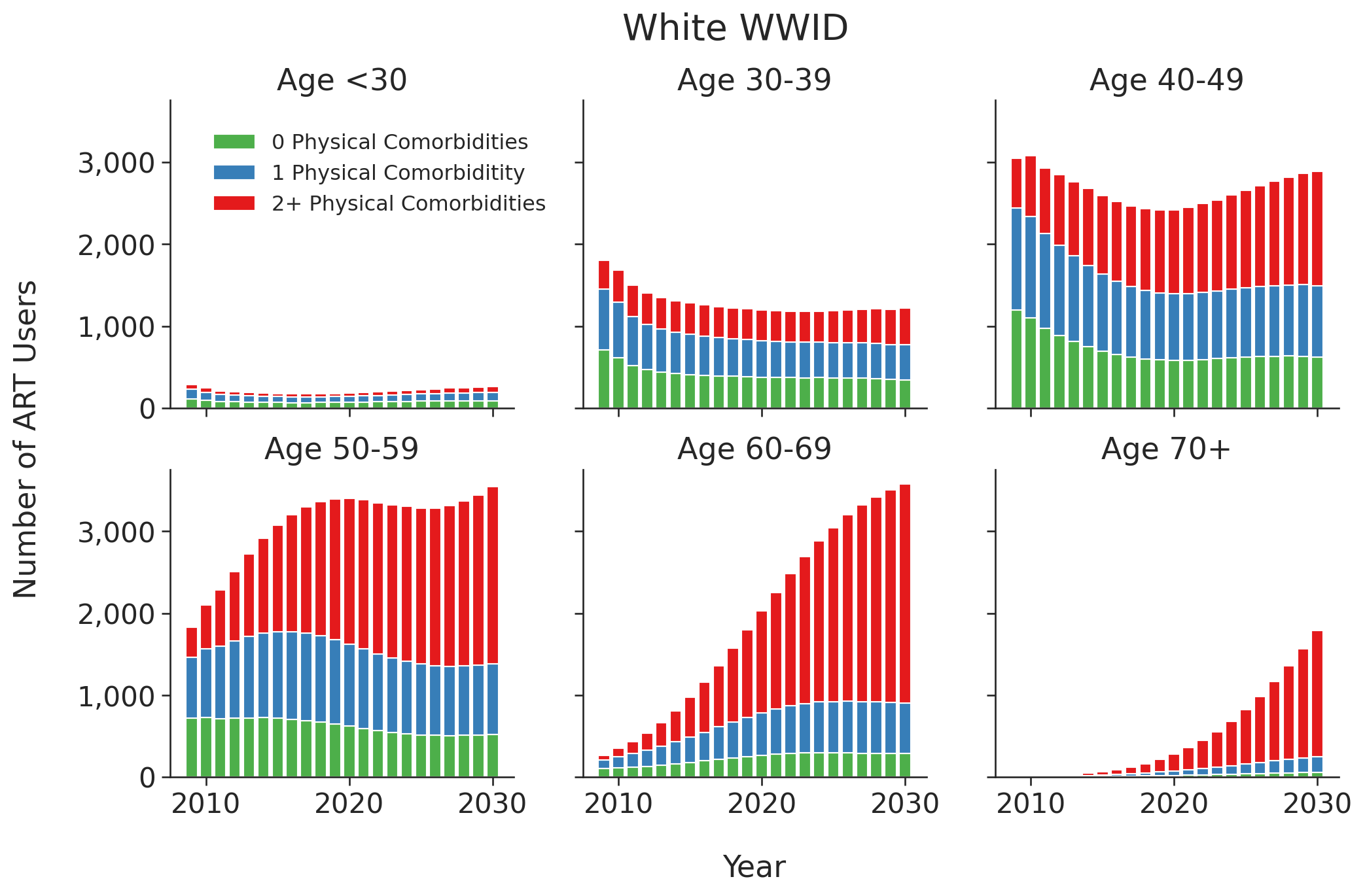


S3i) Black/African American women with injection drug use as their HIV acquisition risk factor

**
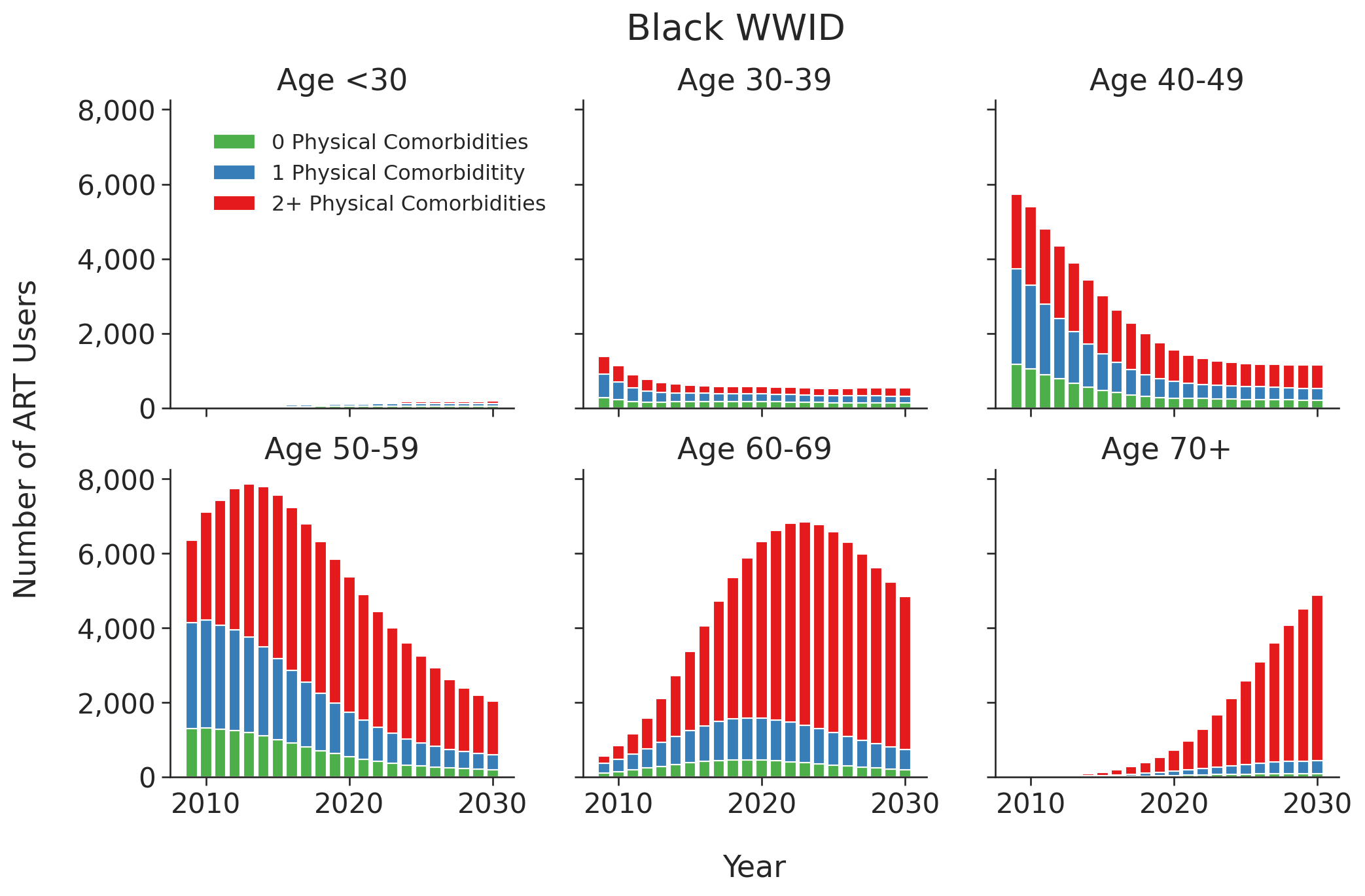
**

S3j) Hispanic women with injection drug use as their HIV acquisition risk factor

**
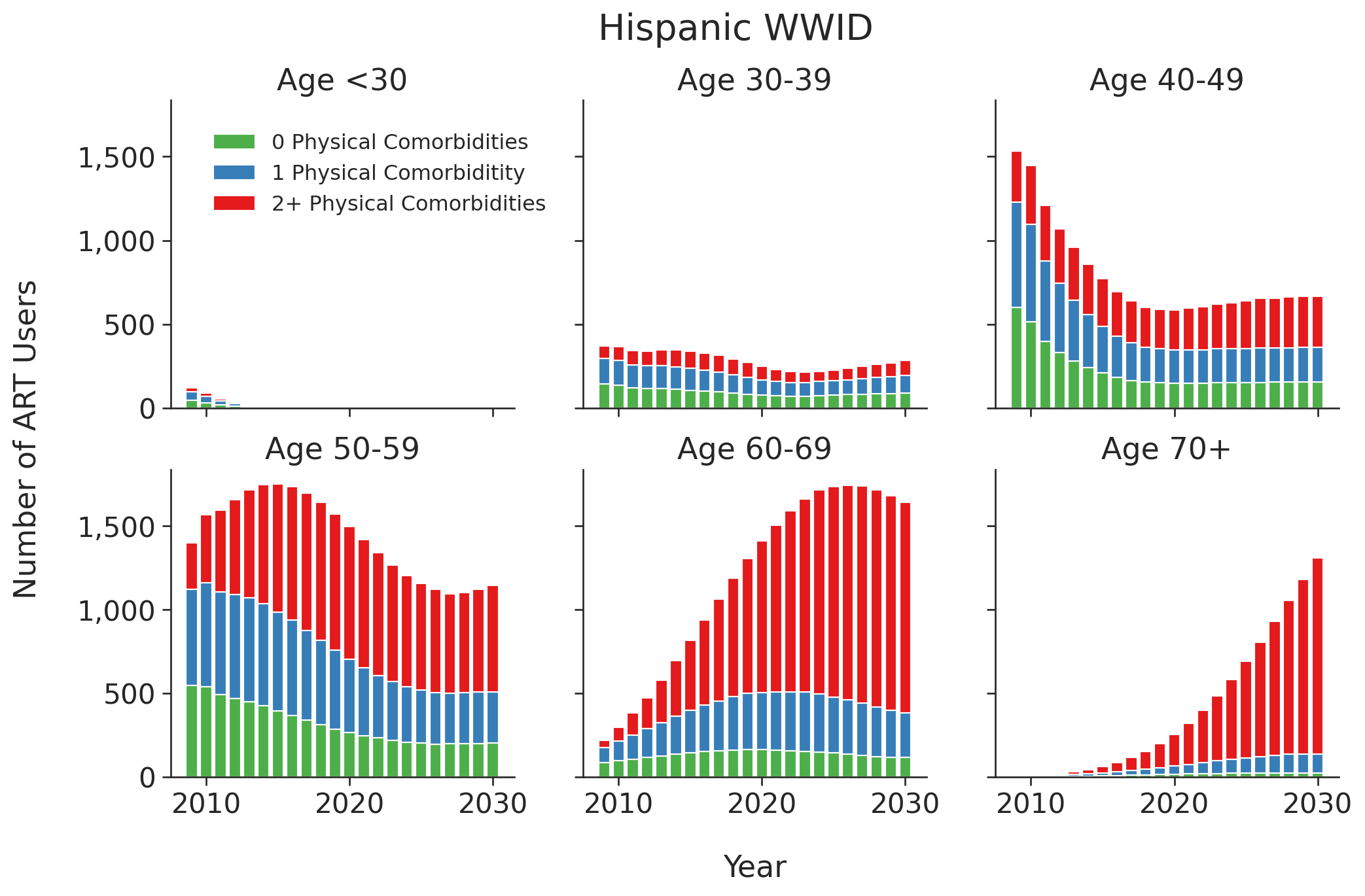
**

S3k) White heterosexual men


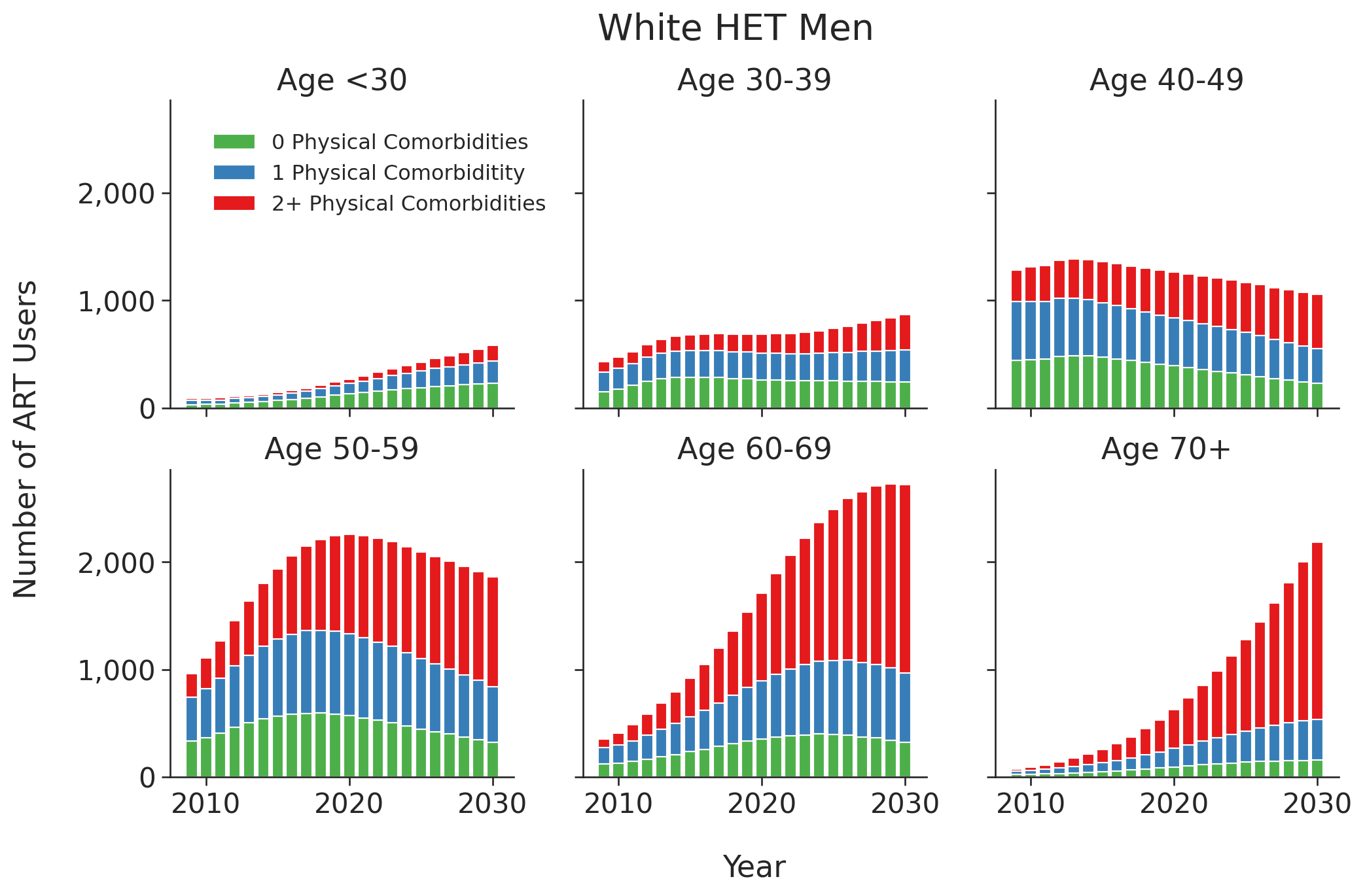


S3l) Black/African American heterosexual men


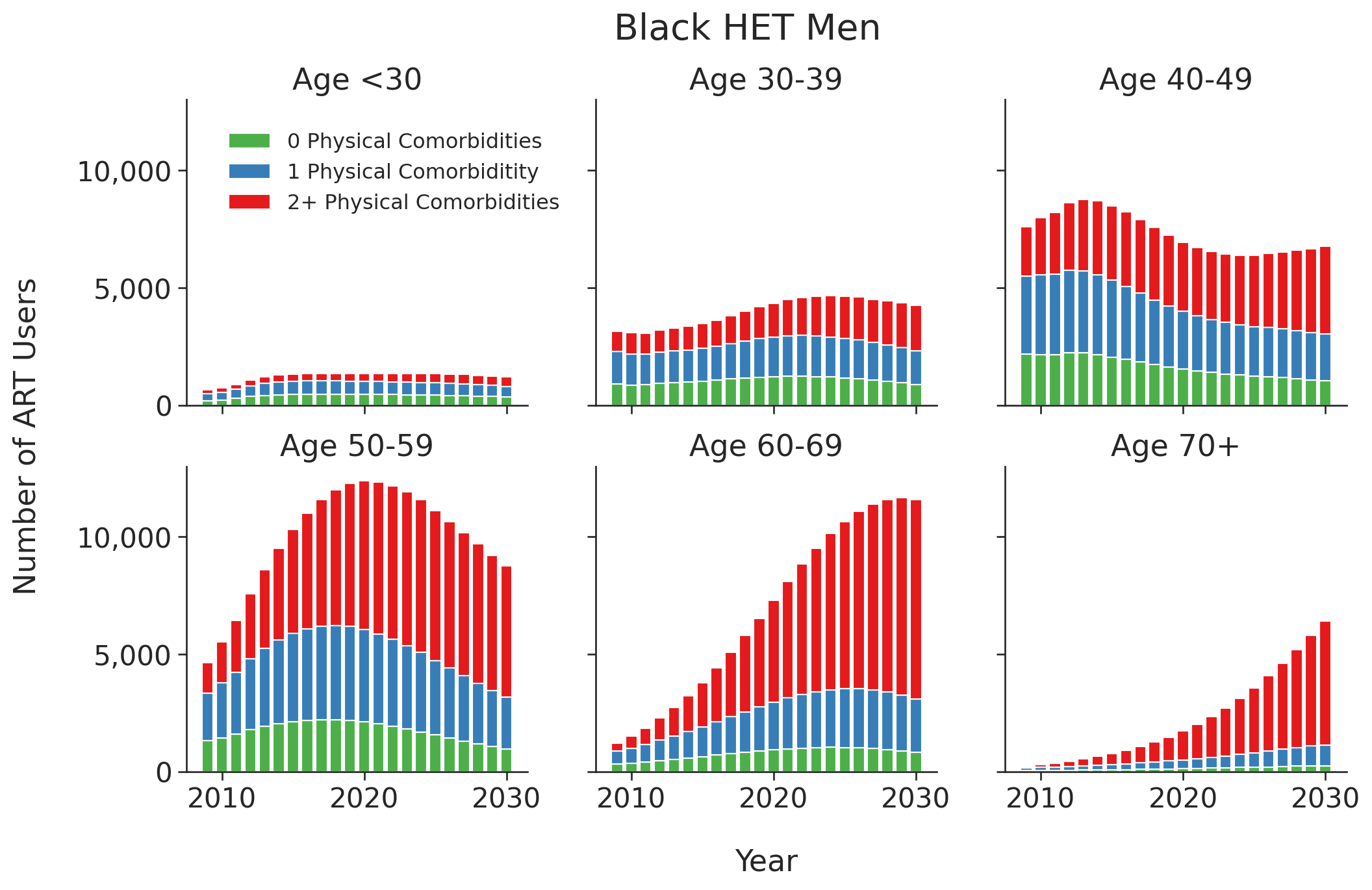


S3m) Hispanic heterosexual men


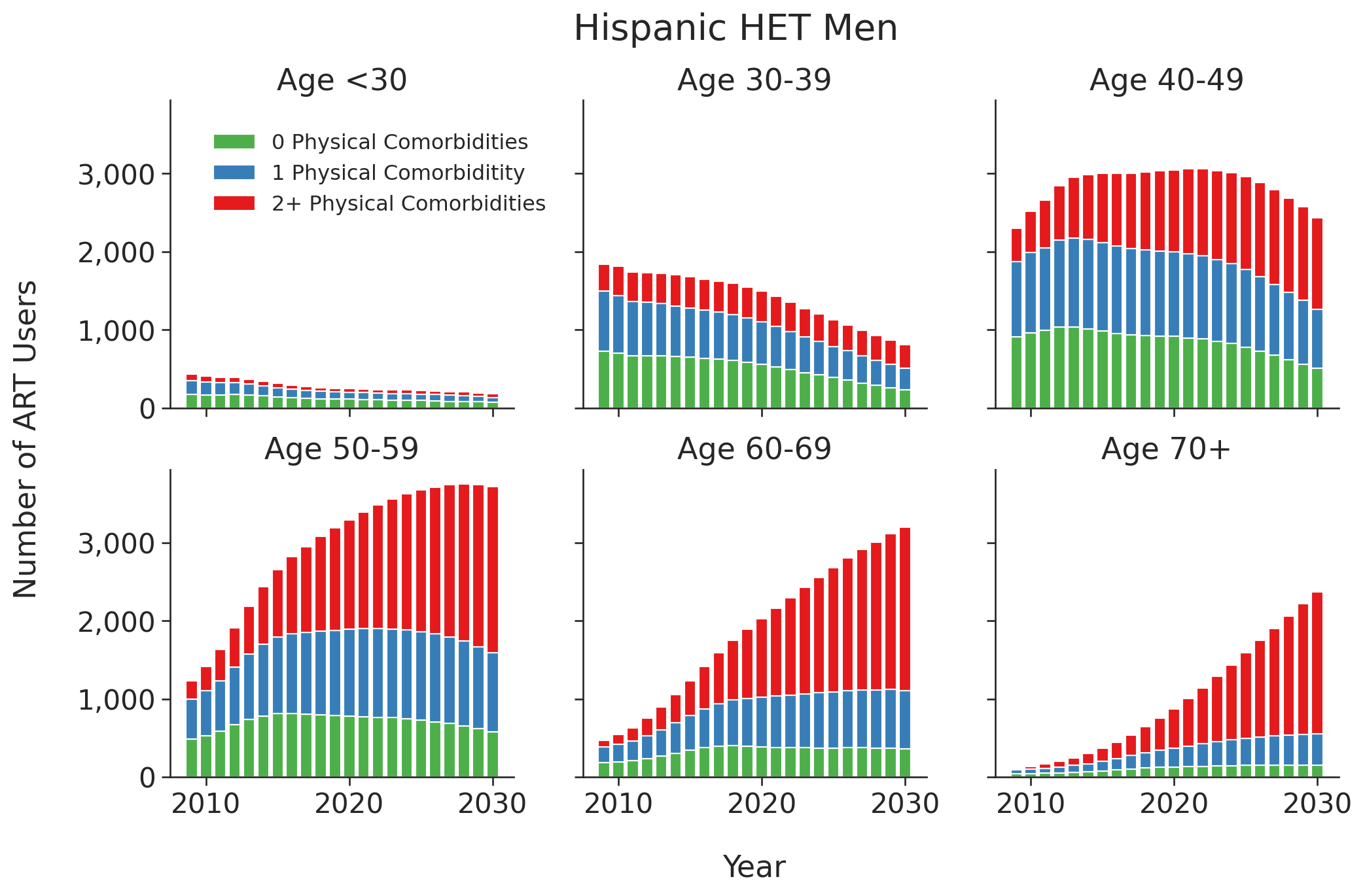


S3n) White heterosexual women


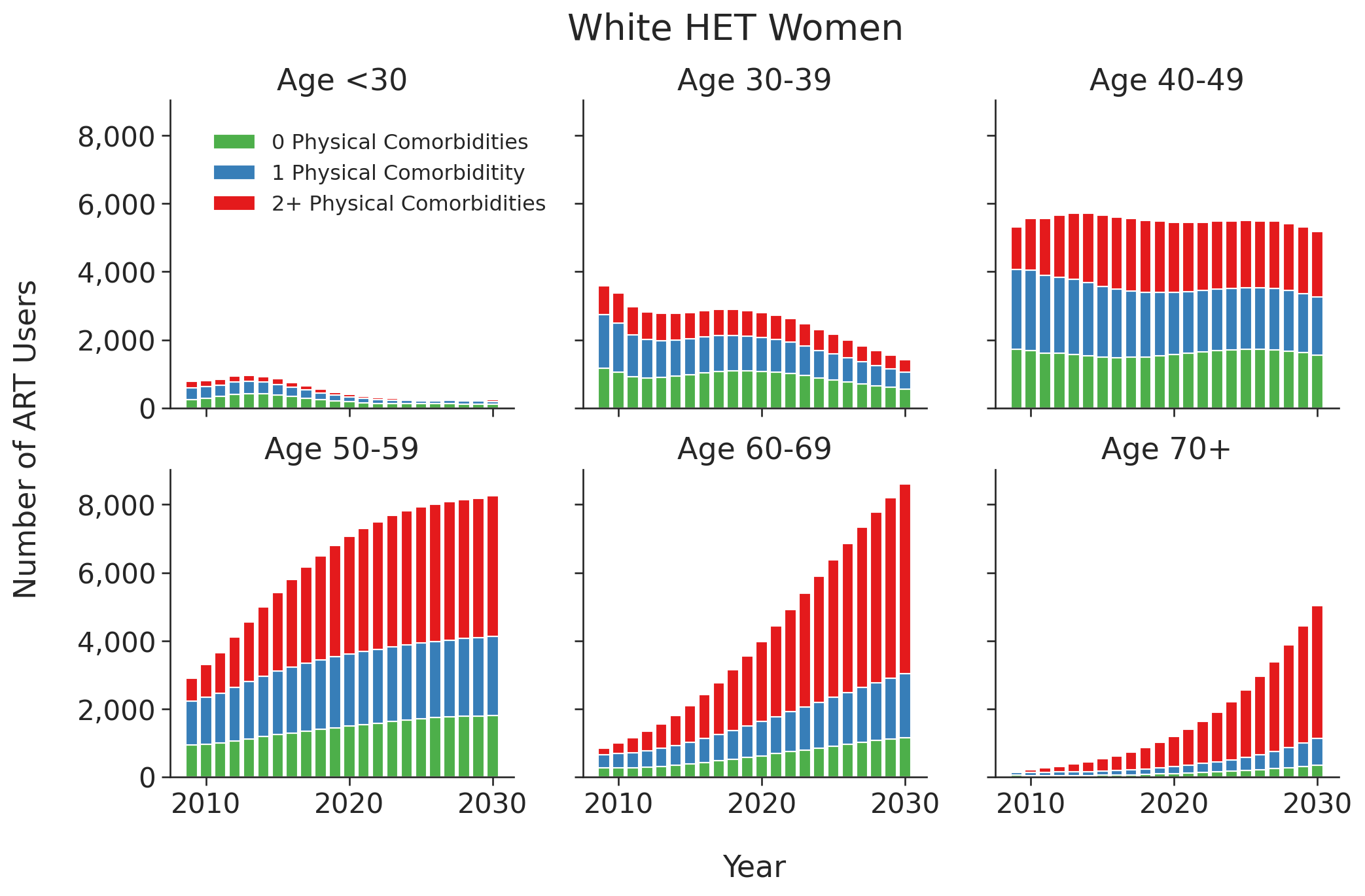


S3o) Black/African American heterosexual women

**
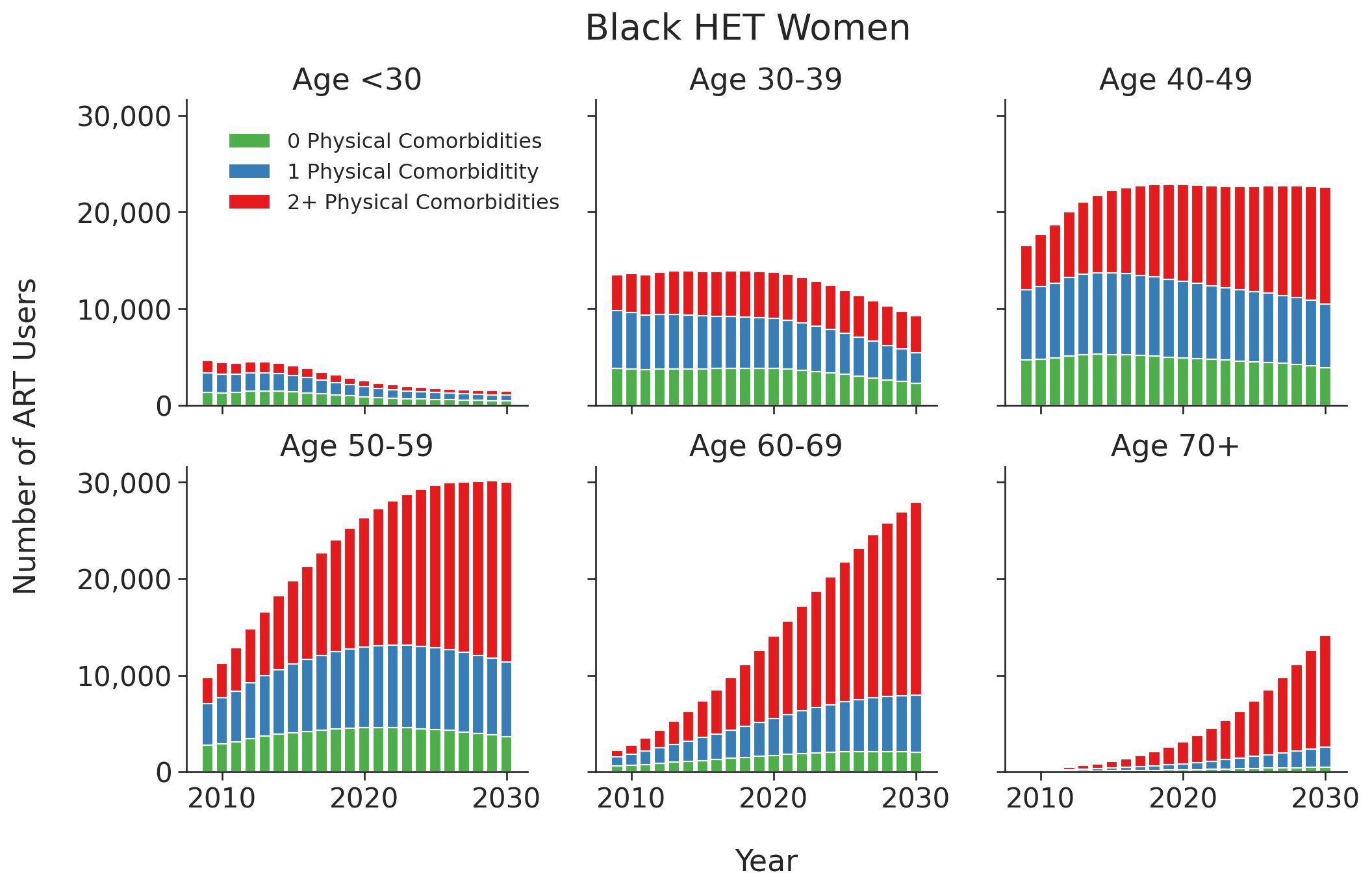
**

S3p) Hispanic heterosexual women

**
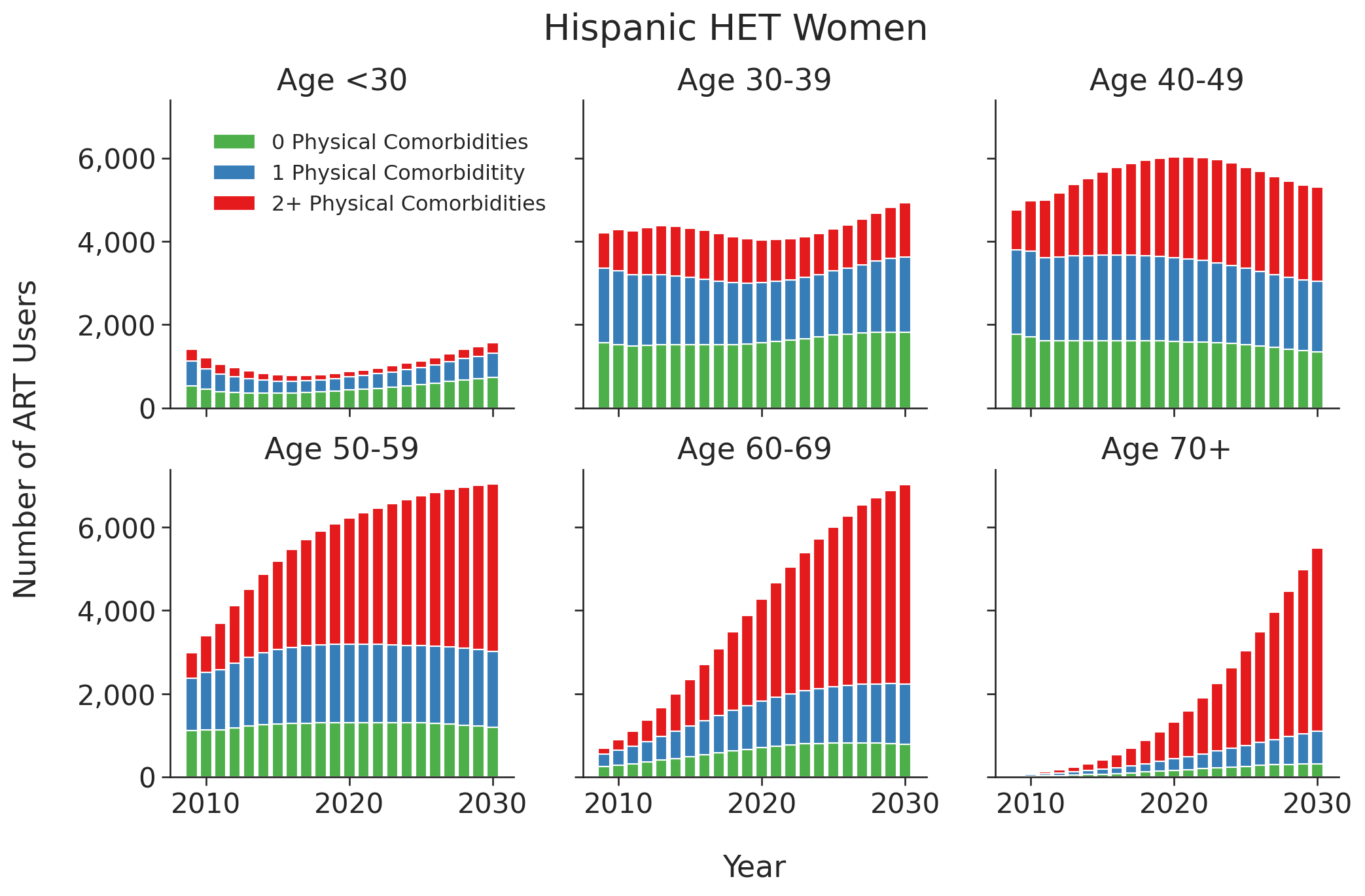
**
