# Supplementary material for: The forecasted prevalence of comorbidities and multimorbidity in people with HIV in the United States through the year 2030: A modeling study
Source: PLoS Med. 2024 Jan 12;21(1):e1004325. doi: 10.1371/journal.pmed.1004325 (PMC10833859; doi:10.1371/journal.pmed.1004325)
Supplement: S5 Fig — Forecasted prevalence (and shaded 95% credibility intervals) of individual comorbidities within the 15 subgroups of people with HIV: (a) White, (b) Black/African American, and (c) Hispanic men who have sex with men; (d) White, (e) Black/African American, and (f) Hispanic men with injection drug use as their HIV acquisition risk factor; (g) White, (h) Black/African American, and (i) Hispanic women with injection drug use as their HIV acquisition risk factor; (j) White, (k) Black/African American, and (l) Hispanic heterosexual men; (m) White, (n) Black/African American, and (o) Hispanic heterosexual women. (DOCX) [file pmed.1004325.s005.docx]

**S5 Figure:** Forecasted prevalence (and shaded 95% credibility intervals) of individual comorbidities within the 15 subgroups of people with HIV: a) White, b) Black/African American, and c) Hispanic men who have sex with men; d) White, e) Black/African American, and f) Hispanic men with injection drug use as their HIV acquisition risk factor; g) White, h) Black/African American, and i) Hispanic women with injection drug use as their HIV acquisition risk factor; j) White, k) Black/African American, and l) Hispanic heterosexual men; m) White, n) Black/African American, and o) Hispanic heterosexual women

S4a) White men who have sex with men


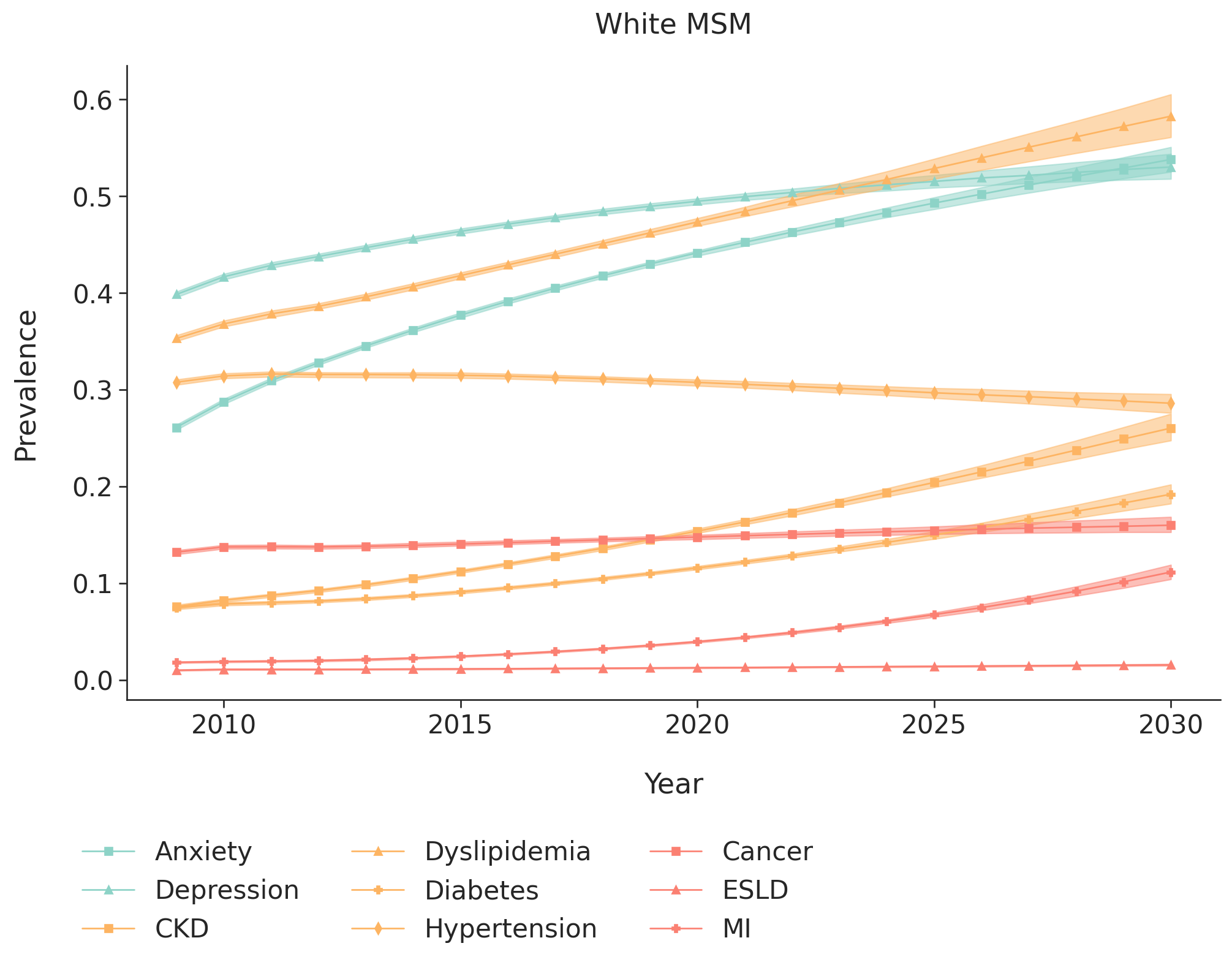


S4b) Black/African American men who have sex with men


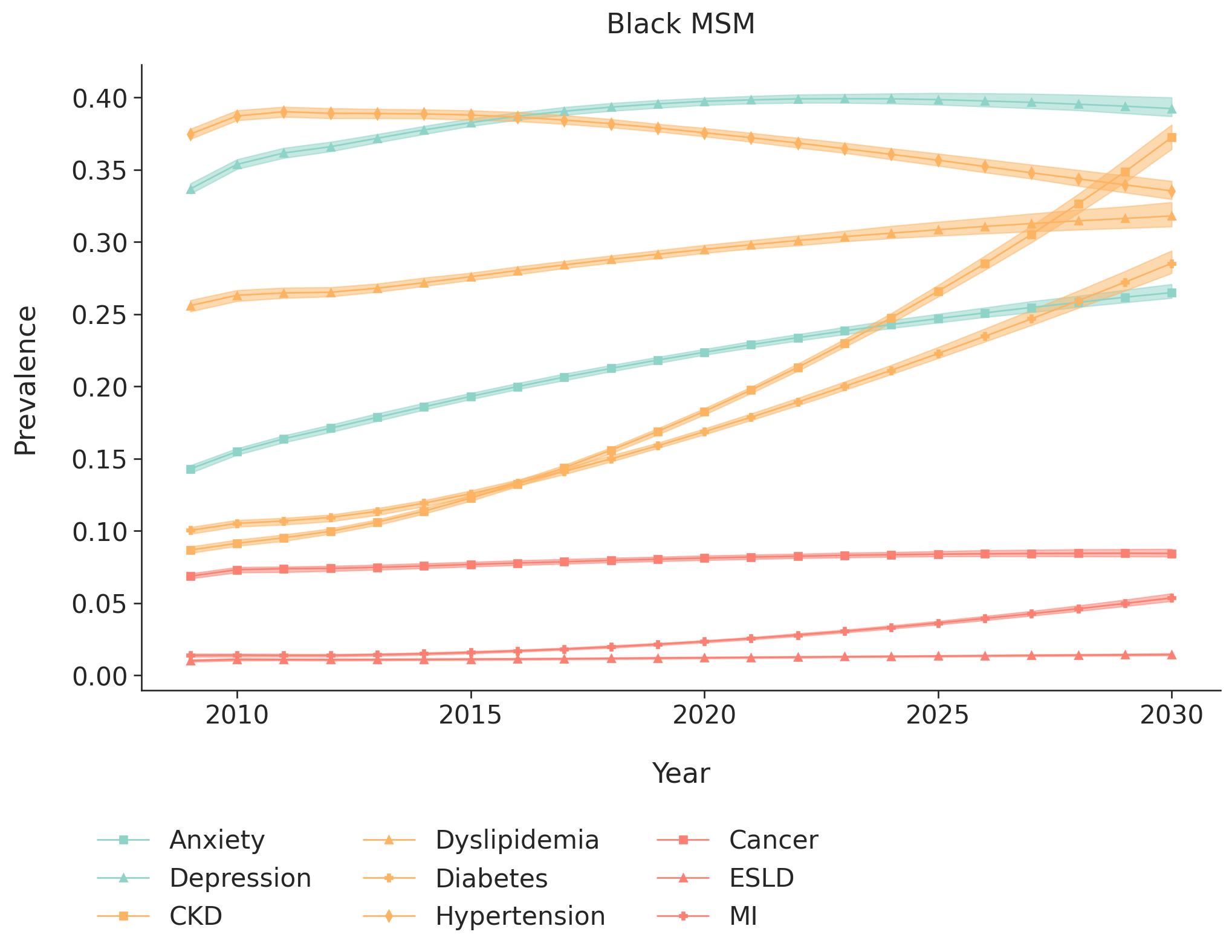


S4c) Hispanic men who have sex with men


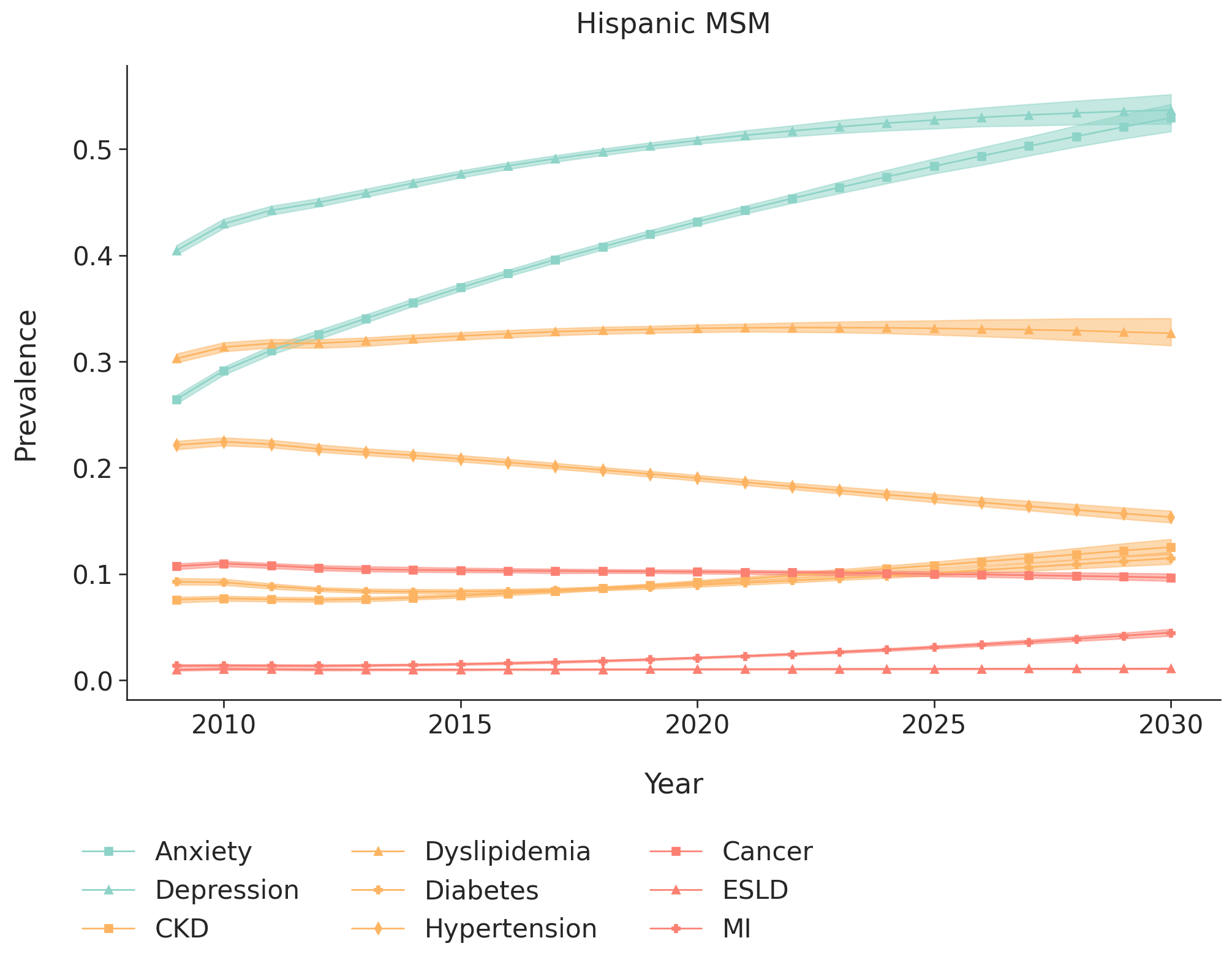


S4d) White men with injection drug use as their HIV acquisition risk factor


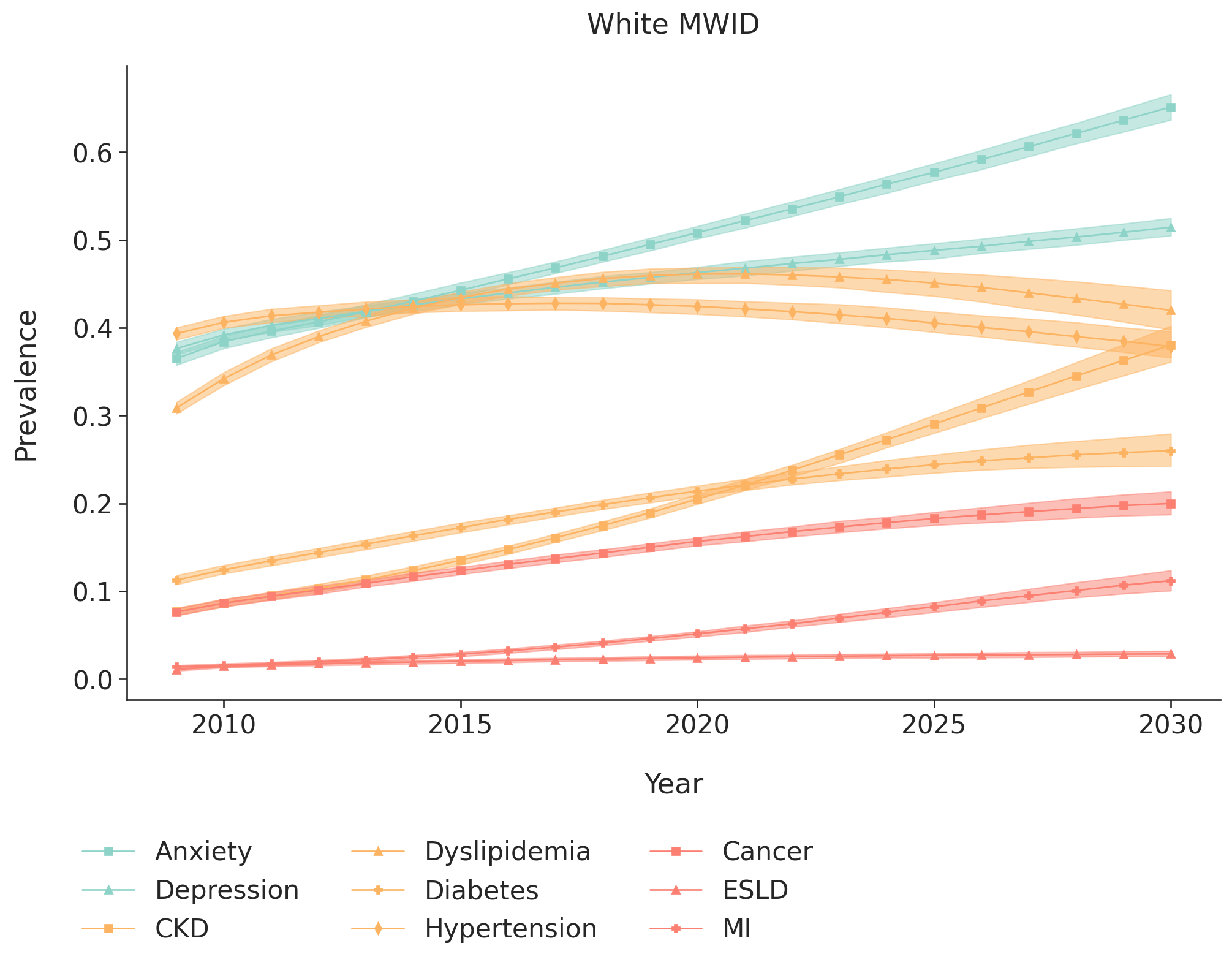


S4e) Black/African American men with injection drug use as their HIV acquisition risk factor


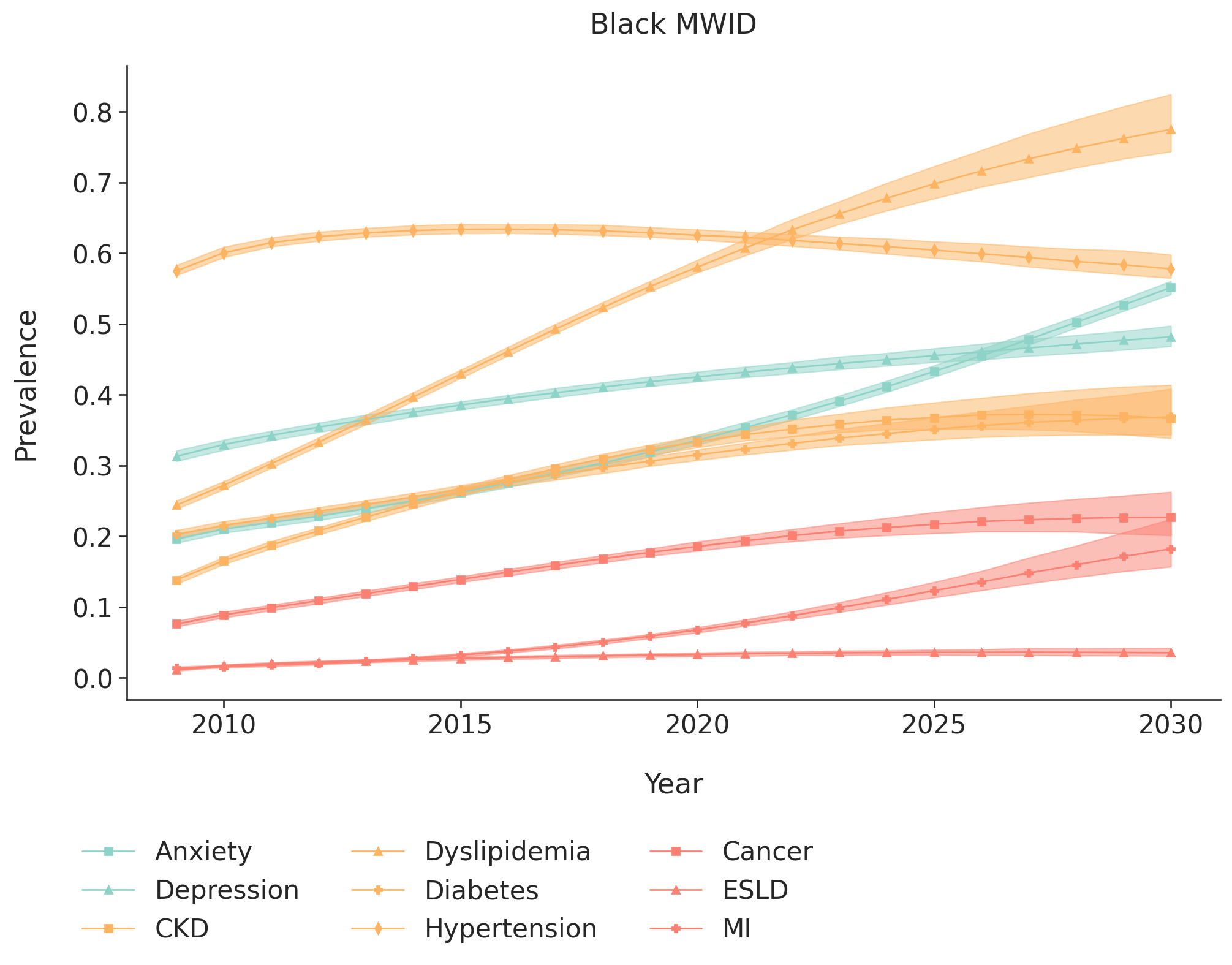


S4f) Hispanic men with injection drug use as their HIV acquisition risk factor


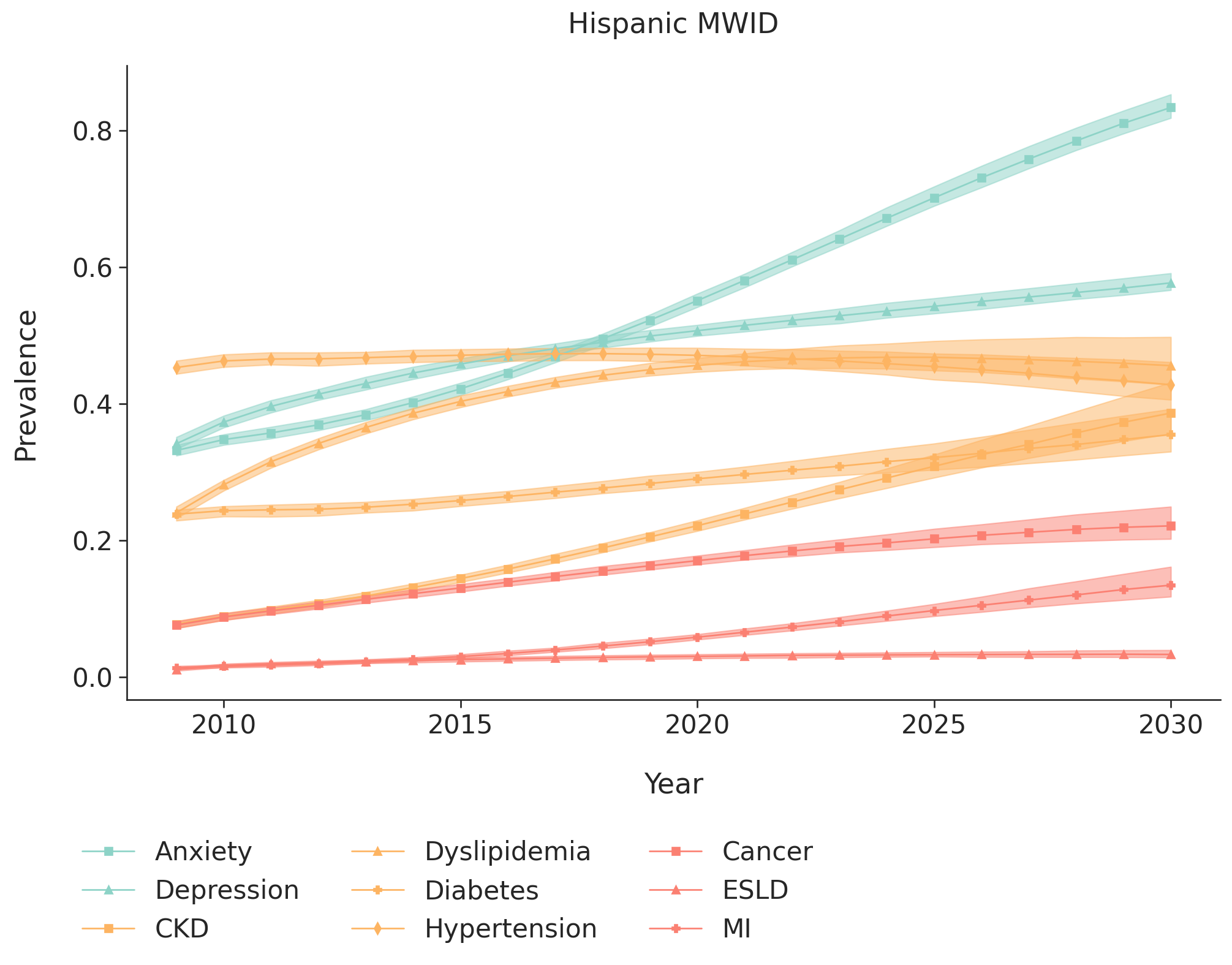


S4g) White women with injection drug use as their HIV acquisition risk factor


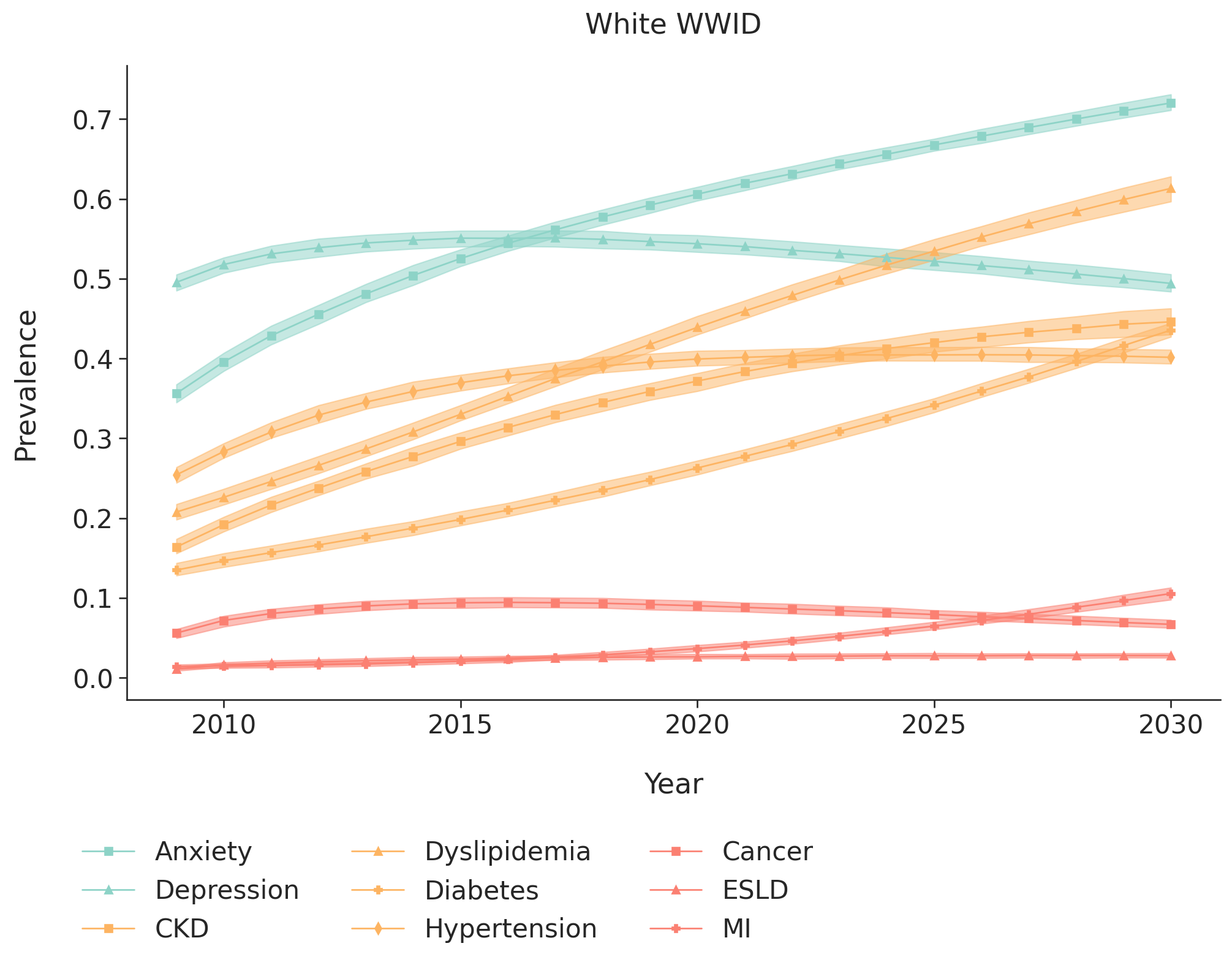


S4h) Black/African American women with injection drug use as their HIV acquisition risk factor


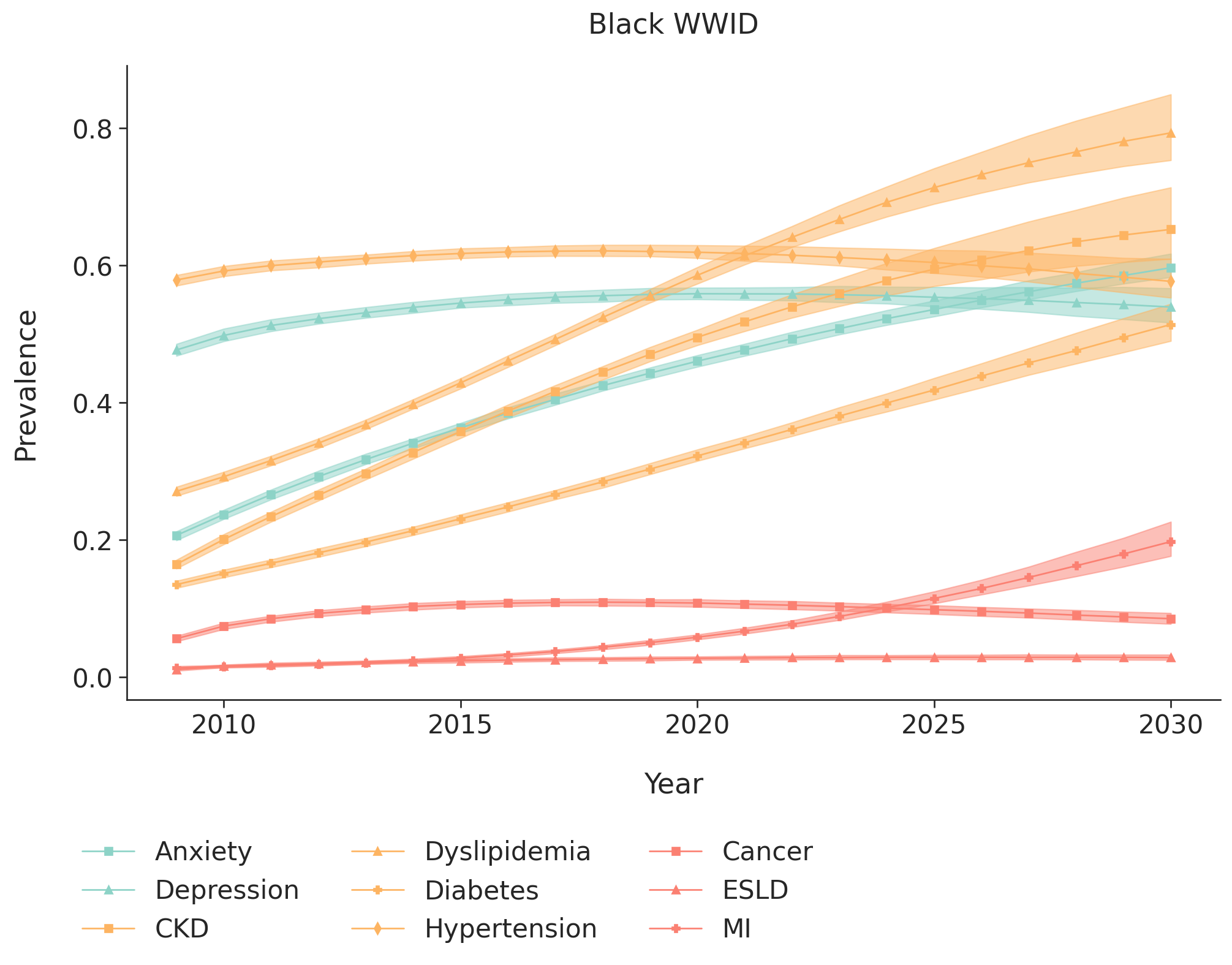


S4i) Hispanic women with injection drug use as their HIV acquisition risk factor


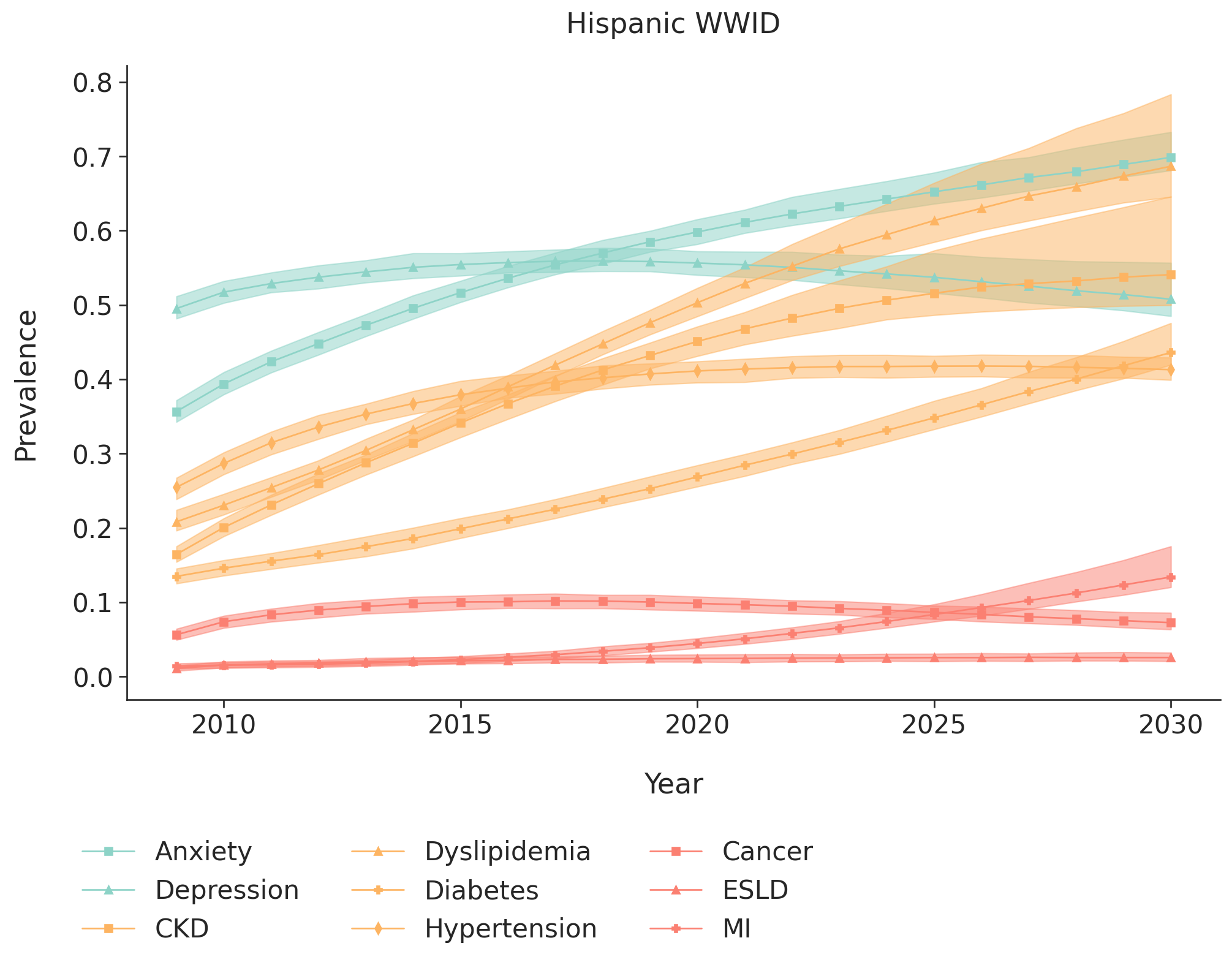


S4j) White heterosexual men


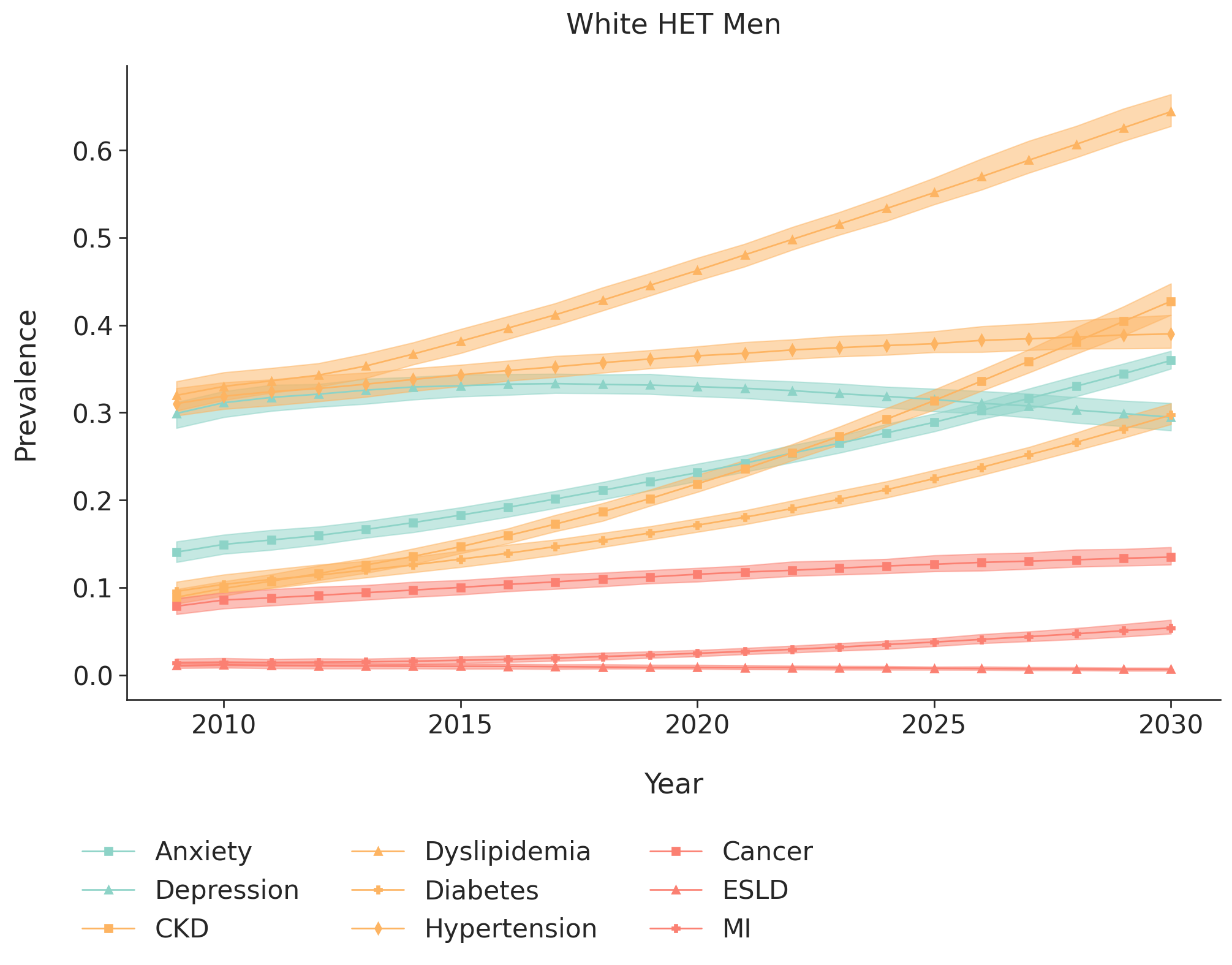


S4k) Black/African American heterosexual men


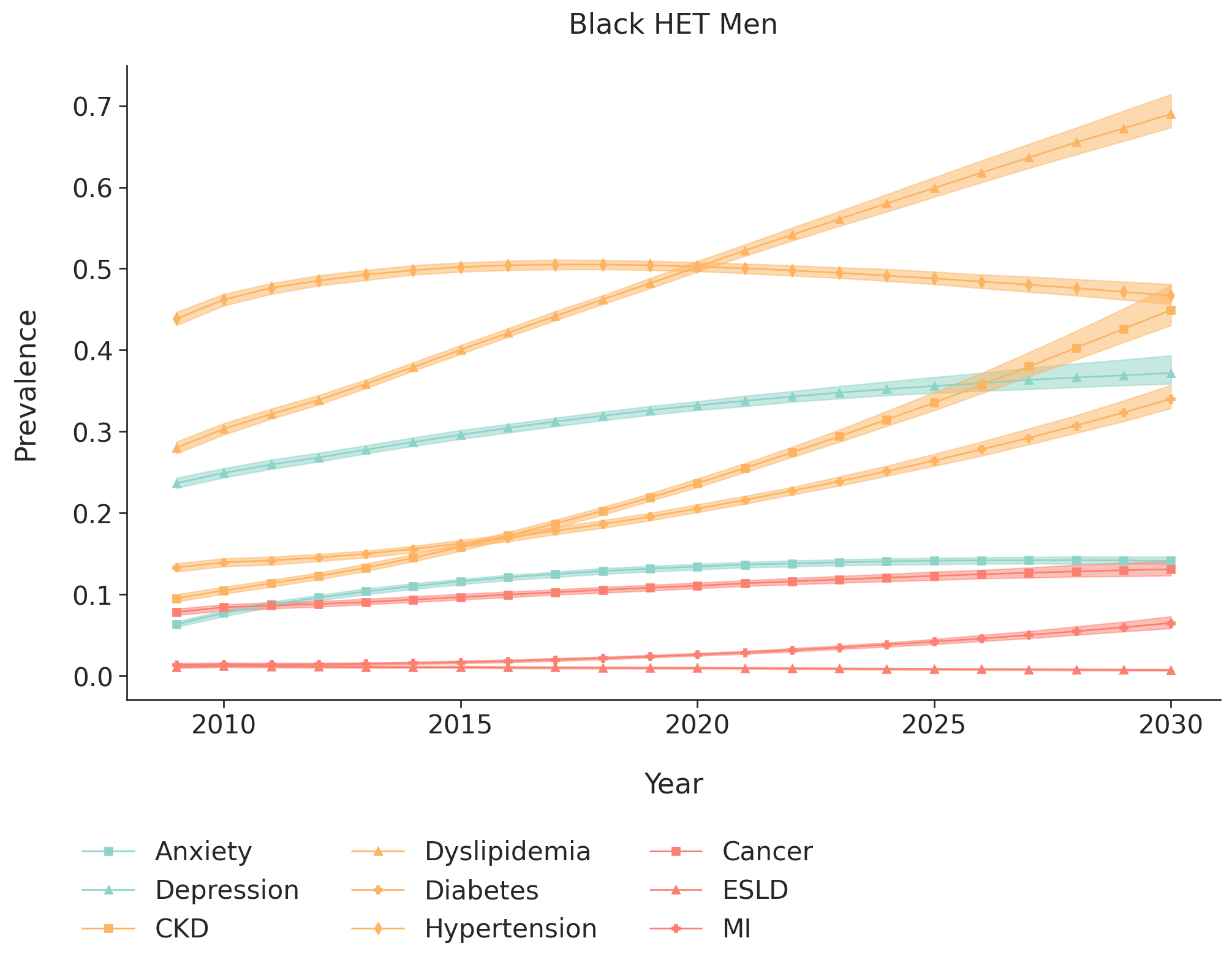


S4l) Hispanic heterosexual men


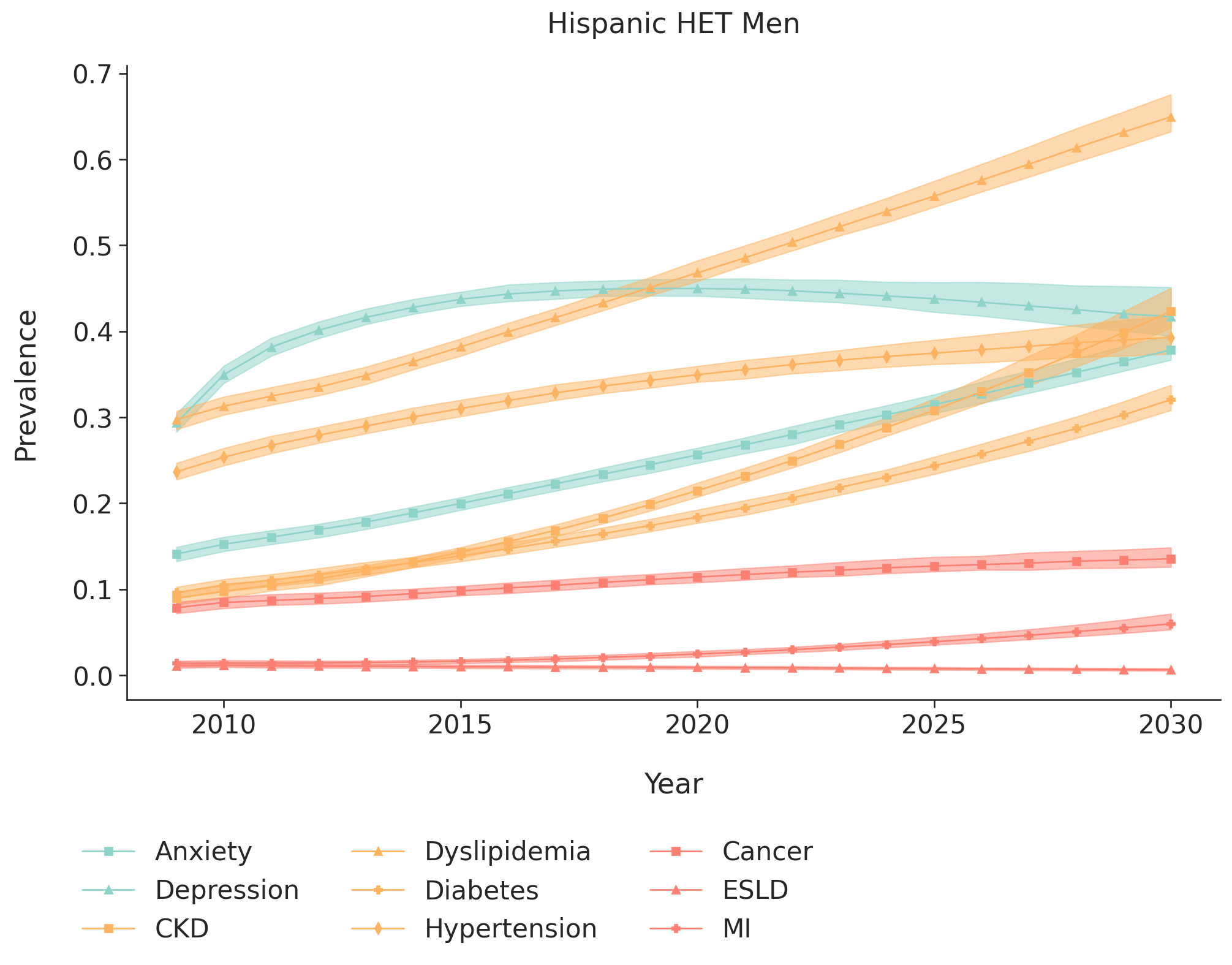


S4m) White heterosexual women


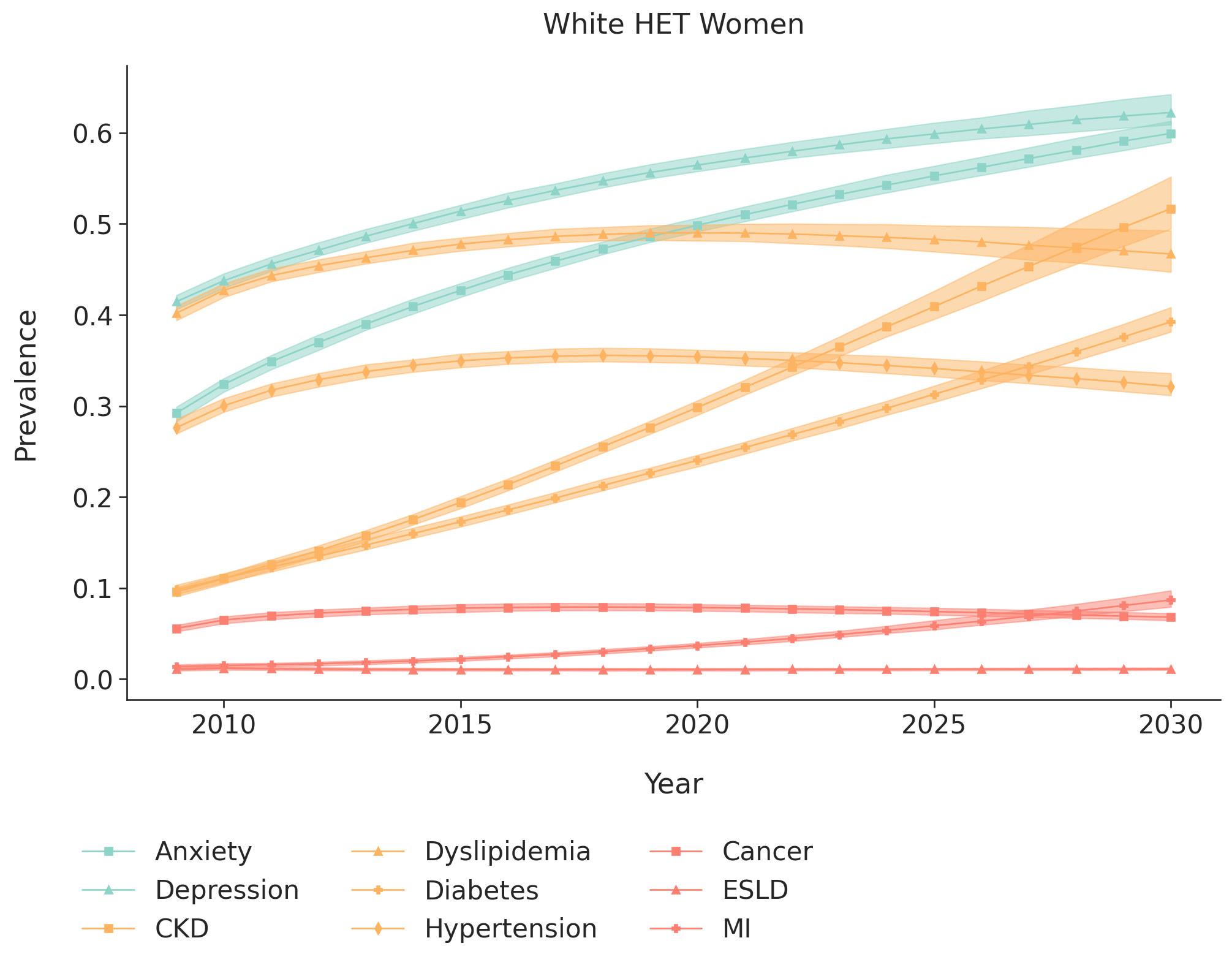


S4n) Black/African American heterosexual women


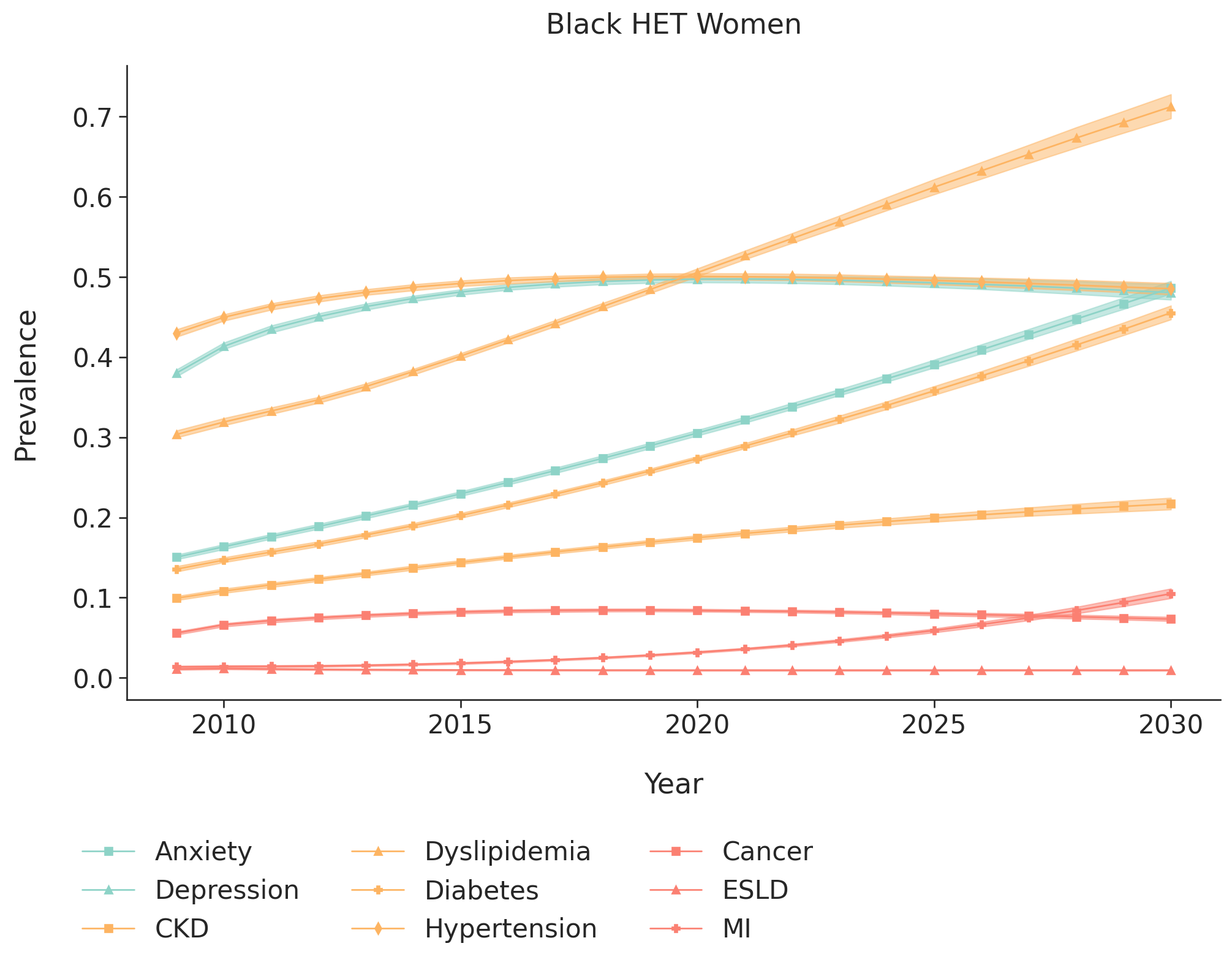


S4o) Hispanic heterosexual women


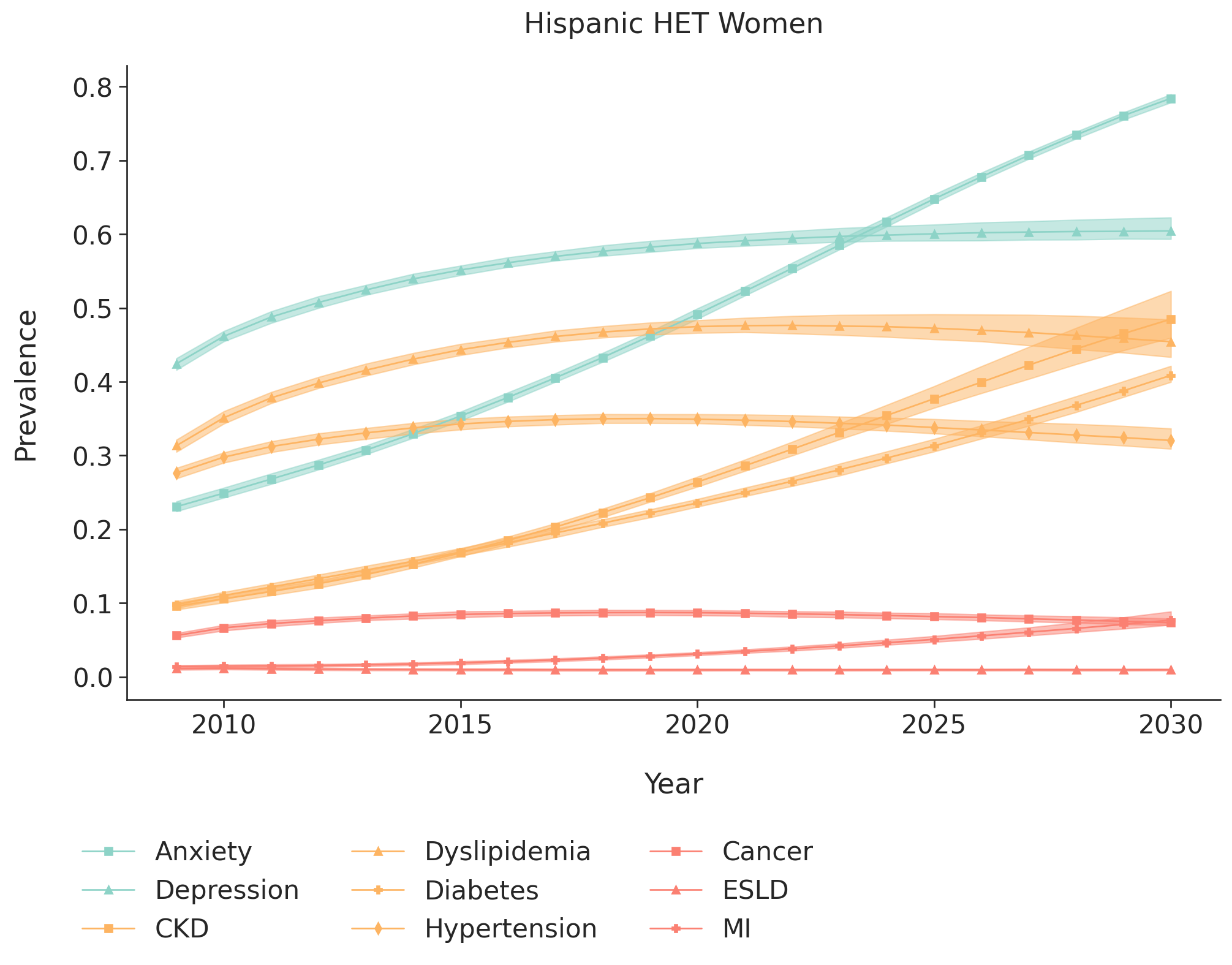


Footnotes:

CKD=stage ≥3 chronic kidney disease

ESLD=end-stage renal disease

MI=myocardial infarction

The 95% interquartile range is estimated as the 2.5% and 97.5% range of results from running the simulation 200 times (also called the 95% uncertainty range).
