# Supplementary material for: The forecasted prevalence of comorbidities and multimorbidity in people with HIV in the United States through the year 2030: A modeling study
Source: PLoS Med. 2024 Jan 12;21(1):e1004325. doi: 10.1371/journal.pmed.1004325 (PMC10833859; doi:10.1371/journal.pmed.1004325)

Various candidate models were proposed to predict the number of new HIV diagnoses from 2006 to 2030. Predicted HIV diagnoses prior to 2010 are used later in the initial 2009 population creation. After removing models with inadequate fit (based on AIC values), the data were fit using a Poisson model, a gamma model and a natural cubic spline model with a single knot. Of these models, those resulting in unrealistic forecasts (>50% increase in new diagnosis from 2020 – 2030) were also removed. The Poisson and gamma fits were accomplished using the glm function of the stats package in base R, while the spline fit was generated using the lm and ns functions of the base R packages stats and splines, respectively. To incorporate additional uncertainties in annual estimates, the 95% prediction intervals around each fit were calculated. These prediction intervals were combined to generate an annual range for the number of new diagnoses in each year for a given subgroup (black, white and Hispanic). The annual ranges are estimated from the largest upper prediction interval and the lowest lower prediction interval of existing models. For each simulation run, a random number between 0 and 1 is drawn that defines the number of new diagnoses in that simulation.

**S6 Fig**: Ranges used to generate the number of new diagnoses, by HIV acquisition risk groups and race and ethnicity: a) heterosexual women; b) heterosexual men; c) women who injected drugs; d) men who injected drugs; e) men who have sex with men

S5a) Heterosexual women


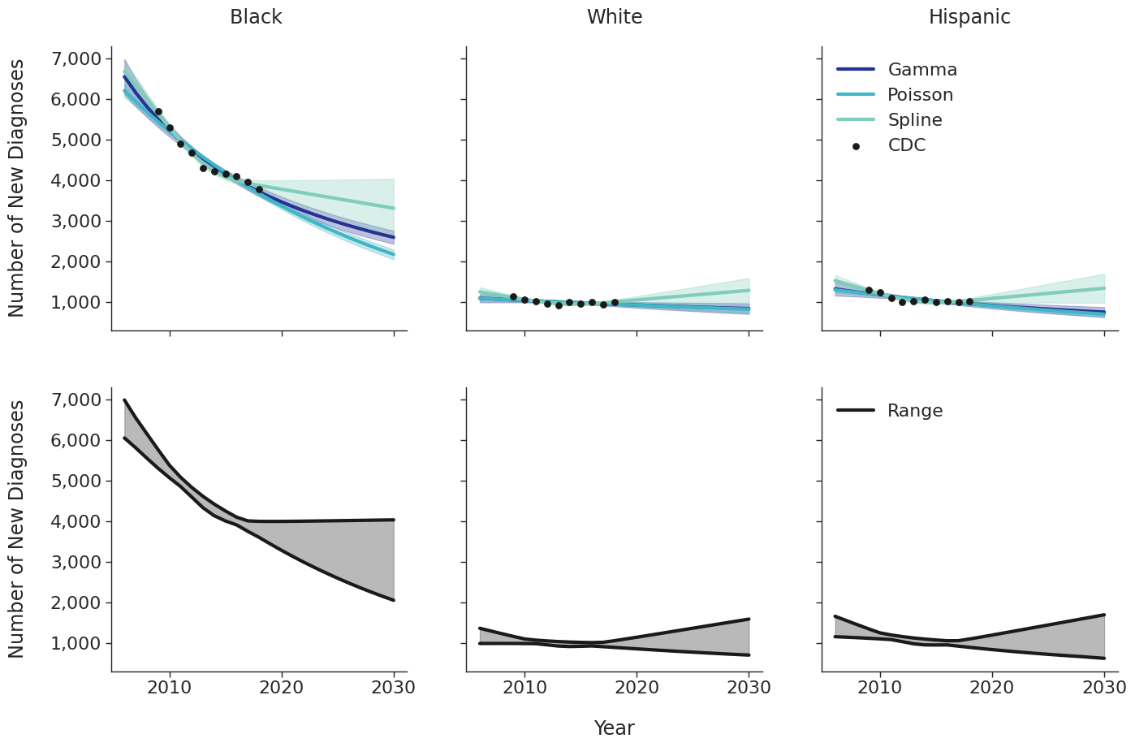


S5b) Heterosexual men


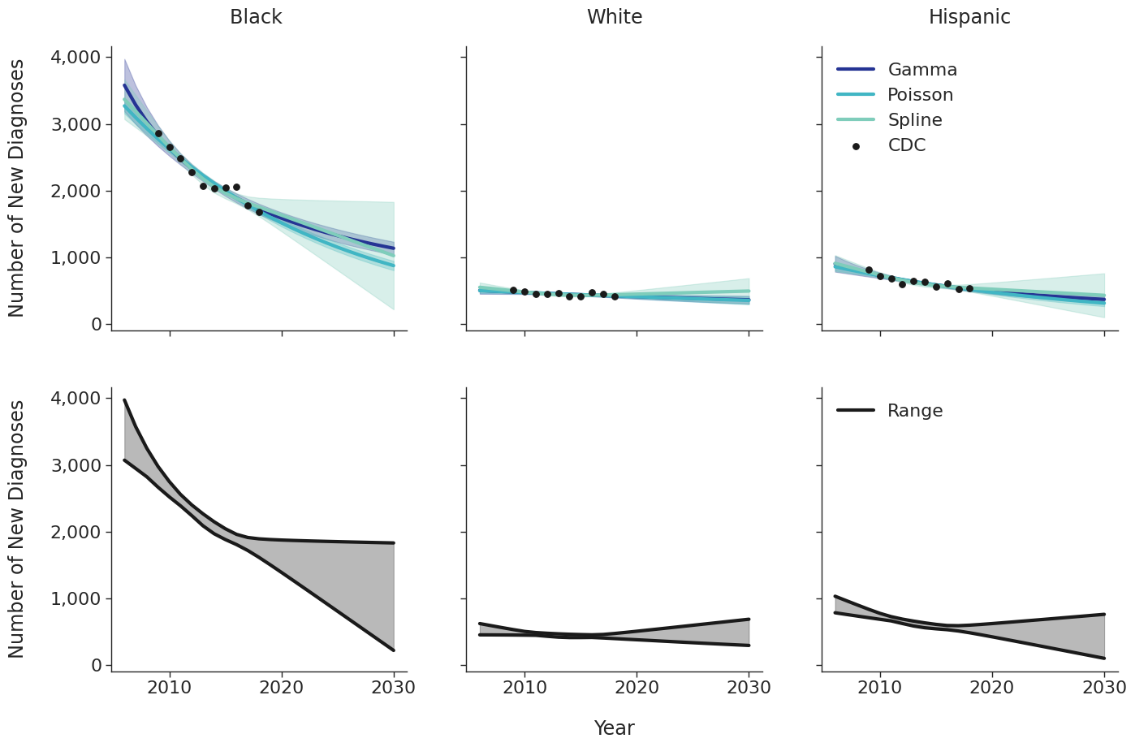


S5c) Women who have injected drugs


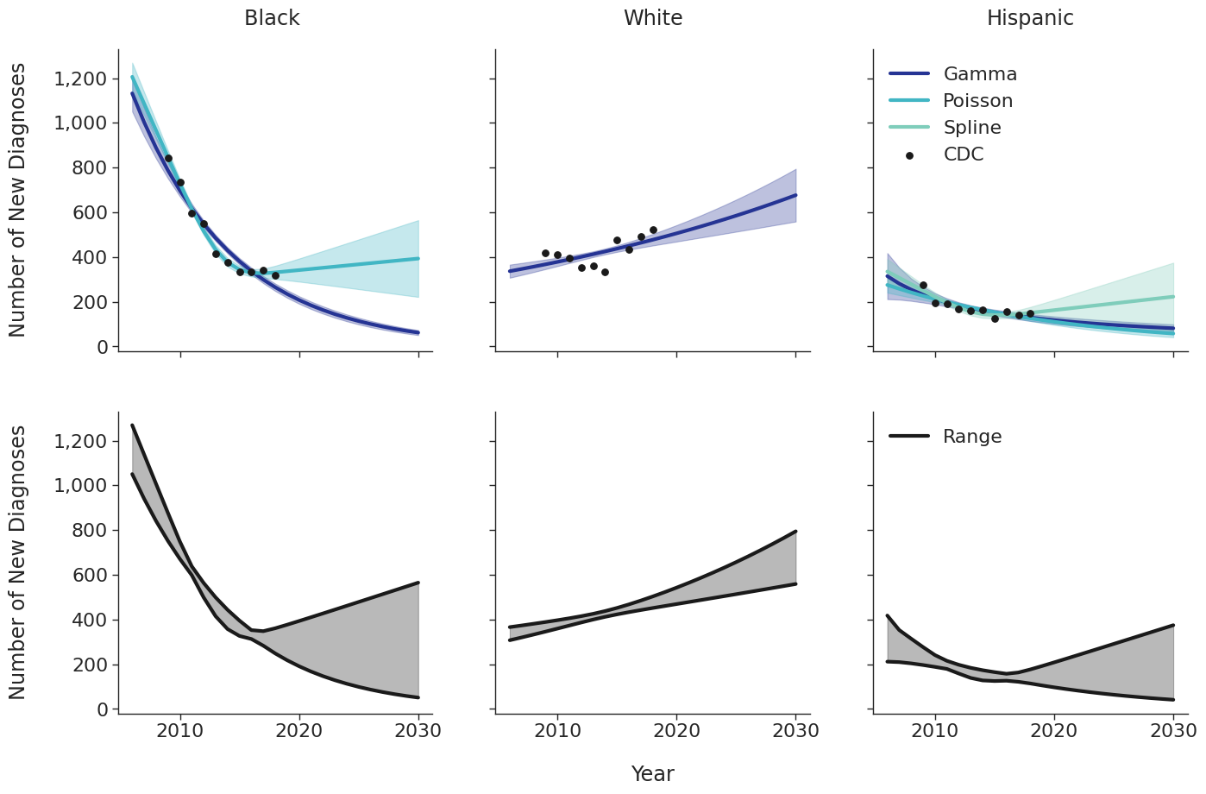


S5d) Men who have injected drugs


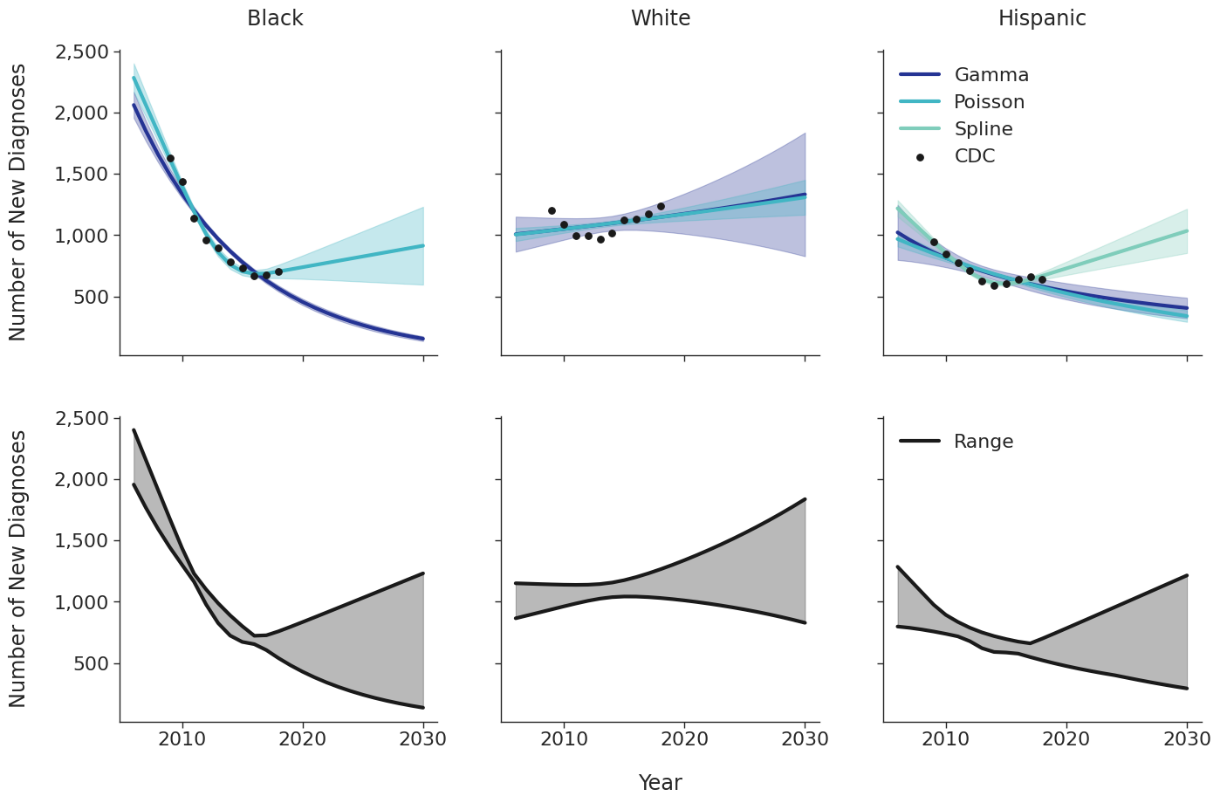


S5e) Men who have sex with men


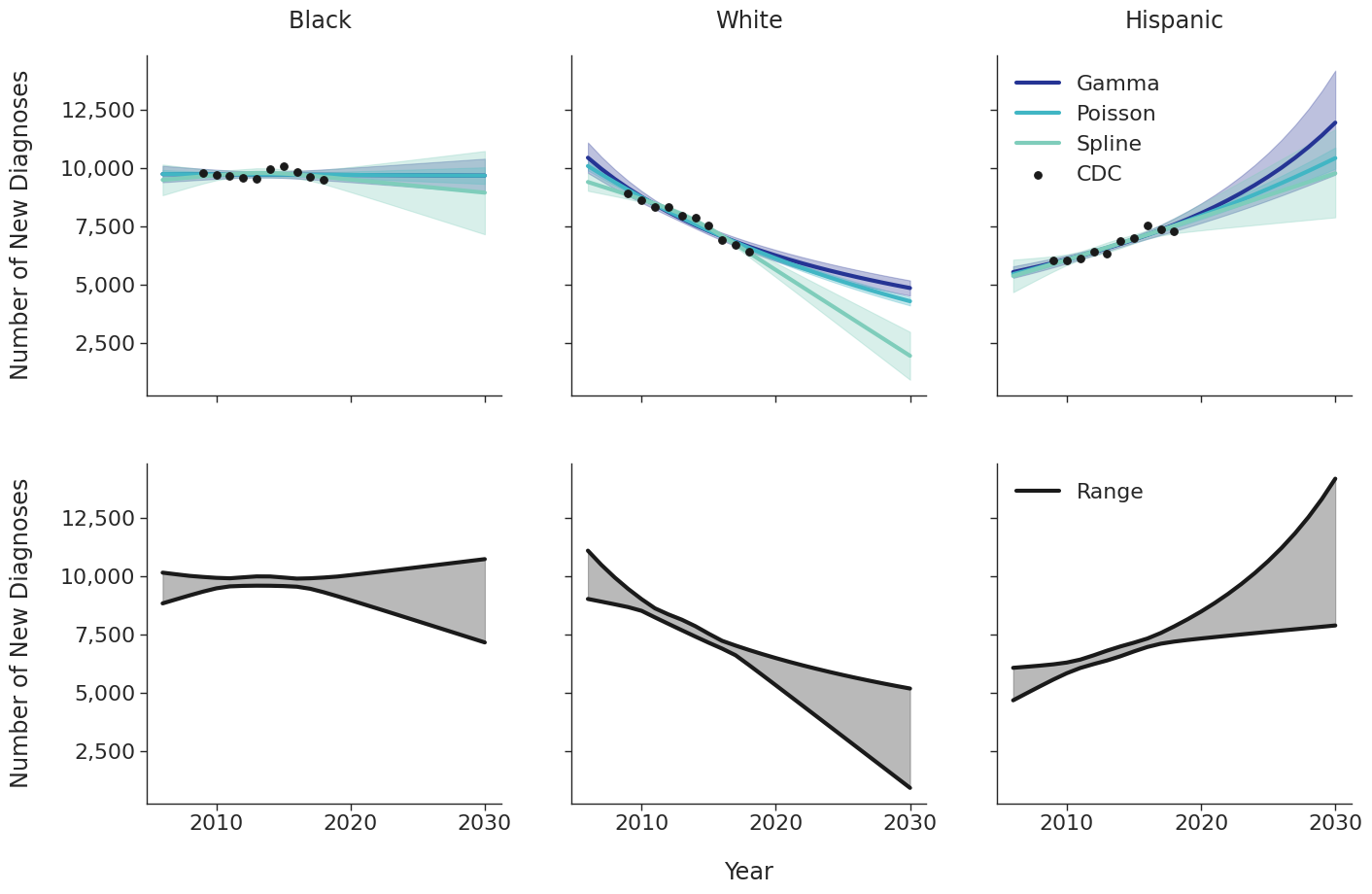

Supplement: S6 Fig — Ranges used to generate the number of new diagnoses, by HIV acquisition risk groups and race and ethnicity: (a) heterosexual women; (b) heterosexual men; (c) women who injected drugs; (d) men who injected drugs; (e) men who have sex with men. (DOCX) [file pmed.1004325.s006.docx]
