# Supplementary material for: The forecasted prevalence of comorbidities and multimorbidity in people with HIV in the United States through the year 2030: A modeling study
Source: PLoS Med. 2024 Jan 12;21(1):e1004325. doi: 10.1371/journal.pmed.1004325 (PMC10833859; doi:10.1371/journal.pmed.1004325)
Supplement: S7 Fig — Forecasted percentage of people linking to HIV care by HIV acquisition risk groups and race and ethnicity: (a) heterosexual females; (b) heterosexual males; (c) women who injected drugs; (d) men who injected drugs; (e) men who have sex with men. (DOCX) [file pmed.1004325.s007.docx]

**Linkage to HIV care and ART initiation:** We estimated percentage of people in each sub-population linking to HIV care in the first 4 months after HIV diagnosis for each year between 2010 – 2015 from the CDC. To forecast future trends from 2016 to 2030, we applied a linear regression and capped at 95% linkage as shown in **Figure S7**. The linear regression was accomplished using the ols function of the statsmodels package for Python. Among remaining cases, we further assumed that 40% link to care over the next three years after initial diagnosis. To estimate the population starting ART, we assumed that 70% of those linking to care begin ART immediately in years prior to 2011. This percentage rises to 85% in 2011 and up to 97% thereafter.

**S7 Fig:** Forecasted percentage of people linking to HIV care by HIV acquisition risk groups and race and ethnicity: a) heterosexual females; b) heterosexual males; c) women who injected drugs; d) men who injected drugs; e) men who have sex with men

S6a) Heterosexual women


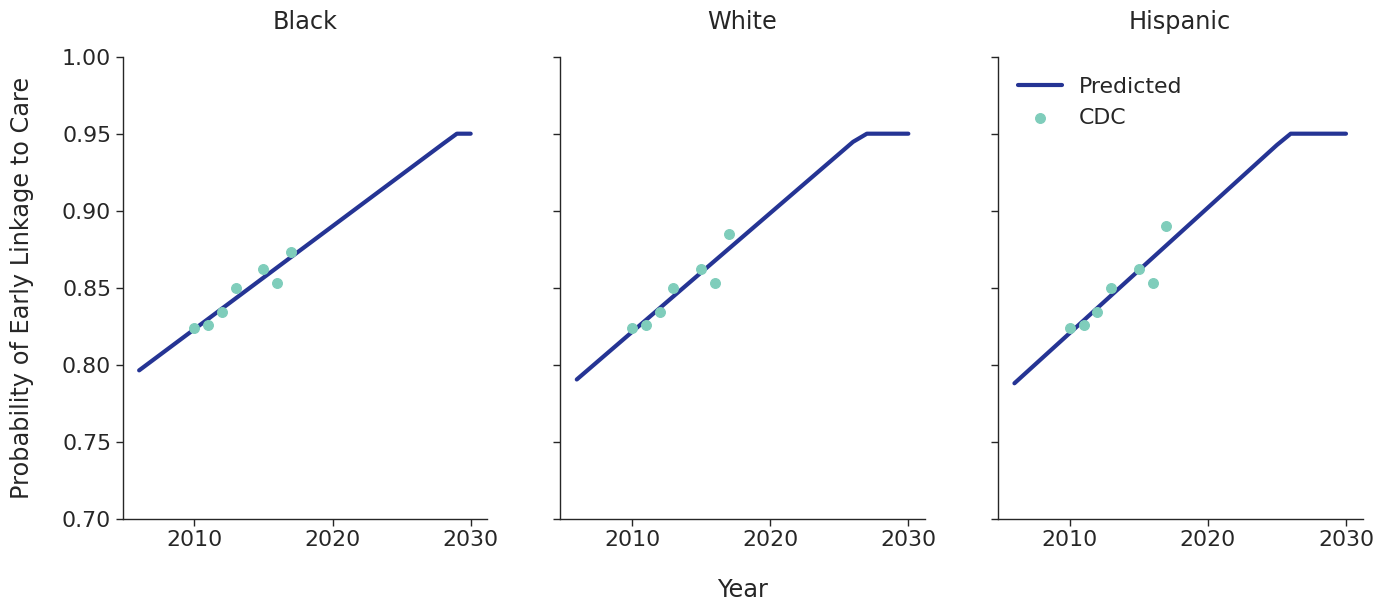


S6b) Heterosexual men

**
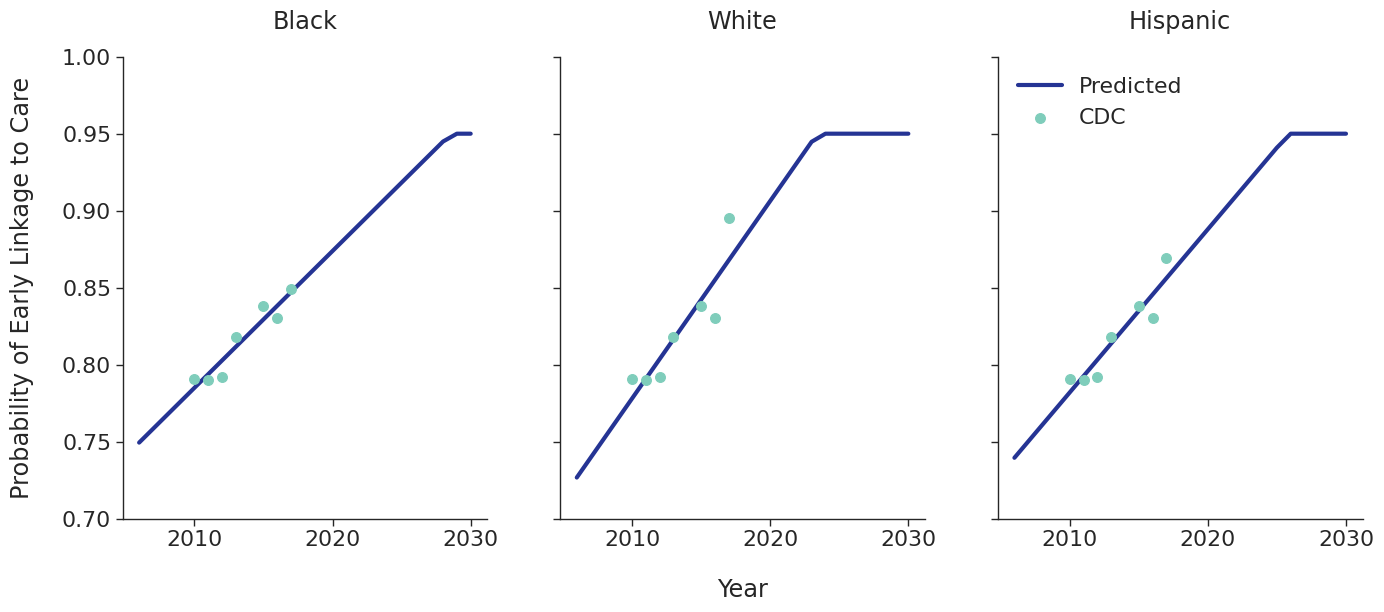
**

S6c) Women who injected drugs

**
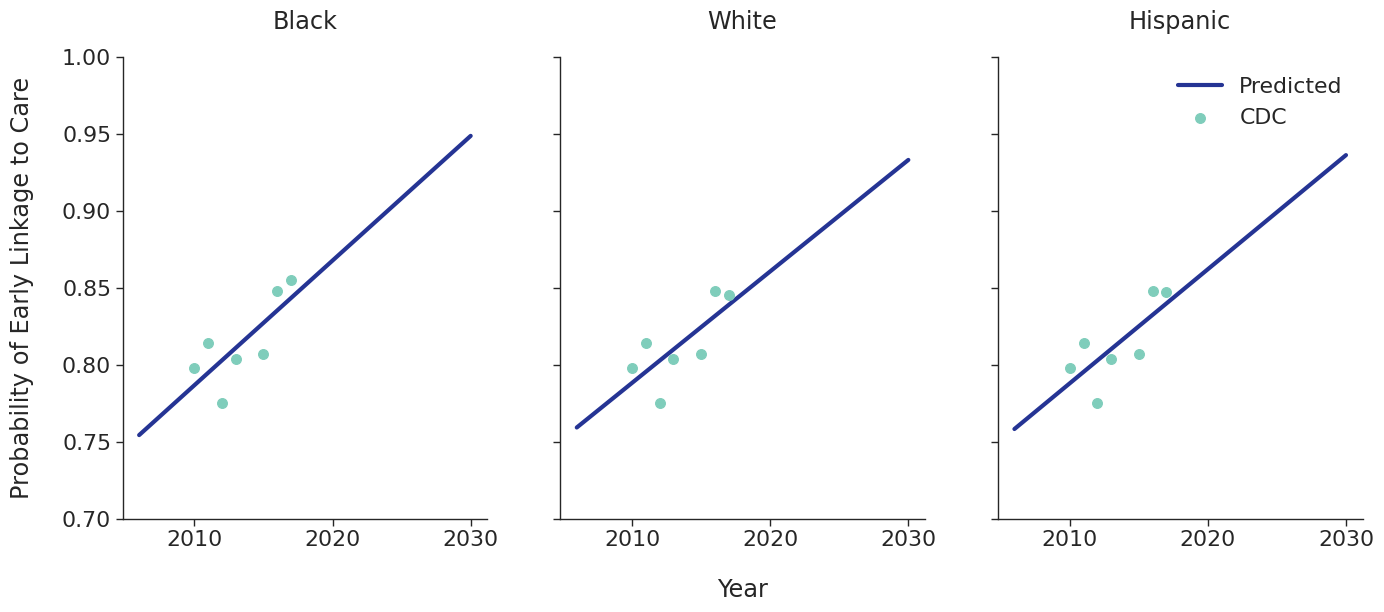
**

D6d) Men who injected drugs

**
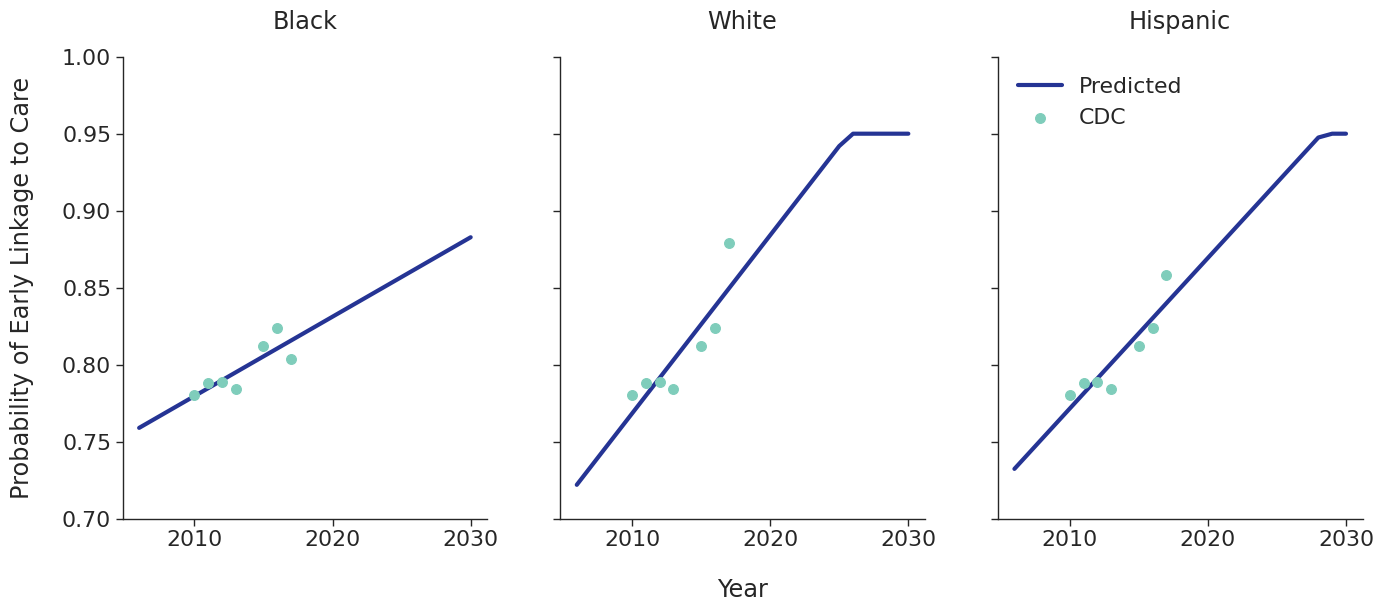
**

D6e) Men who have sex with men **
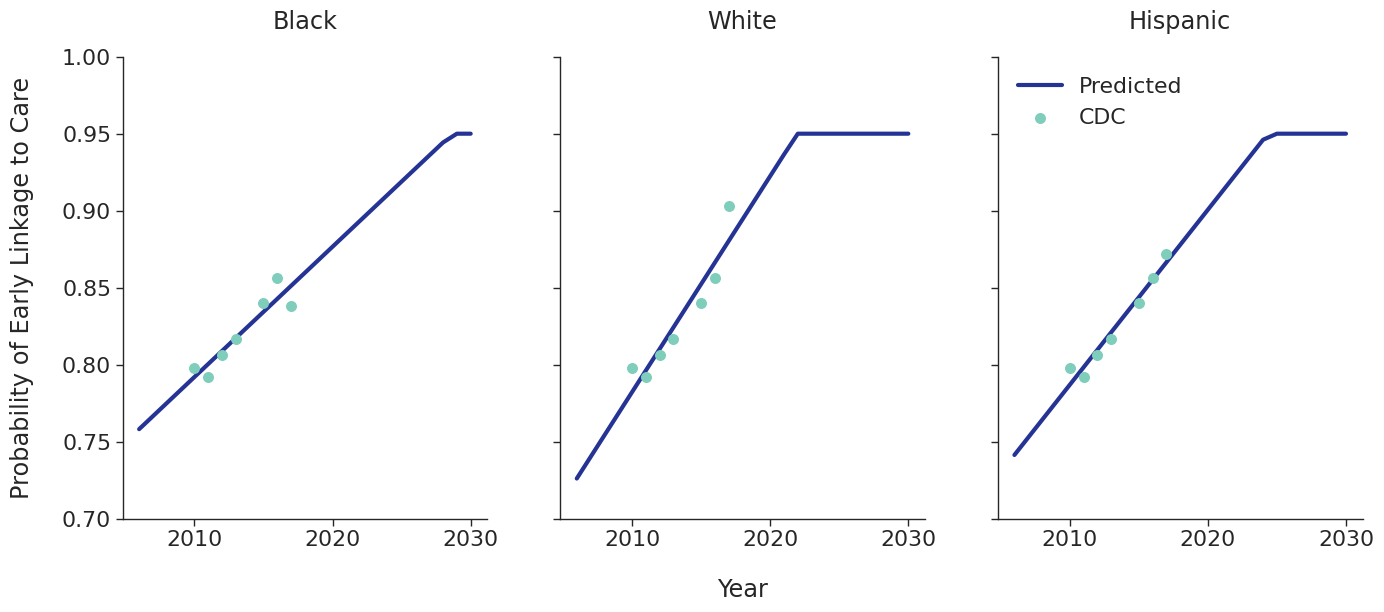
**
