# Supplementary material for: The forecasted prevalence of comorbidities and multimorbidity in people with HIV in the United States through the year 2030: A modeling study
Source: PLoS Med. 2024 Jan 12;21(1):e1004325. doi: 10.1371/journal.pmed.1004325 (PMC10833859; doi:10.1371/journal.pmed.1004325)
Supplement: S1 Table — (DOCX) [file pmed.1004325.s008.docx]

**S1 Table:** Definitions of highly prevalent risk factors and comorbidities measured in the NA-ACCORD and included in the PEARL model

| **Risk factor or comorbidity** | **Data source** | **Definition** |
| --- | --- | --- |
| Smoking | EHR and patient-reported surveys (when available) | Ever having EHR-based evidence or patient-reported smoking (collected via surveys) while under observation |
| Obesity | EHR | Body mass index ≥30 kg/m2 (height and weight measurements are exported from EHR) |
| Hepatitis C virus infection | EHR | Ever having EHR-based evidence of HCV infection while under observation, defined as:  1) a positive HCV antibody test, OR  2) a detectable HCV RNA, OR  3) the presence of an HCV genotype test |
| Depression^1^ | EHR | An absorbing state from the month/day/year an individual has a major depressive disorder diagnosis. |
| Anxiety^1^ | EHR | An absorbing state from the month/day/year an individual has a generalized anxiety disorder diagnosis |
| Dyslipidemia^2^ | EHR | An absorbing state from the month/day/year an individual first meets the following definition:  1) total cholesterol ≥240 mg/dL, OR  2) HDL ≤40 mg/dL for men or ≤50 for women, OR  3) LDL ≥130 mg/dL, OR  4) lipid-lowering medication prescription |
| Hypertension^3^ | EHR | An absorbing state from the month/day/year an individual first meets the following definition:  1) prescription of anti-hypertension medications, AND  2) a hypertension diagnosis |
| Stage ≥3 Chronic Kidney Disease^3^ | EHR | An absorbing state from the month/day/year an individual first meets the following definition: ever had eGFR <60 mL/min/1.73m2 consistently for at least 3 months  eGFR estimated using CKD-Epi equation that included an indicator for race. |
| Diabetes^3^ | EHR | An absorbing state from the month/day/year an individual first meets the following definition:  1) HgA1c >6.5%, OR  2) diabetes specific medication was prescribed OR  3) a Type 2 or unspecified diabetes diagnosis was recorded, and diabetes-related medications were prescribed |
| Myocardial infarction^4^ | EHR with validation by cardiologists | An absorbing state from the month/day/year an individual who screens positive for a potential MI event has a validated MI event (regardless of MI type) as determined by an adjudication process.  Once an MI event is validated, no subsequent events are investigated. |
| Cancer (all types)^5^ | Cancer registries or EHR data that is validated for cancer diagnosis | An absorbing state from the month/day/year an individual first meets the following definition:  1) Validated cancer diagnosis (all types) confirmed in a cancer registry  2) Validated cancer diagnosis (all types) confirmed with review of the patient’s EHR following screening positive for a potential cancer diagnosis |
| End-stage Renal Disease (ESLD)^6^ | EHR data that is validated for cancer diagnosis | An absorbing state from the month/day/year an individual who screens positive for a potential ESLD event has a validated MI event as determined with review of the patient’s EHR |

Abbreviations:

EHR=electronic health record

1. Lang R, et al. The prevalence of mental health disorders in people with HIV and the effects on the HIV Care Continuum. *AIDS*. 2022;*In press.*

2. Drozd D, et al. Increased risk of myocardial infarction in HIV-infected individuals in North American compared with the general population. *Journal of Acquired Immune Deficiency Syndrome*. 2017 Aug 15;75(5):568-576.

3. Wong C, et al. First occurrence of diabetes, chronic kidney disease, and hypertension among North American HIV-infected adults, 2000-2013. *Clinical Infectious Diseases*. 2017 Feb 15;64(4):459-467.

4. Crane HM, et al. Lessons learned from the design and implementation of myocardial infarction adjudication tailored for HIV clinical cohorts. *American Journal of Epidemiology*. 2014 Apr 15;179(8):996-1005.

5. Silverberg MJ, et al. Cumulative incidence of cancer among persons with HIV in North America: A cohort study*. Annals of Internal Medicine*. 2015 Oct 6;163(7):507-518.

6. Kitahata MM, et al. Ascertainment and verification of end-stage renal disease and end-stage liver disease in the North American AIDS Cohort Collaboration on Research and Design. *AIDS Research and Treatment.* 2015:923194.
