# Supplementary material for: The forecasted prevalence of comorbidities and multimorbidity in people with HIV in the United States through the year 2030: A modeling study
Source: PLoS Med. 2024 Jan 12;21(1):e1004325. doi: 10.1371/journal.pmed.1004325 (PMC10833859; doi:10.1371/journal.pmed.1004325)
Supplement: S2 Table — (DOCX) [file pmed.1004325.s009.docx]

**S2 Table:** Prevalence and incidence functions applied to PEARL agents who have initiated ART for a) anxiety prevalence, b) anxiety incidence, c) depression prevalence, d) depression incidence, e) stage ≥3 chronic kidney disease prevalence, f) stage ≥3 chronic kidney disease incidence, g) dyslipidemia prevalence, h) dyslipidemia incidence, i) diabetes prevalence, j) diabetes incidence, k) hypertension prevalence, l) hypertension incidence, m) cancer prevalence, n) cancer incidence, o) end-stage liver disease prevalence, p) end-stage liver disease incidence, q) myocardial infarction prevalence, and r) myocardial infarction incidence

S2a) Anxiety prevalence estimates (from the NA-ACCORD)
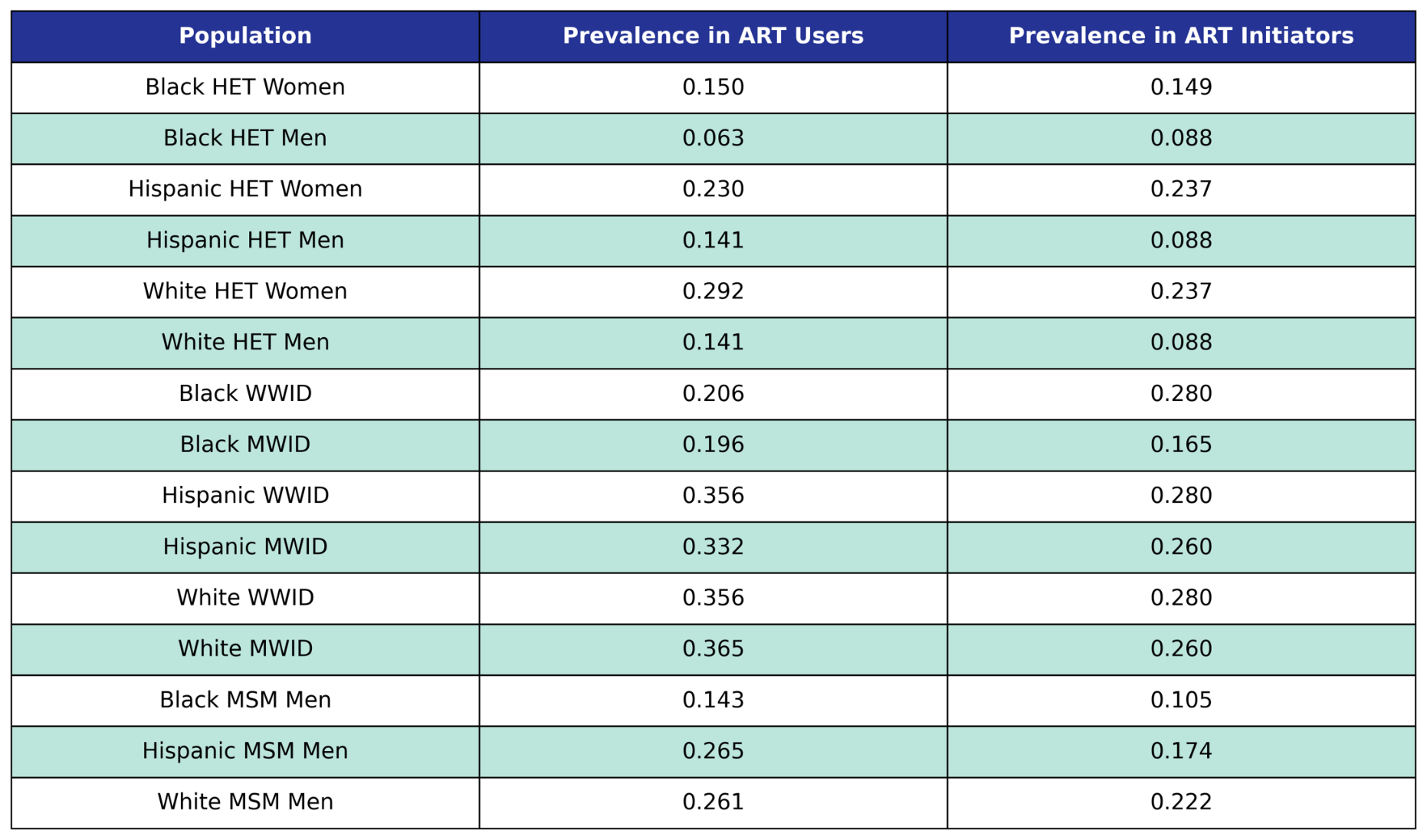


Prevalence in the 2009 ART user population is taken from the 2009 NA-ACCORD population, while prevalence in ART initiators was taken from the 2009 - 2017 NA-ACCORD ART initiator population.

S2b) Coefficient estimates from anxiety incidence functions (from the NA-ACCORD)


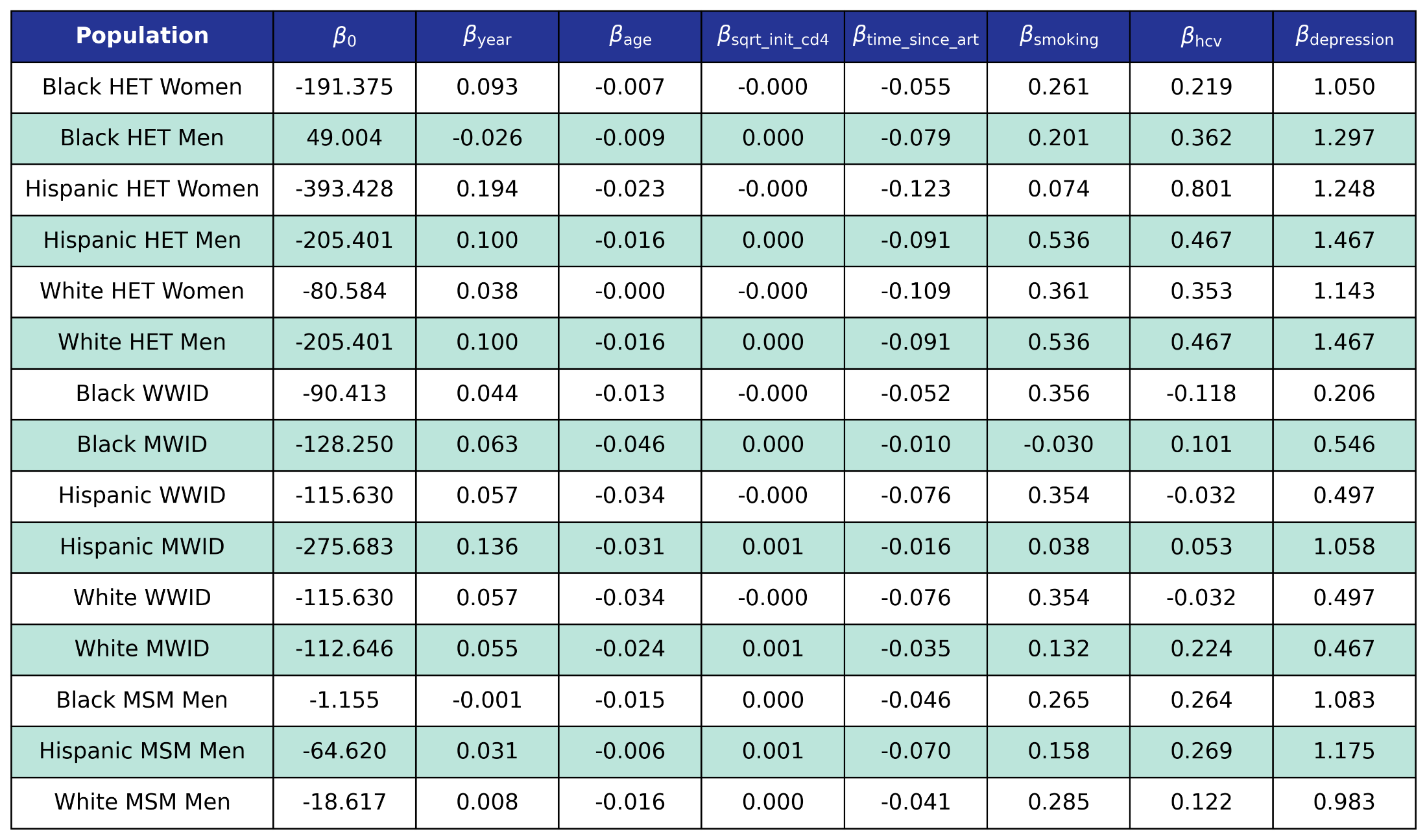


We use logistic regression to model the probability of incidence of anxiety as a function of calendar year (year), age (age), square root of CD4 count at ART initiation (sqrt_init_cd4), number of years since ART initiation (time_since_art), smoking status (smoking), HCV status (hcv) and depression status (depression). The status variables are encoded as Boolean variables.

S2c) Depression prevalence estimates (from the NA-ACCORD)
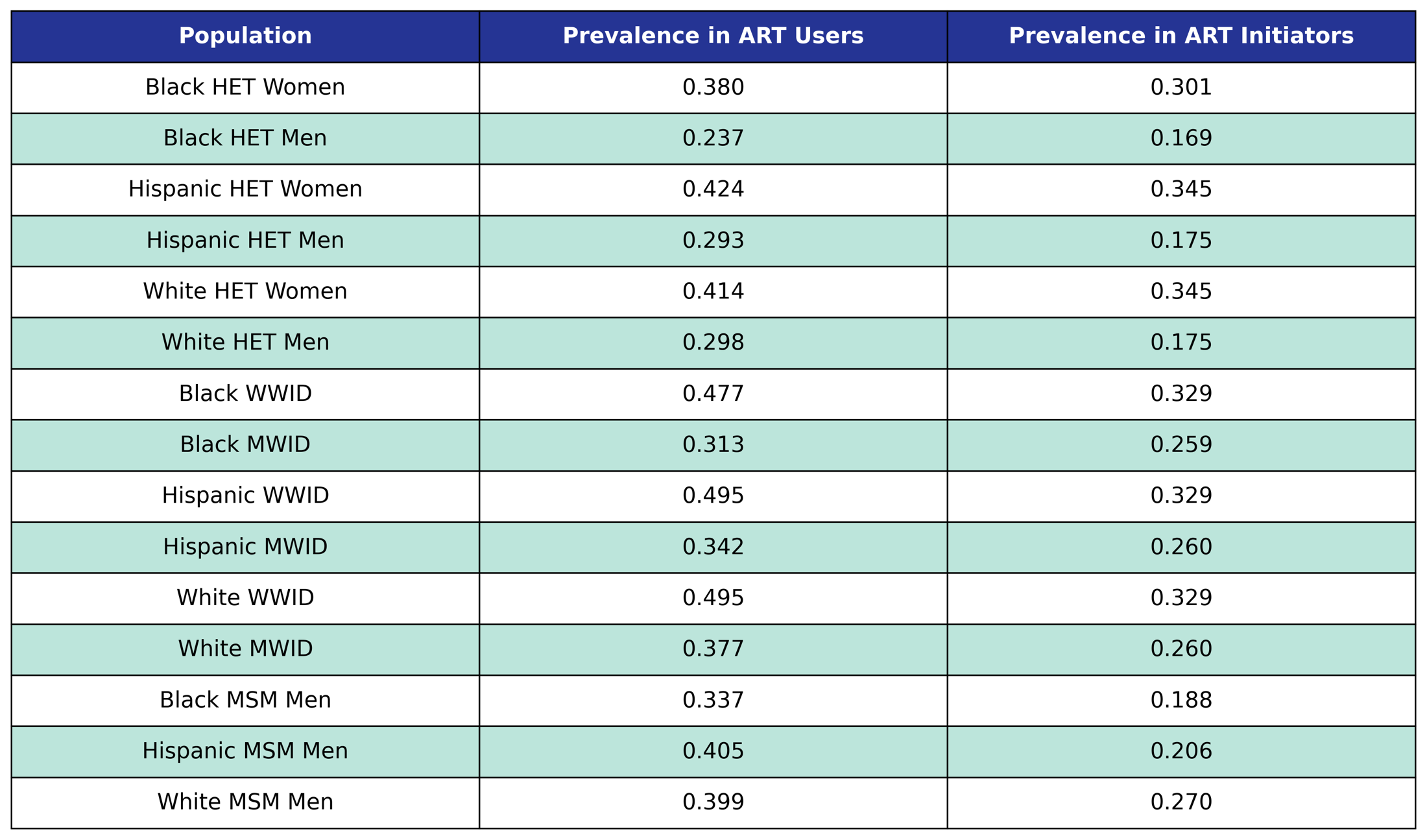


Prevalence in the 2009 ART user population is taken from the 2009 NA-ACCORD population, while prevalence in ART initiators was taken from the 2009 - 2017 NA-ACCORD ART initiator population.

S2d) Coefficient estimates from depression incidence functions (from the NA-ACCORD)
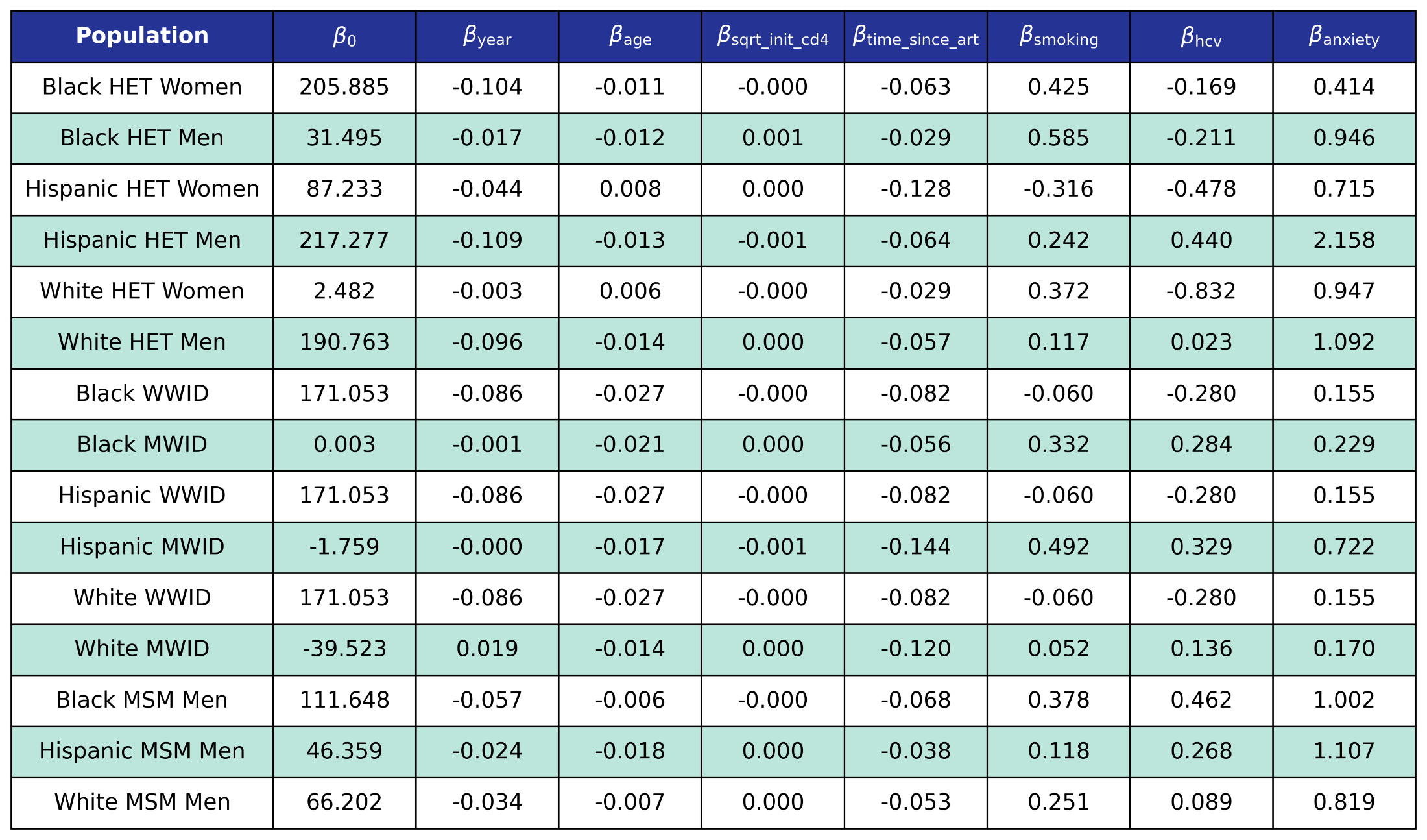


We use logistic regression to model the probability of incidence of depression as a function of calendar year (year), age (age), square root of CD4 count at ART initiation (sqrt_init_cd4), number of years since ART initiation (time_since_art), smoking status (smoking), HCV status (hcv) and anxiety status (anxiety). The status variables are encoded as Boolean variables.

S2e) Stage ≥3 chronic kidney disease prevalence estimates (from the NA-ACCORD)
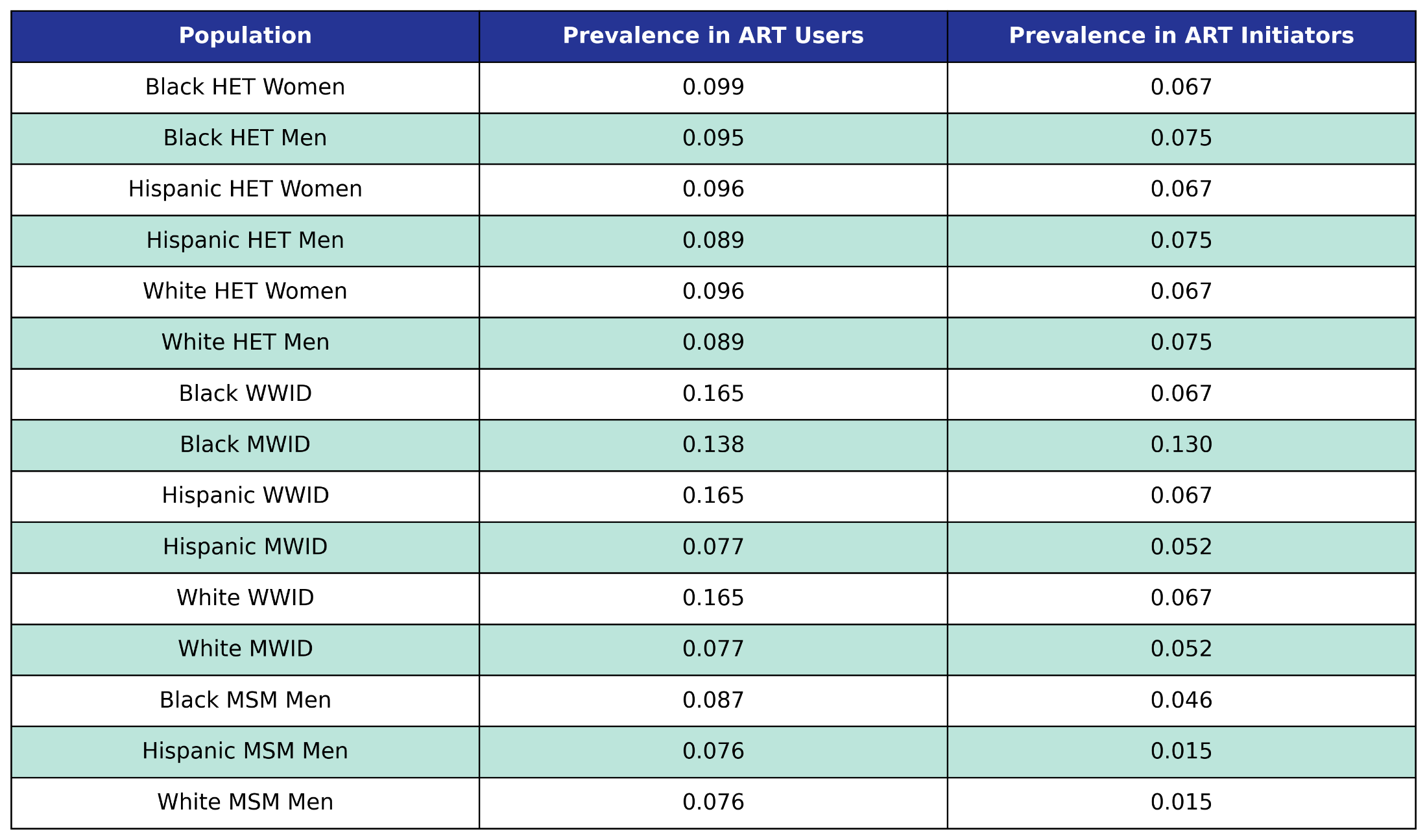


Prevalence in the 2009 ART user population is taken from the 2009 NA-ACCORD population, while prevalence in ART initiators was taken from the 2009 - 2017 NA-ACCORD ART initiator population.

S2f) Coefficient estimates from stage ≥3 chronic kidney disease incidence functions (from the NA-ACCORD)
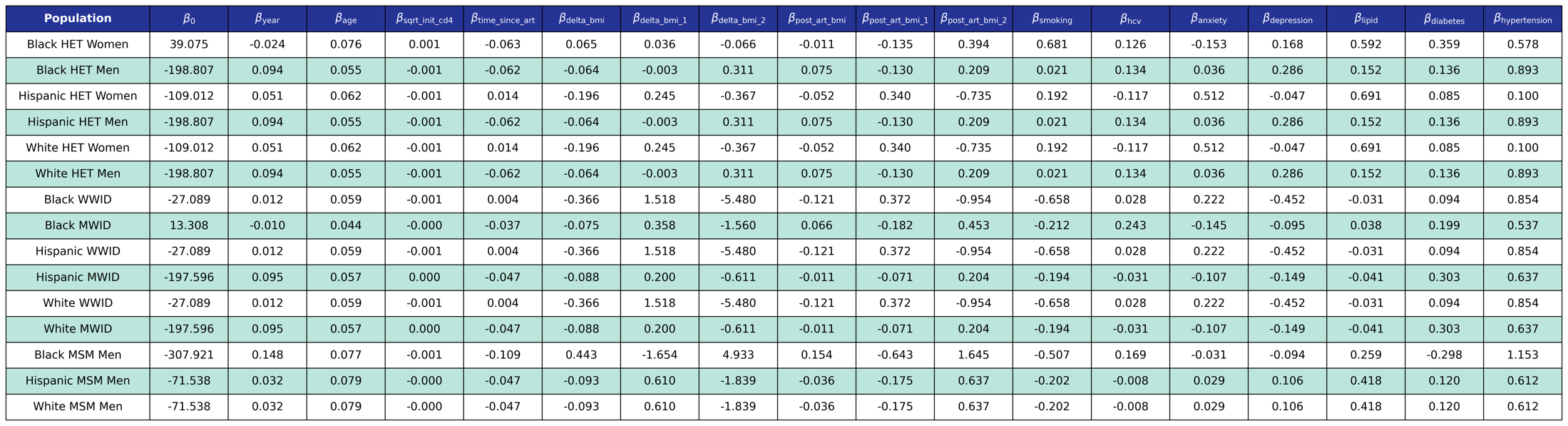


We use logistic regression to model the probability of incidence of this comorbidity as a linear function of calendar year (year), age (age), square root of CD4 count at ART initiation (sqrt_init_cd4), number of years since ART initiation (time_since_art), change in BMI after ART initiation (delta_bmi) and BMI after ART initiation (post_art_bmi) modeled as [restricted cubic splines](https://pearlhivmodel.org/method_details.html#restricted-cubic-spline) (see <https://pearlhivmodel.org/method_details.htm>l for knots), smoking status (smoking), hepatitis C virus (hcv), anxiety (anxiety), depression (depression), dyslipidemia (lipid), diabetes (diabetes), and hypertension (hypertension).

S2g) Dyslipidemia prevalence estimates (from the NA-ACCORD)
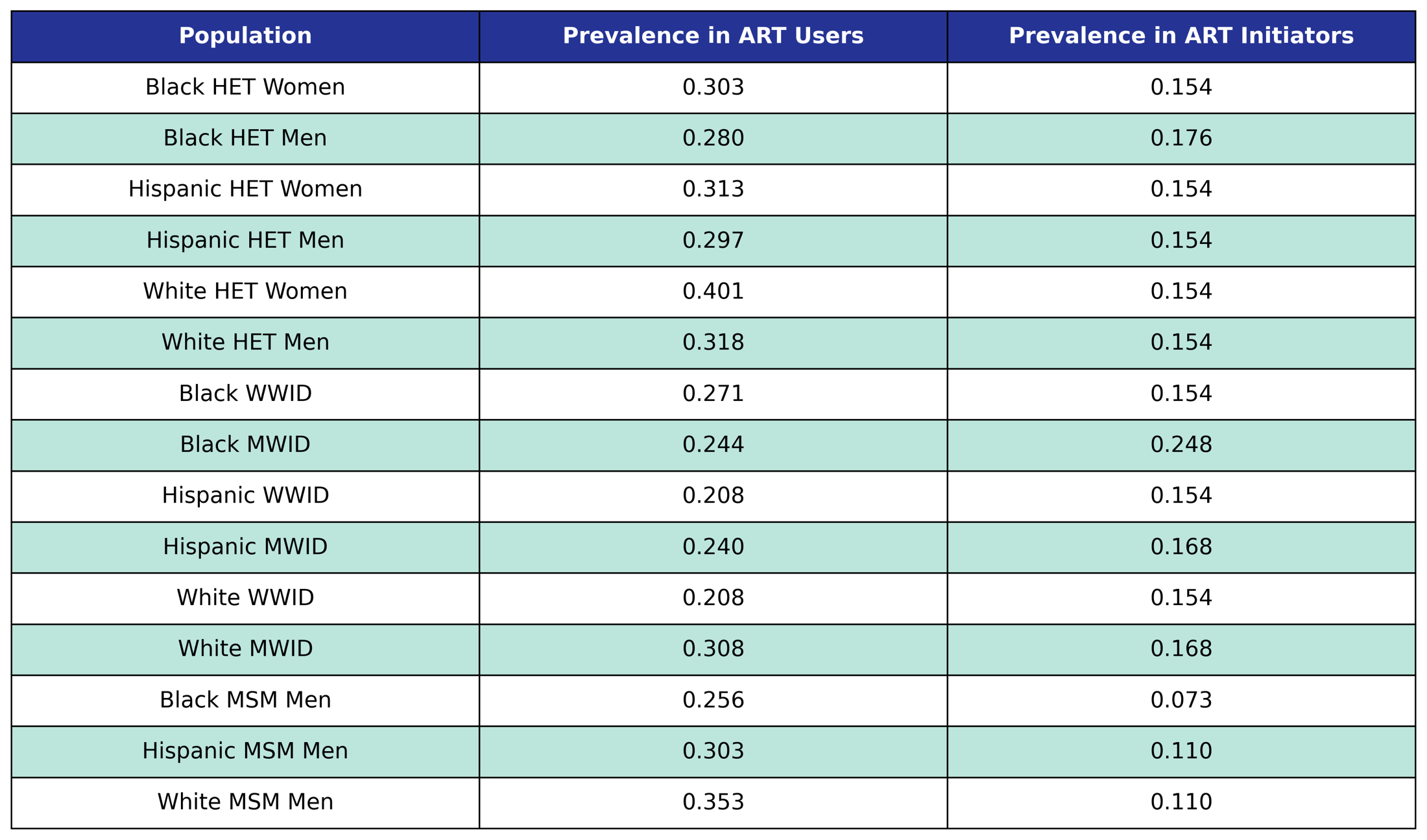


Prevalence in the 2009 ART user population is taken from the 2009 NA-ACCORD population, while prevalence in ART initiators was taken from the 2009 - 2017 NA-ACCORD ART initiator population.

S2h) Coefficient estimates from dyslipidemia incidence functions (from the NA-ACCORD)
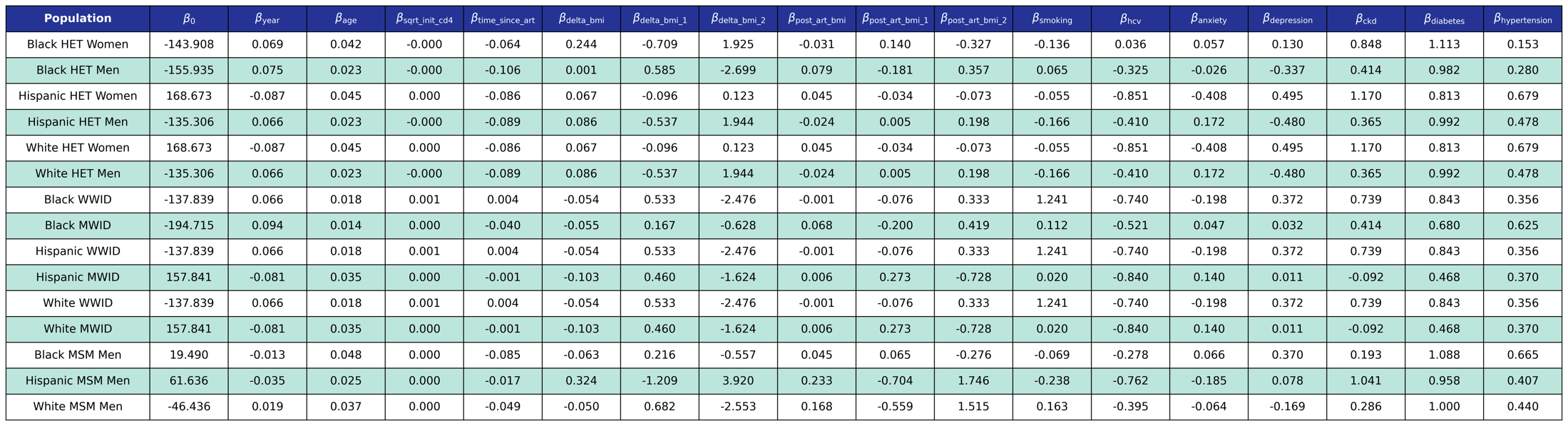


We use logistic regression to model the probability of incidence of this comorbidity as a linear function of calendar year (year), age (age), square root of CD4 count at ART initiation (sqrt_init_cd4), number of years since ART initiation (time_since_art), change in BMI after ART initiation (delta_bmi) and BMI after ART initiation (post_art_bmi) modeled as [restricted cubic splines](https://pearlhivmodel.org/method_details.html#restricted-cubic-spline) (see [https://pearlhivmodel.org/method_details.html](https://pearlhivmodel.org/method_details.html#depression) for knots), smoking status (smoking), hepatitis C virus (hcv), anxiety (anxiety), depression (depression), stage ≥3 chronic kidney disease (ckd), diabetes (diabetes), and hypertension (hypertension).

S2i) Diabetes prevalence estimates (from the NA-ACCORD)


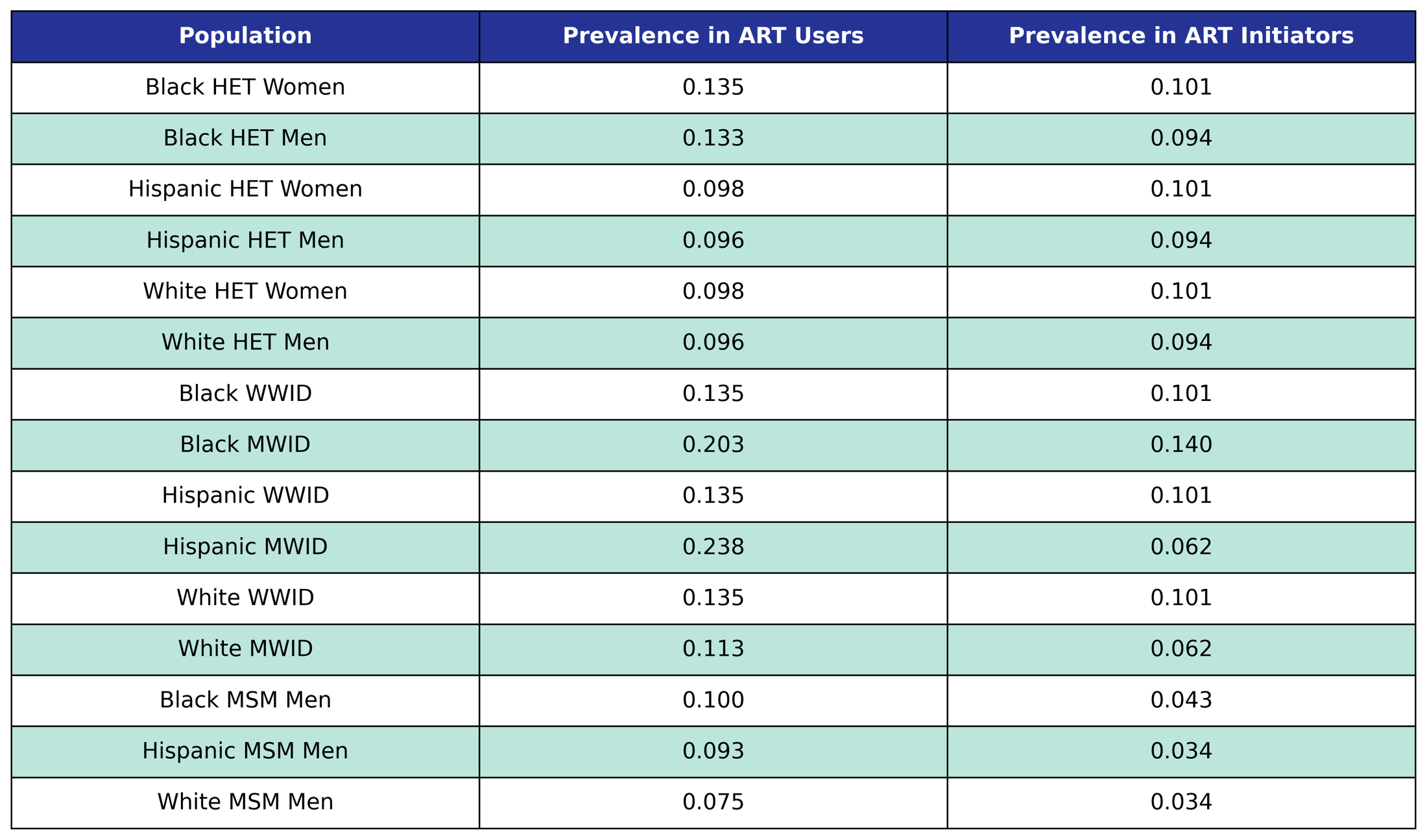


Prevalence in the 2009 ART user population is taken from the 2009 NA-ACCORD population, while prevalence in ART initiators was taken from the 2009 - 2017 NA-ACCORD ART initiator population.

S2j) Coefficient estimates from diabetes incidence functions (from the NA-ACCORD)


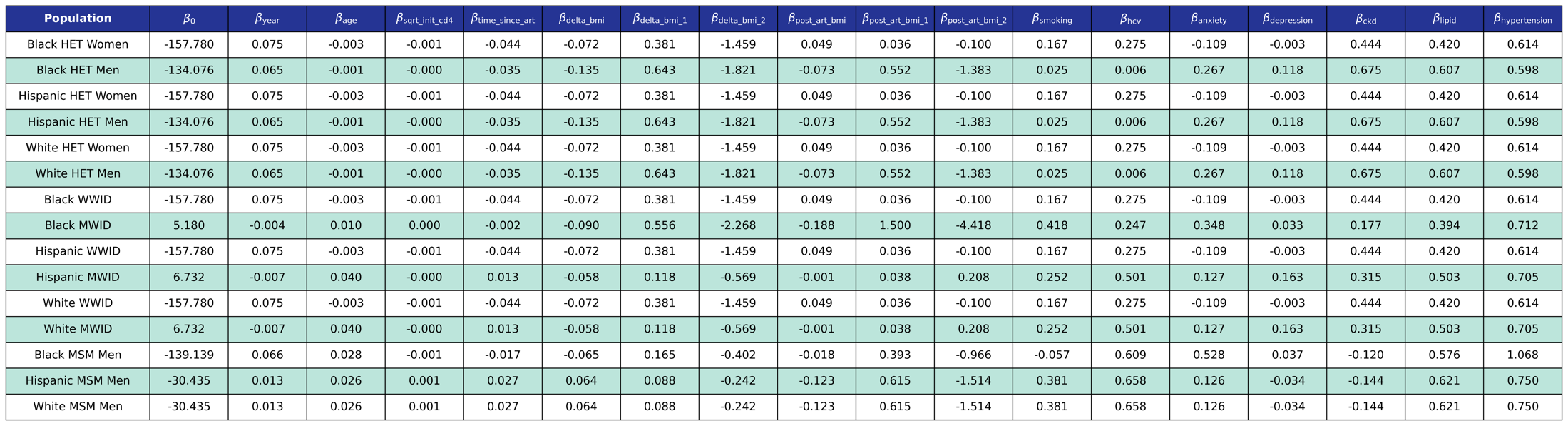


We use logistic regression to model the probability of incidence of this comorbidity as a linear function of calendar year (year), age (age), square root of CD4 count at ART initiation (sqrt_init_cd4), number of years since ART initiation (time_since_art), change in BMI after ART initiation (delta_bmi) and BMI after ART initiation (post_art_bmi) modeled as [restricted cubic splines](https://pearlhivmodel.org/method_details.html#restricted-cubic-spline) (see [https://pearlhivmodel.org/method_details.html](https://pearlhivmodel.org/method_details.html#depression) for knots), smoking status (smoking), hepatitis C virus (hcv), anxiety (anxiety), depression (depression), stage ≥3 chronic kidney disease (ckd), dyslipidemia (lipids), and hypertension (hypertension).

S2k) Hypertension prevalence estimates (from the NA-ACCORD)


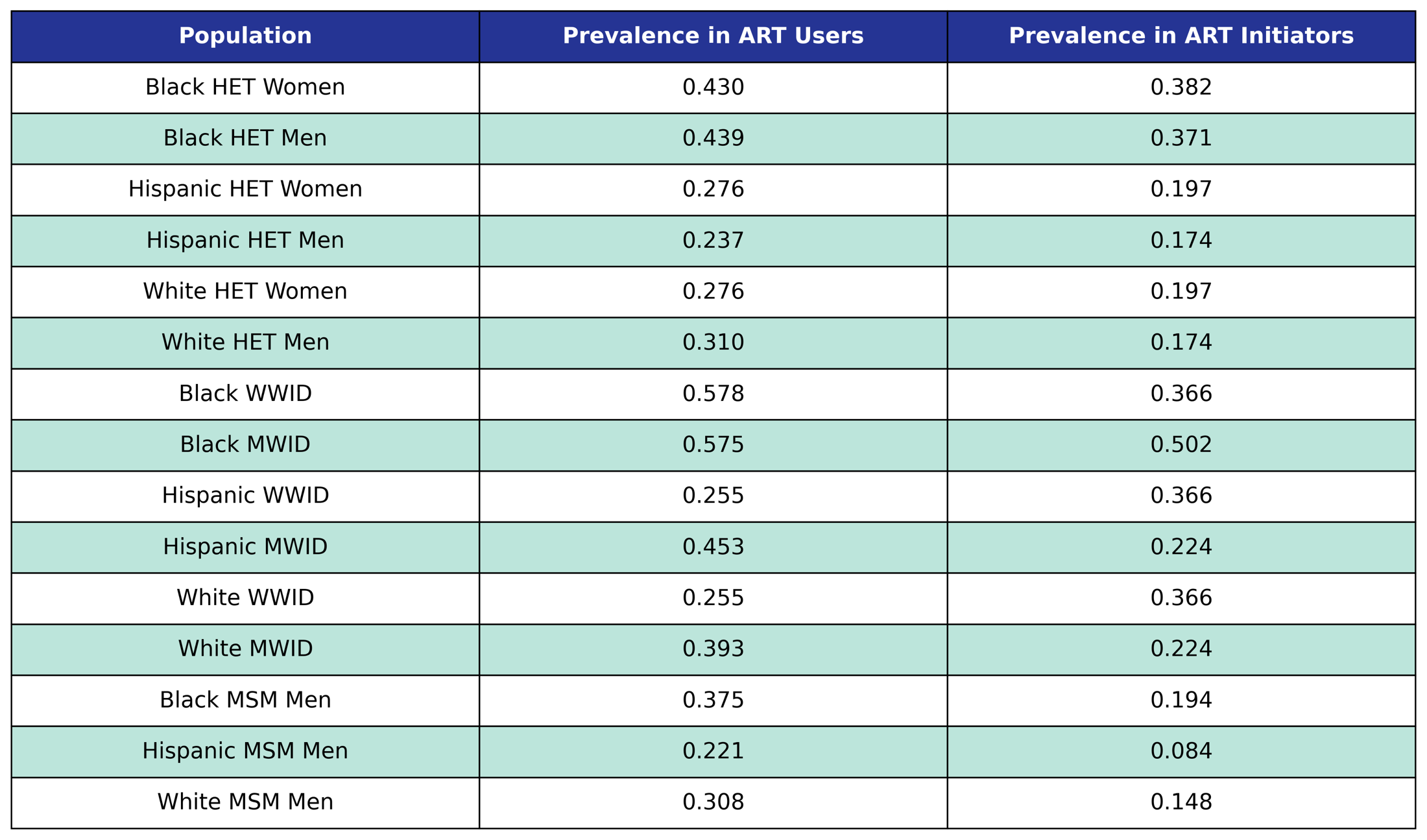


Prevalence in the 2009 ART user population is taken from the 2009 NA-ACCORD population, while prevalence in ART initiators was taken from the 2009 - 2017 NA-ACCORD ART initiator population.

S2l) Coefficient estimates from hypertension incidence functions (from the NA-ACCORD)


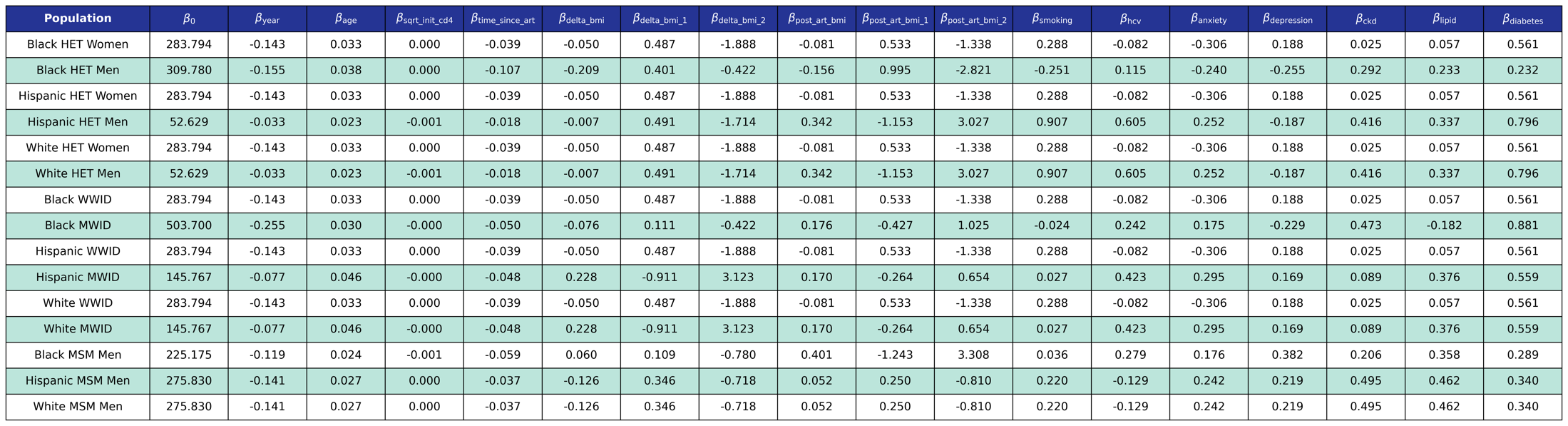


We use logistic regression to model the probability of incidence of this comorbidity as a linear function of calendar year (year), age (age), square root of CD4 count at ART initiation (sqrt_init_cd4), number of years since ART initiation (time_since_art), change in BMI after ART initiation (delta_bmi) and BMI after ART initiation (post_art_bmi) modeled as [restricted cubic splines](https://pearlhivmodel.org/method_details.html#restricted-cubic-spline) (see [https://pearlhivmodel.org/method_details.html](https://pearlhivmodel.org/method_details.html#depression) for knots), smoking status (smoking), hepatitis C virus (hcv), anxiety (anxiety), depression (depression), stage ≥3 chronic kidney disease (ckd), dyslipidemia (lipids), and diabetes (diabetes).

S2m) Cancer prevalence estimates (from the NA-ACCORD)


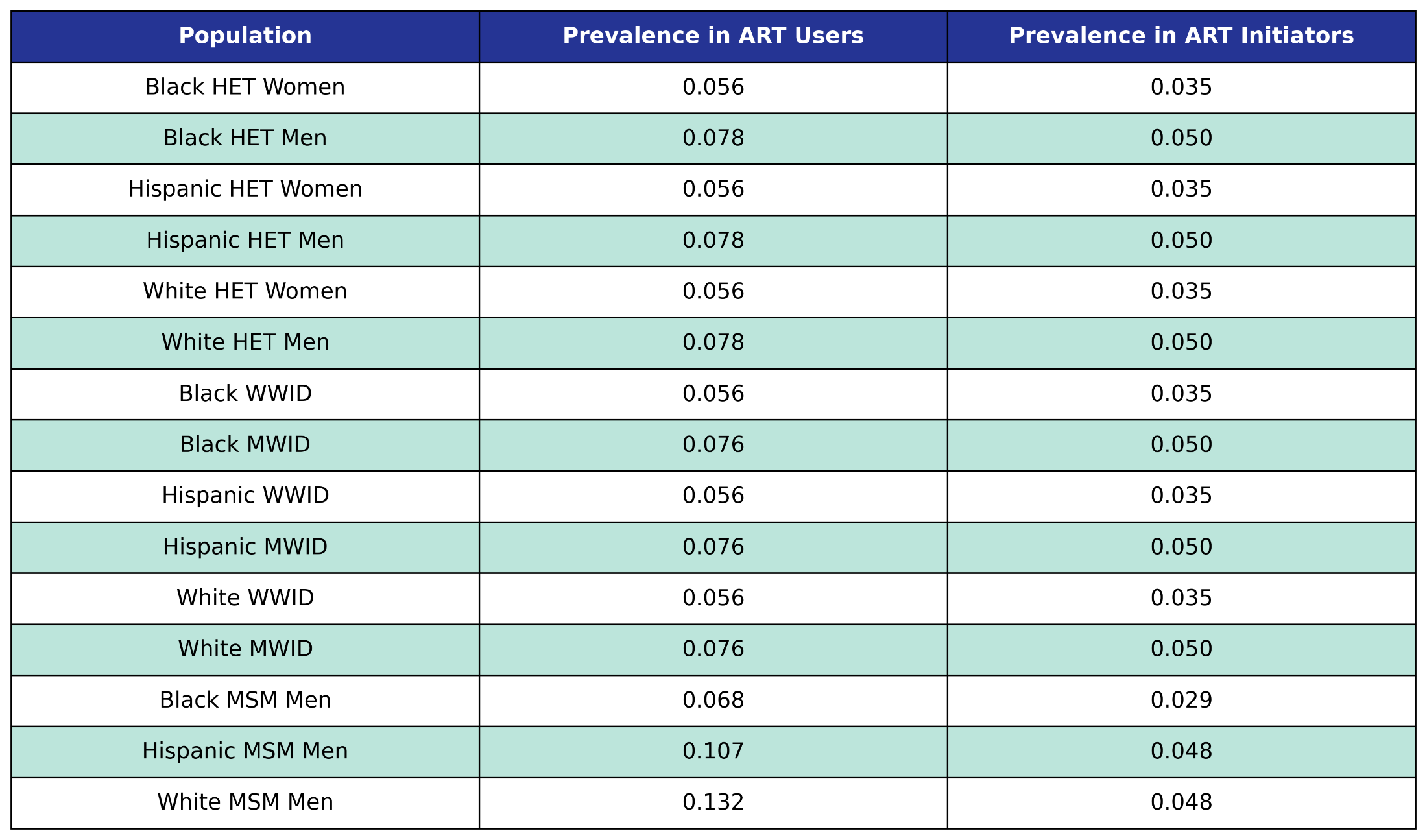


Prevalence in the 2009 ART user population is taken from the 2009 NA-ACCORD population, while prevalence in ART initiators was taken from the 2009 - 2017 NA-ACCORD ART initiator population.

S2n) Coefficient estimates from cancer incidence functions (from the NA-ACCORD)


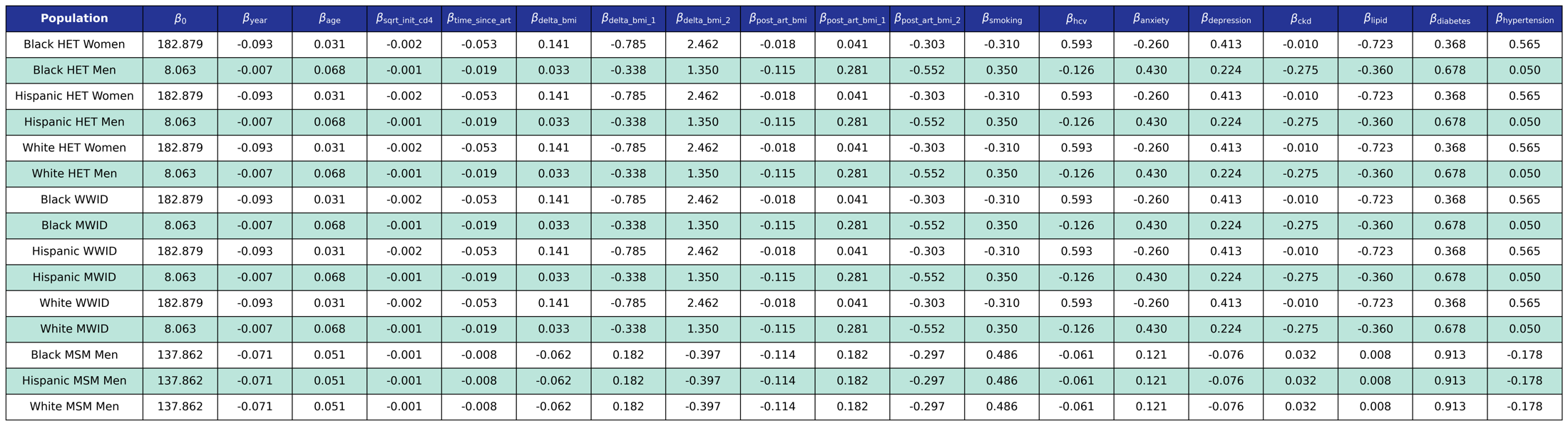


We use logistic regression to model the probability of incidence of this comorbidity as a linear function of calendar year (year), age (age), square root of CD4 count at ART initiation (sqrt_init_cd4), number of years since ART initiation (time_since_art), change in BMI after ART initiation (delta_bmi) and BMI after ART initiation (post_art_bmi) modeled as restricted cubic splines (see [https://pearlhivmodel.org/method_details.html](https://pearlhivmodel.org/method_details.html#depression) for knots), smoking status (smoking), hepatitis C virus (hcv), anxiety (anxiety), depression (depression), stage ≥3 chronic kidney disease (ckd), dyslipidemia (lipid), diabetes (diabetes), and hypertension (hypertension).

S2o) End-stage liver disease prevalence estimates (from the NA-ACCORD)


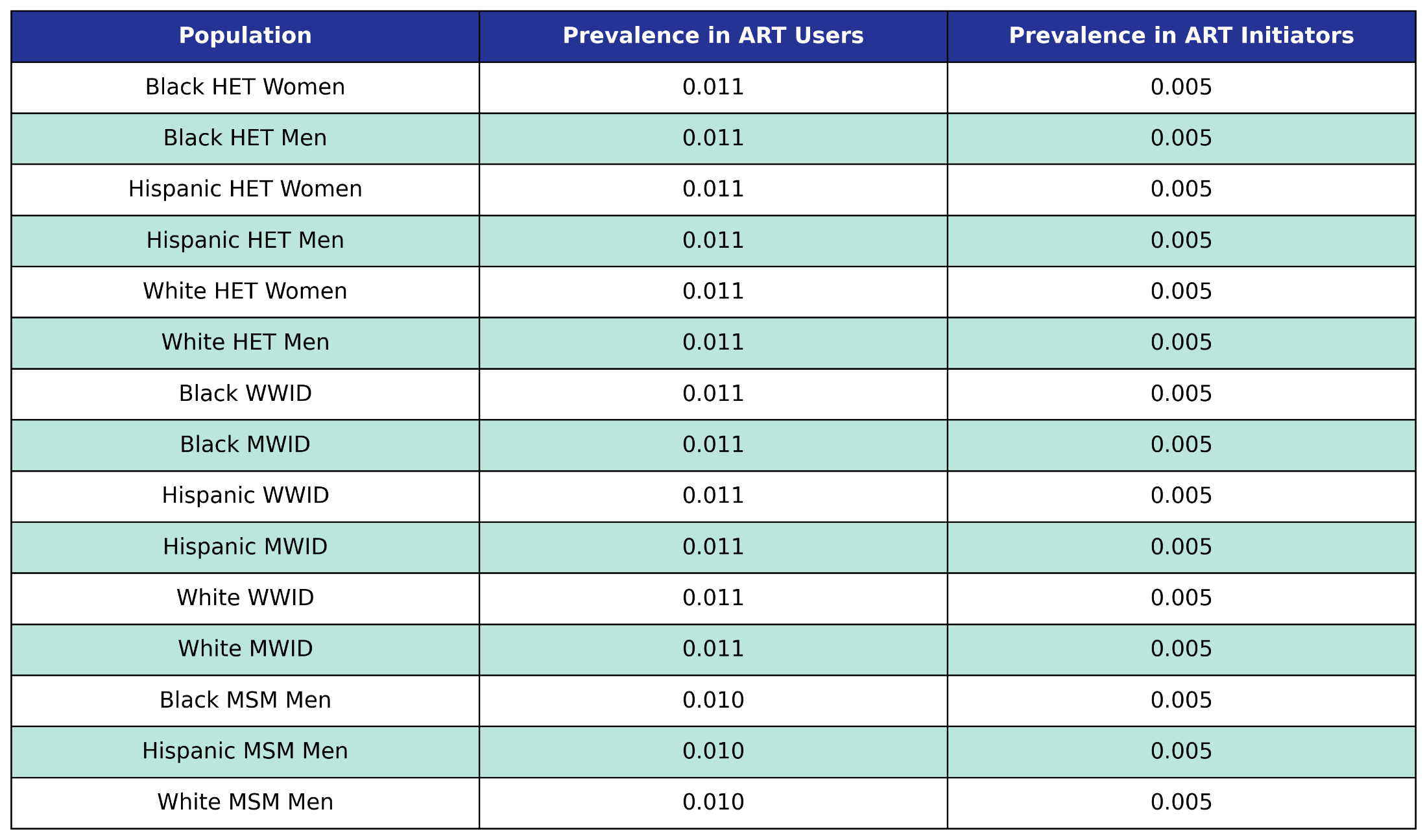


Prevalence in the 2009 ART user population is taken from the 2009 NA-ACCORD population, while prevalence in ART initiators was taken from the 2009 - 2017 NA-ACCORD ART initiator population.

S2p) Coefficient estimates from end-stage liver disease incidence functions (from the NA-ACCORD)


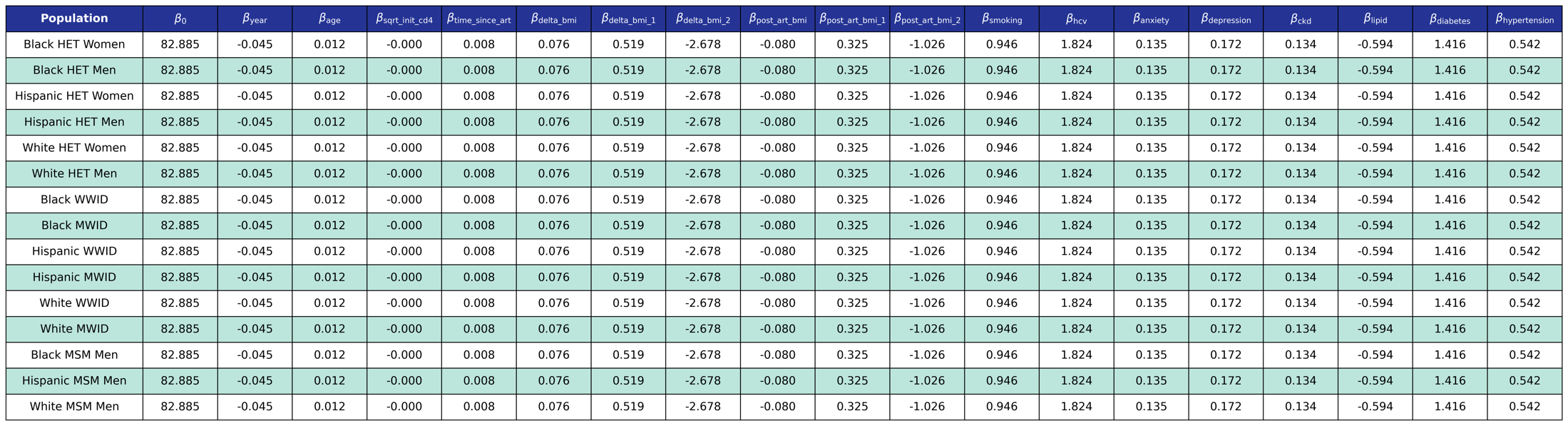


We use logistic regression to model the probability of incidence of this comorbidity as a linear function of calendar year (year), age (age), square root of CD4 count at ART initiation (sqrt_init_cd4), number of years since ART initiation (time_since_art), change in BMI after ART initiation (delta_bmi) and BMI after ART initiation (post_art_bmi) modeled as restricted cubic splines (see [https://pearlhivmodel.org/method_details.html](https://pearlhivmodel.org/method_details.html#depression) for knots), smoking status (smoking), hepatitis C virus (hcv), anxiety (anxiety), depression (depression), stage ≥3 chronic kidney disease (ckd), dyslipidemia (lipid), diabetes (diabetes), and hypertension (hypertension).

S2q) Myocardial infarction prevalence estimates (from the NA-ACCORD)


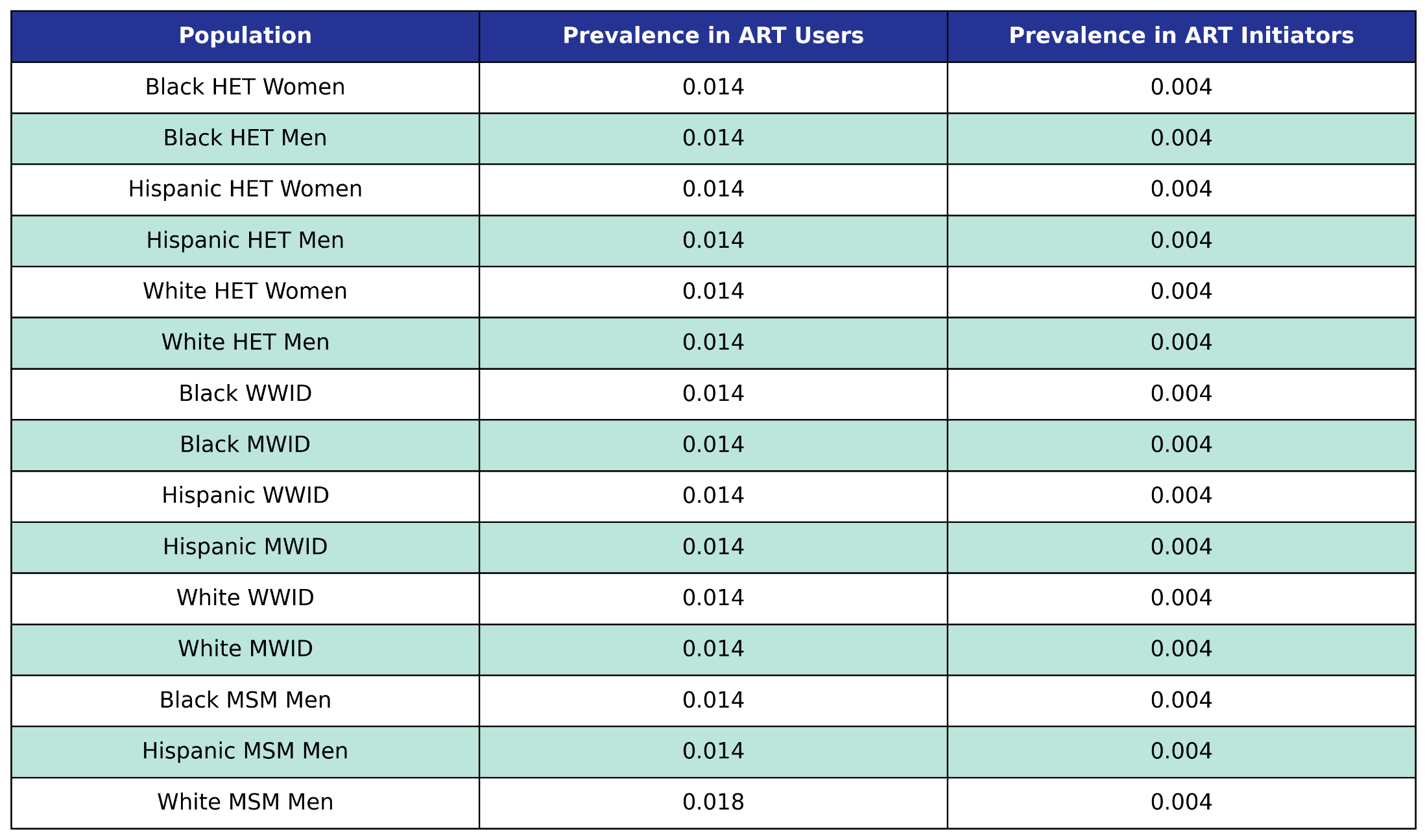
Prevalence in the 2009 ART user population is taken from the 2009 NA-ACCORD population, while prevalence in ART initiators was taken from the 2009 - 2017 NA-ACCORD ART initiator population.

S2r) Coefficient estimates from myocardial infarction incidence functions (from the NA-ACCORD)

**
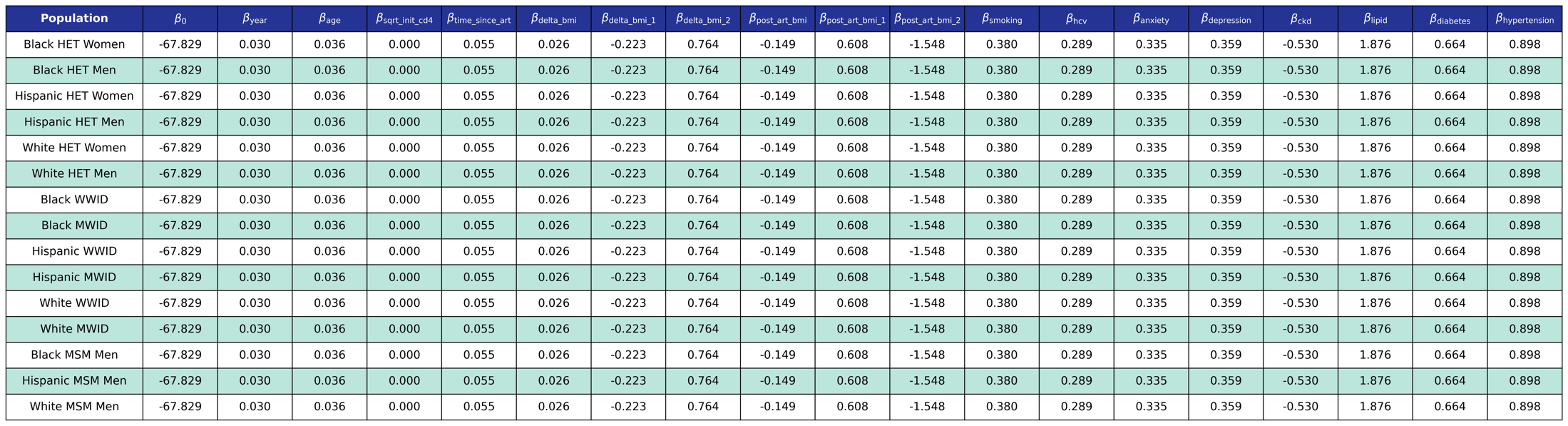
**

We use logistic regression to model the probability of incidence of this comorbidity as a linear function of calendar year (year), age (age), square root of CD4 count at ART initiation (sqrt_init_cd4), number of years since ART initiation (time_since_art), change in BMI after ART initiation (delta_bmi) and BMI after ART initiation (post_art_bmi) modeled as restricted cubic splines (see [https://pearlhivmodel.org/method_details.html](https://pearlhivmodel.org/method_details.html#depression) for knots), smoking status (smoking), hepatitis C virus (hcv), anxiety (anxiety), depression (depression), stage ≥3 chronic kidney disease (ckd), dyslipidemia (lipid), diabetes (diabetes), and hypertension (hypertension).
