# Supplementary material for: The forecasted prevalence of comorbidities and multimorbidity in people with HIV in the United States through the year 2030: A modeling study
Source: PLoS Med. 2024 Jan 12;21(1):e1004325. doi: 10.1371/journal.pmed.1004325 (PMC10833859; doi:10.1371/journal.pmed.1004325)
Supplement: S3 Table — (DOCX) [file pmed.1004325.s010.docx]

**S3 Table:** PEARL a) in-care and b) dis-engaged from care mortality functions that include comorbidity presence

S3a) Coefficient estimates from mortality functions for those in-care


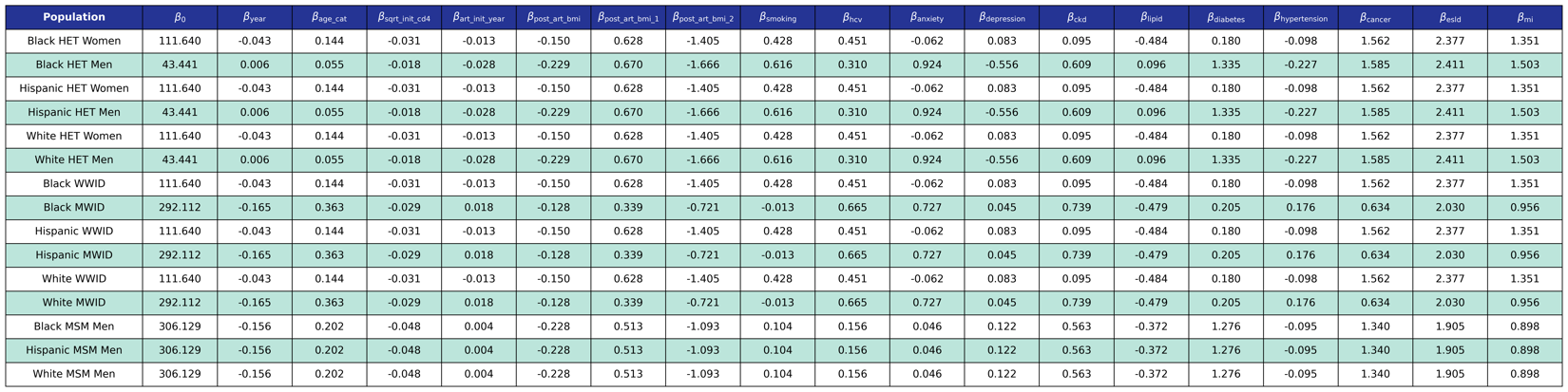


We use logistic regression to model the probability of dying in care as a function of calendar year (year), 10 year age category (age_cat), CD4 count at ART initiation (sqrt_init_cd4), year of ART initiation (art_init_year), BMI after ART initiation (post_art_bmi) modeled as a restricted cubic spline (see [https://pearlhivmodel.org/method_details.html](https://pearlhivmodel.org/method_details.html#depression) for knots), smoking status (smoking), hepatitis C virus (hcv), anxiety (anxiety), depression (depression), stage ≥3 chronic kidney disease (ckd), dyslipidemia (lipids), diabetes (diabetes), hypertension (hypertension), cancer (cancer), end-stage liver disease (esld), and myocardial infarction (mi). The coefficients were estimated using a Generalized Estimating Equation (GEE) with a logit link and an unstructured correlation structure using the geepack software package for R. The NA-ACCORD dataset was restricted to the years 2009-2015 and each patient is represented by a data point for each year they were alive and under observation in NA-ACCORD.

S3b) Coefficient estimates from mortality functions for those dis-engaged from care and ART


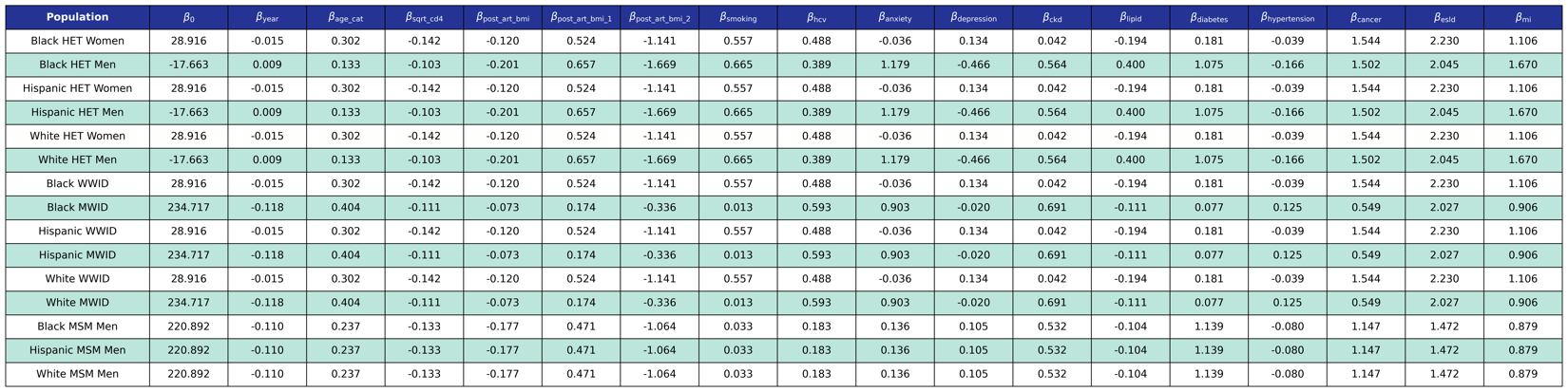


We use logistic regression to model the probability of dying out of care as a function of calendar year (year), 10 year age category (age_cat), CD4 count (sqrt_cd4), BMI after ART initiation (post_art_bmi) modeled as a restricted cubic spline (see [https://pearlhivmodel.org/method_details.html](https://pearlhivmodel.org/method_details.html#depression) for knots), smoking status (smoking), hepatitis C virus (hcv), anxiety (anxiety), depression (depression), stage ≥3 chronic kidney disease (ckd), dyslipidemia (lipids), diabetes (diabetes), hypertension (hypertension), cancer (cancer), end-stage liver disease (esld), and myocardial infarction (mi). The coefficients were estimated using a Generalized Estimating Equation (GEE) with a logit link and an unstructured correlation structure using the geepack software package for R. The NA-ACCORD dataset was restricted to the years 2009-2015.
