# Supplementary material for: The forecasted prevalence of comorbidities and multimorbidity in people with HIV in the United States through the year 2030: A modeling study
Source: PLoS Med. 2024 Jan 12;21(1):e1004325. doi: 10.1371/journal.pmed.1004325 (PMC10833859; doi:10.1371/journal.pmed.1004325)
Supplement: S4 Table — (DOCX) [file pmed.1004325.s011.docx]

**S4 Table:** Characteristics of the PEARL-simulated agents using ART, 2020 and 2030

|  | **2020 (Forecasted)** | | **2030 (Forecasted)** | |  |  |
| --- | --- | --- | --- | --- | --- | --- |
|  | **PEARL^a^** | | **PEARL^a^** | | |  |
|  | **N =** | **670,036 [663,496, 676,801]** | **N =** | **908,504 [875,310, 946,810]** | | |
| **Characteristics** | **n [95% uncertainty range]** | **% [95% uncertainty range]** | **n [95% uncertainty range]** | **% [95% uncertainty range]** | | |
| **Age (in years)** |  |  |  |  | | |
| <20 | 708 [616, 783] | 0.1% [0.1%, 0.1%] | 1,218 [771, 2,187] | 0.1% [0.1%, 0.2%] | | |
| 20-24 | 11,520 [11,023, 11,951] | 1.7% [1.7%, 1.8%] | 12,416 [10,209, 14,913] | 1.4% [1.2%, 1.6%] | | |
| 25-29 | 40,475 [39,501, 41,572] | 6.0% [5.9%, 6.2%] | 40,927 [35,666, 46,302] | 4.5% [4.1%, 5.0%] | | |
| 30-34 | 66,704 [65,542, 67,887] | 10.0% [9.9%, 10.1%] | 78,305 [71,178, 87,266] | 8.6% [8.0%, 9.3%] | | |
| 35-39 | 62,720 [61,827, 63,651] | 9.4% [9.3%, 9.4%] | 101,328 [94,610, 108,518] | 11.1% [10.7%, 11.6%] | | |
| 40-44 | 60,845 [59,976, 61,576] | 9.1% [9.0%, 9.2%] | 102,122 [97,769, 107,381] | 11.2% [11.0%, 11.5%] | | |
| 45-49 | 79,388 [78,595, 80,298] | 11.8% [11.8%, 11.9%] | 85,320 [82,415, 89,088] | 9.4% [9.2%, 9.6%] | | |
| 50-54 | 97,822 [96,957, 98,874] | 14.6% [14.5%, 14.7%] | 81,110 [77,868, 84,222] | 8.9% [8.7%, 9.2%] | | |
| 55-59 | 100,416 [99,474, 101,309] | 15.0% [14.9%, 15.1%] | 94,472 [91,629, 97,330] | 10.4% [10.1%, 10.6%] | | |
| 60-64 | 76,357 [75,617, 76,909] | 11.4% [11.3%, 11.5%] | 102,878 [100,976, 105,213] | 11.3% [11.0%, 11.6%] | | |
| 65-69 | 44,442 [43,956, 44,848] | 6.6% [6.6%, 6.7%] | 94,677 [93,322, 96,294] | 10.4% [10.1%, 10.7%] | | |
| 70-74 | 19,669 [19,334, 19,941] | 2.9% [2.9%, 3.0%] | 64,950 [64,052, 65,811] | 7.1% [6.9%, 7.4%] | | |
| ≥75 | 8,912 [8,751, 9,093] | 1.3% [1.3%, 1.4%] | 48,378 [47,662, 49,104] | 5.3% [5.1%, 5.5%] | | |
| **Male sex** | 515,560 [509,423, 521,993] | 76.9% [76.6%, 77.3%] | 711,684 [680,284, 746,183] | 78.3% [77.0%, 79.5%] | | |
| **Race** |  |  |  |  | | |
| White | 216,936 [213,752, 220,630] | 32.4% [31.9%, 32.8%] | 259,610 [244,388, 276,853] | 28.6% [27.0%, 30.1%] | | |
| Black/AA | 296,512 [291,437, 300,242] | 44.2% [43.8%, 44.7%] | 395,026 [371,066, 412,301] | 43.3% [41.4%, 45.1%] | | |
| Hispanic | 156,835 [153,953, 160,121] | 23.4% [23.0%, 23.9%] | 255,037 [237,922, 274,689] | 28.2% [26.4%, 30.0%] | | |
| **Sub-groups** |  |  |  |  | | |
| MSM | 404,087 [398,380, 409,818] | 60.3% [59.8%, 60.8%] | 584,158 [554,253, 616,656] | 64.4% [62.6%, 65.9%] | | |
| White MSM | 156,068 [153,288, 159,222] | 23.3% [22.9%, 23.7%] | 178,730 [165,142, 194,269] | 19.7% [18.3%, 21.1%] | | |
| Black/AA MSM | 143,910 [141,106, 146,306] | 21.5% [21.1%, 21.8%] | 218,548 [204,454, 229,801] | 24.0% [22.7%, 25.2%] | | |
| Hispanic MSM | 104,138 [101,474, 106,891] | 15.5% [15.1%, 15.9%] | 188,012 [171,134, 205,338] | 20.8% [18.9%, 22.4%] | | |
| Men who injected drugs (MWID)**^b^** | 59,597 [57,934, 61,321] | 8.9% [8.6%, 9.2%] | 66,022 [58,818, 73,523] | 7.2% [6.5%, 8.1%] | | |
| White MWID | 23,462 [22,660, 24,421] | 3.5% [3.4%, 3.6%] | 28,978 [25,751, 32,671] | 3.2% [2.8%, 3.6%] | | |
| Black/AA MWID | 21,177 [20,328, 21,905] | 3.2% [3.0%, 3.3%] | 18,184 [14,487, 21,573] | 2.0% [1.6%, 2.4%] | | |
| Hispanic MWID | 14,920 [14,137, 15,712] | 2.2% [2.1%, 2.3%] | 18,526 [15,071, 21,982] | 2.1% [1.7%, 2.4%] | | |
| Women who injected drugs (WWID) | 28,193 [27,586, 28,832] | 4.2% [4.1%, 4.3%] | 31,962 [29,090, 34,468] | 3.5% [3.2%, 3.8%] | | |
| White WWID | 9,506 [9,301, 9,735] | 1.4% [1.4%, 1.5%] | 13,274 [12,473, 14,135] | 1.5% [1.4%, 1.6%] | | |
| Black/AA WWID | 14,682 [14,199, 15,121] | 2.2% [2.1%, 2.3%] | 13,661 [11,641, 15,563] | 1.5% [1.3%, 1.7%] | | |
| Hispanic WWID | 4,050 [3,687, 4,225] | 0.6% [0.6%, 0.6%] | 5,088 [3,656, 5,892] | 0.6% [0.4%, 0.7%] | | |
| Heterosexual men | 51,900 [50,399, 53,287] | 7.8% [7.5%, 7.9%] | 60,984 [53,861, 67,409] | 6.7% [5.9%, 7.4%] | | |
| White heterosexual men | 6,839 [6,476, 7,128] | 1.0% [1.0%, 1.1%] | 9,390 [7,891, 10,693] | 1.0% [0.9%, 1.2%] | | |
| Black/AA heterosexual men | 34,154 [32,851, 35,297] | 5.1% [4.9%, 5.3%] | 39,292 [32,786, 44,413] | 4.3% [3.6%, 4.9%] | | |
| Hispanic heterosexual men | 11,009 [10,494, 11,466] | 1.6% [1.6%, 1.7%] | 12,806 [10,384, 14,890] | 1.4% [1.2%, 1.7%] | | |
| Heterosexual women | 126,139 [123,621, 128,843] | 18.8% [18.5%, 19.2%] | 165,104 [153,149, 177,622] | 18.2% [17.0%, 19.5%] | | |
| White heterosexual women | 20,968 [20,192, 21,642] | 3.1% [3.0%, 3.2%] | 28,974 [25,378, 32,029] | 3.2% [2.8%, 3.5%] | | |
| Black/AA heterosexual women | 82,536 [80,792, 84,403] | 12.3% [12.1%, 12.6%] | 105,384 [97,445, 113,664] | 11.6% [10.7%, 12.5%] | | |
| Hispanic heterosexual women | 22,806 [21,810, 23,652] | 3.4% [3.3%, 3.5%] | 31,550 [27,116, 35,339] | 3.4% [3.0%, 3.9%] | | |
| **Mental Comorbidities** |  |  |  |  | | |
| Anxiety | 243,968 [241,873, 246,455] | 36.4% [36.3%, 36.6%] | 425,498 [413,523, 439,991] | 46.8% [46.3%, 47.5%] | | |
| Depression | 314,996 [312,366, 317,810] | 47.0% [46.9%, 47.2%] | 442,003 [430,455, 455,949] | 48.6% [48.2%, 49.1%] | | |
| ≥1 mental comorbidity | 402,042 [398,647, 405,826] | 60.0% [59.9%, 60.2%] | 584,875 [569,056, 605,134] | 64.4% [63.8%, 65.0%] | | |
| **Physical Comorbidities** |  |  |  |  | | |
| Chronic Kidney Disease | 126,360 [125,234, 127,304] | 18.8% [18.7%, 19.0%] | 273,896 [267,655, 279,051] | 30.1% [29.4%, 30.8%] | | |
| Dyslipidemia | 283,674 [281,405, 285,802] | 42.3% [42.2%, 42.5%] | 435,806 [425,275, 446,285] | 48.0% [47.1%, 48.9%] | | |
| Diabetes | 119,172 [118,039, 120,225] | 17.8% [17.6%, 17.9%] | 246,172 [240,365, 251,751] | 27.1% [26.4%, 27.7%] | | |
| Hypertension | 246,736 [244,645, 248,756] | 36.8% [36.6%, 37.0%] | 295,184 [286,765, 302,713] | 32.5% [31.8%, 33.1%] | | |
| Cancer | 74,712 [73,993, 75,376] | 11.2% [11.1%, 11.2%] | 101,700 [99,786, 104,328] | 11.2% [11.0%, 11.4%] | | |
| End-Stage Liver Disease | 8,950 [8,776, 9,183] | 1.3% [1.3%, 1.4%] | 12,974 [12,697, 13,337] | 1.4% [1.4%, 1.5%] | | |
| Myocardial Infarction | 22,064 [21,738, 22,382] | 3.3% [3.2%, 3.3%] | 73,666 [72,853, 74,512] | 8.1% [7.9%, 8.3%] | | |
| **Physical Multimorbidity** |  |  |  |  | | |
| No physical comorbidities | 203,713 [200,877, 206,940] | 30.4% [30.2%, 30.6%] | 250,184 [234,740, 269,444] | 27.6% [26.6%, 28.5%] | | |
| 1 physical comorbidity | 210,150 [207,769, 212,659] | 31.4% [31.3%, 31.5%] | 247,158 [236,913, 258,540] | 27.2% [27.0%, 27.4%] | | |
| ≥2 physical comorbidities | 256,174 [254,248, 257,746] | 38.2% [38.0%, 38.4%] | 410,940 [402,067, 418,814] | 45.2% [44.2%, 46.3%] | | |
| **Mental and physical multimorbidity** |  |  |  |  | | |
| ≥2 mental or physical comorbidities | 421,313 [417,838, 424,409] | 62.9% [62.7%, 63.1%] | 631,834 [615,918, 649,810] | 69.5% [68.5%, 70.6%] | | |
| ≥1 mental and ≥2 physical comorbidities | 166,433 [165,253, 167,382] | 24.8% [24.7%, 25.0%] | 283,828 [279,127, 288,851] | 31.3% [30.4%, 32.0%] | | |
| **ART status** |  |  |  |  | | |
| PWH using ART | 670,036 [663,496, 676,801] | n/a | 908,504 [875,310, 946,810] | n/a | | |
| ART initiators | 33,054 [31,918, 34,295] | n/a | 33,334 [28,804, 38,593] | n/a | | |
| Disengaged from ART use^d^ | 41,572 [41,096, 42,052] | n/a | 33,186 [31,812, 34,627] | n/a | | |

Footnotes:

AA=African American

ART=antiretroviral therapy

PEARL = ProjEcting Age, multimoRbidity, and poLypharmacy in Adults with HIV

PWH=people with HIV

MSM=men who have sex with men

^a^Values represent the median and uncertainty ranges for each simulated outcome across 200 random simulation replications.

^b^MSM who also have MWID as their HIV acquisition risk factor were include in the MWID HIV acquisition risk group.

^c^Percentages in this table are calculated using the median numerator and median denominator from 200 simulation runs of the PEARL model so that percentages will sum to 100%.

^d^PEARL forecasts of PWH using ART do not include 41,572 [41,096, 42,052] and 33,186 [31,812, 34,627]

people who initiated ART but were not using ART treatments in 2020 and 2030 (respectively) and people of ‘other’ race and ethnicities.
