# Supplementary material for: The forecasted prevalence of comorbidities and multimorbidity in people with HIV in the United States through the year 2030: A modeling study
Source: PLoS Med. 2024 Jan 12;21(1):e1004325. doi: 10.1371/journal.pmed.1004325 (PMC10833859; doi:10.1371/journal.pmed.1004325)
Supplement: S5 Table — Values represent the difference in the age distribution in each subgroup [NA-ACCORD estimate—PEARL estimate]. A threshold of 5 percentage points (>5% or <-5%) is used to detect significant differences (highlighted in blue). (DOCX) [file pmed.1004325.s012.docx]

**S5 Table:** Comparing the age distributions of ART-users in PEARL to the observed data from NA-ACCORD, from 2010–2017 (Simulation validation “out-of-sample” approach). Values represent the difference in the age distribution in each subgroup [NA-ACCORD estimate - PEARL estimate]. A threshold of 5 percentage points (>5% or <-5%) is used to detect significant differences (highlighted in blue).

|  | **2010** | **2011** | **2012** | **2013** | **2014** | **2015** | **2016** | **2017** |
| --- | --- | --- | --- | --- | --- | --- | --- | --- |
| **White MSM** |  |  |  |  |  |  |  |  |
|  |  |  |  |  |  |  |  |  |
| <30 years | 0.7% | 0.8% | 0.6% | 0.8% | 0.9% | 0.6% | 0.7% | 0.8% |
|  | [4.9% vs. 4.2%] | [5.4% vs. 4.6%] | [5.7% vs. 5.1%] | [6.1% vs. 5.4%] | [6.4% vs. 5.5%] | [6.1% vs. 5.5%] | [6.1% vs. 5.4%] | [6.1% vs. 5.3%] |
| 30-39 years | -1.2% | -0.4% | 0.4% | 0.8% | 1.1% | 1.2% | 1.4% | 2.6% |
|  | [14.8% vs. 16.0%] | [14.2% vs. 14.7%] | [14.3% vs. 13.8%] | [14.1% vs. 13.3%] | [14.1% vs. 13.0%] | [14.0% vs. 12.8%] | [14.2% vs. 12.8%] | [15.5% vs. 13.0%] |
| 40-49 years | -0.9% | -0.7% | -1.5% | -1.8% | -2.5% | -2.4% | -2.1% | -1.4% |
|  | [41.0% vs. 41.9%] | [38.4% vs. 39.1%] | [34.7% vs. 36.2%] | [31.4% vs. 33.3%] | [28.0% vs. 30.5%] | [25.6% vs. 28.0%] | [23.4% vs. 25.6%] | [22.1% vs. 23.5%] |
| 50-59 years | 1.2% | 0.6% | 0.6% | 0.3% | 0.3% | 0.5% | -0.7% | -0.2% |
|  | [29.3% vs. 28.2%] | [31.1% vs. 30.6%] | [33.2% vs. 32.6%] | [34.7% vs. 34.4%] | [36.1% vs. 35.9%] | [37.5% vs. 37.0%] | [37.0% vs. 37.7%] | [37.7% vs. 37.9%] |
| 60-69 years | -0.1% | -0.5% | -0.3% | 0.0% | 0.3% | 0.2% | 0.7% | -1.1% |
|  | [8.4% vs. 8.5%] | [9.1% vs. 9.6%] | [10.2% vs. 10.5%] | [11.5% vs. 11.6%] | [12.9% vs. 12.6%] | [14.0% vs. 13.8%] | [15.8% vs. 15.1%] | [15.3% vs. 16.4%] |
| ≥70 years | 0.3% | 0.2% | 0.1% | 0.0% | 0.0% | -0.1% | 0.0% | -0.7% |
|  | [1.6% vs. 1.3%] | [1.7% vs. 1.5%] | [1.9% vs. 1.8%] | [2.2% vs. 2.1%] | [2.5% vs. 2.5%] | [2.8% vs. 2.9%] | [3.3% vs. 3.4%] | [3.2% vs. 3.9%] |
| **Black/AA MSM** |  |  |  |  |  |  |  |  |
|  |  |  |  |  |  |  |  |  |
| <30 years | 0.8% | 1.8% | 1.9% | 2.6% | 3.7% | 3.7% | 4.0% | 4.4% |
|  | [19.4% vs. 18.6%] | [21.8% vs. 20.0%] | [23.3% vs. 21.3%] | [24.4% vs. 21.8%] | [25.4% vs. 21.8%] | [25.0% vs. 21.3%] | [24.7% vs. 20.6%] | [24.2% vs. 19.8%] |
| 30-39 years | -1.5% | -0.8% | 0.5% | 1.1% | 2.1% | 2.9% | 0.8% | 1.7% |
|  | [21.2% vs. 22.8%] | [20.7% vs. 21.5%] | [21.6% vs. 21.1%] | [22.4% vs. 21.2%] | [24.0% vs. 21.9%] | [26.0% vs. 23.0%] | [25.2% vs. 24.5%] | [27.8% vs. 26.1%] |
| 40-49 years | 1.9% | 0.5% | -0.6% | -2.1% | -4.2% | -4.9% | -4.9% | -4.7% |
|  | [36.7% vs. 34.8%] | [33.5% vs. 33.1%] | [30.6% vs. 31.2%] | [27.3% vs. 29.4%] | [23.3% vs. 27.6%] | [20.9% vs. 25.8%] | [19.2% vs. 24.0%] | [17.6% vs. 22.4%] |
| 50-59 years | -1.8% | -1.5% | -1.6% | -1.2% | -0.7% | -0.3% | 0.9% | 0.3% |
|  | [18.1% vs. 19.9%] | [19.2% vs. 20.8%] | [19.7% vs. 21.3%] | [20.6% vs. 21.8%] | [21.5% vs. 22.2%] | [22.2% vs. 22.5%] | [23.6% vs. 22.7%] | [23.0% vs. 22.7%] |
| 60-69 years | 0.3% | -0.2% | -0.6% | -0.8% | -1.2% | -1.6% | -1.0% | -1.7% |
|  | [4.0% vs. 3.7%] | [4.1% vs. 4.3%] | [4.2% vs. 4.8%] | [4.6% vs. 5.4%] | [4.8% vs. 6.0%] | [5.1% vs. 6.7%] | [6.4% vs. 7.4%] | [6.4% vs. 8.1%] |
| ≥70 years | 0.3% | 0.3% | 0.4% | 0.4% | 0.4% | 0.1% | 0.3% | 0.0% |
|  | [0.6% vs. 0.2%] | [0.6% vs. 0.3%] | [0.7% vs. 0.4%] | [0.8% vs. 0.4%] | [1.0% vs. 0.5%] | [0.8% vs. 0.7%] | [1.1% vs. 0.8%] | [0.9% vs. 1.0%] |
| **Hispanic MSM** |  |  |  |  |  |  |  |  |
|  |  |  |  |  |  |  |  |  |
| <30 years | 1.5% | 1.4% | 1.1% | 1.6% | 1.0% | 0.3% | -0.4% | -0.3% |
|  | [11.5% vs. 10.0%] | [12.1% vs. 10.7%] | [12.7% vs. 11.6%] | [13.7% vs. 12.1%] | [13.4% vs. 12.4%] | [12.7% vs. 12.5%] | [12.1% vs. 12.5%] | [12.1% vs. 12.4%] |
| 30-39 years | -4.2% | -1.8% | -0.8% | -0.9% | 0.8% | 2.0% | 2.3% | 2.6% |
|  | [28.1% vs. 32.3%] | [27.9% vs. 29.6%] | [26.9% vs. 27.7%] | [25.4% vs. 26.3%] | [26.2% vs. 25.4%] | [26.9% vs. 24.9%] | [27.3% vs. 24.9%] | [27.9% vs. 25.4%] |
| 40-49 years | 3.4% | 1.7% | 0.9% | -0.3% | -2.4% | -3.3% | -4.0% | -2.5% |
|  | [42.3% vs. 38.9%] | [40.8% vs. 39.0%] | [39.5% vs. 38.6%] | [37.6% vs. 37.9%] | [34.5% vs. 36.9%] | [32.4% vs. 35.7%] | [30.2% vs. 34.2%] | [30.0% vs. 32.5%] |
| 50-59 years | -1.2% | -1.5% | -0.8% | -0.3% | 0.3% | 0.8% | 1.4% | 0.7% |
|  | [14.0% vs. 15.2%] | [15.3% vs. 16.8%] | [17.2% vs. 18.0%] | [19.0% vs. 19.3%] | [20.8% vs. 20.5%] | [22.4% vs. 21.6%] | [23.9% vs. 22.5%] | [24.0% vs. 23.3%] |
| 60-69 years | 0.6% | 0.4% | -0.3% | -0.1% | 0.4% | 0.4% | 0.5% | -0.6% |
|  | [3.3% vs. 2.7%] | [3.4% vs. 3.0%] | [2.9% vs. 3.3%] | [3.5% vs. 3.6%] | [4.4% vs. 4.0%] | [4.8% vs. 4.4%] | [5.4% vs. 4.9%] | [4.8% vs. 5.5%] |
| ≥70 years | -0.1% | -0.3% | -0.1% | -0.1% | -0.1% | -0.1% | 0.2% | 0.1% |
|  | [0.7% vs. 0.9%] | [0.6% vs. 0.9%] | [0.7% vs. 0.9%] | [0.7% vs. 0.9%] | [0.7% vs. 0.9%] | [0.8% vs. 0.9%] | [1.2% vs. 0.9%] | [1.1% vs. 1.0%] |
| **White IDU men** |  |  |  |  |  |  |  |  |
|  |  |  |  |  |  |  |  |  |
| <30 years | 0.2% | 0.1% | 0.4% | 0.6% | 0.6% | 0.3% | -0.2% | -0.2% |
|  | [2.2% vs. 2.0%] | [2.1% vs. 2.0%] | [2.6% vs. 2.2%] | [2.9% vs. 2.3%] | [3.1% vs. 2.5%] | [3.1% vs. 2.7%] | [2.9% vs. 3.1%] | [3.3% vs. 3.5%] |
| 30-39 years | 0.0% | -0.4% | -0.4% | -0.2% | 0.0% | -0.4% | 3.2% | 1.3% |
|  | [10.6% vs. 10.6%] | [9.3% vs. 9.7%] | [8.9% vs. 9.3%] | [8.9% vs. 9.1%] | [9.2% vs. 9.2%] | [8.9% vs. 9.4%] | [12.9% vs. 9.7%] | [11.5% vs. 10.2%] |
| 40-49 years | -1.1% | -0.5% | -0.9% | -2.0% | -3.2% | -2.7% | 4.0% | 5.5% |
|  | [36.0% vs. 37.1%] | [34.1% vs. 34.6%] | [31.2% vs. 32.1%] | [27.6% vs. 29.6%] | [24.0% vs. 27.2%] | [22.0% vs. 24.7%] | [26.3% vs. 22.3%] | [25.6% vs. 20.1%] |
| 50-59 years | 0.1% | 0.7% | 0.5% | 0.4% | 1.1% | 0.5% | 0.2% | 1.4% |
|  | [38.5% vs. 38.4%] | [40.5% vs. 39.8%] | [41.0% vs. 40.5%] | [41.3% vs. 40.9%] | [42.2% vs. 41.1%] | [41.3% vs. 40.9%] | [40.5% vs. 40.3%] | [40.8% vs. 39.4%] |
| 60-69 years | 0.7% | 0.1% | 0.4% | 1.3% | 1.6% | 2.2% | -5.6% | -5.7% |
|  | [11.7% vs. 11.0%] | [12.9% vs. 12.8%] | [14.8% vs. 14.5%] | [17.4% vs. 16.1%] | [19.4% vs. 17.8%] | [21.7% vs. 19.5%] | [15.5% vs. 21.1%] | [17.0% vs. 22.7%] |
| ≥70 years | 0.1% | 0.0% | 0.0% | 0.0% | -0.1% | 0.1% | -1.6% | -2.3% |
|  | [0.9% vs. 0.9%] | [1.1% vs. 1.2%] | [1.5% vs. 1.5%] | [1.8% vs. 1.9%] | [2.2% vs. 2.3%] | [2.9% vs. 2.8%] | [1.8% vs. 3.4%] | [1.8% vs. 4.1%] |
| **Black/AA IDU men** |  |  |  |  |  |  |  |  |
|  |  |  |  |  |  |  |  |  |
| <30 years | 0.1% | 0.1% | -0.1% | -0.3% | -0.4% | -0.5% | 4.5% | 7.2% |
|  | [0.6% vs. 0.5%] | [0.7% vs. 0.6%] | [0.7% vs. 0.8%] | [0.8% vs. 1.1%] | [1.1% vs. 1.5%] | [1.5% vs. 2.0%] | [7.1% vs. 2.6%] | [10.6% vs. 3.4%] |
| 30-39 years | 0.1% | 0.0% | -0.3% | -0.5% | -0.8% | -1.2% | 3.2% | 5.6% |
|  | [2.6% vs. 2.5%] | [2.6% vs. 2.5%] | [2.5% vs. 2.8%] | [2.7% vs. 3.1%] | [2.7% vs. 3.5%] | [2.6% vs. 3.8%] | [7.3% vs. 4.1%] | [9.9% vs. 4.3%] |
| 40-49 years | -1.3% | -1.1% | -1.0% | -1.2% | -1.2% | -0.8% | 3.4% | 3.2% |
|  | [20.4% vs. 21.7%] | [17.1% vs. 18.2%] | [14.3% vs. 15.3%] | [11.7% vs. 12.9%] | [9.7% vs. 10.9%] | [8.5% vs. 9.3%] | [11.5% vs. 8.1%] | [10.5% vs. 7.2%] |
| 50-59 years | 0.6% | 0.6% | 0.7% | 1.0% | 0.6% | -1.0% | -0.5% | -2.6% |
|  | [56.1% vs. 55.4%] | [55.8% vs. 55.2%] | [54.6% vs. 53.8%] | [52.7% vs. 51.6%] | [49.3% vs. 48.7%] | [44.2% vs. 45.3%] | [40.9% vs. 41.4%] | [34.6% vs. 37.3%] |
| 60-69 years | 0.0% | 0.0% | 0.6% | 0.9% | 1.9% | 3.8% | -9.1% | -10.1% |
|  | [19.1% vs. 19.1%] | [22.4% vs. 22.4%] | [26.3% vs. 25.7%] | [30.1% vs. 29.2%] | [34.5% vs. 32.6%] | [39.7% vs. 35.9%] | [29.8% vs. 38.9%] | [31.4% vs. 41.5%] |
| ≥70 years | 0.4% | 0.4% | 0.1% | 0.0% | -0.2% | -0.2% | -1.5% | -3.3% |
|  | [1.3% vs. 0.8%] | [1.5% vs. 1.1%] | [1.7% vs. 1.6%] | [2.1% vs. 2.1%] | [2.6% vs. 2.9%] | [3.6% vs. 3.8%] | [3.4% vs. 4.8%] | [2.9% vs. 6.2%] |
| **Hispanic IDU men** |  |  |  |  |  |  |  |  |
|  |  |  |  |  |  |  |  |  |
| <30 years | 0.1% | -0.6% | -0.2% | 0.2% | -0.2% | -0.1% | 3.1% | 3.6% |
|  | [1.5% vs. 1.4%] | [1.0% vs. 1.6%] | [1.7% vs. 1.9%] | [2.3% vs. 2.1%] | [2.2% vs. 2.4%] | [2.5% vs. 2.6%] | [6.0% vs. 2.9%] | [6.8% vs. 3.2%] |
| 30-39 years | -1.2% | -0.2% | 0.6% | 0.3% | 0.5% | 0.8% | 10.7% | 12.3% |
|  | [9.8% vs. 11.0%] | [10.3% vs. 10.5%] | [10.8% vs. 10.1%] | [10.0% vs. 9.8%] | [10.1% vs. 9.5%] | [10.2% vs. 9.4%] | [20.1% vs. 9.4%] | [21.8% vs. 9.5%] |
| 40-49 years | 0.4% | -0.1% | -0.8% | -2.0% | -2.1% | -0.8% | 17.3% | 13.0% |
|  | [27.5% vs. 27.2%] | [24.9% vs. 25.0%] | [22.7% vs. 23.4%] | [20.2% vs. 22.2%] | [19.1% vs. 21.2%] | [19.6% vs. 20.4%] | [37.0% vs. 19.6%] | [32.0% vs. 19.0%] |
| 50-59 years | -0.1% | -0.8% | -1.0% | -0.7% | -0.4% | -5.8% | -10.4% | -5.6% |
|  | [45.9% vs. 46.0%] | [44.7% vs. 45.5%] | [43.3% vs. 44.3%] | [42.0% vs. 42.7%] | [40.5% vs. 40.9%] | [33.0% vs. 38.8%] | [26.1% vs. 36.5%] | [28.6% vs. 34.2%] |
| 60-69 years | 0.5% | 1.3% | 0.9% | 2.1% | 2.3% | 6.0% | -19.1% | -20.6% |
|  | [14.3% vs. 13.8%] | [17.8% vs. 16.5%] | [19.9% vs. 19.0%] | [23.5% vs. 21.4%] | [26.0% vs. 23.7%] | [31.8% vs. 25.8%] | [8.7% vs. 27.8%] | [8.8% vs. 29.4%] |
| ≥70 years | 0.3% | 0.3% | 0.5% | 0.3% | -0.1% | 0.0% | -1.6% | -2.7% |
|  | [0.9% vs. 0.6%] | [1.2% vs. 0.9%] | [1.7% vs. 1.2%] | [1.9% vs. 1.7%] | [2.2% vs. 2.3%] | [2.9% vs. 2.9%] | [2.2% vs. 3.8%] | [2.0% vs. 4.8%] |
| **White IDU women** |  |  |  |  |  |  |  |  |
|  |  |  |  |  |  |  |  |  |
| <30 years | 0.6% | 1.6% | 0.7% | -0.4% | 0.3% | 0.8% | 2.1% | 0.6% |
|  | [3.9% vs. 3.3%] | [4.4% vs. 2.8%] | [3.3% vs. 2.6%] | [2.0% vs. 2.4%] | [2.5% vs. 2.2%] | [2.8% vs. 2.1%] | [4.0% vs. 2.0%] | [2.5% vs. 1.9%] |
| 30-39 years | -0.6% | 0.5% | 0.6% | 2.0% | 0.3% | -0.1% | -1.0% | 0.0% |
|  | [21.9% vs. 22.5%] | [20.7% vs. 20.2%] | [19.1% vs. 18.5%] | [19.2% vs. 17.2%] | [16.5% vs. 16.3%] | [15.4% vs. 15.4%] | [13.8% vs. 14.8%] | [14.2% vs. 14.2%] |
| 40-49 years | 0.6% | -1.8% | -2.3% | -2.0% | -2.6% | -2.0% | 2.3% | 3.0% |
|  | [41.9% vs. 41.2%] | [38.2% vs. 40.0%] | [36.0% vs. 38.3%] | [34.5% vs. 36.5%] | [31.9% vs. 34.5%] | [30.8% vs. 32.7%] | [33.3% vs. 31.0%] | [32.7% vs. 29.7%] |
| 50-59 years | 0.6% | 0.9% | 2.2% | 1.1% | 3.0% | 3.1% | -0.9% | -3.5% |
|  | [28.7% vs. 28.1%] | [31.7% vs. 30.9%] | [35.4% vs. 33.2%] | [36.0% vs. 34.9%] | [39.2% vs. 36.3%] | [40.3% vs. 37.2%] | [36.7% vs. 37.6%] | [34.2% vs. 37.6%] |
| 60-69 years | -1.6% | -1.1% | -0.9% | -0.3% | -0.2% | -0.9% | -1.6% | 0.9% |
|  | [3.1% vs. 4.7%] | [4.8% vs. 5.9%] | [6.3% vs. 7.2%] | [8.3% vs. 8.6%] | [9.9% vs. 10.1%] | [10.8% vs. 11.7%] | [11.8% vs. 13.4%] | [16.0% vs. 15.1%] |
| ≥70 years | 0.4% | -0.1% | -0.3% | -0.5% | -0.7% | -0.9% | -0.8% | -1.1% |
|  | [0.6% vs. 0.2%] | [0.2% vs. 0.3%] | [0.0% vs. 0.3%] | [0.0% vs. 0.5%] | [0.0% vs. 0.7%] | [0.0% vs. 0.9%] | [0.3% vs. 1.2%] | [0.4% vs. 1.5%] |
| **Black/AA IDU women** |  |  |  |  |  |  |  |  |
|  |  |  |  |  |  |  |  |  |
| <30 years | 0.2% | 0.3% | 0.0% | -0.4% | -0.4% | -0.4% | 1.5% | 1.9% |
|  | [0.6% vs. 0.4%] | [0.8% vs. 0.5%] | [0.5% vs. 0.5%] | [0.2% vs. 0.6%] | [0.2% vs. 0.6%] | [0.2% vs. 0.6%] | [2.2% vs. 0.7%] | [2.7% vs. 0.7%] |
| 30-39 years | -0.7% | -0.3% | 0.4% | 1.1% | -0.3% | 0.2% | 1.6% | 1.2% |
|  | [7.1% vs. 7.9%] | [6.0% vs. 6.3%] | [5.7% vs. 5.3%] | [5.8% vs. 4.8%] | [4.1% vs. 4.4%] | [4.4% vs. 4.3%] | [5.7% vs. 4.1%] | [5.3% vs. 4.1%] |
| 40-49 years | 0.4% | -3.1% | -2.9% | -2.7% | -0.8% | -2.6% | 3.8% | 3.6% |
|  | [37.6% vs. 37.1%] | [30.4% vs. 33.5%] | [27.1% vs. 30.0%] | [24.0% vs. 26.8%] | [22.9% vs. 23.7%] | [18.2% vs. 20.8%] | [22.0% vs. 18.2%] | [19.6% vs. 16.0%] |
| 50-59 years | -1.6% | 0.1% | -0.7% | -1.2% | -1.2% | 0.9% | -5.7% | -3.0% |
|  | [47.1% vs. 48.6%] | [51.6% vs. 51.5%] | [52.2% vs. 53.0%] | [52.1% vs. 53.3%] | [51.4% vs. 52.6%] | [52.0% vs. 51.1%] | [43.2% vs. 48.9%] | [43.1% vs. 46.2%] |
| 60-69 years | 1.7% | 2.9% | 2.8% | 2.5% | 2.4% | 1.9% | -1.3% | -3.8% |
|  | [7.4% vs. 5.8%] | [10.9% vs. 8.0%] | [13.6% vs. 10.8%] | [16.7% vs. 14.2%] | [20.4% vs. 18.0%] | [24.2% vs. 22.3%] | [25.6% vs. 26.8%] | [27.6% vs. 31.3%] |
| ≥70 years | 0.0% | 0.1% | 0.6% | 0.8% | 0.4% | 0.0% | 0.1% | 0.0% |
|  | [0.2% vs. 0.1%] | [0.3% vs. 0.2%] | [0.8% vs. 0.3%] | [1.2% vs. 0.4%] | [1.0% vs. 0.6%] | [0.9% vs. 0.9%] | [1.3% vs. 1.2%] | [1.8% vs. 1.8%] |
| **Hispanic IDU women** |  |  |  |  |  |  |  |  |
|  |  |  |  |  |  |  |  |  |
| <30 years | 1.2% | 0.3% | 0.1% | 0.6% | -0.3% | -0.3% | -0.2% | -0.2% |
|  | [3.5% vs. 2.3%] | [1.7% vs. 1.4%] | [1.0% vs. 0.9%] | [1.1% vs. 0.5%] | [0.0% vs. 0.3%] | [0.0% vs. 0.3%] | [0.0% vs. 0.2%] | [0.0% vs. 0.2%] |
| 30-39 years | -0.2% | 1.0% | 1.7% | 2.7% | -0.3% | -2.2% | -0.2% | 10.0% |
|  | [9.6% vs. 9.7%] | [10.4% vs. 9.4%] | [11.0% vs. 9.3%] | [12.0% vs. 9.2%] | [8.8% vs. 9.1%] | [6.7% vs. 8.9%] | [8.3% vs. 8.5%] | [18.2% vs. 8.1%] |
| 40-49 years | -2.6% | -1.7% | -6.0% | -7.2% | -4.1% | -1.3% | 5.9% | 0.6% |
|  | [35.7% vs. 38.3%] | [32.2% vs. 33.8%] | [24.0% vs. 30.0%] | [19.6% vs. 26.8%] | [19.8% vs. 23.8%] | [20.0% vs. 21.3%] | [25.0% vs. 19.1%] | [18.2% vs. 17.6%] |
| 50-59 years | 0.3% | -0.8% | -0.9% | 0.1% | -2.9% | 2.3% | -28.9% | -26.0% |
|  | [41.7% vs. 41.5%] | [43.5% vs. 44.3%] | [45.0% vs. 45.9%] | [46.7% vs. 46.7%] | [44.0% vs. 46.9%] | [48.9% vs. 46.6%] | [16.7% vs. 45.5%] | [18.2% vs. 44.1%] |
| 60-69 years | 0.7% | 0.6% | 4.7% | 2.6% | 6.7% | -1.3% | 9.3% | 0.5% |
|  | [8.7% vs. 8.0%] | [11.3% vs. 10.7%] | [18.0% vs. 13.3%] | [18.5% vs. 15.9%] | [25.3% vs. 18.6%] | [20.0% vs. 21.3%] | [33.3% vs. 24.0%] | [27.3% vs. 26.8%] |
| ≥70 years | 0.6% | 0.5% | 0.4% | 1.2% | 0.9% | 2.7% | 14.2% | 15.0% |
|  | [0.9% vs. 0.2%] | [0.9% vs. 0.4%] | [1.0% vs. 0.6%] | [2.2% vs. 0.9%] | [2.2% vs. 1.3%] | [4.4% vs. 1.8%] | [16.7% vs. 2.4%] | [18.2% vs. 3.2%] |
| **White heterosexual men** |  |  |  |  |  |  |  |  |
|  |  |  |  |  |  |  |  |  |
| <30 years | 0.4% | 0.2% | 0.0% | 1.0% | 1.7% | 1.1% | 0.2% | 0.5% |
|  | [3.0% vs. 2.6%] | [2.7% vs. 2.5%] | [2.5% vs. 2.5%] | [3.6% vs. 2.6%] | [4.4% vs. 2.6%] | [3.8% vs. 2.7%] | [3.0% vs. 2.9%] | [3.5% vs. 3.1%] |
| 30-39 years | -1.0% | -2.7% | -2.3% | -2.7% | -2.8% | -1.7% | -1.0% | -0.3% |
|  | [12.5% vs. 13.5%] | [11.1% vs. 13.8%] | [11.6% vs. 14.0%] | [11.2% vs. 13.8%] | [10.7% vs. 13.5%] | [11.2% vs. 12.9%] | [11.3% vs. 12.4%] | [11.5% vs. 11.8%] |
| 40-49 years | 0.0% | 0.4% | -0.6% | -0.7% | -1.8% | -0.1% | -2.8% | -1.4% |
|  | [37.5% vs. 37.5%] | [35.2% vs. 34.8%] | [31.8% vs. 32.4%] | [29.4% vs. 30.1%] | [26.2% vs. 28.0%] | [26.1% vs. 26.2%] | [21.6% vs. 24.4%] | [21.3% vs. 22.7%] |
| 50-59 years | -0.8% | 0.8% | 2.3% | 1.6% | 2.6% | 0.9% | 0.5% | 1.0% |
|  | [31.0% vs. 31.8%] | [34.1% vs. 33.2%] | [36.7% vs. 34.4%] | [37.1% vs. 35.5%] | [39.1% vs. 36.5%] | [37.9% vs. 37.0%] | [37.6% vs. 37.1%] | [37.8% vs. 36.9%] |
| 60-69 years | 0.5% | 0.5% | -0.1% | -0.4% | -0.2% | -0.2% | 1.6% | -1.1% |
|  | [12.3% vs. 11.8%] | [13.1% vs. 12.7%] | [13.4% vs. 13.5%] | [14.1% vs. 14.5%] | [15.5% vs. 15.6%] | [16.6% vs. 16.9%] | [19.9% vs. 18.3%] | [18.9% vs. 20.0%] |
| ≥70 years | 1.0% | 0.8% | 0.8% | 1.2% | 0.4% | -0.1% | 1.6% | 1.4% |
|  | [3.7% vs. 2.6%] | [3.8% vs. 2.9%] | [3.9% vs. 3.2%] | [4.6% vs. 3.5%] | [4.2% vs. 3.9%] | [4.3% vs. 4.3%] | [6.5% vs. 4.9%] | [6.9% vs. 5.6%] |
| **Black/AA heterosexual men** |  |  |  |  |  |  |  |  |
|  |  |  |  |  |  |  |  |  |
| <30 years | -0.2% | -0.4% | 0.0% | -0.1% | 0.1% | 0.9% | 0.7% | 0.9% |
|  | [3.7% vs. 3.9%] | [3.9% vs. 4.3%] | [4.7% vs. 4.7%] | [4.7% vs. 4.8%] | [4.9% vs. 4.8%] | [5.6% vs. 4.7%] | [5.3% vs. 4.6%] | [5.3% vs. 4.4%] |
| 30-39 years | 1.0% | 1.3% | 1.6% | 1.7% | 1.9% | 2.2% | 1.2% | 1.3% |
|  | [17.1% vs. 16.1%] | [16.0% vs. 14.7%] | [15.3% vs. 13.8%] | [14.8% vs. 13.1%] | [14.5% vs. 12.6%] | [14.5% vs. 12.4%] | [13.4% vs. 12.3%] | [13.6% vs. 12.4%] |
| 40-49 years | -1.5% | -2.0% | -2.8% | -2.6% | -2.3% | -1.4% | 0.4% | 1.6% |
|  | [40.1% vs. 41.6%] | [37.5% vs. 39.5%] | [34.5% vs. 37.2%] | [32.4% vs. 35.0%] | [30.4% vs. 32.7%] | [28.9% vs. 30.4%] | [28.5% vs. 28.1%] | [27.5% vs. 25.9%] |
| 50-59 years | 1.0% | 0.9% | 1.3% | 1.1% | 0.5% | -1.1% | -0.5% | -1.2% |
|  | [30.0% vs. 28.9%] | [31.8% vs. 30.9%] | [33.9% vs. 32.6%] | [35.3% vs. 34.2%] | [36.0% vs. 35.5%] | [35.5% vs. 36.6%] | [36.8% vs. 37.4%] | [36.5% vs. 37.7%] |
| 60-69 years | -1.1% | -0.6% | -0.5% | -0.4% | -0.4% | -0.5% | -1.1% | -1.5% |
|  | [6.8% vs. 7.9%] | [8.2% vs. 8.8%] | [9.2% vs. 9.7%] | [10.4% vs. 10.8%] | [11.5% vs. 11.9%] | [12.8% vs. 13.3%] | [13.6% vs. 14.7%] | [14.8% vs. 16.2%] |
| ≥70 years | 0.7% | 0.8% | 0.5% | 0.3% | 0.2% | -0.1% | -0.7% | -1.1% |
|  | [2.3% vs. 1.6%] | [2.5% vs. 1.8%] | [2.4% vs. 1.9%] | [2.5% vs. 2.2%] | [2.6% vs. 2.4%] | [2.6% vs. 2.7%] | [2.3% vs. 3.0%] | [2.3% vs. 3.4%] |
| **Hispanic heterosexual men** |  |  |  |  |  |  |  |  |
|  |  |  |  |  |  |  |  |  |
| <30 years | -0.1% | -0.6% | -0.2% | -0.2% | 0.1% | 0.5% | -0.6% | -0.9% |
|  | [6.0% vs. 6.0%] | [4.9% vs. 5.5%] | [4.8% vs. 5.0%] | [4.2% vs. 4.4%] | [4.1% vs. 3.9%] | [3.9% vs. 3.4%] | [2.5% vs. 3.1%] | [1.8% vs. 2.8%] |
| 30-39 years | -1.9% | -0.6% | -0.3% | -0.1% | -0.2% | 1.6% | -0.5% | -0.4% |
|  | [24.6% vs. 26.6%] | [23.6% vs. 24.2%] | [22.2% vs. 22.4%] | [20.8% vs. 20.9%] | [19.5% vs. 19.7%] | [20.1% vs. 18.5%] | [17.0% vs. 17.5%] | [16.2% vs. 16.6%] |
| 40-49 years | 0.0% | -1.2% | -2.1% | -1.0% | -1.2% | 0.4% | -1.3% | 1.5% |
|  | [36.7% vs. 36.7%] | [35.7% vs. 36.9%] | [34.3% vs. 36.4%] | [34.5% vs. 35.5%] | [33.1% vs. 34.3%] | [33.4% vs. 33.0%] | [30.5% vs. 31.8%] | [32.4% vs. 30.8%] |
| 50-59 years | 2.3% | 2.7% | 1.9% | 1.2% | 0.3% | -0.3% | 3.3% | 1.0% |
|  | [23.0% vs. 20.7%] | [25.2% vs. 22.5%] | [26.3% vs. 24.3%] | [27.2% vs. 26.1%] | [27.9% vs. 27.6%] | [28.4% vs. 28.7%] | [32.7% vs. 29.4%] | [30.9% vs. 29.9%] |
| 60-69 years | -0.5% | -0.8% | 0.1% | -0.4% | 0.6% | -1.9% | -0.5% | -0.3% |
|  | [7.5% vs. 8.0%] | [7.9% vs. 8.7%] | [9.6% vs. 9.4%] | [10.0% vs. 10.4%] | [12.2% vs. 11.6%] | [11.0% vs. 12.9%] | [13.7% vs. 14.2%] | [15.1% vs. 15.4%] |
| ≥70 years | 0.2% | 0.5% | 0.6% | 0.6% | 0.3% | -0.2% | -0.4% | -0.9% |
|  | [2.2% vs. 2.0%] | [2.7% vs. 2.2%] | [2.9% vs. 2.4%] | [3.3% vs. 2.6%] | [3.3% vs. 3.0%] | [3.2% vs. 3.4%] | [3.6% vs. 3.9%] | [3.7% vs. 4.6%] |
| **White heterosexual women** |  |  |  |  |  |  |  |  |
|  |  |  |  |  |  |  |  |  |
| <30 years | 0.8% | 1.5% | 1.0% | 1.3% | 1.3% | 1.0% | 1.3% | 1.9% |
|  | [6.4% vs. 5.6%] | [7.3% vs. 5.9%] | [7.2% vs. 6.2%] | [7.3% vs. 6.0%] | [6.8% vs. 5.6%] | [5.9% vs. 4.9%] | [5.5% vs. 4.2%] | [5.4% vs. 3.5%] |
| 30-39 years | -2.1% | -3.0% | -0.5% | 0.4% | -0.2% | 0.0% | -1.0% | -0.3% |
|  | [21.6% vs. 23.6%] | [17.7% vs. 20.7%] | [18.2% vs. 18.7%] | [17.9% vs. 17.5%] | [16.6% vs. 16.8%] | [16.3% vs. 16.3%] | [14.9% vs. 15.9%] | [15.2% vs. 15.5%] |
| 40-49 years | -1.3% | -0.2% | -1.2% | -3.0% | -2.4% | -3.1% | -5.2% | -1.6% |
|  | [37.6% vs. 38.9%] | [38.2% vs. 38.4%] | [36.0% vs. 37.2%] | [32.9% vs. 35.8%] | [31.8% vs. 34.2%] | [29.5% vs. 32.6%] | [25.8% vs. 31.0%] | [28.0% vs. 29.6%] |
| 50-59 years | 2.0% | 1.5% | 0.5% | 0.9% | 0.0% | 1.4% | 4.0% | 0.7% |
|  | [25.2% vs. 23.2%] | [26.8% vs. 25.3%] | [27.5% vs. 27.0%] | [29.4% vs. 28.6%] | [30.0% vs. 30.0%] | [32.6% vs. 31.2%] | [36.2% vs. 32.2%] | [33.6% vs. 32.9%] |
| 60-69 years | 0.6% | 0.4% | 0.4% | 0.3% | 0.8% | 0.2% | -0.3% | -1.4% |
|  | [7.6% vs. 7.1%] | [8.3% vs. 8.0%] | [9.2% vs. 8.8%] | [10.1% vs. 9.8%] | [11.6% vs. 10.8%] | [12.2% vs. 12.0%] | [12.9% vs. 13.2%] | [13.2% vs. 14.6%] |
| ≥70 years | 0.0% | -0.1% | -0.2% | 0.1% | 0.5% | 0.5% | 1.3% | 0.8% |
|  | [1.6% vs. 1.6%] | [1.7% vs. 1.9%] | [1.9% vs. 2.1%] | [2.4% vs. 2.3%] | [3.2% vs. 2.7%] | [3.5% vs. 3.0%] | [4.7% vs. 3.4%] | [4.6% vs. 3.8%] |
| **Black/AA heterosexual women** |  |  |  |  |  |  |  |  |
|  |  |  |  |  |  |  |  |  |
| <30 years | 0.0% | -0.1% | 0.2% | 0.3% | 0.4% | 0.5% | 0.3% | 0.9% |
|  | [8.9% vs. 8.8%] | [8.1% vs. 8.2%] | [7.9% vs. 7.8%] | [7.5% vs. 7.2%] | [7.0% vs. 6.6%] | [6.5% vs. 5.9%] | [5.6% vs. 5.2%] | [5.5% vs. 4.6%] |
| 30-39 years | -1.7% | -1.5% | -0.6% | 0.2% | 0.4% | 1.5% | -0.3% | -0.9% |
|  | [25.5% vs. 27.2%] | [23.9% vs. 25.3%] | [23.2% vs. 23.7%] | [22.6% vs. 22.4%] | [21.6% vs. 21.2%] | [21.8% vs. 20.2%] | [19.1% vs. 19.3%] | [17.7% vs. 18.6%] |
| 40-49 years | 1.2% | 1.8% | 0.7% | 0.3% | -0.1% | 0.1% | 1.2% | 0.8% |
|  | [36.5% vs. 35.3%] | [36.9% vs. 35.1%] | [35.3% vs. 34.6%] | [34.4% vs. 34.1%] | [33.3% vs. 33.4%] | [32.8% vs. 32.6%] | [32.9% vs. 31.7%] | [31.6% vs. 30.7%] |
| 50-59 years | 0.3% | -0.5% | -0.7% | -0.6% | -0.4% | -1.8% | -0.9% | 0.2% |
|  | [22.7% vs. 22.4%] | [23.6% vs. 24.1%] | [24.9% vs. 25.6%] | [26.3% vs. 26.8%] | [27.6% vs. 28.0%] | [27.3% vs. 29.1%] | [29.1% vs. 30.0%] | [31.0% vs. 30.8%] |
| 60-69 years | -0.3% | 0.0% | 0.2% | -0.5% | -0.7% | -1.1% | -0.8% | -1.3% |
|  | [5.3% vs. 5.6%] | [6.5% vs. 6.5%] | [7.7% vs. 7.5%] | [8.0% vs. 8.5%] | [8.8% vs. 9.5%] | [9.5% vs. 10.6%] | [11.0% vs. 11.9%] | [11.8% vs. 13.1%] |
| ≥70 years | 0.5% | 0.4% | 0.2% | 0.3% | 0.5% | 0.6% | 0.5% | 0.3% |
|  | [1.1% vs. 0.5%] | [1.1% vs. 0.7%] | [1.0% vs. 0.8%] | [1.3% vs. 1.0%] | [1.7% vs. 1.3%] | [2.2% vs. 1.5%] | [2.3% vs. 1.8%] | [2.5% vs. 2.2%] |
| **Hispanic heterosexual women** |  |  |  |  |  |  |  |  |
|  |  |  |  |  |  |  |  |  |
| <30 years | 0.5% | 0.7% | 0.9% | 0.8% | -0.2% | 0.5% | -1.2% | 1.0% |
|  | [8.7% vs. 8.2%] | [7.7% vs. 7.0%] | [7.0% vs. 6.1%] | [6.1% vs. 5.3%] | [4.6% vs. 4.7%] | [4.8% vs. 4.3%] | [2.9% vs. 4.0%] | [4.8% vs. 3.9%] |
| 30-39 years | -0.3% | -0.5% | -0.8% | -0.7% | 0.1% | 1.3% | 4.2% | 4.1% |
|  | [28.5% vs. 28.8%] | [27.4% vs. 27.9%] | [26.2% vs. 27.0%] | [25.2% vs. 25.9%] | [24.7% vs. 24.7%] | [24.7% vs. 23.4%] | [26.3% vs. 22.1%] | [25.0% vs. 20.9%] |
| 40-49 years | 1.1% | 0.8% | 0.5% | 0.9% | 1.0% | 1.2% | 2.2% | 5.0% |
|  | [34.6% vs. 33.5%] | [33.5% vs. 32.7%] | [32.4% vs. 32.0%] | [32.2% vs. 31.3%] | [31.7% vs. 30.7%] | [31.3% vs. 30.1%] | [31.7% vs. 29.5%] | [33.9% vs. 28.9%] |
| 50-59 years | -2.9% | -2.2% | -1.7% | -1.2% | -0.6% | -1.0% | -3.5% | -4.2% |
|  | [19.9% vs. 22.8%] | [22.1% vs. 24.3%] | [23.7% vs. 25.4%] | [25.1% vs. 26.3%] | [26.6% vs. 27.1%] | [26.6% vs. 27.6%] | [24.4% vs. 27.9%] | [23.8% vs. 28.0%] |
| 60-69 years | 1.4% | 0.4% | 0.6% | -0.2% | -0.8% | -2.1% | -1.9% | -5.8% |
|  | [7.5% vs. 6.1%] | [7.7% vs. 7.2%] | [9.1% vs. 8.5%] | [9.6% vs. 9.7%] | [10.2% vs. 11.0%] | [10.2% vs. 12.4%] | [11.7% vs. 13.7%] | [9.3% vs. 15.0%] |
| ≥70 years | 0.3% | 0.8% | 0.6% | 0.4% | 0.4% | 0.1% | 0.2% | 0.0% |
|  | [0.9% vs. 0.6%] | [1.6% vs. 0.8%] | [1.7% vs. 1.1%] | [1.8% vs. 1.3%] | [2.1% vs. 1.7%] | [2.3% vs. 2.2%] | [2.9% vs. 2.7%] | [3.2% vs. 3.3%] |

The discrepancies were <10% difference in all subgroups and years (15 subgroups * 8 calendar years = 120 subgroups and years) with the exception of 12 (10%) subgroup and year cells. The biggest differences were in Hispanic males and females who injected drugs, the subgroups with smallest observed sample size (and therefore largest estimate variances) in the NA-ACCORD.
