# Supplementary material for: The forecasted prevalence of comorbidities and multimorbidity in people with HIV in the United States through the year 2030: A modeling study
Source: PLoS Med. 2024 Jan 12;21(1):e1004325. doi: 10.1371/journal.pmed.1004325 (PMC10833859; doi:10.1371/journal.pmed.1004325)
Supplement: S6 Table — (DOCX) [file pmed.1004325.s013.docx]

**S6 Table:** PEARL-forecasted multimorbidity prevalence, by year^a^ and within each subgroup of PWH using ART in the US

| Subgroup | **Multimorbidity measurement** | **2009** | **2010** | | **2011** | | **2012** | | **2013** | | **2014** | | **2015** | | **2016** | | **2017** | | **2018** | | **2019** | | **2020** | | **2021** | | **2022** | | **2023** | | **2024** | | **2025** | | **2026** | | **2027** | | **2028** | | **2029** | | **2030** | |
| --- | --- | --- | --- | --- | --- | --- | --- | --- | --- | --- | --- | --- | --- | --- | --- | --- | --- | --- | --- | --- | --- | --- | --- | --- | --- | --- | --- | --- | --- | --- | --- | --- | --- | --- | --- | --- | --- | --- | --- | --- | --- | --- | --- | --- |
| Overall | ≥1 Ment. | 51% | 53% | | 54% | | 55% | | 55% | | 56% | | 57% | | 58% | | 58% | | 59% | | 59% | | 60% | | 61% | | 61% | | 61% | | 62% | | 62% | | 63% | | 63% | | 64% | | 64% | | 64% | |
| Overall | ≥2 Phys. | 25% | 28% | | 29% | | 30% | | 32% | | 33% | | 34% | | 35% | | 36% | | 37% | | 37% | | 38% | | 39% | | 40% | | 40% | | 41% | | 42% | | 43% | | 43% | | 44% | | 45% | | 45% | |
| Overall | ≥2 Any | 50% | 53% | | 54% | | 55% | | 56% | | 58% | | 59% | | 60% | | 60% | | 61% | | 62% | | 63% | | 64% | | 64% | | 65% | | 66% | | 66% | | 67% | | 68% | | 68% | | 69% | | 70% | |
| Overall | ≥1 Ment. & 2 Phys. | 13% | 15% | | 16% | | 17% | | 19% | | 20% | | 21% | | 22% | | 23% | | 23% | | 24% | | 25% | | 26% | | 26% | | 27% | | 28% | | 28% | | 29% | | 29% | | 30% | | 31% | | 31% | |
| White MSM | ≥1 Ment. | 56% | 57% | | 58% | | 59% | | 60% | | 61% | | 62% | | 63% | | 64% | | 64% | | 65% | | 66% | | 66% | | 67% | | 67% | | 68% | | 68% | | 69% | | 69% | | 70% | | 70% | | 71% | |
| White MSM | ≥2 Phys. | 25% | 27% | | 28% | | 29% | | 30% | | 31% | | 31% | | 32% | | 33% | | 34% | | 35% | | 36% | | 37% | | 38% | | 39% | | 40% | | 41% | | 42% | | 43% | | 44% | | 45% | | 46% | |
| White MSM | ≥2 Any | 52% | 54% | | 56% | | 57% | | 58% | | 59% | | 60% | | 61% | | 62% | | 63% | | 64% | | 65% | | 66% | | 67% | | 68% | | 69% | | 70% | | 70% | | 71% | | 72% | | 73% | | 74% | |
| White MSM | ≥1 Ment. & 2 Phys. | 14% | 15% | | 17% | | 18% | | 19% | | 20% | | 21% | | 22% | | 23% | | 23% | | 24% | | 25% | | 26% | | 27% | | 28% | | 29% | | 30% | | 30% | | 31% | | 32% | | 33% | | 34% | |
| Black/AA MSM | ≥1 Ment. | 43% | 44% | | 45% | | 45% | | 46% | | 46% | | 47% | | 47% | | 47% | | 48% | | 48% | | 48% | | 49% | | 49% | | 49% | | 49% | | 49% | | 49% | | 49% | | 49% | | 49% | | 49% | |
| Black/AA MSM | ≥2 Phys. | 22% | 24% | | 25% | | 25% | | 26% | | 27% | | 28% | | 29% | | 29% | | 30% | | 31% | | 32% | | 33% | | 33% | | 34% | | 35% | | 36% | | 37% | | 38% | | 39% | | 40% | | 41% | |
| Black/AA MSM | ≥2 Any | 42% | 44% | | 45% | | 45% | | 46% | | 46% | | 47% | | 48% | | 48% | | 49% | | 49% | | 50% | | 51% | | 51% | | 52% | | 53% | | 53% | | 54% | | 55% | | 56% | | 56% | | 57% | |
| Black/AA MSM | ≥1 Ment. & 2 Phys. | 9% | 11% | | 12% | | 13% | | 14% | | 15% | | 15% | | 16% | | 16% | | 17% | | 18% | | 18% | | 19% | | 19% | | 20% | | 20% | | 21% | | 21% | | 22% | | 22% | | 23% | | 23% | |
| Hispanic MSM | ≥1 Ment. | 56% | 57% | 58% | | 58% | | 59% | | 59% | | 60% | | 60% | | 61% | | 62% | | 62% | | 63% | | 63% | | 64% | | 64% | | 65% | | 65% | | 66% | | 66% | | 67% | | 67% | | 67% | |  |
| Hispanic MSM | ≥2 Phys. | 19% | 20% | 20% | | 20% | | 21% | | 21% | | 21% | | 21% | | 21% | | 21% | | 21% | | 21% | | 21% | | 22% | | 22% | | 22% | | 22% | | 22% | | 22% | | 22% | | 22% | | 22% | |  |
| Hispanic MSM | ≥2 Any | 46% | 49% | 49% | | 49% | | 50% | | 50% | | 51% | | 52% | | 52% | | 53% | | 53% | | 54% | | 54% | | 55% | | 55% | | 56% | | 56% | | 57% | | 57% | | 57% | | 58% | | 58% | |  |
| Hispanic MSM | ≥1 Ment. & 2 Phys. | 11% | 12% | 13% | | 13% | | 13% | | 14% | | 14% | | 15% | | 15% | | 15% | | 15% | | 16% | | 16% | | 16% | | 16% | | 16% | | 16% | | 17% | | 17% | | 17% | | 17% | | 17% | |  |
| White MWID | ≥1 Ment. | 60% | 62% | 63% | | 64% | | 65% | | 66% | | 67% | | 67% | | 68% | | 69% | | 70% | | 71% | | 72% | | 73% | | 73% | | 74% | | 75% | | 76% | | 77% | | 78% | | 78% | | 79% | |  |
| White MWID | ≥2 Phys. | 25% | 29% | 32% | | 34% | | 36% | | 38% | | 40% | | 41% | | 43% | | 44% | | 45% | | 45% | | 46% | | 47% | | 47% | | 48% | | 48% | | 48% | | 49% | | 49% | | 49% | | 49% | |  |
| White MWID | ≥2 Any | 56% | 60% | 62% | | 64% | | 65% | | 66% | | 68% | | 69% | | 70% | | 71% | | 72% | | 72% | | 73% | | 74% | | 74% | | 75% | | 76% | | 76% | | 77% | | 77% | | 78% | | 78% | |  |
| White MWID | ≥1 Ment. & 2 Phys. | 15% | 18% | 21% | | 23% | | 25% | | 26% | | 28% | | 29% | | 31% | | 32% | | 33% | | 34% | | 35% | | 35% | | 36% | | 37% | | 38% | | 38% | | 39% | | 39% | | 40% | | 40% | |  |
| Black/AA MWID | ≥1 Ment. | 45% | 47% | 48% | | 49% | | 51% | | 52% | | 53% | | 54% | | 56% | | 57% | | 58% | | 59% | | 61% | | 62% | | 63% | | 64% | | 66% | | 67% | | 68% | | 70% | | 71% | | 73% | |  |
| Black/AA MWID | ≥2 Phys. | 36% | 41% | 45% | | 48% | | 51% | | 54% | | 57% | | 59% | | 61% | | 63% | | 64% | | 66% | | 67% | | 68% | | 69% | | 70% | | 71% | | 72% | | 72% | | 72% | | 73% | | 73% | |  |
| Black/AA MWID | ≥2 Any | 57% | 62% | 65% | | 67% | | 70% | | 72% | | 74% | | 76% | | 77% | | 79% | | 80% | | 81% | | 83% | | 84% | | 85% | | 85% | | 86% | | 87% | | 87% | | 88% | | 88% | | 89% | |  |
| Black/AA MWID | ≥1 Ment. & 2 Phys. | 16% | 19% | 22% | | 24% | | 26% | | 28% | | 30% | | 32% | | 34% | | 36% | | 38% | | 40% | | 41% | | 43% | | 44% | | 46% | | 47% | | 48% | | 50% | | 51% | | 52% | | 53% | |  |
| Hispanic MWID | ≥1 Ment. | 56% | 58% | 59% | | 60% | | 62% | | 63% | | 64% | | 65% | | 67% | | 68% | | 69% | | 71% | | 72% | | 74% | | 75% | | 77% | | 79% | | 80% | | 82% | | 84% | | 85% | | 87% | |  |
| Hispanic MWID | ≥2 Phys. | 30% | 34% | 37% | | 39% | | 41% | | 43% | | 44% | | 46% | | 47% | | 48% | | 49% | | 50% | | 51% | | 52% | | 52% | | 53% | | 53% | | 54% | | 54% | | 54% | | 55% | | 55% | |  |
| Hispanic MWID | ≥2 Any | 58% | 61% | 63% | | 65% | | 67% | | 68% | | 70% | | 72% | | 73% | | 75% | | 76% | | 77% | | 78% | | 80% | | 81% | | 82% | | 83% | | 84% | | 84% | | 85% | | 86% | | 87% | |  |
| Hispanic MWID | ≥1 Ment. & 2 Phys. | 17% | 20% | 22% | | 24% | | 26% | | 28% | | 29% | | 31% | | 32% | | 34% | | 35% | | 37% | | 38% | | 39% | | 40% | | 42% | | 43% | | 44% | | 45% | | 46% | | 47% | | 48% | |  |
| White WWID | ≥1 Ment. | 67% | 70% | 72% | | 73% | | 74% | | 75% | | 76% | | 77% | | 77% | | 78% | | 78% | | 79% | | 79% | | 79% | | 80% | | 80% | | 81% | | 81% | | 81% | | 82% | | 82% | | 82% | |  |
| White WWID | ≥2 Phys. | 20% | 24% | 28% | | 31% | | 34% | | 37% | | 39% | | 42% | | 44% | | 46% | | 47% | | 49% | | 51% | | 52% | | 54% | | 55% | | 56% | | 58% | | 59% | | 60% | | 61% | | 62% | |  |
| White WWID | ≥2 Any | 54% | 60% | 64% | | 67% | | 70% | | 72% | | 74% | | 75% | | 77% | | 78% | | 79% | | 79% | | 80% | | 81% | | 82% | | 82% | | 83% | | 83% | | 84% | | 84% | | 85% | | 85% | |  |
| White WWID | ≥1 Ment. & 2 Phys. | 13% | 17% | 20% | | 23% | | 26% | | 28% | | 30% | | 33% | | 34% | | 36% | | 38% | | 40% | | 41% | | 43% | | 44% | | 45% | | 47% | | 48% | | 49% | | 51% | | 52% | | 53% | |  |
| Black/AA WWID | ≥1 Ment. | 58% | 62% | 64% | | 66% | | 67% | | 69% | | 70% | | 71% | | 72% | | 73% | | 74% | | 75% | | 75% | | 76% | | 77% | | 77% | | 78% | | 78% | | 78% | | 79% | | 79% | | 80% | |  |
| Black/AA WWID | ≥2 Phys. | 35% | 40% | 44% | | 47% | | 51% | | 54% | | 57% | | 59% | | 62% | | 64% | | 66% | | 68% | | 70% | | 72% | | 73% | | 74% | | 75% | | 77% | | 77% | | 78% | | 79% | | 80% | |  |
| Black/AA WWID | ≥2 Any | 63% | 68% | 71% | | 73% | | 76% | | 78% | | 80% | | 82% | | 83% | | 84% | | 85% | | 86% | | 87% | | 88% | | 89% | | 89% | | 90% | | 90% | | 91% | | 91% | | 91% | | 92% | |  |
| Black/AA WWID | ≥1 Ment. & 2 Phys. | 20% | 25% | 28% | | 31% | | 35% | | 37% | | 40% | | 43% | | 45% | | 47% | | 50% | | 52% | | 53% | | 55% | | 57% | | 58% | | 59% | | 61% | | 62% | | 63% | | 64% | | 65% | |  |
| Hispanic WWID | ≥1 Ment. | 68% | 70% | 72% | | 73% | | 74% | | 75% | | 76% | | 77% | | 78% | | 78% | | 79% | | 79% | | 79% | | 80% | | 80% | | 80% | | 80% | | 81% | | 81% | | 81% | | 81% | | 82% | |  |
| Hispanic WWID | ≥2 Phys. | 20% | 25% | 29% | | 33% | | 36% | | 40% | | 43% | | 46% | | 48% | | 51% | | 53% | | 55% | | 57% | | 59% | | 60% | | 62% | | 63% | | 64% | | 65% | | 66% | | 67% | | 68% | |  |
| Hispanic WWID | ≥2 Any | 54% | 60% | 65% | | 68% | | 71% | | 73% | | 76% | | 77% | | 79% | | 80% | | 82% | | 82% | | 83% | | 84% | | 85% | | 85% | | 85% | | 86% | | 86% | | 87% | | 87% | | 87% | |  |
| Hispanic WWID | ≥1 Ment. & 2 Phys. | 13% | 18% | 21% | | 24% | | 27% | | 30% | | 33% | | 36% | | 38% | | 40% | | 42% | | 44% | | 46% | | 48% | | 49% | | 51% | | 52% | | 53% | | 54% | | 55% | | 56% | | 58% | |  |
| White Heterosexual Men | ≥1 Ment. | 40% | 40% | 41% | | 41% | | 41% | | 41% | | 42% | | 42% | | 42% | | 42% | | 43% | | 43% | | 43% | | 43% | | 44% | | 44% | | 44% | | 45% | | 45% | | 46% | | 47% | | 47% | |  |
| White Heterosexual Men | ≥2 Phys. | 22% | 25% | 26% | | 27% | | 29% | | 30% | | 32% | | 33% | | 35% | | 37% | | 38% | | 40% | | 42% | | 44% | | 45% | | 47% | | 49% | | 51% | | 53% | | 55% | | 56% | | 58% | |  |
| White Heterosexual Men | ≥2 Any | 41% | 43% | 44% | | 45% | | 47% | | 48% | | 50% | | 51% | | 53% | | 54% | | 56% | | 57% | | 59% | | 60% | | 61% | | 63% | | 65% | | 66% | | 68% | | 69% | | 71% | | 72% | |  |
| White Heterosexual Men | ≥1 Ment. & 2 Phys. | 9% | 10% | 11% | | 12% | | 12% | | 13% | | 14% | | 15% | | 16% | | 17% | | 18% | | 18% | | 19% | | 20% | | 21% | | 22% | | 23% | | 24% | | 25% | | 26% | | 27% | | 29% | |  |
| Black/AA Heterosexual Men | ≥1 Ment. | 29% | 30% | 32% | | 33% | | 34% | | 35% | | 36% | | 37% | | 37% | | 38% | | 39% | | 39% | | 40% | | 40% | | 41% | | 41% | | 41% | | 42% | | 42% | | 42% | | 42% | | 43% | |  |
| Black/AA Heterosexual Men | ≥2 Phys. | 28% | 31% | 33% | | 34% | | 36% | | 38% | | 40% | | 42% | | 44% | | 45% | | 47% | | 49% | | 50% | | 52% | | 54% | | 55% | | 57% | | 58% | | 60% | | 62% | | 63% | | 65% | |  |
| Black/AA Heterosexual Men | ≥2 Any | 41% | 44% | 47% | | 48% | | 50% | | 52% | | 54% | | 56% | | 58% | | 59% | | 61% | | 62% | | 64% | | 65% | | 66% | | 68% | | 69% | | 70% | | 72% | | 73% | | 74% | | 75% | |  |
| Black/AA Heterosexual Men | ≥1 Ment. & 2 Phys. | 8% | 9% | 10% | | 11% | | 12% | | 14% | | 15% | | 16% | | 17% | | 18% | | 19% | | 20% | | 21% | | 22% | | 23% | | 23% | | 24% | | 25% | | 26% | | 27% | | 28% | | 29% | |  |
| Hispanic Heterosexual Men | ≥1 Ment. | 39% | 42% | 44% | | 45% | | 46% | | 47% | | 47% | | 48% | | 49% | | 49% | | 49% | | 49% | | 50% | | 50% | | 50% | | 50% | | 51% | | 51% | | 51% | | 52% | | 52% | | 52% | |  |
| Hispanic Heterosexual Men | ≥2 Phys. | 19% | 21% | 23% | | 25% | | 27% | | 29% | | 30% | | 32% | | 34% | | 36% | | 38% | | 40% | | 42% | | 44% | | 46% | | 48% | | 49% | | 51% | | 53% | | 55% | | 57% | | 59% | |  |
| Hispanic Heterosexual Men | ≥2 Any | 37% | 42% | 44% | | 46% | | 49% | | 51% | | 53% | | 55% | | 56% | | 58% | | 60% | | 61% | | 63% | | 64% | | 66% | | 67% | | 68% | | 70% | | 71% | | 73% | | 74% | | 76% | |  |
| Hispanic Heterosexual Men | ≥1 Ment. & 2 Phys. | 7% | 9% | 10% | | 12% | | 13% | | 14% | | 15% | | 16% | | 18% | | 19% | | 20% | | 21% | | 22% | | 23% | | 24% | | 25% | | 26% | | 27% | | 29% | | 30% | | 31% | | 32% | |  |
| White Heterosexual Women | ≥1 Ment. | 59% | 60% | 62% | | 63% | | 64% | | 66% | | 67% | | 68% | | 69% | | 69% | | 70% | | 71% | | 72% | | 72% | | 73% | | 73% | | 74% | | 75% | | 75% | | 76% | | 76% | | 77% | |  |
| White Heterosexual Women | ≥2 Phys. | 24% | 28% | 31% | | 33% | | 35% | | 37% | | 39% | | 40% | | 42% | | 43% | | 45% | | 46% | | 47% | | 48% | | 49% | | 50% | | 51% | | 52% | | 52% | | 53% | | 54% | | 55% | |  |
| White Heterosexual Women | ≥2 Any | 53% | 58% | 61% | | 63% | | 65% | | 67% | | 68% | | 70% | | 71% | | 72% | | 73% | | 74% | | 75% | | 75% | | 76% | | 77% | | 77% | | 78% | | 79% | | 79% | | 80% | | 81% | |  |
| White Heterosexual Women | ≥1 Ment. & 2 Phys. | 14% | 17% | 19% | | 22% | | 24% | | 25% | | 27% | | 29% | | 30% | | 32% | | 33% | | 34% | | 35% | | 37% | | 38% | | 39% | | 40% | | 41% | | 42% | | 43% | | 44% | | 45% | |  |
| Black/AA Heterosexual Women | ≥1 Ment. | 47% | 50% | 52% | | 54% | | 55% | | 56% | | 57% | | 58% | | 59% | | 60% | | 60% | | 61% | | 61% | | 62% | | 62% | | 63% | | 63% | | 64% | | 64% | | 65% | | 66% | | 66% | |  |
| Black/AA Heterosexual Women | ≥2 Phys. | 28% | 31% | 33% | | 34% | | 36% | | 38% | | 40% | | 41% | | 43% | | 45% | | 46% | | 48% | | 49% | | 51% | | 52% | | 54% | | 56% | | 57% | | 59% | | 60% | | 62% | | 63% | |  |
| Black/AA Heterosexual Women | ≥2 Any | 50% | 54% | 56% | | 58% | | 60% | | 62% | | 64% | | 65% | | 67% | | 68% | | 69% | | 70% | | 72% | | 73% | | 74% | | 75% | | 76% | | 77% | | 78% | | 80% | | 81% | | 82% | |  |
| Black/AA Heterosexual Women | ≥1 Ment. & 2 Phys. | 13% | 16% | 18% | | 19% | | 21% | | 22% | | 24% | | 25% | | 27% | | 28% | | 29% | | 30% | | 32% | | 33% | | 34% | | 35% | | 37% | | 38% | | 39% | | 41% | | 42% | | 43% | |  |
| Hispanic Heterosexual Women | ≥1 Ment. | 56% | 59% | 61% | | 62% | | 64% | | 65% | | 67% | | 68% | | 69% | | 70% | | 71% | | 72% | | 73% | | 75% | | 76% | | 77% | | 79% | | 80% | | 81% | | 83% | | 84% | | 86% | |  |
| Hispanic Heterosexual Women | ≥2 Phys. | 20% | 24% | 27% | | 30% | | 32% | | 34% | | 36% | | 38% | | 40% | | 41% | | 42% | | 44% | | 45% | | 46% | | 47% | | 48% | | 49% | | 50% | | 51% | | 52% | | 53% | | 54% | |  |
| Hispanic Heterosexual Women | ≥2 Any | 47% | 53% | 56% | | 59% | | 62% | | 64% | | 66% | | 68% | | 70% | | 71% | | 73% | | 74% | | 76% | | 77% | | 78% | | 79% | | 81% | | 82% | | 83% | | 84% | | 85% | | 86% | |  |
| Hispanic Heterosexual Women | ≥1 Ment. & 2 Phys. | 11% | 15% | 17% | | 19% | | 21% | | 23% | | 25% | | 27% | | 28% | | 30% | | 31% | | 33% | | 34% | | 35% | | 37% | | 38% | | 39% | | 41% | | 42% | | 44% | | 45% | | 46% | |  |

Footnotes:

AA=African American

PEARL = ProjEcting Age, multimoRbidity, and poLypharmacy in Adults with HIV

PWH=people with HIV

MSM=men who have sex with men

MWID=men who had injection drug use as their HIV acquisition risk factor

WWID=men who had injection drug use as their HIV acquisition risk factor

≥1 Ment. = Anxiety or depression or both (i.e., ≥1 of the mental comorbidities included)

≥2 Phys. = physical multimorbidity, defined as ≥2 physical comorbidities

≥2 Any = physical or mental multimorbidity, defined as ≥2 physical or mental comorbidities

≥1 Ment. & 2 Phys. = mental comorbidity and physical multimorbidity, defined as ≥1 mental comorbidity and ≥2 physical comorbidities

^a^Although these estimates are all PEARL forecasts, 2010 was during the calibration period (where observed NA-ACCORD data were available to inform the estimates) and 2020 and 2030 were forecast periods (without observed NA-ACCORD data).
