# Supplementary material for: The forecasted prevalence of comorbidities and multimorbidity in people with HIV in the United States through the year 2030: A modeling study
Source: PLoS Med. 2024 Jan 12;21(1):e1004325. doi: 10.1371/journal.pmed.1004325 (PMC10833859; doi:10.1371/journal.pmed.1004325)
Supplement: S7 Table — (DOCX) [file pmed.1004325.s014.docx]

**S7 Table:** PEARL-forecasted comorbidity and multimorbidity prevalence [95% uncertainty range], by subgroup, in 2010^a^, 2020, and 2030

| **Subgroup** | **Comorbidity /**  **Multimorbidity** | **2010** | **2020** | **2030** |
| --- | --- | --- | --- | --- |
| Overall | Anxiety | 24.0% [23.9%, 24.2%] | 36.4% [36.3%, 36.6%] | 46.8% [46.3%, 47.5%] |
| Overall | Depression | 39.9% [39.7%, 40.0%] | 47.0% [46.9%, 47.2%] | 48.6% [48.2%, 49.1%] |
| Overall | CKD | 10.2% [10.1%, 10.3%] | 18.8% [18.7%, 19.0%] | 30.1% [29.4%, 30.8%] |
| Overall | Dyslipidemia | 32.0% [31.9%, 32.2%] | 42.3% [42.2%, 42.5%] | 48.0% [47.1%, 48.9%] |
| Overall | Diabetes | 11.8% [11.7%, 11.9%] | 17.8% [17.6%, 17.9%] | 27.1% [26.4%, 27.7%] |
| Overall | Hypertension | 37.1% [36.9%, 37.2%] | 36.8% [36.6%, 37.0%] | 32.5% [31.8%, 33.1%] |
| Overall | Cancer | 9.5% [9.4%, 9.6%] | 11.2% [11.1%, 11.2%] | 11.2% [11.0%, 11.4%] |
| Overall | ESLD | 1.2% [1.2%, 1.2%] | 1.3% [1.3%, 1.4%] | 1.4% [1.4%, 1.5%] |
| Overall | MI | 1.6% [1.5%, 1.6%] | 3.3% [3.2%, 3.3%] | 8.1% [7.9%, 8.3%] |
| Overall | ≥1 Ment. | 53.0% [52.9%, 53.2%] | 60.0% [59.9%, 60.2%] | 64.4% [63.8%, 65.0%] |
| Overall | ≥2 Phys. | 27.6% [27.4%, 27.7%] | 38.2% [38.0%, 38.4%] | 45.2% [44.2%, 46.3%] |
| Overall | ≥2 Any | 52.8% [52.7%, 53.0%] | 62.9% [62.7%, 63.1%] | 69.5% [68.5%, 70.6%] |
| Overall | ≥1 Ment. & ≥2 Phys. | 14.8% [14.7%, 14.9%] | 24.8% [24.7%, 25.0%] | 31.3% [30.4%, 32.0%] |
| White MSM | Anxiety | 28.8% [28.5%, 29.0%] | 44.1% [43.8%, 44.4%] | 53.8% [52.5%, 55.1%] |
| White MSM | Depression | 41.7% [41.4%, 42.0%] | 49.5% [49.1%, 49.7%] | 53.0% [51.8%, 54.4%] |
| White MSM | CKD | 8.2% [8.1%, 8.4%] | 15.4% [15.2%, 15.6%] | 26.0% [24.7%, 27.5%] |
| White MSM | Dyslipidemia | 36.8% [36.5%, 37.1%] | 47.3% [46.9%, 47.7%] | 58.3% [56.1%, 60.5%] |
| White MSM | Diabetes | 7.9% [7.7%, 8.0%] | 11.6% [11.4%, 11.8%] | 19.2% [18.2%, 20.2%] |
| White MSM | Hypertension | 31.4% [31.2%, 31.7%] | 30.7% [30.4%, 31.0%] | 28.6% [27.6%, 29.5%] |
| White MSM | Cancer | 13.7% [13.6%, 13.9%] | 14.8% [14.5%, 15.0%] | 16.0% [15.3%, 16.8%] |
| White MSM | ESLD | 1.1% [1.0%, 1.2%] | 1.3% [1.2%, 1.3%] | 1.6% [1.5%, 1.6%] |
| White MSM | MI | 1.9% [1.8%, 2.0%] | 4.0% [3.9%, 4.0%] | 11.2% [10.4%, 11.9%] |
| White MSM | ≥1 Ment. | 57.3% [57.0%, 57.6%] | 65.6% [65.4%, 65.9%] | 70.6% [69.5%, 71.9%] |
| White MSM | ≥2 Phys. | 26.7% [26.4%, 26.9%] | 36.1% [35.8%, 36.5%] | 46.3% [43.9%, 48.7%] |
| White MSM | ≥2 Any | 54.5% [54.2%, 54.8%] | 65.0% [64.6%, 65.4%] | 74.0% [71.6%, 76.4%] |
| White MSM | ≥1 Ment. & ≥2 Phys. | 15.5% [15.3%, 15.7%] | 25.1% [24.8%, 25.5%] | 34.3% [32.3%, 36.2%] |
| Black/AA MSM | Anxiety | 15.5% [15.3%, 15.7%] | 22.4% [22.2%, 22.6%] | 26.5% [26.1%, 27.1%] |
| Black/AA MSM | Depression | 35.4% [35.1%, 35.7%] | 39.7% [39.5%, 40.0%] | 39.2% [38.7%, 40.0%] |
| Black/AA MSM | CKD | 9.1% [8.9%, 9.4%] | 18.3% [18.0%, 18.5%] | 37.2% [36.4%, 38.1%] |
| Black/AA MSM | Dyslipidemia | 26.3% [25.9%, 26.7%] | 29.5% [29.2%, 29.8%] | 31.8% [31.1%, 32.8%] |
| Black/AA MSM | Diabetes | 10.5% [10.3%, 10.7%] | 16.9% [16.7%, 17.1%] | 28.5% [27.8%, 29.4%] |
| Black/AA MSM | Hypertension | 38.7% [38.4%, 39.1%] | 37.6% [37.3%, 37.9%] | 33.5% [33.0%, 34.2%] |
| Black/AA MSM | Cancer | 7.3% [7.1%, 7.5%] | 8.1% [8.0%, 8.3%] | 8.4% [8.2%, 8.7%] |
| Black/AA MSM | ESLD | 1.1% [1.0%, 1.2%] | 1.2% [1.2%, 1.3%] | 1.4% [1.4%, 1.5%] |
| Black/AA MSM | MI | 1.4% [1.3%, 1.5%] | 2.3% [2.3%, 2.4%] | 5.4% [5.1%, 5.7%] |
| Black/AA MSM | ≥1 Ment. | 44.4% [44.1%, 44.8%] | 48.4% [48.1%, 48.6%] | 49.3% [48.8%, 50.1%] |
| Black/AA MSM | ≥2 Phys. | 23.9% [23.7%, 24.3%] | 31.7% [31.4%, 32.1%] | 40.7% [39.8%, 42.0%] |
| Black/AA MSM | ≥2 Any | 44.4% [44.0%, 44.8%] | 50.1% [49.9%, 50.5%] | 57.3% [56.4%, 58.7%] |
| Black/AA MSM | ≥1 Ment. & ≥2 Phys. | 11.2% [11.0%, 11.4%] | 18.1% [17.8%, 18.3%] | 23.1% [22.4%, 24.0%] |
| Hispanic MSM | Anxiety | 29.1% [28.7%, 29.5%] | 43.1% [42.8%, 43.5%] | 52.9% [51.6%, 54.2%] |
| Hispanic MSM | Depression | 43.0% [42.5%, 43.4%] | 50.8% [50.5%, 51.1%] | 53.7% [52.4%, 55.1%] |
| Hispanic MSM | CKD | 7.7% [7.5%, 7.9%] | 9.2% [9.1%, 9.4%] | 12.5% [11.9%, 13.3%] |
| Hispanic MSM | Dyslipidemia | 31.4% [30.9%, 31.8%] | 33.1% [32.7%, 33.5%] | 32.7% [31.5%, 34.1%] |
| Hispanic MSM | Diabetes | 9.2% [9.0%, 9.5%] | 9.0% [8.8%, 9.2%] | 11.5% [10.9%, 12.1%] |
| Hispanic MSM | Hypertension | 22.5% [22.1%, 22.8%] | 19.0% [18.8%, 19.3%] | 15.4% [14.8%, 15.9%] |
| Hispanic MSM | Cancer | 11.0% [10.7%, 11.2%] | 10.2% [10.0%, 10.4%] | 9.7% [9.3%, 10.0%] |
| Hispanic MSM | ESLD | 1.1% [1.0%, 1.2%] | 1.0% [1.0%, 1.1%] | 1.1% [1.0%, 1.2%] |
| Hispanic MSM | MI | 1.4% [1.3%, 1.5%] | 2.1% [2.0%, 2.2%] | 4.5% [4.2%, 4.8%] |
| Hispanic MSM | ≥1 Ment. | 57.5% [57.1%, 58.0%] | 62.9% [62.5%, 63.2%] | 67.4% [66.3%, 68.6%] |
| Hispanic MSM | ≥2 Phys. | 20.1% [19.7%, 20.5%] | 21.4% [21.0%, 21.7%] | 22.1% [20.9%, 23.5%] |
| Hispanic MSM | ≥2 Any | 48.8% [48.3%, 49.2%] | 53.8% [53.3%, 54.2%] | 58.2% [56.4%, 60.2%] |
| Hispanic MSM | ≥1 Ment. & ≥2 Phys. | 12.0% [11.7%, 12.3%] | 15.5% [15.3%, 15.8%] | 17.1% [16.1%, 18.2%] |
| White MWID | Anxiety | 38.4% [37.7%, 39.0%] | 50.8% [50.1%, 51.6%] | 65.1% [63.7%, 66.6%] |
| White MWID | Depression | 39.2% [38.5%, 39.9%] | 46.3% [45.5%, 46.9%] | 51.4% [50.5%, 52.5%] |
| White MWID | CKD | 8.7% [8.2%, 9.1%] | 20.5% [19.9%, 21.0%] | 38.0% [36.1%, 40.2%] |
| White MWID | Dyslipidemia | 34.2% [33.4%, 34.9%] | 46.1% [45.1%, 46.8%] | 42.0% [39.8%, 44.2%] |
| White MWID | Diabetes | 12.4% [12.0%, 13.0%] | 21.4% [20.9%, 22.0%] | 26.0% [24.2%, 27.9%] |
| White MWID | Hypertension | 40.6% [40.0%, 41.3%] | 42.4% [41.6%, 43.2%] | 37.9% [36.6%, 39.6%] |
| White MWID | Cancer | 8.6% [8.2%, 9.1%] | 15.7% [15.2%, 16.1%] | 20.0% [18.7%, 21.3%] |
| White MWID | ESLD | 1.5% [1.3%, 1.6%] | 2.4% [2.2%, 2.7%] | 2.9% [2.6%, 3.2%] |
| White MWID | MI | 1.6% [1.4%, 1.8%] | 5.1% [4.8%, 5.4%] | 11.2% [10.1%, 12.4%] |
| White MWID | ≥1 Ment. | 62.1% [61.5%, 62.7%] | 70.9% [70.2%, 71.5%] | 79.4% [78.1%, 80.4%] |
| White MWID | ≥2 Phys. | 29.2% [28.5%, 29.8%] | 45.4% [44.5%, 46.4%] | 48.9% [46.0%, 51.9%] |
| White MWID | ≥2 Any | 59.6% [58.8%, 60.3%] | 72.4% [71.4%, 73.3%] | 78.5% [76.3%, 80.7%] |
| White MWID | ≥1 Ment. & ≥2 Phys. | 18.4% [17.9%, 19.0%] | 33.7% [32.8%, 34.5%] | 40.2% [37.6%, 42.6%] |
| Black/AA MWID | Anxiety | 21.0% [20.5%, 21.6%] | 33.5% [32.9%, 34.3%] | 55.2% [54.2%, 56.1%] |
| Black/AA MWID | Depression | 33.0% [32.2%, 33.6%] | 42.5% [41.9%, 43.3%] | 48.2% [46.8%, 49.7%] |
| Black/AA MWID | CKD | 16.6% [16.0%, 17.0%] | 33.3% [32.5%, 34.2%] | 36.6% [33.8%, 41.4%] |
| Black/AA MWID | Dyslipidemia | 27.2% [26.6%, 27.8%] | 58.0% [57.3%, 59.1%] | 77.5% [74.4%, 82.5%] |
| Black/AA MWID | Diabetes | 21.5% [21.0%, 22.1%] | 31.5% [30.7%, 32.2%] | 36.9% [34.4%, 40.8%] |
| Black/AA MWID | Hypertension | 60.1% [59.4%, 60.9%] | 62.5% [61.9%, 63.4%] | 57.8% [56.5%, 59.8%] |
| Black/AA MWID | Cancer | 8.9% [8.5%, 9.3%] | 18.6% [17.9%, 19.2%] | 22.7% [20.1%, 26.3%] |
| Black/AA MWID | ESLD | 1.7% [1.6%, 1.9%] | 3.3% [3.0%, 3.5%] | 3.5% [3.1%, 4.2%] |
| Black/AA MWID | MI | 1.6% [1.4%, 1.8%] | 6.8% [6.4%, 7.1%] | 18.2% [15.7%, 22.4%] |
| Black/AA MWID | ≥1 Ment. | 46.7% [46.1%, 47.4%] | 59.4% [58.8%, 60.2%] | 72.6% [71.5%, 73.6%] |
| Black/AA MWID | ≥2 Phys. | 41.4% [40.7%, 42.1%] | 65.9% [65.0%, 66.9%] | 72.8% [69.2%, 78.4%] |
| Black/AA MWID | ≥2 Any | 61.7% [61.1%, 62.4%] | 81.4% [80.7%, 82.2%] | 88.9% [86.8%, 92.1%] |
| Black/AA MWID | ≥1 Ment. & ≥2 Phys. | 19.4% [18.9%, 20.0%] | 39.7% [38.9%, 40.5%] | 53.3% [50.8%, 57.5%] |
| Hispanic MWID | Anxiety | 34.7% [34.0%, 35.5%] | 55.1% [54.1%, 56.1%] | 83.4% [81.8%, 85.3%] |
| Hispanic MWID | Depression | 37.3% [36.5%, 38.2%] | 50.7% [49.9%, 51.5%] | 57.7% [56.6%, 59.1%] |
| Hispanic MWID | CKD | 8.8% [8.3%, 9.3%] | 22.1% [21.4%, 22.9%] | 38.6% [35.6%, 43.1%] |
| Hispanic MWID | Dyslipidemia | 28.1% [27.3%, 28.8%] | 45.6% [44.6%, 46.7%] | 45.6% [42.8%, 49.8%] |
| Hispanic MWID | Diabetes | 24.3% [23.5%, 25.0%] | 29.0% [28.1%, 30.0%] | 35.5% [33.0%, 39.2%] |
| Hispanic MWID | Hypertension | 46.3% [45.4%, 47.2%] | 47.1% [45.9%, 48.2%] | 42.8% [40.6%, 46.1%] |
| Hispanic MWID | Cancer | 8.8% [8.3%, 9.3%] | 17.0% [16.4%, 17.7%] | 22.1% [20.2%, 24.9%] |
| Hispanic MWID | ESLD | 1.7% [1.4%, 1.9%] | 3.0% [2.7%, 3.3%] | 3.3% [2.9%, 3.9%] |
| Hispanic MWID | MI | 1.6% [1.3%, 1.8%] | 5.8% [5.5%, 6.2%] | 13.4% [11.7%, 16.1%] |
| Hispanic MWID | ≥1 Ment. | 58.1% [57.2%, 58.8%] | 70.9% [70.0%, 71.8%] | 86.8% [85.7%, 88.2%] |
| Hispanic MWID | ≥2 Phys. | 34.2% [33.3%, 35.2%] | 50.4% [49.2%, 51.9%] | 54.7% [50.8%, 60.9%] |
| Hispanic MWID | ≥2 Any | 61.3% [60.3%, 62.2%] | 77.2% [76.1%, 78.5%] | 86.8% [84.4%, 90.1%] |
| Hispanic MWID | ≥1 Ment. & ≥2 Phys. | 20.2% [19.4%, 20.8%] | 36.6% [35.6%, 37.9%] | 48.2% [44.7%, 53.8%] |
| White WWID | Anxiety | 39.6% [38.4%, 40.7%] | 60.6% [59.8%, 61.5%] | 72.0% [71.1%, 73.1%] |
| White WWID | Depression | 51.8% [50.7%, 52.6%] | 54.4% [53.3%, 55.4%] | 49.4% [48.4%, 50.6%] |
| White WWID | CKD | 19.2% [18.3%, 20.2%] | 37.2% [35.9%, 38.2%] | 44.6% [43.0%, 46.3%] |
| White WWID | Dyslipidemia | 22.6% [21.7%, 23.7%] | 43.9% [43.0%, 45.3%] | 61.3% [59.7%, 62.8%] |
| White WWID | Diabetes | 14.7% [13.9%, 15.6%] | 26.3% [25.5%, 27.2%] | 43.6% [42.7%, 44.5%] |
| White WWID | Hypertension | 28.3% [27.6%, 29.4%] | 39.9% [39.1%, 41.0%] | 40.2% [39.4%, 41.1%] |
| White WWID | Cancer | 7.2% [6.4%, 7.7%] | 9.0% [8.4%, 9.6%] | 6.7% [6.3%, 7.3%] |
| White WWID | ESLD | 1.6% [1.4%, 1.9%] | 2.6% [2.4%, 3.0%] | 2.8% [2.5%, 3.1%] |
| White WWID | MI | 1.5% [1.2%, 1.8%] | 3.7% [3.3%, 4.1%] | 10.6% [9.8%, 11.3%] |
| White WWID | ≥1 Ment. | 70.2% [69.1%, 71.2%] | 78.7% [77.9%, 79.6%] | 82.4% [81.7%, 83.5%] |
| White WWID | ≥2 Phys. | 24.4% [23.4%, 25.3%] | 49.0% [48.2%, 50.3%] | 62.3% [60.9%, 63.8%] |
| White WWID | ≥2 Any | 60.0% [58.9%, 61.2%] | 79.5% [78.7%, 80.3%] | 85.0% [84.1%, 86.1%] |
| White WWID | ≥1 Ment. & ≥2 Phys. | 17.2% [16.3%, 17.9%] | 39.6% [38.6%, 40.9%] | 53.0% [51.7%, 54.6%] |
| Black/AA WWID | Anxiety | 23.7% [23.0%, 24.4%] | 46.0% [45.2%, 46.9%] | 59.7% [58.3%, 61.7%] |
| Black/AA WWID | Depression | 49.8% [48.9%, 50.8%] | 55.9% [55.1%, 56.7%] | 53.9% [51.7%, 56.7%] |
| Black/AA WWID | CKD | 20.1% [19.3%, 20.8%] | 49.5% [48.4%, 50.6%] | 65.2% [60.9%, 71.4%] |
| Black/AA WWID | Dyslipidemia | 29.2% [28.5%, 29.9%] | 58.5% [57.3%, 59.7%] | 79.3% [75.3%, 84.9%] |
| Black/AA WWID | Diabetes | 15.1% [14.5%, 15.6%] | 32.2% [31.5%, 33.2%] | 51.4% [49.0%, 54.4%] |
| Black/AA WWID | Hypertension | 59.2% [58.4%, 59.9%] | 61.9% [61.1%, 62.9%] | 57.7% [55.3%, 60.9%] |
| Black/AA WWID | Cancer | 7.4% [7.0%, 7.9%] | 10.8% [10.2%, 11.3%] | 8.5% [7.8%, 9.3%] |
| Black/AA WWID | ESLD | 1.6% [1.4%, 1.8%] | 2.7% [2.5%, 3.0%] | 2.9% [2.5%, 3.3%] |
| Black/AA WWID | MI | 1.6% [1.4%, 1.8%] | 5.8% [5.5%, 6.2%] | 19.7% [17.6%, 22.6%] |
| Black/AA WWID | ≥1 Ment. | 61.5% [60.7%, 62.2%] | 74.8% [74.1%, 75.6%] | 79.5% [77.8%, 81.8%] |
| Black/AA WWID | ≥2 Phys. | 40.0% [39.2%, 40.8%] | 68.1% [67.0%, 69.5%] | 80.0% [75.8%, 85.4%] |
| Black/AA WWID | ≥2 Any | 67.6% [66.8%, 68.3%] | 86.5% [85.6%, 87.3%] | 91.7% [89.2%, 95.2%] |
| Black/AA WWID | ≥1 Ment. & ≥2 Phys. | 24.7% [23.9%, 25.4%] | 51.6% [50.5%, 52.8%] | 64.6% [60.9%, 70.1%] |
| Hispanic WWID | Anxiety | 39.4% [38.0%, 40.9%] | 59.8% [58.2%, 61.5%] | 69.9% [68.1%, 73.3%] |
| Hispanic WWID | Depression | 51.7% [50.3%, 53.2%] | 55.6% [54.0%, 57.2%] | 50.8% [48.5%, 55.7%] |
| Hispanic WWID | CKD | 20.1% [18.9%, 21.3%] | 45.1% [43.1%, 47.1%] | 54.1% [50.0%, 64.6%] |
| Hispanic WWID | Dyslipidemia | 23.0% [21.8%, 24.6%] | 50.3% [48.5%, 52.3%] | 68.7% [64.5%, 78.3%] |
| Hispanic WWID | Diabetes | 14.6% [13.6%, 15.6%] | 26.9% [25.5%, 28.4%] | 43.6% [41.9%, 47.6%] |
| Hispanic WWID | Hypertension | 28.7% [27.2%, 30.1%] | 41.1% [39.5%, 42.4%] | 41.3% [39.9%, 42.9%] |
| Hispanic WWID | Cancer | 7.4% [6.5%, 8.2%] | 9.8% [8.8%, 10.7%] | 7.2% [6.3%, 8.6%] |
| Hispanic WWID | ESLD | 1.5% [1.2%, 2.0%] | 2.4% [2.0%, 2.9%] | 2.5% [2.1%, 3.2%] |
| Hispanic WWID | MI | 1.5% [1.2%, 1.9%] | 4.4% [3.8%, 5.1%] | 13.4% [12.0%, 17.5%] |
| Hispanic WWID | ≥1 Ment. | 70.1% [68.8%, 71.4%] | 79.0% [77.7%, 80.5%] | 81.7% [80.1%, 85.3%] |
| Hispanic WWID | ≥2 Phys. | 25.0% [23.9%, 26.4%] | 55.1% [53.4%, 57.0%] | 68.3% [64.8%, 77.5%] |
| Hispanic WWID | ≥2 Any | 60.4% [59.0%, 61.9%] | 82.5% [81.0%, 84.2%] | 87.2% [84.8%, 93.3%] |
| Hispanic WWID | ≥1 Ment. & ≥2 Phys. | 17.7% [16.5%, 18.8%] | 44.4% [42.6%, 46.2%] | 57.5% [53.9%, 66.5%] |
| White Heterosexual Men | Anxiety | 14.9% [13.9%, 16.0%] | 23.1% [22.2%, 24.1%] | 36.0% [35.0%, 37.0%] |
| White Heterosexual Men | Depression | 31.1% [29.5%, 32.4%] | 33.0% [31.9%, 34.1%] | 29.5% [27.9%, 31.1%] |
| White Heterosexual Men | CKD | 9.9% [9.1%, 10.7%] | 21.9% [20.9%, 22.9%] | 42.7% [41.2%, 44.7%] |
| White Heterosexual Men | Dyslipidemia | 33.0% [31.4%, 34.6%] | 46.2% [45.1%, 47.7%] | 64.4% [62.7%, 66.4%] |
| White Heterosexual Men | Diabetes | 10.4% [9.5%, 11.5%] | 17.1% [16.4%, 17.9%] | 29.7% [28.7%, 31.0%] |
| White Heterosexual Men | Hypertension | 31.9% [30.4%, 33.5%] | 36.5% [35.4%, 37.6%] | 39.0% [37.4%, 41.1%] |
| White Heterosexual Men | Cancer | 8.6% [7.6%, 9.4%] | 11.5% [10.7%, 12.2%] | 13.5% [12.6%, 14.6%] |
| White Heterosexual Men | ESLD | 1.1% [0.8%, 1.5%] | 0.9% [0.7%, 1.1%] | 0.6% [0.5%, 0.8%] |
| White Heterosexual Men | MI | 1.4% [1.1%, 1.9%] | 2.5% [2.1%, 2.8%] | 5.4% [4.7%, 6.3%] |
| White Heterosexual Men | ≥1 Ment. | 40.5% [38.8%, 42.0%] | 42.9% [41.6%, 44.1%] | 47.4% [46.0%, 48.7%] |
| White Heterosexual Men | ≥2 Phys. | 24.8% [23.4%, 26.0%] | 40.2% [39.0%, 41.3%] | 58.3% [56.3%, 60.8%] |
| White Heterosexual Men | ≥2 Any | 43.3% [41.5%, 44.8%] | 57.1% [55.8%, 58.3%] | 72.3% [70.3%, 74.9%] |
| White Heterosexual Men | ≥1 Ment. & ≥2 Phys. | 10.2% [9.3%, 11.2%] | 18.5% [17.6%, 19.3%] | 28.7% [27.1%, 30.5%] |
| Black/AA Heterosexual Men | Anxiety | 7.7% [7.3%, 8.0%] | 13.4% [13.1%, 13.7%] | 14.1% [13.7%, 14.6%] |
| Black/AA Heterosexual Men | Depression | 24.9% [24.3%, 25.5%] | 33.2% [32.6%, 33.7%] | 37.2% [35.9%, 39.3%] |
| Black/AA Heterosexual Men | CKD | 10.5% [10.1%, 10.9%] | 23.7% [23.2%, 24.2%] | 44.9% [43.1%, 47.9%] |
| Black/AA Heterosexual Men | Dyslipidemia | 30.3% [29.6%, 31.0%] | 50.2% [49.6%, 50.9%] | 69.0% [67.3%, 71.4%] |
| Black/AA Heterosexual Men | Diabetes | 13.9% [13.4%, 14.4%] | 20.5% [20.1%, 21.0%] | 34.0% [32.9%, 35.7%] |
| Black/AA Heterosexual Men | Hypertension | 46.2% [45.5%, 46.9%] | 50.3% [49.7%, 50.8%] | 46.7% [45.7%, 48.1%] |
| Black/AA Heterosexual Men | Cancer | 8.4% [8.0%, 8.8%] | 11.1% [10.7%, 11.4%] | 13.1% [12.3%, 14.1%] |
| Black/AA Heterosexual Men | ESLD | 1.2% [1.1%, 1.4%] | 1.0% [0.9%, 1.1%] | 0.7% [0.6%, 0.8%] |
| Black/AA Heterosexual Men | MI | 1.4% [1.2%, 1.6%] | 2.6% [2.5%, 2.8%] | 6.5% [5.8%, 7.3%] |
| Black/AA Heterosexual Men | ≥1 Ment. | 30.2% [29.5%, 30.8%] | 39.2% [38.7%, 39.7%] | 42.5% [41.4%, 44.5%] |
| Black/AA Heterosexual Men | ≥2 Phys. | 30.7% [30.1%, 31.4%] | 48.7% [48.1%, 49.6%] | 65.0% [63.0%, 68.2%] |
| Black/AA Heterosexual Men | ≥2 Any | 44.2% [43.5%, 44.9%] | 62.4% [61.7%, 63.1%] | 75.4% [73.4%, 78.5%] |
| Black/AA Heterosexual Men | ≥1 Ment. & ≥2 Phys. | 9.3% [8.9%, 9.6%] | 19.7% [19.2%, 20.3%] | 28.5% [27.2%, 30.7%] |
| Hispanic Heterosexual Men | Anxiety | 15.2% [14.4%, 16.1%] | 25.6% [24.7%, 26.4%] | 37.8% [36.7%, 39.6%] |
| Hispanic Heterosexual Men | Depression | 35.0% [34.0%, 36.0%] | 45.0% [44.1%, 46.1%] | 41.7% [39.5%, 45.1%] |
| Hispanic Heterosexual Men | CKD | 9.8% [9.0%, 10.4%] | 21.5% [20.7%, 22.4%] | 42.3% [40.4%, 45.0%] |
| Hispanic Heterosexual Men | Dyslipidemia | 31.3% [30.3%, 32.4%] | 46.8% [45.8%, 48.2%] | 64.9% [63.2%, 67.6%] |
| Hispanic Heterosexual Men | Diabetes | 10.5% [9.7%, 11.2%] | 18.4% [17.7%, 19.2%] | 32.0% [30.8%, 33.8%] |
| Hispanic Heterosexual Men | Hypertension | 25.4% [24.4%, 26.4%] | 35.0% [34.1%, 36.0%] | 39.3% [37.4%, 41.8%] |
| Hispanic Heterosexual Men | Cancer | 8.5% [7.8%, 9.1%] | 11.4% [10.8%, 12.1%] | 13.5% [12.6%, 14.9%] |
| Hispanic Heterosexual Men | ESLD | 1.2% [0.9%, 1.4%] | 0.9% [0.7%, 1.1%] | 0.6% [0.5%, 0.8%] |
| Hispanic Heterosexual Men | MI | 1.4% [1.2%, 1.7%] | 2.5% [2.2%, 2.8%] | 6.0% [5.3%, 7.2%] |
| Hispanic Heterosexual Men | ≥1 Ment. | 42.0% [41.0%, 43.0%] | 49.5% [48.7%, 50.5%] | 52.3% [50.5%, 55.0%] |
| Hispanic Heterosexual Men | ≥2 Phys. | 21.3% [20.5%, 22.3%] | 40.0% [38.9%, 41.1%] | 59.4% [56.9%, 62.6%] |
| Hispanic Heterosexual Men | ≥2 Any | 41.5% [40.5%, 42.6%] | 61.3% [60.2%, 62.3%] | 75.7% [73.3%, 79.3%] |
| Hispanic Heterosexual Men | ≥1 Ment. & ≥2 Phys. | 9.1% [8.5%, 9.7%] | 20.9% [20.1%, 21.9%] | 32.2% [30.4%, 35.0%] |
| White Heterosexual Women | Anxiety | 32.4% [31.5%, 33.0%] | 49.9% [49.1%, 50.7%] | 59.9% [59.0%, 61.3%] |
| White Heterosexual Women | Depression | 43.8% [43.0%, 44.5%] | 56.5% [55.8%, 57.4%] | 62.2% [60.9%, 64.2%] |
| White Heterosexual Women | CKD | 11.0% [10.5%, 11.5%] | 29.8% [29.0%, 30.6%] | 51.7% [49.4%, 55.2%] |
| White Heterosexual Women | Dyslipidemia | 42.7% [41.9%, 43.4%] | 49.0% [48.1%, 49.9%] | 46.7% [44.7%, 49.2%] |
| White Heterosexual Women | Diabetes | 11.1% [10.6%, 11.6%] | 24.0% [23.3%, 24.6%] | 39.2% [38.1%, 40.8%] |
| White Heterosexual Women | Hypertension | 30.0% [29.3%, 30.8%] | 35.4% [34.7%, 36.1%] | 32.1% [31.2%, 33.6%] |
| White Heterosexual Women | Cancer | 6.5% [6.1%, 6.9%] | 7.9% [7.5%, 8.2%] | 6.8% [6.4%, 7.2%] |
| White Heterosexual Women | ESLD | 1.2% [1.0%, 1.3%] | 1.1% [0.9%, 1.2%] | 1.1% [1.0%, 1.2%] |
| White Heterosexual Women | MI | 1.5% [1.3%, 1.7%] | 3.7% [3.5%, 3.9%] | 8.7% [7.9%, 9.7%] |
| White Heterosexual Women | ≥1 Ment. | 60.5% [59.8%, 61.4%] | 71.0% [70.3%, 71.7%] | 76.6% [75.6%, 78.0%] |
| White Heterosexual Women | ≥2 Phys. | 27.6% [26.9%, 28.3%] | 45.7% [44.8%, 46.7%] | 55.1% [53.0%, 58.4%] |
| White Heterosexual Women | ≥2 Any | 57.7% [56.9%, 58.5%] | 73.8% [73.0%, 74.7%] | 80.6% [79.1%, 83.1%] |
| White Heterosexual Women | ≥1 Ment. & ≥2 Phys. | 17.0% [16.4%, 17.6%] | 34.3% [33.4%, 35.0%] | 44.5% [42.6%, 47.4%] |
| Black/AA Heterosexual Women | Anxiety | 16.4% [16.0%, 16.6%] | 30.5% [30.2%, 30.9%] | 48.6% [47.9%, 49.5%] |
| Black/AA Heterosexual Women | Depression | 41.3% [41.0%, 41.8%] | 49.7% [49.3%, 50.1%] | 48.0% [47.2%, 49.1%] |
| Black/AA Heterosexual Women | CKD | 10.8% [10.6%, 11.1%] | 17.5% [17.2%, 17.8%] | 21.7% [21.0%, 22.4%] |
| Black/AA Heterosexual Women | Dyslipidemia | 31.9% [31.5%, 32.4%] | 50.5% [50.1%, 51.0%] | 71.2% [69.8%, 72.8%] |
| Black/AA Heterosexual Women | Diabetes | 14.7% [14.4%, 15.0%] | 27.3% [27.0%, 27.6%] | 45.5% [44.7%, 46.4%] |
| Black/AA Heterosexual Women | Hypertension | 45.0% [44.5%, 45.3%] | 50.1% [49.7%, 50.4%] | 48.5% [47.8%, 49.2%] |
| Black/AA Heterosexual Women | Cancer | 6.6% [6.4%, 6.8%] | 8.4% [8.3%, 8.6%] | 7.3% [7.1%, 7.6%] |
| Black/AA Heterosexual Women | ESLD | 1.2% [1.1%, 1.3%] | 0.9% [0.9%, 1.0%] | 1.0% [0.9%, 1.0%] |
| Black/AA Heterosexual Women | MI | 1.4% [1.3%, 1.5%] | 3.2% [3.1%, 3.3%] | 10.5% [9.9%, 11.1%] |
| Black/AA Heterosexual Women | ≥1 Ment. | 50.4% [50.0%, 50.9%] | 60.8% [60.4%, 61.1%] | 66.3% [65.6%, 67.3%] |
| Black/AA Heterosexual Women | ≥2 Phys. | 30.6% [30.2%, 30.9%] | 47.9% [47.5%, 48.3%] | 63.2% [61.9%, 64.6%] |
| Black/AA Heterosexual Women | ≥2 Any | 53.9% [53.4%, 54.3%] | 70.4% [70.1%, 70.9%] | 81.7% [80.6%, 83.0%] |
| Black/AA Heterosexual Women | ≥1 Ment. & ≥2 Phys. | 15.6% [15.3%, 15.9%] | 30.5% [30.1%, 30.9%] | 43.4% [42.1%, 44.7%] |
| Hispanic Heterosexual Women | Anxiety | 24.9% [24.3%, 25.6%] | 49.2% [48.5%, 49.9%] | 78.4% [77.9%, 79.0%] |
| Hispanic Heterosexual Women | Depression | 46.2% [45.5%, 46.9%] | 58.7% [58.1%, 59.5%] | 60.4% [59.3%, 62.3%] |
| Hispanic Heterosexual Women | CKD | 10.6% [10.1%, 11.1%] | 26.4% [25.8%, 27.1%] | 48.5% [46.0%, 52.3%] |
| Hispanic Heterosexual Women | Dyslipidemia | 35.1% [34.3%, 36.0%] | 47.5% [46.6%, 48.3%] | 45.4% [43.3%, 48.4%] |
| Hispanic Heterosexual Women | Diabetes | 11.0% [10.6%, 11.5%] | 23.6% [23.0%, 24.1%] | 40.9% [40.0%, 42.1%] |
| Hispanic Heterosexual Women | Hypertension | 29.8% [29.0%, 30.4%] | 34.9% [34.4%, 35.6%] | 32.0% [30.9%, 33.7%] |
| Hispanic Heterosexual Women | Cancer | 6.6% [6.3%, 7.0%] | 8.7% [8.3%, 9.0%] | 7.3% [7.0%, 7.9%] |
| Hispanic Heterosexual Women | ESLD | 1.1% [1.0%, 1.3%] | 0.9% [0.8%, 1.1%] | 0.9% [0.8%, 1.1%] |
| Hispanic Heterosexual Women | MI | 1.5% [1.3%, 1.6%] | 3.1% [2.9%, 3.3%] | 7.7% [7.0%, 8.8%] |
| Hispanic Heterosexual Women | ≥1 Ment. | 58.5% [57.7%, 59.2%] | 72.2% [71.6%, 73.0%] | 85.7% [85.3%, 86.1%] |
| Hispanic Heterosexual Women | ≥2 Phys. | 24.4% [23.7%, 25.0%] | 43.7% [42.8%, 44.6%] | 54.1% [51.7%, 57.6%] |
| Hispanic Heterosexual Women | ≥2 Any | 52.7% [51.9%, 53.5%] | 74.1% [73.5%, 75.0%] | 85.9% [84.7%, 87.8%] |
| Hispanic Heterosexual Women | ≥1 Ment. & ≥2 Phys. | 14.5% [14.1%, 15.1%] | 32.7% [32.0%, 33.5%] | 46.5% [44.5%, 49.4%] |

Footnotes:

AA = African American

PEARL = ProjEcting Age, multimoRbidity, and poLypharmacy in Adults with HIV

PWH = people with HIV

MSM = men who have sex with men

MWID = men who had injection drug use as their HIV acquisition risk factor

WWID = men who had injection drug use as their HIV acquisition risk factor

≥1 Ment. = Anxiety or depression or both (i.e., ≥1 of the mental comorbidities included)

≥2 Phys. = physical multimorbidity, defined as ≥2 physical comorbidities

≥2 Any = physical or mental multimorbidity, defined as ≥2 physical or mental comorbidities

≥1 Ment. & 2 Phys. = mental comorbidity and physical multimorbidity, defined as ≥1 mental comorbidity and ≥2 physical comorbidities

CKD = Stage ≥3 chronic kidney disease

ESLD = End-stage liver disease

MI = myocardial infarction

^a^Although these estimates are all PEARL forecasts, 2010 was during the calibration period (where observed NA-ACCORD data were available to inform the estimates) and 2020 and 2030 were forecast periods (without observed NA-ACCORD data).

The 95% interquartile range is estimated as the 2.5% and 97.5% range of results from running the simulation 200 times (also called the 95% uncertainty range).
