# Supplementary material for: The forecasted prevalence of comorbidities and multimorbidity in people with HIV in the United States through the year 2030: A modeling study
Source: PLoS Med. 2024 Jan 12;21(1):e1004325. doi: 10.1371/journal.pmed.1004325 (PMC10833859; doi:10.1371/journal.pmed.1004325)

**New HIV diagnosis:** To predict the number of new people linking to HIV care and starting ART in a given year, we begin with data on the number of new HIV diagnoses per year as estimated by the CDC’s Medical Monitoring Project (MMP) as shown in **S8 Table**. Dates before 2016 came from Table 1 of the [2015 HIV Surveillance Report](https://www.cdc.gov/hiv/pdf/library/reports/surveillance/cdc-hiv-surveillance-report-2015-vol-27.pdf), while data 2016 and after came from Table 1 of the [2018 HIV Surveillance Report](https://www.cdc.gov/hiv/pdf/library/reports/surveillance/cdc-hiv-surveillance-report-2018-updated-vol-31.pdf). See <https://pearlhivmodel.org/method_details.html> for more details on methodological details.

**S8 Table:** Number of new HIV diagnoses by year and subgroup


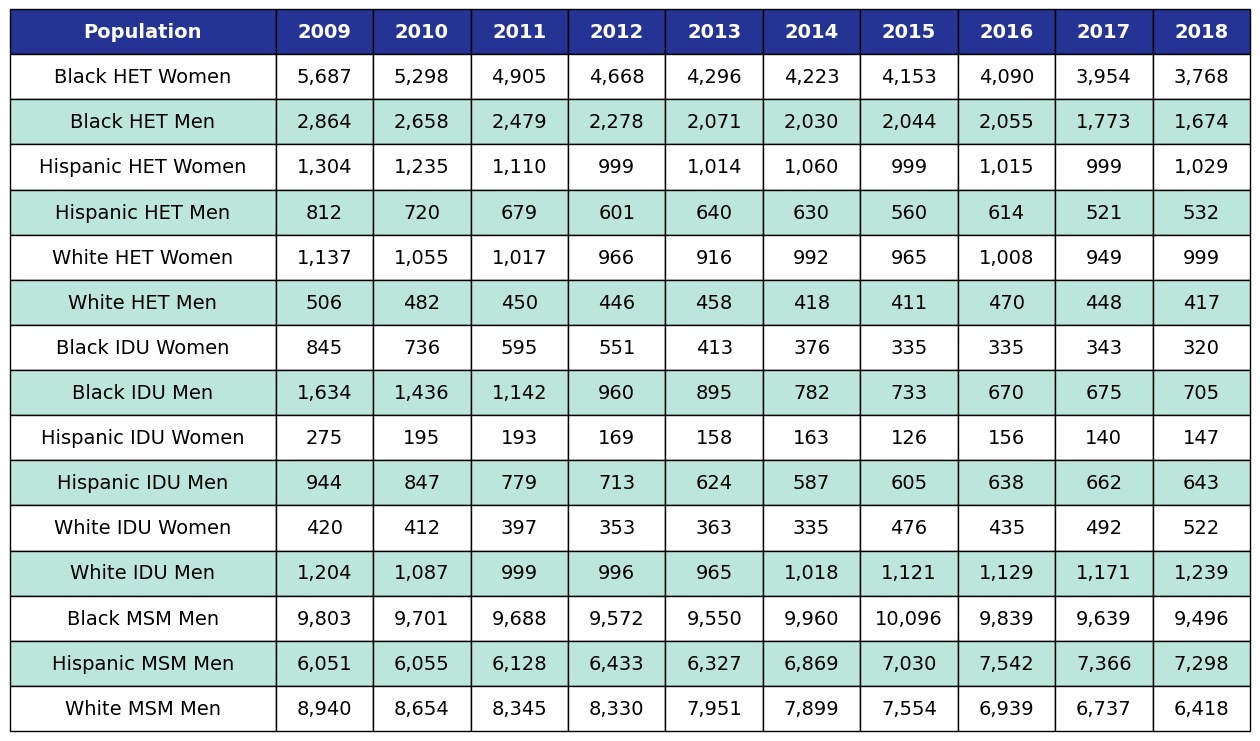

Supplement: S8 Table — (DOCX) [file pmed.1004325.s015.docx]
