# Supplementary material for: The forecasted prevalence of comorbidities and multimorbidity in people with HIV in the United States through the year 2030: A modeling study
Source: PLoS Med. 2024 Jan 12;21(1):e1004325. doi: 10.1371/journal.pmed.1004325 (PMC10833859; doi:10.1371/journal.pmed.1004325)
Supplement: Attachment — Submitted filename: althoff.pdf [file pmed.1004325.s016.pdf]

This paper describes a series of projections of comorbidity in people living with HIV in the USA.

I was asked for a statistical report and I interpret that to include all aspects of the design and conduct of the study. The version I received did not have page numbers. My numbering has the abstract starting on page 6

Points of detail

Page 6 The results here are presented as facts in the past ('increased', 'decreased') rather than projections ('would increase') which seems potentially misleading in the abstract. In the main text it is fine.

Page 10 I am afraid I did not understand the paragraph on validation and Figure S2 did not enlighten me. I suspect a few more words would clarify it.

Page 11 The sentence starting 'The number of Blck/AA MWID and WWID' would make more sense to me if 'but' was replaced by 'and' as the two clauses do not seem to contradict one another. Have I failed to understand the meaning here? I assume decreased is a typo for decrease.

Page 15 Some of the speculation about limitations seems to me to go beyond what the data shows. It would be equally plausible that the selective mortality from COVID would have removed the people with most morbidities resulting in fewer surviving to 2030.

Page 15 I am not an HIV researcher but I wonder whether the widespread adoption of pre–exposure prophylaxis should be mentioned too. Obviously it cannot yet be in the model since its long–term effects are as yet unknown.

Table 1 What are the numbers in parentheses or brackets?

Figure 3 The advertised shading did not appear in the version I received although I suspect it would make the plots unreadable if it did. The different comorbities are quite hard to distinguish here. Different line type might help to clear up which is which. I had to blow up the PDF to 200% on my ancient monitor to even see there were different symbols. For some reason Figures S3a–o do have shading and more distinguishable lines.

Figure 5 It looks Cam did sort the plot but then forgot to delete the comment.

Summary

Mostly for clarification. I will leave it to the clinical reviewers to comment on the plausibility of the projections.

Michael Dewey
