# Supplementary material for: The forecasted prevalence of comorbidities and multimorbidity in people with HIV in the United States through the year 2030: A modeling study
Source: PLoS Med. 2024 Jan 12;21(1):e1004325. doi: 10.1371/journal.pmed.1004325 (PMC10833859; doi:10.1371/journal.pmed.1004325)
Supplement: Attachment — Submitted filename: 2023_0822 response to reviewers.docx [file pmed.1004325.s017.docx]

**Message from the authors**

PEARL is a dynamic model; we are always improving the model. Since our Jan 5, 20223 submission to *PLoS Medicine*, we have made further debugged the model and re-running calibration checks and **the entire manuscript and supplement have been updated**. Two changes to PEARL that impacted our presented results include:

- The estimate glomerular filtration rate (eGFR) was updated to exclude race, as is now the recommended standard. The older approach over-estimated the eGFR measurement, which under-estimated the severity of chronic kidney disease among Black individuals. CKD estimates from the PEARL model *increased* *slightly* in Black subgroups.
- Expansion of the definition of dyslipidemia from elevated total cholesterol to include *a statin prescription*, which has *increased* the forecasted incidence and prevalence of dyslipidemia. Our clinical investigators believed the inclusion of statin prescriptions was important for an accurate estimate of dyslipidemia burden.

These definitions, and the definitions fall comorbidities and risk factors are located in **Supplement Table S1**. These changes are also documented in the Methods section of our website (<https://pearlhivmodel.org/method_details.html>). Additionally, we have posted the code for the PEARL model that produced the results presented in the manuscript and added this link to the second paragraph of the Methods section (<https://github.com/PearlHivModelingTeam/comorbidityPaper>).

**Comments from the reviewers:**

**Reviewer #1:** Thanks for sharing your manuscript entitled: "The projected prevalence of comorbidities and multimorbidity in people with HIV in the United States through the year 2030". I really enjoy reading your work, I think this kind of modeling efforts are very important to define the potential burden of diseases in a population, and to plan the care and needs of HIV people further than their HIV traditional care, even more if they are focused on groups of specific and different risk.

We appreciate the reviewer’s enthusiasm and understanding of the population-level focus of the modeling output.

I have only minor comments related to the description of the model. I couldn´t understand in the manuscript, how new people incorporates in the follow-up, and neither I could open the link which authors mention that details are (Please double-check the link is really working). I would like to see some lines into the manuscript to understand how is the entrance in the model by group of risk year by year. If this is explained in previous papers, I think readers interested could be informed.

We are sorry about the difficulty that the reviewer experienced with accessing our website. We double checked the link to the methodological details of the model, it works when we click on it (<https://pearlhivmodel.org/method_details.html>). This page provides the methodological details on the “population initiation ART from 2010-2030”, including the procedures used to estimate the annual population sizes, age distribution and CD4 count at ART initiation.

We have clarified how the agents with HIV enter the model when they initiate ART (specifically, in the calendar year of ART initiation from 2010 to 2030) in the second paragraph of the Methods section. To further address the reviewer’s comment, we have added Section S2 to the Supplement, which details the approach to estimating the population size of people with HIV initiating ART.

I would also like to know why ART regimen was not incorporated in some of the comorbidities´ incidence estimations? Is not a current potential association of ART with some of the variables involved, such as changes in BMI?

We agree with the reviewer about the potential impact of ART regimen on comorbidity outcomes. However, we did not have enough data within all 15 subgroups to explicitly integrate ART regimen into the current version of the PEARL model. While the mathematical functions predicting comorbidity incidence do not explicitly include ART regimens, they are estimated among people who initiated ART in the NA-ACCORD from 2010-2017, and as such, potential associations are implicitly impacting the frequency of outcomes. In response to reviewer’s comment, we have added this limitation to the Discussion:

*“ART regimen type is not explicitly contained in the PEARL model, but the impact of various ART regimens on comorbidity incidence is implicitly contained in the mathematical functions from observed NA-ACCORD data.”*

Each risk group has their own probabilities of ART disengagement and re-engagement?

Correct, each subgroup has their own predicted probabilities of disengagement *(see Table 9 at* [*https://pearlhivmodel.org/method_details.html*](https://pearlhivmodel.org/method_details.html) *for more details*).

The sample size observed to re-engage (after disengagement) in the NA-ACCORD was insufficient in many of the 15 subgroups; therefore, we aggregated all sub-populations and assessed at the number of years spent out of care, estimated between 1 to 7 years. The probability of spending a certain number of years out of care was estimated via a normalized Poisson distribution such that the probability of staying disengaged for more than 7 years was zero (*see Figure 7 at* [*https://pearlhivmodel.org/method_details.html*](https://pearlhivmodel.org/method_details.html) *for more details*). Upon disengagement, we apply this distribution to generate the number of years that a simulated person will spend off ART before reengaging back with treatment.

In response to this comment, we have added the following description to the second paragraph of the Methods section:

*“All mathematical functions and parameters in PEARL are estimated separately for each of the 15 subgroups, or collapsed if a priori specifications of minimum sample size or number of events were not met (collapsing race/ethnicity groups first, followed by sex and then HIV-acquisition risk until the minimum is met).”*

Additionally, I´m curious about if this model could be used to predict the comorbidities by group by US state, Is this possible? Or the model calibration and other details to program it don´t allow it? I think this kind a state stratification could be useful for local policy makers and that other social factors associated with local resources, education level or income could be incorporated in the estimations. Is this an issue that authors could comment/discuss into their work? New potential policies to have a better care for some of the populations: those avoiding racism in black and Hispanic population, or those addressed on drug users, could have any impact on your estimations? How big could be this impact?

The PEARL model is well suited to address potential questions concerning the projected prevalence of comorbidities and multimorbidity among 15-subgroups of PLWH at a “national-level”, but as of now, it does not project outcomes by geography.

We agree with the reviewer on the importance of expanding our projections to local settings. Currently, we are conversing with Dr. James Tesoriero, Director of the Center for Program Development, Implementation, Research, and Evaluation at the New York State Department of Health AIDS Institute, and his team to review modeling assumptions needing further revisions to make simulations outputs applicable to the New York state. This will likely include a wide array of assumptions and parameters specific to population size, demographic characteristic, and ART initiation among people with HIV in the state of New York within the 15 subgroups. Potential assumptions regarding individual’s socioeconomic status can influence several parameters in our model (e.g., lower rates of engagement in care among certain vulnerable populations). Other efforts to prevent new infections (via PrEP or community outreach program for different groups) can be further impact the future projections of HIV incidence, and subsequently the population size and age of those initiating ART.

In response to reviewer’s comment, we have added this limitation to the Discussion section:

*“PEARL model forecasts are currently at the national-level, and we are examining the availability of data to forecast at the state-level.”*

I think authors could also mention some advantages of the agent-based models compared to other type of models in the discussion.

We have added a sentence to the last paragraph in the Discussion:

*“Agent-based simulation models are recommended for chronic disease forecasting because they capture the complex interactions among individual-level risk factors that determine the risk of comorbidities and mortality as well as feedback loops needed for disengagement and re-engagement in care.”*

**Reviewer #2:** This very interesting study aims to predict the rates of co-morbidities in different groups of US PLHIV.

Abstract and title are confusing because they do not indicate that this is a modeling study.

We have added “*A modeling study”* to the end of the title.

In the abstract: “***Design:*** *Agent-based simulation model”*

The Introduction discusses the criticality of SDoH, but as-written, it is not clear which SDoH are included in the model. Please incorporate these into Figure 1. For broader introduction, please also define SDoH and list which are the most important determinants for the populations modeled.

We have added the following to the second paragraph of the Introduction:

*“Key SDoH include race, ethnicity, sex, and HIV acquisition risk group.”*

Social determinants of health are included in the model by building the model as the sum of the experiences of 15 subgroups defined by the key SDoH. We have added the following to the first paragraph of the Methods section to clarify:

*“The ProjEcting Age, multimoRbidity, and poLypharmacy (PEARL) model is an agent-based computer simulation model of 15 subgroups of PWH who have initiated ART in the US, including those who disengage from HIV care (****Figure 1****). The 15 subgroups are defined at the intersections of key social determinants of health: (1 & 2) men and women with history of injection drug use as an HIV acquisition risk factor, including those who have injection drug use and any additional HIV acquisition risk category specified (MWID and WWID respectively); (3) men who have sex with men (MSM); and (4 & 5) heterosexual men and women. These five groups were further stratified into non-Hispanic White, non-Hispanic Black/AA, and Hispanic.”*

The last sentence of paragraph 2 of the Intro is difficult to parse: "Multimorbidity is a function of the comorbidities included in the definition and estimates among PWH in the US have a wide range." There seem to be two different points being made here. Make them separately and in sufficient detail, i.e., one sentence explaining the definition of multimorbidity, and the other stating the ranges on multimorbidity estimates from prior studies.

The definition of multimorbidity is in the second to last sentence of the second paragraph in the Introduction:

*“The disproportionate burdens of comorbidities and subsequent multimorbidity (i.e., ≥2 comorbidities not including HIV) pose persistent challenges in ensuring adequate healthcare for people with HIV.”*

We have modified the last sentence of the paragraph to clarify that the wide-ranging estimates of multimorbidity in PWH is due in part to the differences in comorbidities include in the definition of multimorbidity across studies:

*“Estimates among PWH in the US have ranged from 8.2% in 2000 to 65% in 2010-2011; the variability is in part due to modification to the definition of multimorbidity and differences in comorbidities included.^24-26^”*

Is it necessary to subdivide factors correlated with but not caused by HIV (such as smoking an injecting drugs), versus ones caused by HIV (cancer and diseases associated with chronic immune activation)?

Both correlated factors and causal factors related to the comorbidity outcomes are necessary for accurate probability estimates; sub-dividing factors correlated vs. caused (in part) by HIV within the model is not necessary.

The model appears to really be fifteen different models for each of 5 HIV transmission group (MSM, MWID, WWID, heterosexual men, heterosexual women) X 3 racial groups (white, black/AA, Hispanic). It does not consider that individuals can be multiracial or have overlapping risks. Does it include transmission between racial groups and risk groups? It is important to clarify these limitations and think about whether the results are still valid subject to these limitations.

The reviewer’s understanding is correct. PEARL includes 15 subgroups of sex, race and HIV acquisition risk, but does not consider individuals who may be multiracial or those with overlapping risks.

We do not simulate HIV transmissions within the PEARL model. The population size of persons starting ART in each calendar year is estimated from CDC HIV Surveillance data on number of new HIV diagnosis and the rate of linkage to care and ART initiation (as presented in *Supplement Section 2*). These limitations have been discussed in previous publications of the PEARL model, and we have now added them to the last paragraph of the Discussion section:

*“PEARL includes 15 subgroups defined by sex, HIV acquisition risk groups and race/ethnicity, however, it does not represent individuals who are multiracial or those with overlapping risks.”*

*“The PEARL model does not include HIV transmission. The forecasted annual number of new ART initiators relies on CDC's reported HIV diagnoses and linkage to care during the calibration period and does not consider changes in HIV transmission dynamics after the calibration period, including changes due to HIV prevention efforts (e.g., pre-exposure prophylaxis or PrEP) or during the COVID-19 pandemic (e.g., 2020-2021).”*

To the reviewer’s point, we are interested to understand how changes in HIV incidence impact the forecasted age distributions, comorbidity prevalence, and multimorbidity prevalence over the next decade. We are currently working with a modeling team to link their forecasted number of new HIV diagnosis as PEARL input parameters. This would allow us to toggle between two sources of estimated new HIV diagnosis: the observed CDC HIV surveillance data and model-generated data from various scenarios involving *Ending the HIV Epidemic* efforts. This work is ongoing.

The model appears to only include people from the time they first initiate ART. How is the rate of people newly initiating ART (i.e., new individuals entering the model) determined? Presumably this depends on future ART coverage as well as future rates of new HIV infections which give rise to people never before on ART - and HIV infections in turn depend on the populations with unsuppressed HIV viral load participating in different forms of behavioral risk - how is all of this handled?

The approach to estimating the number of new ART-initiators relies on CDC HIV Surveillance data of new HIV diagnoses, CDC estimates of receipt of HIV care 1-to-3 months after HIV diagnosis, and HIV treatment guideline estimates that estimates 97% of people with HIV are eligible to initiate ART at presentation for HIV care. The PEARL model includes the assumption that trends in ART initiation from 2010-2017 persist to 2030; the potential impact of emerging care technologies like oral or long-acting PrEP and ART, or potential changes in HIV transmission dynamics due to COVID-19 is unaccounted for.

We added a new section to the Supplement (Section S2) to detail the methodology for estimating the number of new ART initiators entering the PEARL model, and have expanded the Discussion on limitations of the PEARL model due to lack a transmission engine:

*“The PEARL model does not include HIV transmission. The forecasted annual number of new ART initiators relies on CDC's reported HIV diagnoses and linkage to care during the calibration period and does not consider changes in HIV transmission dynamics after the calibration period, including changes due to HIV prevention efforts (e.g., pre-exposure prophylaxis or PrEP) or during the COVID-19 pandemic (e.g., 2020-2021). Similarly, the calibration of mathematical functions in PEARL is based on NA-ACCORD data available during the calibration period. The PEARL model assumes that the trends observed during the calibration period will continue and does not account for influences occurring outside the calibration period (e.g., the impact of emerging care technologies like long-acting ART or the impact of COVID-19 on mortality); such factors influencing comorbidities incidence can be incorporated into PEARL by extending the calibration period when there are more current data available within the 15 subgroups.”*

Drug use and risk of drug overdose is a large driver of morbidity and mortality, in PWID, constituting six of the fifteen populations being modeled. How can the model accurately capture life expectancy without accounting for drug use morbidities and risk of overdose?

The reviewer raises an astute point that we have examined closely in another study under review that dives deeper into how the subgroup specific mortality rates used in PEARL are essential for predicting the future age distributions and size of the population aging with HIV through the year 2030.

Because mortality rates are subgroup specific, the higher mortality rate of people who use drugs is reflected in the mortality rates used to create the experience of the PEARL agents who are classified as people who use drugs. We have re-written the second paragraph of the Methods section to clarify this point:

*“The agents are followed after ART initiation and observed to experience aging, disengagement and re-engagement in care, changing CD4 counts, risk factors, comorbidities, and mortality via mathematical functions and parameters from the NA-ACCORD and CDC data. The mathematical functions forecast the agents’ experience in the future after observed data end. All mathematical functions and parameters in PEARL are estimated separately for each of the 15 subgroups, or collapsed if a priori specifications of minimum sample size or number of events were not met (collapsing race/ethnicity groups first, followed by sex and then HIV-acquisition risk until the minimum is met).”*

Additionally, the engaged and dis-engaged-specific mortality for the subgroups is in *Supplemental Table S3*, which we refer to in the “*Comorbidities parameterizations*” sub-section of the Methods:

*“Finally, mortality was estimated for simulated persons as a function of individual-level attributes, existing risk factors, and present comorbidities, and was estimated separately for those (1) engaged and (2) disengaged from care (≥2 years without CD4 or HIV RNA measurement) and in each of the 15 subgroups using observed NA-ACCORD data from 2009-2017 (Supplement Table S3).”*

The calibration plots in Supplementary Appendix are an excellent component of this study and very well-presented. There don't appear to be any incidence plots where the model fails to match the data, but there are a number of prevalence plots where the model fails to match the data. How can this be? Is it a problem with the initial conditions?

Thank you for reviewing the Supplementary Appendix. The prevalence of certain comorbidities that do not fit tightly to the observed NA-ACCORD data are noted with orange shading in *Supplemental Figure S2*. This less than desirable fit occurs among the Hispanic subgroups, white heterosexual men, and Black/AA and White women who injected drugs. These groups have smaller observed sample sizes in NA-ACCORD and were combined with other subgroups resulting in differences between the NA-ACCORD observed estimates and PEARL estimates using the *a priori* collapsing strategy of collapsing first by race/ethnicity and second by gender; we have not needed to collapse HIV transmission risk groups. This information is included in the title and footnotes of *Supplemental Figure S2.*

The model estimates the incidence of comorbidities (e.g., depression) as a function of calendar year, age, CD4 count at ART initiation, and time since ART initiation. These are shown as betas from a regression. Does that assume that incidence of comorbidities must always be a linear function of age? Many comorbidities have an age of maximum or minimum incidence (hump-shaped of bowl-shaped function of age) and many others have low incidence until older age (aging-related conditions like HTN, diabetes…) How are these common age patterns taken into account?

The reviewer is correct that age is an independent variable in the model estimating the probability of comorbidity incidence; the relationship is assumed to be linear (i.e., the change in probability per unit increase in age). We agree with the reviewer that the relationship between age and comorbidities are not always necessarily linear. When selecting the mathematical functions estimating the probability of the incident comorbidity, we modified the parameterization of age (using splines and other approaches) and found no appreciable benefit in the fit of the function. Conversely, the functions estimating the probabilities of HIV mortality while on ART and while disengaged from care that included cubic splines for age were superior in fit and were selected for PEARL. As the data from the NA-ACCORD more substantially reflects these nonlinear relationships with age, the updates to the PEARL model functions will incorporate the best fitting functions.

Why did Black WWID have the oldest median age in 2030? One would think their life expectancy would be shorter than heterosexual women due to many health risks and comorbidities. Could this possibly be an error in the model? If so, this could explain why WWID are predicted to have so many co-morbidities compared to other groups (they are simply older and risks of many chronic health conditions grow with age).

Survivor bias is inherent in PWH who reach older ages and may be strongest among those of older age in the subgroups that have the greatest threats to mortality. Black women classified with injection drug use as their HIV acquisition risk who survive to older ages are likely to have survivor bias and are more likely to accumulate age-related comorbidities with increasing age (as the reviewer suggests). Given that Black WWID agents in PEARL are populated with CDC HIV Surveillance estimates of new diagnoses and NA-ACCORD estimates specific to this subgroup, and experience mortality rates estimated among those who injected drugs as their HIV acquisition group, we believe these estimates are accurate. The same pattern was presented in the prior PEARL study of the forecasted age distributions of subgroups (Althoff KN et al., AIDS, 2022. doi: 10.1097/QAD.0000000000003128). We have included this point in the Discussion:

*“Subgroup stratification is essential due to the heterogeneity of PWH. For example, Black/AA WWID are forecasted to have the oldest age distribution in 2030, which is likely a function of gender differences in life expectancy, a survivor effect of these women who had injected drugs during the period they acquired HIV but could be in recovery from substance use disorder, and the national reduction in new HIV infections (which typically happen in younger adults) among people who use injection drugs.^47,48^”*

Could the authors provide calibration graphs showing how their modeled population age structure compares to observed age structure of the 15 groups in different years? I wonder if discrepancies there are contributing to the unintuitive median age findings in the model outputs.

The comparison of the age distributions estimated by PEARL and observed in the NA-ACCORD were presented for the years of 2010-2017 in the Supplement of Althoff KN et al., *AIDS*, 2022 (doi: 10.1097/QAD.00000000000031280) and are on the following pages:

Table S 1: Comparing the age distributions of ART-users in PEARL to the observed data from NA-ACCORD, from 2010–2017 (Simulation validation “out-of-sample” approach). Values represent the difference in the age distribution in each subgroup [NA-ACCORD estimate - PEARL projection]. A threshold of 5 percentage points (>5% or <-5%) is used to detect significant differences (highlighted in blue).

|  | **2010** | **2011** | **2012** | **2013** | **2014** | **2015** | **2016** | **2017** |
| --- | --- | --- | --- | --- | --- | --- | --- | --- |
| **White MSM** |  |  |  |  |  |  |  |  |
|  |  |  |  |  |  |  |  |  |
| <30 years | 0.7% | 0.8% | 0.6% | 0.8% | 0.9% | 0.6% | 0.7% | 0.8% |
|  | [4.9% vs. 4.2%] | [5.4% vs. 4.6%] | [5.7% vs. 5.1%] | [6.1% vs. 5.4%] | [6.4% vs. 5.5%] | [6.1% vs. 5.5%] | [6.1% vs. 5.4%] | [6.1% vs. 5.3%] |
| 30-39 years | -1.2% | -0.4% | 0.4% | 0.8% | 1.1% | 1.2% | 1.4% | 2.6% |
|  | [14.8% vs. 16.0%] | [14.2% vs. 14.7%] | [14.3% vs. 13.8%] | [14.1% vs. 13.3%] | [14.1% vs. 13.0%] | [14.0% vs. 12.8%] | [14.2% vs. 12.8%] | [15.5% vs. 13.0%] |
| 40-49 years | -0.9% | -0.7% | -1.5% | -1.8% | -2.5% | -2.4% | -2.1% | -1.4% |
|  | [41.0% vs. 41.9%] | [38.4% vs. 39.1%] | [34.7% vs. 36.2%] | [31.4% vs. 33.3%] | [28.0% vs. 30.5%] | [25.6% vs. 28.0%] | [23.4% vs. 25.6%] | [22.1% vs. 23.5%] |
| 50-59 years | 1.2% | 0.6% | 0.6% | 0.3% | 0.3% | 0.5% | -0.7% | -0.2% |
|  | [29.3% vs. 28.2%] | [31.1% vs. 30.6%] | [33.2% vs. 32.6%] | [34.7% vs. 34.4%] | [36.1% vs. 35.9%] | [37.5% vs. 37.0%] | [37.0% vs. 37.7%] | [37.7% vs. 37.9%] |
| 60-69 years | -0.1% | -0.5% | -0.3% | 0.0% | 0.3% | 0.2% | 0.7% | -1.1% |
|  | [8.4% vs. 8.5%] | [9.1% vs. 9.6%] | [10.2% vs. 10.5%] | [11.5% vs. 11.6%] | [12.9% vs. 12.6%] | [14.0% vs. 13.8%] | [15.8% vs. 15.1%] | [15.3% vs. 16.4%] |
| ≥70 years | 0.3% | 0.2% | 0.1% | 0.0% | 0.0% | -0.1% | 0.0% | -0.7% |
|  | [1.6% vs. 1.3%] | [1.7% vs. 1.5%] | [1.9% vs. 1.8%] | [2.2% vs. 2.1%] | [2.5% vs. 2.5%] | [2.8% vs. 2.9%] | [3.3% vs. 3.4%] | [3.2% vs. 3.9%] |
| **Black/AA MSM** |  |  |  |  |  |  |  |  |
|  |  |  |  |  |  |  |  |  |
| <30 years | 0.8% | 1.8% | 1.9% | 2.6% | 3.7% | 3.7% | 4.0% | 4.4% |
|  | [19.4% vs. 18.6%] | [21.8% vs. 20.0%] | [23.3% vs. 21.3%] | [24.4% vs. 21.8%] | [25.4% vs. 21.8%] | [25.0% vs. 21.3%] | [24.7% vs. 20.6%] | [24.2% vs. 19.8%] |
| 30-39 years | -1.5% | -0.8% | 0.5% | 1.1% | 2.1% | 2.9% | 0.8% | 1.7% |
|  | [21.2% vs. 22.8%] | [20.7% vs. 21.5%] | [21.6% vs. 21.1%] | [22.4% vs. 21.2%] | [24.0% vs. 21.9%] | [26.0% vs. 23.0%] | [25.2% vs. 24.5%] | [27.8% vs. 26.1%] |
| 40-49 years | 1.9% | 0.5% | -0.6% | -2.1% | -4.2% | -4.9% | -4.9% | -4.7% |
|  | [36.7% vs. 34.8%] | [33.5% vs. 33.1%] | [30.6% vs. 31.2%] | [27.3% vs. 29.4%] | [23.3% vs. 27.6%] | [20.9% vs. 25.8%] | [19.2% vs. 24.0%] | [17.6% vs. 22.4%] |
| 50-59 years | -1.8% | -1.5% | -1.6% | -1.2% | -0.7% | -0.3% | 0.9% | 0.3% |
|  | [18.1% vs. 19.9%] | [19.2% vs. 20.8%] | [19.7% vs. 21.3%] | [20.6% vs. 21.8%] | [21.5% vs. 22.2%] | [22.2% vs. 22.5%] | [23.6% vs. 22.7%] | [23.0% vs. 22.7%] |
| 60-69 years | 0.3% | -0.2% | -0.6% | -0.8% | -1.2% | -1.6% | -1.0% | -1.7% |
|  | [4.0% vs. 3.7%] | [4.1% vs. 4.3%] | [4.2% vs. 4.8%] | [4.6% vs. 5.4%] | [4.8% vs. 6.0%] | [5.1% vs. 6.7%] | [6.4% vs. 7.4%] | [6.4% vs. 8.1%] |
| ≥70 years | 0.3% | 0.3% | 0.4% | 0.4% | 0.4% | 0.1% | 0.3% | 0.0% |
|  | [0.6% vs. 0.2%] | [0.6% vs. 0.3%] | [0.7% vs. 0.4%] | [0.8% vs. 0.4%] | [1.0% vs. 0.5%] | [0.8% vs. 0.7%] | [1.1% vs. 0.8%] | [0.9% vs. 1.0%] |
| **Hispanic MSM** |  |  |  |  |  |  |  |  |
|  |  |  |  |  |  |  |  |  |
| <30 years | 1.5% | 1.4% | 1.1% | 1.6% | 1.0% | 0.3% | -0.4% | -0.3% |
|  | [11.5% vs. 10.0%] | [12.1% vs. 10.7%] | [12.7% vs. 11.6%] | [13.7% vs. 12.1%] | [13.4% vs. 12.4%] | [12.7% vs. 12.5%] | [12.1% vs. 12.5%] | [12.1% vs. 12.4%] |
| 30-39 years | -4.2% | -1.8% | -0.8% | -0.9% | 0.8% | 2.0% | 2.3% | 2.6% |
|  | [28.1% vs. 32.3%] | [27.9% vs. 29.6%] | [26.9% vs. 27.7%] | [25.4% vs. 26.3%] | [26.2% vs. 25.4%] | [26.9% vs. 24.9%] | [27.3% vs. 24.9%] | [27.9% vs. 25.4%] |
| 40-49 years | 3.4% | 1.7% | 0.9% | -0.3% | -2.4% | -3.3% | -4.0% | -2.5% |
|  | [42.3% vs. 38.9%] | [40.8% vs. 39.0%] | [39.5% vs. 38.6%] | [37.6% vs. 37.9%] | [34.5% vs. 36.9%] | [32.4% vs. 35.7%] | [30.2% vs. 34.2%] | [30.0% vs. 32.5%] |
| 50-59 years | -1.2% | -1.5% | -0.8% | -0.3% | 0.3% | 0.8% | 1.4% | 0.7% |
|  | [14.0% vs. 15.2%] | [15.3% vs. 16.8%] | [17.2% vs. 18.0%] | [19.0% vs. 19.3%] | [20.8% vs. 20.5%] | [22.4% vs. 21.6%] | [23.9% vs. 22.5%] | [24.0% vs. 23.3%] |
| 60-69 years | 0.6% | 0.4% | -0.3% | -0.1% | 0.4% | 0.4% | 0.5% | -0.6% |
|  | [3.3% vs. 2.7%] | [3.4% vs. 3.0%] | [2.9% vs. 3.3%] | [3.5% vs. 3.6%] | [4.4% vs. 4.0%] | [4.8% vs. 4.4%] | [5.4% vs. 4.9%] | [4.8% vs. 5.5%] |
| ≥70 years | -0.1% | -0.3% | -0.1% | -0.1% | -0.1% | -0.1% | 0.2% | 0.1% |
|  | [0.7% vs. 0.9%] | [0.6% vs. 0.9%] | [0.7% vs. 0.9%] | [0.7% vs. 0.9%] | [0.7% vs. 0.9%] | [0.8% vs. 0.9%] | [1.2% vs. 0.9%] | [1.1% vs. 1.0%] |
| **White IDU men** |  |  |  |  |  |  |  |  |
|  |  |  |  |  |  |  |  |  |
| <30 years | 0.2% | 0.1% | 0.4% | 0.6% | 0.6% | 0.3% | -0.2% | -0.2% |
|  | [2.2% vs. 2.0%] | [2.1% vs. 2.0%] | [2.6% vs. 2.2%] | [2.9% vs. 2.3%] | [3.1% vs. 2.5%] | [3.1% vs. 2.7%] | [2.9% vs. 3.1%] | [3.3% vs. 3.5%] |
| 30-39 years | 0.0% | -0.4% | -0.4% | -0.2% | 0.0% | -0.4% | 3.2% | 1.3% |
|  | [10.6% vs. 10.6%] | [9.3% vs. 9.7%] | [8.9% vs. 9.3%] | [8.9% vs. 9.1%] | [9.2% vs. 9.2%] | [8.9% vs. 9.4%] | [12.9% vs. 9.7%] | [11.5% vs. 10.2%] |
| 40-49 years | -1.1% | -0.5% | -0.9% | -2.0% | -3.2% | -2.7% | 4.0% | 5.5% |
|  | [36.0% vs. 37.1%] | [34.1% vs. 34.6%] | [31.2% vs. 32.1%] | [27.6% vs. 29.6%] | [24.0% vs. 27.2%] | [22.0% vs. 24.7%] | [26.3% vs. 22.3%] | [25.6% vs. 20.1%] |
| 50-59 years | 0.1% | 0.7% | 0.5% | 0.4% | 1.1% | 0.5% | 0.2% | 1.4% |
|  | [38.5% vs. 38.4%] | [40.5% vs. 39.8%] | [41.0% vs. 40.5%] | [41.3% vs. 40.9%] | [42.2% vs. 41.1%] | [41.3% vs. 40.9%] | [40.5% vs. 40.3%] | [40.8% vs. 39.4%] |
| 60-69 years | 0.7% | 0.1% | 0.4% | 1.3% | 1.6% | 2.2% | -5.6% | -5.7% |
|  | [11.7% vs. 11.0%] | [12.9% vs. 12.8%] | [14.8% vs. 14.5%] | [17.4% vs. 16.1%] | [19.4% vs. 17.8%] | [21.7% vs. 19.5%] | [15.5% vs. 21.1%] | [17.0% vs. 22.7%] |
| ≥70 years | 0.1% | 0.0% | 0.0% | 0.0% | -0.1% | 0.1% | -1.6% | -2.3% |
|  | [0.9% vs. 0.9%] | [1.1% vs. 1.2%] | [1.5% vs. 1.5%] | [1.8% vs. 1.9%] | [2.2% vs. 2.3%] | [2.9% vs. 2.8%] | [1.8% vs. 3.4%] | [1.8% vs. 4.1%] |
| **Black/AA IDU men** |  |  |  |  |  |  |  |  |
|  |  |  |  |  |  |  |  |  |
| <30 years | 0.1% | 0.1% | -0.1% | -0.3% | -0.4% | -0.5% | 4.5% | 7.2% |
|  | [0.6% vs. 0.5%] | [0.7% vs. 0.6%] | [0.7% vs. 0.8%] | [0.8% vs. 1.1%] | [1.1% vs. 1.5%] | [1.5% vs. 2.0%] | [7.1% vs. 2.6%] | [10.6% vs. 3.4%] |
| 30-39 years | 0.1% | 0.0% | -0.3% | -0.5% | -0.8% | -1.2% | 3.2% | 5.6% |
|  | [2.6% vs. 2.5%] | [2.6% vs. 2.5%] | [2.5% vs. 2.8%] | [2.7% vs. 3.1%] | [2.7% vs. 3.5%] | [2.6% vs. 3.8%] | [7.3% vs. 4.1%] | [9.9% vs. 4.3%] |
| 40-49 years | -1.3% | -1.1% | -1.0% | -1.2% | -1.2% | -0.8% | 3.4% | 3.2% |
|  | [20.4% vs. 21.7%] | [17.1% vs. 18.2%] | [14.3% vs. 15.3%] | [11.7% vs. 12.9%] | [9.7% vs. 10.9%] | [8.5% vs. 9.3%] | [11.5% vs. 8.1%] | [10.5% vs. 7.2%] |
| 50-59 years | 0.6% | 0.6% | 0.7% | 1.0% | 0.6% | -1.0% | -0.5% | -2.6% |
|  | [56.1% vs. 55.4%] | [55.8% vs. 55.2%] | [54.6% vs. 53.8%] | [52.7% vs. 51.6%] | [49.3% vs. 48.7%] | [44.2% vs. 45.3%] | [40.9% vs. 41.4%] | [34.6% vs. 37.3%] |
| 60-69 years | 0.0% | 0.0% | 0.6% | 0.9% | 1.9% | 3.8% | -9.1% | -10.1% |
|  | [19.1% vs. 19.1%] | [22.4% vs. 22.4%] | [26.3% vs. 25.7%] | [30.1% vs. 29.2%] | [34.5% vs. 32.6%] | [39.7% vs. 35.9%] | [29.8% vs. 38.9%] | [31.4% vs. 41.5%] |
| ≥70 years | 0.4% | 0.4% | 0.1% | 0.0% | -0.2% | -0.2% | -1.5% | -3.3% |
|  | [1.3% vs. 0.8%] | [1.5% vs. 1.1%] | [1.7% vs. 1.6%] | [2.1% vs. 2.1%] | [2.6% vs. 2.9%] | [3.6% vs. 3.8%] | [3.4% vs. 4.8%] | [2.9% vs. 6.2%] |
| **Hispanic IDU men** |  |  |  |  |  |  |  |  |
|  |  |  |  |  |  |  |  |  |
| <30 years | 0.1% | -0.6% | -0.2% | 0.2% | -0.2% | -0.1% | 3.1% | 3.6% |
|  | [1.5% vs. 1.4%] | [1.0% vs. 1.6%] | [1.7% vs. 1.9%] | [2.3% vs. 2.1%] | [2.2% vs. 2.4%] | [2.5% vs. 2.6%] | [6.0% vs. 2.9%] | [6.8% vs. 3.2%] |
| 30-39 years | -1.2% | -0.2% | 0.6% | 0.3% | 0.5% | 0.8% | 10.7% | 12.3% |
|  | [9.8% vs. 11.0%] | [10.3% vs. 10.5%] | [10.8% vs. 10.1%] | [10.0% vs. 9.8%] | [10.1% vs. 9.5%] | [10.2% vs. 9.4%] | [20.1% vs. 9.4%] | [21.8% vs. 9.5%] |
| 40-49 years | 0.4% | -0.1% | -0.8% | -2.0% | -2.1% | -0.8% | 17.3% | 13.0% |
|  | [27.5% vs. 27.2%] | [24.9% vs. 25.0%] | [22.7% vs. 23.4%] | [20.2% vs. 22.2%] | [19.1% vs. 21.2%] | [19.6% vs. 20.4%] | [37.0% vs. 19.6%] | [32.0% vs. 19.0%] |
| 50-59 years | -0.1% | -0.8% | -1.0% | -0.7% | -0.4% | -5.8% | -10.4% | -5.6% |
|  | [45.9% vs. 46.0%] | [44.7% vs. 45.5%] | [43.3% vs. 44.3%] | [42.0% vs. 42.7%] | [40.5% vs. 40.9%] | [33.0% vs. 38.8%] | [26.1% vs. 36.5%] | [28.6% vs. 34.2%] |
| 60-69 years | 0.5% | 1.3% | 0.9% | 2.1% | 2.3% | 6.0% | -19.1% | -20.6% |
|  | [14.3% vs. 13.8%] | [17.8% vs. 16.5%] | [19.9% vs. 19.0%] | [23.5% vs. 21.4%] | [26.0% vs. 23.7%] | [31.8% vs. 25.8%] | [8.7% vs. 27.8%] | [8.8% vs. 29.4%] |
| ≥70 years | 0.3% | 0.3% | 0.5% | 0.3% | -0.1% | 0.0% | -1.6% | -2.7% |
|  | [0.9% vs. 0.6%] | [1.2% vs. 0.9%] | [1.7% vs. 1.2%] | [1.9% vs. 1.7%] | [2.2% vs. 2.3%] | [2.9% vs. 2.9%] | [2.2% vs. 3.8%] | [2.0% vs. 4.8%] |
| **White IDU women** |  |  |  |  |  |  |  |  |
|  |  |  |  |  |  |  |  |  |
| <30 years | 0.6% | 1.6% | 0.7% | -0.4% | 0.3% | 0.8% | 2.1% | 0.6% |
|  | [3.9% vs. 3.3%] | [4.4% vs. 2.8%] | [3.3% vs. 2.6%] | [2.0% vs. 2.4%] | [2.5% vs. 2.2%] | [2.8% vs. 2.1%] | [4.0% vs. 2.0%] | [2.5% vs. 1.9%] |
| 30-39 years | -0.6% | 0.5% | 0.6% | 2.0% | 0.3% | -0.1% | -1.0% | 0.0% |
|  | [21.9% vs. 22.5%] | [20.7% vs. 20.2%] | [19.1% vs. 18.5%] | [19.2% vs. 17.2%] | [16.5% vs. 16.3%] | [15.4% vs. 15.4%] | [13.8% vs. 14.8%] | [14.2% vs. 14.2%] |
| 40-49 years | 0.6% | -1.8% | -2.3% | -2.0% | -2.6% | -2.0% | 2.3% | 3.0% |
|  | [41.9% vs. 41.2%] | [38.2% vs. 40.0%] | [36.0% vs. 38.3%] | [34.5% vs. 36.5%] | [31.9% vs. 34.5%] | [30.8% vs. 32.7%] | [33.3% vs. 31.0%] | [32.7% vs. 29.7%] |
| 50-59 years | 0.6% | 0.9% | 2.2% | 1.1% | 3.0% | 3.1% | -0.9% | -3.5% |
|  | [28.7% vs. 28.1%] | [31.7% vs. 30.9%] | [35.4% vs. 33.2%] | [36.0% vs. 34.9%] | [39.2% vs. 36.3%] | [40.3% vs. 37.2%] | [36.7% vs. 37.6%] | [34.2% vs. 37.6%] |
| 60-69 years | -1.6% | -1.1% | -0.9% | -0.3% | -0.2% | -0.9% | -1.6% | 0.9% |
|  | [3.1% vs. 4.7%] | [4.8% vs. 5.9%] | [6.3% vs. 7.2%] | [8.3% vs. 8.6%] | [9.9% vs. 10.1%] | [10.8% vs. 11.7%] | [11.8% vs. 13.4%] | [16.0% vs. 15.1%] |
| ≥70 years | 0.4% | -0.1% | -0.3% | -0.5% | -0.7% | -0.9% | -0.8% | -1.1% |
|  | [0.6% vs. 0.2%] | [0.2% vs. 0.3%] | [0.0% vs. 0.3%] | [0.0% vs. 0.5%] | [0.0% vs. 0.7%] | [0.0% vs. 0.9%] | [0.3% vs. 1.2%] | [0.4% vs. 1.5%] |
| **Black/AA IDU women** |  |  |  |  |  |  |  |  |
|  |  |  |  |  |  |  |  |  |
| <30 years | 0.2% | 0.3% | 0.0% | -0.4% | -0.4% | -0.4% | 1.5% | 1.9% |
|  | [0.6% vs. 0.4%] | [0.8% vs. 0.5%] | [0.5% vs. 0.5%] | [0.2% vs. 0.6%] | [0.2% vs. 0.6%] | [0.2% vs. 0.6%] | [2.2% vs. 0.7%] | [2.7% vs. 0.7%] |
| 30-39 years | -0.7% | -0.3% | 0.4% | 1.1% | -0.3% | 0.2% | 1.6% | 1.2% |
|  | [7.1% vs. 7.9%] | [6.0% vs. 6.3%] | [5.7% vs. 5.3%] | [5.8% vs. 4.8%] | [4.1% vs. 4.4%] | [4.4% vs. 4.3%] | [5.7% vs. 4.1%] | [5.3% vs. 4.1%] |
| 40-49 years | 0.4% | -3.1% | -2.9% | -2.7% | -0.8% | -2.6% | 3.8% | 3.6% |
|  | [37.6% vs. 37.1%] | [30.4% vs. 33.5%] | [27.1% vs. 30.0%] | [24.0% vs. 26.8%] | [22.9% vs. 23.7%] | [18.2% vs. 20.8%] | [22.0% vs. 18.2%] | [19.6% vs. 16.0%] |
| 50-59 years | -1.6% | 0.1% | -0.7% | -1.2% | -1.2% | 0.9% | -5.7% | -3.0% |
|  | [47.1% vs. 48.6%] | [51.6% vs. 51.5%] | [52.2% vs. 53.0%] | [52.1% vs. 53.3%] | [51.4% vs. 52.6%] | [52.0% vs. 51.1%] | [43.2% vs. 48.9%] | [43.1% vs. 46.2%] |
| 60-69 years | 1.7% | 2.9% | 2.8% | 2.5% | 2.4% | 1.9% | -1.3% | -3.8% |
|  | [7.4% vs. 5.8%] | [10.9% vs. 8.0%] | [13.6% vs. 10.8%] | [16.7% vs. 14.2%] | [20.4% vs. 18.0%] | [24.2% vs. 22.3%] | [25.6% vs. 26.8%] | [27.6% vs. 31.3%] |
| ≥70 years | 0.0% | 0.1% | 0.6% | 0.8% | 0.4% | 0.0% | 0.1% | 0.0% |
|  | [0.2% vs. 0.1%] | [0.3% vs. 0.2%] | [0.8% vs. 0.3%] | [1.2% vs. 0.4%] | [1.0% vs. 0.6%] | [0.9% vs. 0.9%] | [1.3% vs. 1.2%] | [1.8% vs. 1.8%] |
| **Hispanic IDU women** |  |  |  |  |  |  |  |  |
|  |  |  |  |  |  |  |  |  |
| <30 years | 1.2% | 0.3% | 0.1% | 0.6% | -0.3% | -0.3% | -0.2% | -0.2% |
|  | [3.5% vs. 2.3%] | [1.7% vs. 1.4%] | [1.0% vs. 0.9%] | [1.1% vs. 0.5%] | [0.0% vs. 0.3%] | [0.0% vs. 0.3%] | [0.0% vs. 0.2%] | [0.0% vs. 0.2%] |
| 30-39 years | -0.2% | 1.0% | 1.7% | 2.7% | -0.3% | -2.2% | -0.2% | 10.0% |
|  | [9.6% vs. 9.7%] | [10.4% vs. 9.4%] | [11.0% vs. 9.3%] | [12.0% vs. 9.2%] | [8.8% vs. 9.1%] | [6.7% vs. 8.9%] | [8.3% vs. 8.5%] | [18.2% vs. 8.1%] |
| 40-49 years | -2.6% | -1.7% | -6.0% | -7.2% | -4.1% | -1.3% | 5.9% | 0.6% |
|  | [35.7% vs. 38.3%] | [32.2% vs. 33.8%] | [24.0% vs. 30.0%] | [19.6% vs. 26.8%] | [19.8% vs. 23.8%] | [20.0% vs. 21.3%] | [25.0% vs. 19.1%] | [18.2% vs. 17.6%] |
| 50-59 years | 0.3% | -0.8% | -0.9% | 0.1% | -2.9% | 2.3% | -28.9% | -26.0% |
|  | [41.7% vs. 41.5%] | [43.5% vs. 44.3%] | [45.0% vs. 45.9%] | [46.7% vs. 46.7%] | [44.0% vs. 46.9%] | [48.9% vs. 46.6%] | [16.7% vs. 45.5%] | [18.2% vs. 44.1%] |
| 60-69 years | 0.7% | 0.6% | 4.7% | 2.6% | 6.7% | -1.3% | 9.3% | 0.5% |
|  | [8.7% vs. 8.0%] | [11.3% vs. 10.7%] | [18.0% vs. 13.3%] | [18.5% vs. 15.9%] | [25.3% vs. 18.6%] | [20.0% vs. 21.3%] | [33.3% vs. 24.0%] | [27.3% vs. 26.8%] |
| ≥70 years | 0.6% | 0.5% | 0.4% | 1.2% | 0.9% | 2.7% | 14.2% | 15.0% |
|  | [0.9% vs. 0.2%] | [0.9% vs. 0.4%] | [1.0% vs. 0.6%] | [2.2% vs. 0.9%] | [2.2% vs. 1.3%] | [4.4% vs. 1.8%] | [16.7% vs. 2.4%] | [18.2% vs. 3.2%] |
| **White heterosexual men** |  |  |  |  |  |  |  |  |
|  |  |  |  |  |  |  |  |  |
| <30 years | 0.4% | 0.2% | 0.0% | 1.0% | 1.7% | 1.1% | 0.2% | 0.5% |
|  | [3.0% vs. 2.6%] | [2.7% vs. 2.5%] | [2.5% vs. 2.5%] | [3.6% vs. 2.6%] | [4.4% vs. 2.6%] | [3.8% vs. 2.7%] | [3.0% vs. 2.9%] | [3.5% vs. 3.1%] |
| 30-39 years | -1.0% | -2.7% | -2.3% | -2.7% | -2.8% | -1.7% | -1.0% | -0.3% |
|  | [12.5% vs. 13.5%] | [11.1% vs. 13.8%] | [11.6% vs. 14.0%] | [11.2% vs. 13.8%] | [10.7% vs. 13.5%] | [11.2% vs. 12.9%] | [11.3% vs. 12.4%] | [11.5% vs. 11.8%] |
| 40-49 years | 0.0% | 0.4% | -0.6% | -0.7% | -1.8% | -0.1% | -2.8% | -1.4% |
|  | [37.5% vs. 37.5%] | [35.2% vs. 34.8%] | [31.8% vs. 32.4%] | [29.4% vs. 30.1%] | [26.2% vs. 28.0%] | [26.1% vs. 26.2%] | [21.6% vs. 24.4%] | [21.3% vs. 22.7%] |
| 50-59 years | -0.8% | 0.8% | 2.3% | 1.6% | 2.6% | 0.9% | 0.5% | 1.0% |
|  | [31.0% vs. 31.8%] | [34.1% vs. 33.2%] | [36.7% vs. 34.4%] | [37.1% vs. 35.5%] | [39.1% vs. 36.5%] | [37.9% vs. 37.0%] | [37.6% vs. 37.1%] | [37.8% vs. 36.9%] |
| 60-69 years | 0.5% | 0.5% | -0.1% | -0.4% | -0.2% | -0.2% | 1.6% | -1.1% |
|  | [12.3% vs. 11.8%] | [13.1% vs. 12.7%] | [13.4% vs. 13.5%] | [14.1% vs. 14.5%] | [15.5% vs. 15.6%] | [16.6% vs. 16.9%] | [19.9% vs. 18.3%] | [18.9% vs. 20.0%] |
| ≥70 years | 1.0% | 0.8% | 0.8% | 1.2% | 0.4% | -0.1% | 1.6% | 1.4% |
|  | [3.7% vs. 2.6%] | [3.8% vs. 2.9%] | [3.9% vs. 3.2%] | [4.6% vs. 3.5%] | [4.2% vs. 3.9%] | [4.3% vs. 4.3%] | [6.5% vs. 4.9%] | [6.9% vs. 5.6%] |
| **Black/AA heterosexual men** |  |  |  |  |  |  |  |  |
|  |  |  |  |  |  |  |  |  |
| <30 years | -0.2% | -0.4% | 0.0% | -0.1% | 0.1% | 0.9% | 0.7% | 0.9% |
|  | [3.7% vs. 3.9%] | [3.9% vs. 4.3%] | [4.7% vs. 4.7%] | [4.7% vs. 4.8%] | [4.9% vs. 4.8%] | [5.6% vs. 4.7%] | [5.3% vs. 4.6%] | [5.3% vs. 4.4%] |
| 30-39 years | 1.0% | 1.3% | 1.6% | 1.7% | 1.9% | 2.2% | 1.2% | 1.3% |
|  | [17.1% vs. 16.1%] | [16.0% vs. 14.7%] | [15.3% vs. 13.8%] | [14.8% vs. 13.1%] | [14.5% vs. 12.6%] | [14.5% vs. 12.4%] | [13.4% vs. 12.3%] | [13.6% vs. 12.4%] |
| 40-49 years | -1.5% | -2.0% | -2.8% | -2.6% | -2.3% | -1.4% | 0.4% | 1.6% |
|  | [40.1% vs. 41.6%] | [37.5% vs. 39.5%] | [34.5% vs. 37.2%] | [32.4% vs. 35.0%] | [30.4% vs. 32.7%] | [28.9% vs. 30.4%] | [28.5% vs. 28.1%] | [27.5% vs. 25.9%] |
| 50-59 years | 1.0% | 0.9% | 1.3% | 1.1% | 0.5% | -1.1% | -0.5% | -1.2% |
|  | [30.0% vs. 28.9%] | [31.8% vs. 30.9%] | [33.9% vs. 32.6%] | [35.3% vs. 34.2%] | [36.0% vs. 35.5%] | [35.5% vs. 36.6%] | [36.8% vs. 37.4%] | [36.5% vs. 37.7%] |
| 60-69 years | -1.1% | -0.6% | -0.5% | -0.4% | -0.4% | -0.5% | -1.1% | -1.5% |
|  | [6.8% vs. 7.9%] | [8.2% vs. 8.8%] | [9.2% vs. 9.7%] | [10.4% vs. 10.8%] | [11.5% vs. 11.9%] | [12.8% vs. 13.3%] | [13.6% vs. 14.7%] | [14.8% vs. 16.2%] |
| ≥70 years | 0.7% | 0.8% | 0.5% | 0.3% | 0.2% | -0.1% | -0.7% | -1.1% |
|  | [2.3% vs. 1.6%] | [2.5% vs. 1.8%] | [2.4% vs. 1.9%] | [2.5% vs. 2.2%] | [2.6% vs. 2.4%] | [2.6% vs. 2.7%] | [2.3% vs. 3.0%] | [2.3% vs. 3.4%] |
| **Hispanic heterosexual men** |  |  |  |  |  |  |  |  |
|  |  |  |  |  |  |  |  |  |
| <30 years | -0.1% | -0.6% | -0.2% | -0.2% | 0.1% | 0.5% | -0.6% | -0.9% |
|  | [6.0% vs. 6.0%] | [4.9% vs. 5.5%] | [4.8% vs. 5.0%] | [4.2% vs. 4.4%] | [4.1% vs. 3.9%] | [3.9% vs. 3.4%] | [2.5% vs. 3.1%] | [1.8% vs. 2.8%] |
| 30-39 years | -1.9% | -0.6% | -0.3% | -0.1% | -0.2% | 1.6% | -0.5% | -0.4% |
|  | [24.6% vs. 26.6%] | [23.6% vs. 24.2%] | [22.2% vs. 22.4%] | [20.8% vs. 20.9%] | [19.5% vs. 19.7%] | [20.1% vs. 18.5%] | [17.0% vs. 17.5%] | [16.2% vs. 16.6%] |
| 40-49 years | 0.0% | -1.2% | -2.1% | -1.0% | -1.2% | 0.4% | -1.3% | 1.5% |
|  | [36.7% vs. 36.7%] | [35.7% vs. 36.9%] | [34.3% vs. 36.4%] | [34.5% vs. 35.5%] | [33.1% vs. 34.3%] | [33.4% vs. 33.0%] | [30.5% vs. 31.8%] | [32.4% vs. 30.8%] |
| 50-59 years | 2.3% | 2.7% | 1.9% | 1.2% | 0.3% | -0.3% | 3.3% | 1.0% |
|  | [23.0% vs. 20.7%] | [25.2% vs. 22.5%] | [26.3% vs. 24.3%] | [27.2% vs. 26.1%] | [27.9% vs. 27.6%] | [28.4% vs. 28.7%] | [32.7% vs. 29.4%] | [30.9% vs. 29.9%] |
| 60-69 years | -0.5% | -0.8% | 0.1% | -0.4% | 0.6% | -1.9% | -0.5% | -0.3% |
|  | [7.5% vs. 8.0%] | [7.9% vs. 8.7%] | [9.6% vs. 9.4%] | [10.0% vs. 10.4%] | [12.2% vs. 11.6%] | [11.0% vs. 12.9%] | [13.7% vs. 14.2%] | [15.1% vs. 15.4%] |
| ≥70 years | 0.2% | 0.5% | 0.6% | 0.6% | 0.3% | -0.2% | -0.4% | -0.9% |
|  | [2.2% vs. 2.0%] | [2.7% vs. 2.2%] | [2.9% vs. 2.4%] | [3.3% vs. 2.6%] | [3.3% vs. 3.0%] | [3.2% vs. 3.4%] | [3.6% vs. 3.9%] | [3.7% vs. 4.6%] |
| **White heterosexual women** |  |  |  |  |  |  |  |  |
|  |  |  |  |  |  |  |  |  |
| <30 years | 0.8% | 1.5% | 1.0% | 1.3% | 1.3% | 1.0% | 1.3% | 1.9% |
|  | [6.4% vs. 5.6%] | [7.3% vs. 5.9%] | [7.2% vs. 6.2%] | [7.3% vs. 6.0%] | [6.8% vs. 5.6%] | [5.9% vs. 4.9%] | [5.5% vs. 4.2%] | [5.4% vs. 3.5%] |
| 30-39 years | -2.1% | -3.0% | -0.5% | 0.4% | -0.2% | 0.0% | -1.0% | -0.3% |
|  | [21.6% vs. 23.6%] | [17.7% vs. 20.7%] | [18.2% vs. 18.7%] | [17.9% vs. 17.5%] | [16.6% vs. 16.8%] | [16.3% vs. 16.3%] | [14.9% vs. 15.9%] | [15.2% vs. 15.5%] |
| 40-49 years | -1.3% | -0.2% | -1.2% | -3.0% | -2.4% | -3.1% | -5.2% | -1.6% |
|  | [37.6% vs. 38.9%] | [38.2% vs. 38.4%] | [36.0% vs. 37.2%] | [32.9% vs. 35.8%] | [31.8% vs. 34.2%] | [29.5% vs. 32.6%] | [25.8% vs. 31.0%] | [28.0% vs. 29.6%] |
| 50-59 years | 2.0% | 1.5% | 0.5% | 0.9% | 0.0% | 1.4% | 4.0% | 0.7% |
|  | [25.2% vs. 23.2%] | [26.8% vs. 25.3%] | [27.5% vs. 27.0%] | [29.4% vs. 28.6%] | [30.0% vs. 30.0%] | [32.6% vs. 31.2%] | [36.2% vs. 32.2%] | [33.6% vs. 32.9%] |
| 60-69 years | 0.6% | 0.4% | 0.4% | 0.3% | 0.8% | 0.2% | -0.3% | -1.4% |
|  | [7.6% vs. 7.1%] | [8.3% vs. 8.0%] | [9.2% vs. 8.8%] | [10.1% vs. 9.8%] | [11.6% vs. 10.8%] | [12.2% vs. 12.0%] | [12.9% vs. 13.2%] | [13.2% vs. 14.6%] |
| ≥70 years | 0.0% | -0.1% | -0.2% | 0.1% | 0.5% | 0.5% | 1.3% | 0.8% |
|  | [1.6% vs. 1.6%] | [1.7% vs. 1.9%] | [1.9% vs. 2.1%] | [2.4% vs. 2.3%] | [3.2% vs. 2.7%] | [3.5% vs. 3.0%] | [4.7% vs. 3.4%] | [4.6% vs. 3.8%] |
| **Black/AA heterosexual women** |  |  |  |  |  |  |  |  |
|  |  |  |  |  |  |  |  |  |
| <30 years | 0.0% | -0.1% | 0.2% | 0.3% | 0.4% | 0.5% | 0.3% | 0.9% |
|  | [8.9% vs. 8.8%] | [8.1% vs. 8.2%] | [7.9% vs. 7.8%] | [7.5% vs. 7.2%] | [7.0% vs. 6.6%] | [6.5% vs. 5.9%] | [5.6% vs. 5.2%] | [5.5% vs. 4.6%] |
| 30-39 years | -1.7% | -1.5% | -0.6% | 0.2% | 0.4% | 1.5% | -0.3% | -0.9% |
|  | [25.5% vs. 27.2%] | [23.9% vs. 25.3%] | [23.2% vs. 23.7%] | [22.6% vs. 22.4%] | [21.6% vs. 21.2%] | [21.8% vs. 20.2%] | [19.1% vs. 19.3%] | [17.7% vs. 18.6%] |
| 40-49 years | 1.2% | 1.8% | 0.7% | 0.3% | -0.1% | 0.1% | 1.2% | 0.8% |
|  | [36.5% vs. 35.3%] | [36.9% vs. 35.1%] | [35.3% vs. 34.6%] | [34.4% vs. 34.1%] | [33.3% vs. 33.4%] | [32.8% vs. 32.6%] | [32.9% vs. 31.7%] | [31.6% vs. 30.7%] |
| 50-59 years | 0.3% | -0.5% | -0.7% | -0.6% | -0.4% | -1.8% | -0.9% | 0.2% |
|  | [22.7% vs. 22.4%] | [23.6% vs. 24.1%] | [24.9% vs. 25.6%] | [26.3% vs. 26.8%] | [27.6% vs. 28.0%] | [27.3% vs. 29.1%] | [29.1% vs. 30.0%] | [31.0% vs. 30.8%] |
| 60-69 years | -0.3% | 0.0% | 0.2% | -0.5% | -0.7% | -1.1% | -0.8% | -1.3% |
|  | [5.3% vs. 5.6%] | [6.5% vs. 6.5%] | [7.7% vs. 7.5%] | [8.0% vs. 8.5%] | [8.8% vs. 9.5%] | [9.5% vs. 10.6%] | [11.0% vs. 11.9%] | [11.8% vs. 13.1%] |
| ≥70 years | 0.5% | 0.4% | 0.2% | 0.3% | 0.5% | 0.6% | 0.5% | 0.3% |
|  | [1.1% vs. 0.5%] | [1.1% vs. 0.7%] | [1.0% vs. 0.8%] | [1.3% vs. 1.0%] | [1.7% vs. 1.3%] | [2.2% vs. 1.5%] | [2.3% vs. 1.8%] | [2.5% vs. 2.2%] |
| **Hispanic heterosexual women** |  |  |  |  |  |  |  |  |
|  |  |  |  |  |  |  |  |  |
| <30 years | 0.5% | 0.7% | 0.9% | 0.8% | -0.2% | 0.5% | -1.2% | 1.0% |
|  | [8.7% vs. 8.2%] | [7.7% vs. 7.0%] | [7.0% vs. 6.1%] | [6.1% vs. 5.3%] | [4.6% vs. 4.7%] | [4.8% vs. 4.3%] | [2.9% vs. 4.0%] | [4.8% vs. 3.9%] |
| 30-39 years | -0.3% | -0.5% | -0.8% | -0.7% | 0.1% | 1.3% | 4.2% | 4.1% |
|  | [28.5% vs. 28.8%] | [27.4% vs. 27.9%] | [26.2% vs. 27.0%] | [25.2% vs. 25.9%] | [24.7% vs. 24.7%] | [24.7% vs. 23.4%] | [26.3% vs. 22.1%] | [25.0% vs. 20.9%] |
| 40-49 years | 1.1% | 0.8% | 0.5% | 0.9% | 1.0% | 1.2% | 2.2% | 5.0% |
|  | [34.6% vs. 33.5%] | [33.5% vs. 32.7%] | [32.4% vs. 32.0%] | [32.2% vs. 31.3%] | [31.7% vs. 30.7%] | [31.3% vs. 30.1%] | [31.7% vs. 29.5%] | [33.9% vs. 28.9%] |
| 50-59 years | -2.9% | -2.2% | -1.7% | -1.2% | -0.6% | -1.0% | -3.5% | -4.2% |
|  | [19.9% vs. 22.8%] | [22.1% vs. 24.3%] | [23.7% vs. 25.4%] | [25.1% vs. 26.3%] | [26.6% vs. 27.1%] | [26.6% vs. 27.6%] | [24.4% vs. 27.9%] | [23.8% vs. 28.0%] |
| 60-69 years | 1.4% | 0.4% | 0.6% | -0.2% | -0.8% | -2.1% | -1.9% | -5.8% |
|  | [7.5% vs. 6.1%] | [7.7% vs. 7.2%] | [9.1% vs. 8.5%] | [9.6% vs. 9.7%] | [10.2% vs. 11.0%] | [10.2% vs. 12.4%] | [11.7% vs. 13.7%] | [9.3% vs. 15.0%] |
| ≥70 years | 0.3% | 0.8% | 0.6% | 0.4% | 0.4% | 0.1% | 0.2% | 0.0% |
|  | [0.9% vs. 0.6%] | [1.6% vs. 0.8%] | [1.7% vs. 1.1%] | [1.8% vs. 1.3%] | [2.1% vs. 1.7%] | [2.3% vs. 2.2%] | [2.9% vs. 2.7%] | [3.2% vs. 3.3%] |

The discrepancies were <10% difference in all subgroups and years (15 subgroups * 8 calendar years = 120 subgroups and years) with the exception of 12 (10%) subgroup and year cells. The biggest differences were in Hispanic males and females who injected drugs, the subgroups with smallest observed sample size (and therefore largest estimate variances) in the NA-ACCORD.

As a benchmark, could the authors show what co-morbidities the model would predict in HIV-negative people, and whether the model does a reasonable job of those predictions? This could help validate the comorbidity predictions in a broader population and troubleshoot issues like linear relationships with age.

This agent-based simulation model is composed of mathematical functions from observed data of people with HIV. To forecast comorbidity in people without HIV would require building a separate model with mathematical functions from observed data of people without HIV; this is outside the scope of the PEARL model. We note the scope of the model in the Discussion:

*“We did not compare the comorbidities and multimorbidity forecasts to similar people without HIV. Although this comparison would be useful, our goal was to provide future morbidity predictions to inform HIV clinical directors’ planning and direct HIV policy decision-making.”*

Overall, a very interesting study, but the model may require further debugging and validation to ensure the reported predictions are accurate.

PEARL is a dynamic model. We are always improving the model to ensure the best calibration possible and have re-run all estimates for this re-submission to incorporate improvements made to PEARL in the last 6 months (since our Jan 5, 2023 submission).

To document improvements to the PEARL model, we are diligent about updating the methods section of our website (<https://pearlhivmodel.org/method_details.html>). Additionally, we have posted the code for the PEARL model that produced these results (<https://github.com/PearlHivModelingTeam/comorbidityPaper>) and added this link to the second paragraph of the Methods section.

**Statistical Reviewer**

This paper describes a series of projections of comorbidity in people living with HIV in the USA. I was asked for a statistical report and I interpret that to include all aspects of the design and conduct of the study. The version I received did not have page numbers. My numbering has the abstract starting on page 6.

The .pdf file of the submitted manuscript includes the abstract on page 17.

*Points of detail*

Page 6 The results here are presented as facts in the past (`increased', `de-creased') rather than projections (`would increase') which seems potentially misleading in the abstract. In the main text it is fine.

We begin the reporting of results by stating “PEARL forecasts showed” to clarify that these are future forecasts produced from a model.

Page 10 I am afraid I did not understand the paragraph on validation and Figure S2 did not enlighten me. I suspect a few more words would clarify it.

Although there is ambiguity about which concepts the reviewer comments are unclear in the “*Validation*” sub-section of the Methods section, we have attempted to re-write the paragraph for clarity:

*“We compared the estimated annual incidence of each comorbidity from the NA-ACCORD data with the forecasts from PEARL during the calibration period (where both observed NA-ACCORD estimates and PEARL forecasts were available, i.e., 2009-2017). Within each subgroup, we noted the comorbidities with <75% of PEARL forecasts falling within 5% of the NA-ACCORD observed incidence prevalence or within the NA-ACCORD observed 95% confidence (whichever interval was larger,* ***Supplement Figure S1****). We repeated this validation approach for the annual prevalence of each comorbidity (****Supplement Figure S2****).”*

Page 11 The sentence starting `The number of Black/AA MWID and WWID' would make more sense to me if `but' was replaced by `and' as the two clauses do not seem to contradict one another. Have I failed to under-

stand the meaning here? I assume decreased is a typo for decrease.

We have replaced “but” with “and.” “Decreased” is a typo for decrease; this has been corrected.

Page 15 Some of the speculation about limitations seems to me to go beyond what the data shows. It would be equally plausible that the selective mortality from COVID would have removed the people with most morbidities resulting in fewer surviving to 2030.

We have added this to the limitations paragraph in the Discussion:

*“The PEARL model assumes that the trends observed during the calibration period will continue and does not account for influences occurring outside the calibration period (e.g., the impact of emerging care technologies like long-acting ART or the impact of COVID-19 on mortality); such factors influencing comorbidities incidence can be incorporated into PEARL by extending the calibration period when there are more current data available within the 15 subgroups.”*

Page 15 I am not an HIV researcher but I wonder whether the widespread adoption of pre-exposure prophylaxis should be mentioned too. Obviously it cannot yet be in the model since its long-term effects are as yet unknown.”

We appreciate this insight. We added the following statement to the last paragraph of the Discussion: *“The PEARL model does not include HIV transmission. The forecasted annual number of new ART initiators relies on CDC's reported HIV diagnoses and linkage to care during the calibration period and does not consider changes in HIV transmission dynamics after the calibration period, including changes due to HIV prevention efforts (e.g., pre-exposure prophylaxis or PrEP) or during the COVID-19 pandemic (e.g., 2020-2021).“*

Table 1 What are the numbers in parentheses or brackets?

The brackets are the interquartile range (IQR) of the median age for each of the subgroups noted. This is conveyed in the row header “*Subgroups, n % (median age) [IQR*].”

Figure 3 The advertised shading did not appear in the version I received although I suspect it would make the plots unreadable if it did. The different comorbidities are quite hard to distinguish here. Different line type might help to clear up which is which. I had to blow up the PDF to 200% on my ancient monitor to even see there were different symbols. For some reason Figures S3a-o do have shading and more distinguishable lines.

Shaded uncertainty ranges are present around line in *Figure 3*, but the uncertainty range is so narrow due to the large sample size of all agents. You may notice the shading around the estimates close to the year 2030 in *Figure 3. Figures S3a-*o have more visible shading because the sample size of agents is small in each subgroup, so the shaded uncertainty ranges are more visible.

There are different line types for each comorbidity, visible on more recent computer monitors. Additionally, green is used to predict the mental comorbidities with higher prevalence at younger ages, yellow represents the comorbidities more prevalent in middle age, and red comorbidities represent late-stage comorbidities.

Figure 5 It looks Cam did sort the plot but then forgot to delete the comment.

The comment has been omitted.

Mostly for clarification. I will leave it to the clinical reviewers to comment on the plausibility of the projections.

Michael Dewey

**COMMENTS FROM THE ACADEMIC EDITOR**

I agree that this is potentially an interesting paper, for the US readers and policy-makers. It projects the potential burden of multimorbidity in people on ART by ethnic, sex and risk group, in the USA.

However, what I think is missing is the information that a clinician would like to have (rather than a policy/programme person) in understanding whether there is a greater or lesser likelihood that the person with HIV on ART under his/her care will develop multimorbidities, and which ones. With that knowledge some preventive action may be possible. Would it be possible to use the output from the model to give an example of what change there would be from 2020 to 2030 for such a clinician? The projected prevalences of multimorbidities are not that high, and although this is important to know about a population-level, it is not entirely clear for the clinician.

The PEARL model is a population-based simulation of people living with HIV, with the capacity to forecast trends in aging with HIV and burden of comorbidities through the year 2030 among 15 key subgroups in the US. As the academic editor correctly alludes to, the PEARL model can act as **a decision support tool** for HIV policymakers to inform future HIV care policies in the US: for example, the PEARL projections suggest an alarming increase in burden of CKD among PWH over the next decade which will assert high financial burdens, particularly if renal replacement therapy is needed. Black women with injection drug use as their HIV acquisition risk group have a high burden of CKD, and this group may experience additional barriers to accessing care. Such forecasts can provide information to plan for CKD healthcare costs and lower barriers to nephrology care in anticipation of this growing healthcare need.

Following this, the take-home message for clinicians is that comorbidity care needs of their patient populations (as a mix of the 15 subgroups) is likely to change from 2020-2030, potentially forcing the need for clinicians to stay up-to-date on care guidelines for the most burdensome comorbidities, or build/strengthen connections with subspecialty clinicians who care for the most burdensome comorbidities.

The PEARL model can also act as an experimental tool to forecast the impact of preventive interventions for different comorbidities. We are currently working on a new study focusing on impact of a weight management program in the first 24 months after ART initiation on future reductions in incident diabetes diagnosis and other comorbidities among persons aging with HIV. Such results can inform the design of new clinical trials to assess the impact of such programs and to inform clinicians about the potential effectiveness of such interventions at the patient- and population-level.

Finally, it is important to keep in mind that the PEARL model is programmed as a series of mathematical functions that assign incident comorbidities to the agents as they age with HIV, ultimately producing a **population-level perspective** on the changing burdens of comorbidities and multimorbidity through 2030. This is a different methodologic and conceptual approach than developing prediction models for risk of disease among patients, as has been done via other models such as the *Framingham Risk Score for Hard Coronary Heart Disease* (<https://www.framinghamheartstudy.org/fhs-risk-functions/cardiovascular-disease-10-year-risk/>). While such models can predict an individual’s risk for certain comorbidities, they cannot forecast the population-level burden of these comorbidities in a defined population; this is the main aim of the PEARL model.

Also, the age distribution in PEARL 2020 and projected PEARL 2030 is somewhat different. How much of the increase is associated with changes in the projected age distribution of people on ART?

The age distributions within the 15 subgroups and overall change from 2020 to 2030, and are fully described in Althoff KN et al., AIDS, 2022 (doi: 10.1097/QAD.00000000000031280). It is unclear which outcome the editors are referring to when they inquire “How much of the increase,” but age is playing a role in the mathematical functions estimating the probability of each comorbidity (see the coefficients for age [βage] in *Supplemental Table S2*).

The model allows for age, but is it possible to clarify whether some (or all) of the projected multimorbidity increase is related to increases in particular age groups (as opposed to a general increase overall)? Would there be changes in the distribution of multi-morbidity by age in 2030, compared to 2020? What drives this increase?

The following Figure shows the forecasted burden of multimorbidity among people with HIV in the US by age group from 2010 to 2030. Along with the increase in population size of people with HIV and the aging phenomena, the older age groups show an increasing number of people with HIV in 2030 compared to 2010. Moreover, among those surviving to older ages, the proportion of persons living with 2+ physical comorbidities is also increasing (69% of persons age 70+ living with 2+ comorbidities in 2020 [n= 28,568 persons], and 80% of persons age 70+ living with 2+ comorbidities in 2030 [n=113,291 persons]).

This forecasted trend in aging and the burden of multimorbidity is heterogeneous among the 15 subgroups. In response to this comment, we have now included the set of plots showing the trend in multimorbidity burden by age groups among all people with HIV and 15-subgroups in the Supplement (**Figure S3a-p**) and noted it in the Results:

*“Multimorbidity burden by age group overall and within each of the 15 subgroups is available in* ***Supplement Figure S3a-p****.”*

Further, why is there no allowance for ART regimen? And ART history? Both regimen and previous history could bear on prevalence/incidence of morbidity. This is something that should at least be discussed, and if the data are not available then it should be noted under limitations.

Please see our more elaborate response to this question posed by Reviewer #1 (above).

Briefly, the mathematical functions predicting comorbidity incidence do not explicitly include ART regimens. Because the functions were estimated among people who initiated ART observed in the NA-ACCORD, these associations are implicitly impacting the frequency of outcomes. We have added the following limitation to the Discussion:

*“ART regimen type is not explicitly contained in the PEARL model, but the impact of various ART regimens on comorbidity incidence is implicitly contained in the mathematical functions from observed NA-ACCORD data.”*

It is not clear to me how specific to the PEARL model and available data this model is - can it be generalized across the USA (assuming not all HIV infected people start ART and likelihood of starting may change over time). This discussion could be informed by providing some understanding of what drives the increases in multimorbidity.

We have added the following sentence to the second paragraph of the Methods section:

*“Due to the structure of PEARL and the data sources, findings are generalizable to PWH using ART in the US who identify with the 15 subgroups included in PEARL.”*

The Discussion includes the following statements of what is driving the increases in multimorbidity:

*“**Among the comorbidities included in the PEARL model, the forecasts suggest that two of the greatest contributors to multimorbidity are two common mental health diagnoses that occur throughout the lifespan: depression and anxiety. The prevalence of anxiety was forecasted to increase in all the subgroups by the year 2030 and by 10 ppc overall. In 2030, the prevalence of mental or physical multimorbidity is forecasted to be 70% and nearly 1 in 3 (31%) PWH using ART will have mental comorbidity and physical multimorbidity by 2030; these estimates are a lower limit due to the inclusion of only 2 mental and 7 physical comorbidities when forecasting multimorbidity.”*

*“Over the next decade, these metabolic and vascular diseases may drive the PEARL-forecasted increases in dyslipidemia, CKD, diabetes, and MIs. Importantly, the prevalence of hypertension is forecasted to decline slightly by 4 ppc to 33% in 2030, perhaps due to increases in younger, healthier adults entering HIV care through successful test-and-treat (without delay) initiatives and the “Treat All” clinical guidelines, the reduced use of protease inhibitors (which have been linked to metabolic disorders), or the greater likelihood of a plateau or decrease when prevalence is high.^18,45^ Although hypertension prevalence may decline slightly, the MI burden is forecasted to increase, which is influenced by the increasing prevalence of other comorbidities and the increasing number of PWH at older ages by 2030.^14,46^”*

*“Implementing clinical program interventions that are tailored to Black/AA women and focused on prevention and management of diabetes, hypertension, and anxiety may prove beneficial in slowing future multimorbidity growth among Black/AA WWID. The PEARL modeling team is pursuing estimates of forecasted comorbidities in scenarios where a prominent risk factor is reduced by a specified amount.”*

**Comments from the editors, sent via e-mail on July 11, August 4, August 7, and August 2x, 2023**

1.Please amend your detailed Financial Disclosure statement according to point “a” or point “b” below. This is published with the article. It must therefore be completed in full sentences and contain the exact wording you wish to be published.

a.If authors did not receive any funding for this study, please simply state: “The authors received no specific funding for this work.”

b.If authors received funding for this study, please state the initials, alongside each funding source, of each author to receive each grant. For example: "This work was supported by the National Institutes of Health (####### to AM; ###### to CJ) and the National Science Foundation (###### to AM)." Also, please state what role the funders took in the study. If the funders had no role in your study, please state: “The funders had no role in study design, data collection and analysis, decision to publish, or preparation of the manuscript.”

We have amended the Financial Disclosure statement following the instructions provided.

2. Please ensure that the funders and grant numbers match between the Financial Disclosure field and the Funding Information tab in your submission form. Note that the funders must be provided in the same order in both places as well. Currently, the Funding Information box in your submission form states “Office of AIDS Research (U01 AI069918) Keri N Althoff" whereas the same statement is not present at the Financial Disclosure field.

We requested the addition of “Jerome and Celia Reich Endowed Scholar Award” to the drop-down list of funders and it was added by a Olena Szabo, Journal Assistant.

Per the suggestion of Olena Szabo on August 7 (in response to our inquiry), our Financial Disclosure statement now reads: “The presented research was supported by the National Institutes of Health (R01 AG053100 to KNA; U01AI069918 to KNA and RDM; K01AI138853 to PK; R01AG069575 to EPH) and the Jerome and Celia Reich Endowed Scholar Award (to EPH). The funders had no role in study design, data collection and analysis, decision to publish, or preparation of the manuscript.”

We have moved the NA-ACCORD funding statement to the Acknowledgements (following the Discussion and before the References), at the direction of Olena Szabo.

The Financial Disclosure statement in the manuscript and the Financial Disclosure fields in the PLoS Medicine online submission system are now identical.

3.We have amended your Competing Interest statement to comply with journal style. We kindly ask that you double check the statement and let us know if anything is incorrect.

To my knowledge, this is correct.

4.We have amended your Competing Interest statement to include a competing interest declared by your co-author, Dr. KG, PFR, JT, VFM, HNK. Please review the added statement below and let us know if anything is incorrect.

“KG declares that his institution receives funding from U.S. Department of Defense’s (DOD) Joint Program Executive Office for Chemical, Biological, Radiological and Nuclear Defense (JPEO-CBRND), in collaboration with the Defense Health Agency (DHA) (contract number: W911QY2090012) Bloomberg Philanthropies, State of Maryland, NIH National Center for Advancing Translational Sciences (NCATS) U24TR001609, Division of Intramural Research NIAID NIH, Mental Wellness Foundation, Moriah Fund, Octapharma, HealthNetwork Foundation, and the Shear Family Foundation for his work. KG received royalties from UptoDate and served as a paid consultant to Aspen Institute, adn Teach for America. KG declares that none of these funding sources are related to this manuscript.

PFR declares consultation with Gilead & Janssen pharmaceuticals (money paid to individual); research grants from NIH/NIAID (money paid to institution).

JT declares to be Consultant for AbbVie, Canfield, Gilead, Roche and Tarsier and being Equity owner for Tarsier.

VFM has received support from the Emory CFAR (P30 AI050409) and received investigator-initiated research grants (to the institution) and consultation fees (both unrelated to the current work) from Eli Lilly, Bayer, Gilead Sciences, and ViiV.

HNK declares that Gilead Sciences program funding paid to the author’s institution.”

To my knowledge, this is correct; we corrected incorrect pronouns and typos.

5.We have noticed that your submission form mentions that an ethics statement is included in the manuscript. In the manuscript file we have not found an ethics statement under the subheading of the methods section. Please insert an Ethics Statement at the beginning of your Methods section, under a subheading 'Ethics Statement'. It must include:

a. The name(s) of the Institutional Review Board(s) or Ethics Committee(s)

b. The approval number(s), or a statement that approval was granted by the named board(s)

c.In case the study involved a human participants/donors please include: A statement that formal consent was obtained (must state whether verbal/written) OR the reason consent was not obtained (e.g. anonymity). NOTE: If child participants, the statement must declare that formal consent was obtained from the parent/guardian.

The following has been added:

**“Ethics Statement**

The PEARL model was classified as “Exempt under 45 CRF 46.101(b), Category (4)” by the Johns Hopkins Bloomberg School of Public Health Institutional Review Board.”

6. Please ensure that the Title in your manuscript file and the Title provided in your online submission form are the same. Currently the manuscript has the title as “The forecasted prevalence of comorbidities and multimorbidity in people with HIV in the United States through the year 2030: A modeling study” whereas the title in the submission is written as “The projected prevalence of comorbidities and multimorbidity in people with HIV in the United States through the year 2030”

The title “The forecasted prevalence of comorbidities and multimorbidity in people with HIV in the United States through the year 2030: A modeling study” is now in both places (online submission form and manuscript).

7.We have noticed that you have uploaded Supporting Information files, but you have not included a list of legends. Please add a full list of legends for your Supporting Information files after the references list.

We have included a list of legends on the first page of the supplement (i.e., Supporting Information document).

8.We have noticed that Supplementary / Supporting Figure S3 is not included in your supporting information files. Please amend the supporting information file in a way that Figure S3 file is included in it.

We have amended the Supplement to include Figure S3.

9.Some material included in your submission may be copyrighted. According to PLOS’s copyright policy, authors who use figures or other material (e.g., graphics, clipart, maps) from another author or copyright holder must demonstrate or obtain permission to publish this material under the Creative Commons Attribution 4.0 International (CC BY 4.0) License used by PLOS journals. Please closely review the details of PLOS’s copyright requirements here: PLOS Licenses and Copyright. If you need to request permissions from a copyright holder, you may use PLOS's Copyright Content Permission form.

Please respond directly to this email and provide any known details concerning your material's license terms and permissions required for reuse, even if you have not yet obtained copyright permissions or are unsure of your material's copyright compatibility. Once you have responded and addressed all other outstanding technical requirements, you may resubmit your manuscript within Editorial Manager.

Potential Copyright Issues:

‘Figure 1A’: Please confirm whether you drew the images / clip-art within the figure panels by hand. If you did not draw the images, please provide (a) a link to the source of the images or icons and their license / terms of use; or (b) written permission from the copyright holder to publish the images or icons under our CC BY 4.0 license. Alternatively, you may replace the images with open source alternatives. See these open source resources you may use to replace images / clip-art:

We have removed the CDC logo in Figure 1.

The image of the people within Figure 1A has been replaced with images from Wikimedia Commons (available at [https://commons.wikimedia.org/wiki/File:Person_icon_(based_on_Noun-Project-3797130).svg](https://nam02.safelinks.protection.outlook.com/?url=https%3A%2F%2Fcommons.wikimedia.org%2Fwiki%2FFile%3APerson_icon_(based_on_Noun-Project-3797130).svg&data=05%7C01%7Ckalthoff%40jhu.edu%7C656783b3d71d45ab117108dba285a9ec%7C9fa4f438b1e6473b803f86f8aedf0dec%7C0%7C0%7C638282467191203592%7CUnknown%7CTWFpbGZsb3d8eyJWIjoiMC4wLjAwMDAiLCJQIjoiV2luMzIiLCJBTiI6Ik1haWwiLCJXVCI6Mn0%3D%7C3000%7C%7C%7C&sdata=DyVZf%2FeehNuwsF0uEjzTuy40%2Bs4BQbHUuGSlAh8U5sQ%3D&reserved=0)). The source of the image is specified in the footnote as directed by Olena Szabo, Journal Assistant, on August 22, 2023. We believe the figure is now copyright compatible.
