# Supplementary material for: The forecasted prevalence of comorbidities and multimorbidity in people with HIV in the United States through the year 2030: A modeling study
Source: PLoS Med. 2024 Jan 12;21(1):e1004325. doi: 10.1371/journal.pmed.1004325 (PMC10833859; doi:10.1371/journal.pmed.1004325)
Supplement: Attachment — Submitted filename: 2023_1027 response to reviewers.docx [file pmed.1004325.s018.docx]

**Response to reviewers submitted October 6, 2023 (in response to comments from the editors sent via e-mail on September 15, 2023)**

**COMMENTS FROM THE ACADEMIC EDITOR**

I am a little unsure about this paper - the model is becoming more complex but the value of the paper for the readership of PLoS Med has become more, rather than less. And I do not see what is new knowledge? I continue to struggle with who this paper is for - given the various caveats noted by the authors in their responses to the previous comments.

We confirmed the Academic Editor misplace the words “more” and “less.”

We believe your readership includes ***HIV clinicians who make care decisions for a panel of patients with HIV***. HIV clinicians should continue to use the *HIV and the Older Person* section of the *Guidelines for the Use of Antiretroviral Agents in Adults and Adolescents with HIV^[1]^* and clinical decision-making tools (like the Atherosclerotic Cardiovascular Disease [ASCVD] Risk Estimator) when making clinical care decisions for an individual patient with HIV. Clinicians should also review PEARL’s output to identify the comorbidity-specific screening, diagnoses, and treatment guidelines and tools that will be necessary to care for their panel of patients. For example, the rise in anxiety diagnoses suggest the need to screen for anxiety symptoms among individuals with HIV. The United States Preventative Services Task Force issued a recommendation in June 2023 that adults <64 years should be screened for anxiety disorders, but there is insufficient evidence to recommend screening for anxiety disorders in adults ≥65 years. Given the disproportionately high prevalence of anxiety in people with HIV over the next decade, clinicians may implement a screening tool (such as the Generalized Anxiety Disorder 7-item located in the National HIV Curriculum for the US and available at <https://www.hiv.uw.edu/page/mental-health-screening/gad-7>).^[2]^ Additionally, HIV clinicians will need to be mindful of anxiety symptom side-effects of comorbidity treatments. Another example is the rise in the prevalence of dyslipidemia and myocardial infarction over the next decade. Given the recent findings of the REPRIEVE phase 3 clinical trial findings on statin use guidelines for people with HIV, clinicians will want to stay informed on future guideline changes for statins in people with HIV to attenuate the forecasted steep increases in dyslipidemia and myocardial infarction.^[3]^

We believe your readership also includes ***HIV care program decision-makers***, such as HIV clinical directors. The findings from the PEARL model provide forecasts of the rise in the comorbidity and multimorbidity prevalence of people aging with HIV (and heterogeneity within 15 key population subgroups), for whom healthcare will be needed. The PEARL output will help HIV clinical directors determine gaps in comorbidity treatment knowledge among clinicians within their HIV clinic and choose continuing medical education opportunities to address such gaps. Additionally, clinical directors will need to predict the subspecialities that will be in highest demand by their clinical population and either bring those subspecialists to the HIV clinic or arrange for referrals to subspecialists engaged in caring for people with HIV.

Finally, we believe your readership also includes ***HIV policy decision-makers***. The PEARL model output suggests the demand for subspecialty care for persons aging with HIV will exceed the capacity of the current HIV care models. The Department of Health and Human Services funding applicants in response to the following announcements through Ryan White HIAV/AIDS Program Part F Special Project of National Significance Awards: (a) People Aging with HIV: Capacity-Building Provider (HRSA-22027); and (b) Emerging Strategies to Improve Health Outcomes for People Aging with HIV: Capacity-Demonstration Sites (HRSA-22-028 and HRSA-22-029). The comorbidity and multimorbidity forecasts from PEARL will inform the selection of HIV care models that meet the healthcare needs of people with HIV, as well as resource allocation to those services.

The Discussion has been extensively revised to state the link between these various readerships and the findings, including:

- In the first paragraph of the Discussion: *“Our findings show the most prevalent comorbidities in the next decade will differ by gender, HIV acquisition risk group, race, and ethnicity, suggesting the clinical population composition is important when preparing to meet the future care needs of people with HIV. HIV clinicians must be up-to-date on comorbidity-specific screening, diagnoses, and treatment guidelines and clinical decision-making tools that will be in highest demand by the subgroups represented in their panel of patients with HIV. HIV clinical directors must select comorbidities-specific refresher courses for their clinical staff and establish subspecialty care access within their clinics or by referral. HIV policy decision-makers must identify care models with capacity and ensure adequate payor resources (e.g., the Ryan White HIV/AIDS Program funding) to meet the growing healthcare needs, in particular the mental healthcare needs, of PWH using ART.”*
- In the second paragraph of the Discussion: *“With the forecasted rise in anxiety prevalence among people with HIV, clinicians should consult the US Preventive Services Task Force’s recommendation to screen for anxiety symptoms in those age <64 years (HIV-specific recommendations are not available), and be mindful of comorbidity medications that have been linked to symptoms of anxiety when caring for people with HIV.(43) Mental health services are allowable costs for PWH eligible for the federally-allocated Ryan White HIV/AIDS Program support; funding mental health services within HIV clinics or by referral and ensuring adequate staffing will be necessary to meet the forecasted prevalence of mental health comorbidity.“*
- In the third paragraph of the Discussion: *“The prevalence of these metabolic and vascular diseases shown in the NA-ACCORD data influence the PEARL-forecasted increases in dyslipidemia, CKD, diabetes, and MIs. With the recent findings from the REPRIEVE phase 3 clinical trial demonstrating a protective effect of pitavastatin on cardiovascular events, clinicians will need to stay informed on guideline changes for statins in people with HIV and recognize the gap between people with HIV prescribed statins and those eligible for statins.(45,46)”*
- In the last paragraph of the Discussion: “*HIV clinicians must consider a host of comorbidity-specific guidelines to care for the increasing prevalence of comorbidities and multimorbidity among people with HIV. HIV clinical program and policy decision-makers must act now to identify effective multidisciplinary care models and resources to prevent and manage comorbidities and multimorbidity among the growing population of PWH using ART in the US.”*

I remain unclear as to how much of the increase in multimorbidity is due to the ageing of the HIV cohort in the USA, and whether there is any much change in the incidence/prevalence of multimorbidity by age forecast over the period to 2030.

As the Academic Editor noted, the increase in multimorbidity prevalence from 2020 to 2030 is influenced by the aging of the population; however, multimorbidity prevalence is also increasing within decades of age, suggesting the contribution of risk factors – other than age alone – to the increase in multimorbidity prevalence. i.e., multimorbidity increased from 45% to 50% of those age 50-59 years from 2020 to 2030:

In response to this question, we have now added this figure to the supplement (**Figure S4a**) and included it in the Results:
*“To demonstrate the influence of risk factors – other than age alone – on forecasted multimorbidity, the multimorbidity prevalence by decade of age is shown in* ***Supplement Figure S4a-p****, which depicts an increase in multimorbidity prevalence in all age groups ≥50 years.”*

We have also commented on this in the Discussion: *“The PEARL-forecasted age-specific multimorbidity prevalence increased from 2020-2030 among those age 50-59, 60-69, and ≥70 years suggesting the contribution of risk factors – in addition to age itself – is resulting in an increased risk of comorbidities among older adults with HIV.”*

Reviewer 2 has a point regarding the age of the black women with injecting drug use - but that may be because there is ageing of the USA cohort with younger cohorts having a different HIV acquisition factor compared to older cohorts. And also, in their population, injecting drug use only account for a small percentage of all acquisition risk.

Please see our response to Reviewer #2 on the topic of Black women with injection drug use for an HIV acquisition risk factor (below).

To me, this paper does not show an understanding of HIV itself, and how HIV would interact with ageing. Further, the point about not being able to, or not seeing the need, to include ART regimen as a factor in the forecast model is not a satisfactory response. There may not be much data for individual ART regimen, but it should be possible to divide ART regimen in classes, on the basis of how they work, what they address and what side effects there may be. Surely, an HIV clinical expert would know this. To me this is an important point as there are changes in ART regimen and if those would be associated with changes in specific multimorbidity patterns than that is something policymakers and programme leaders should be aware of.

In our prior response, we agreed with the Academic Editor about the importance of adding ART regimen into the PEARL model. Where we disagree with the Academic Editor is the suggestion that ART regimen type is necessary to address the objective of our study: “To forecast the prevalence of future comorbidities and multimorbidity among PWH using ART in the US through the year 2030, overall and within 15 demographic subgroups.”

The probabilities of comorbidity incidence and subsequent multimorbidity were estimated using observational data of people with HIV in the NA-ACCORD, many of whom have long histories of ART use that include multiple changes in regimen class:

Source: NA-ACCORD, www.naaccord.org/monitoring.

Our decision for excluding ART regimen from the current analysis is grounded in the complexity of modeling changes in ART regimen over time. The intricate nature of potential switches in ART regimen at an individual level, coupled with the absence of necessary data to model these shifts at a subgroup level, poses a challenge. Additional factors, such as clinical practice and patient’s response to specific regiment, also extend beyond the current scope of the PEARL model. We are considering funding and data sources to support the inclusion of ART regimen class in the PEARL model for future research questions of ART regimen on HIV and comorbidity outcomes that would be answered with PEARL forecasts.

While ART regimen class is not explicitly included as a variable in our model, the influence of time-varying ART regimen class use is present in the data used to estimate the probabilities of incident comorbidities in the PEARL model.

The Discussion includes the following statement:

*“ART regimen class is not explicitly contained in the PEARL model, but the impact of various ART regimen classes on comorbidity incidence is implicitly contained in the mathematical functions from observed NA-ACCORD data, which is reflective of exposures to ART in PWH in the US; future expansion of the PEARL model to include ART regimen class is possible.”*

The conclusion of the abstract states:

*Conclusion and relevance: The distribution of multimorbidity will continue to differ by race/ethnicity, gender, and HIV acquisition risk subgroups, and be influenced by age and risk factor distributions that reflect the impact of social disparities of the health on women, people of color, and people who use drugs. HIV clinical care models and funding are urgently required to meet the healthcare needs of people with HIV in the next decade.*

But no specifics are provided and this leaves the reader wondering what then the paper contributes to knowledge useful for clinical care?

The Conclusions and relevance have been revised as follow:

*“Comorbidity and multimorbidity prevalence are heterogenous by race/ethnicity, gender, and HIV acquisition risk subgroups. HIV clinicians must stay current on the ever-changing comorbidities-specific guidelines to provide guideline-recommended care. HIV clinical directors should ensure linkages to subspecialty care within the clinic or by referral. HIV policy decision-makers must allocate resources and support extended clinical capacity to meet the healthcare needs of people aging with HIV.”*

COMMENTS FROM THE REVIEWERS:

Reviewer #2: The authors have been responsive to most of the suggestions from this reviewer, except for two crucial ones:

- Longer LE in Black WWID: The authors postulate that Black women who inject drugs may have longer life expectancy due to gender differences in life expectancy, a survivor bias effect, or national reductions in HIV incidence among PWID. To me, none of these putative mechanisms make sense as a plausible driver of the model results - and some explanations simply do not make sense in the context of this model. I urge the authors to think deeply and investigate thoroughly, i.e., use their data and model to interrogate the underlying causes of this unexpected result. Spurious results are often useful "leads' that help to diagnose bugs in the model. Better to find out now than have this lead to a retraction of the article in the future.

As the reviewer suggested, we worked to further validate the number and age distribution of Black women with injection drug use as their HIV acquisition risk factor using HIV surveillance data from the US Centers for Disease Control and Prevention (CDC, believed to be a complete capture of all persons with HIV in the US) and the observed NA-ACCORD data, which is the largest capture of people with HIV who have linked into HIV care in the US. Our investigation findings are described below.

The number and age distribution of Black women with injection drug use as their HIV acquisition risk factor with diagnosed HIV are a function of the following:

- Very low numbers of new HIV infections in this subgroup: The number of new HIV diagnosis among Black women with injection drug use as their HIV acquisition risk factor has consistently declined since 2009 when new HIV diagnoses were concentrated in 45-54 year olds in 2009. In 2021, there were similar numbers of new HIV diagnoses in the among those 25-34 years, 35-44 years, and 55+ years:^[4]^

Number of New HIV diagnoses

| Age groups (from the CDC) | 2009 | 2011 | 2013 | 2015 | 2017 | 2019 | 2021 |
| --- | --- | --- | --- | --- | --- | --- | --- |
| 13-24 | 114 | 88 | 70 | 64 | 71 | 62 | 57 |
| 25-34 | 251 | 167 | 146 | 146 | 157 | 195 | 157 |
| 35-44 | 448 | 288 | 180 | 153 | 156 | 161 | 161 |
| 45-54 | 641 | 411 | 289 | 198 | 165 | 139 | 121 |
| 55+ | 356 | 284 | 252 | 196 | 197 | 191 | 177 |

- The concentration of deaths in older ages: The number of deaths in Black women with injection drug use as their HIV acquisition risk factor peaked in the 45-54 year old age group in 2009. By 2015 the peak occurred in the 55+ year old age group. In 2021, 71% of all deaths occurring in Black women with injection drug use as their HIV acquisition risk factor were occurring among those 55+ years:^[5]^

Number of Deaths

| Age group (from CDC) | 2009 | 2011 | 2013 | 2015 | 2017 | 2019 | 2021 |
| --- | --- | --- | --- | --- | --- | --- | --- |
| 13-24 | 7 | 5 | 0 | 2 | 2 | 1 | 1 |
| 25-34 | 86 | 63 | 44 | 17 | 39 | 21 | 24 |
| 35-44 | 418 | 264 | 177 | 164 | 96 | 77 | 80 |
| 45-54 | 842 | 646 | 579 | 372 | 373 | 285 | 257 |
| 55+ | 518 | 546 | 664 | 629 | 671 | 714 | 866 |

The result of change over time in the number of, and age, at HIV diagnosis and death among that Black women with injection drug use as their HIV acquisition risk factor is a small and diminishing subgroup of PWH in the US with an older age distribution:^[6]^

Number of Black women with injection drug use as their HIV acquisition risk factor with diagnosed HIV

| Age group (from CDC) | 2009 | 2011 | 2013 | 2015 | 2017 | 2019 | 2021 |
| --- | --- | --- | --- | --- | --- | --- | --- |
| 13-24 | 335 | 274 | 224 | 165 | 145 | 129 | 109 |
| 25-34 | 2135 | 1843 | 1533 | 1301 | 1110 | 991 | 873 |
| 35-44 | 7089 | 5676 | 4564 | 3645 | 3050 | 2663 | 2354 |
| 45-54 | 10868 | 10751 | 10062 | 9169 | 7918 | 6555 | 5300 |
| 55+ | 5129 | 6607 | 8164 | 9637 | 11134 | 12556 | 13499 |

The size and age distribution among Black women with injection drug use as their HIV acquisition risk factor agents is supported by the CDC’s HIV surveillance data (which is believed to be a complete ascertainment of all persons with diagnosed HIV in the US). It is also supported by the NA-ACCORD data, which is the largest cohort of people with HIV who have linked to care in the US. Below is a depiction to demonstrate the agreement between the CDC’s HIV Surveillance data (available via AtlasPlus^[7]^), the NA-ACCORD data, and the PEARL estimates for the Black women with injection drug use as their HIV acquisition risk factor in 2010, 2013, and 2017:

The CDC data is available publicly in age groups specified by the CDC in the AtlasPlus online database. The AtlasPlus estimates were plotted as follows: estimate for those age 13-24 years was plotted at age 20 years; estimate for those aged 25-34 years was plotted at age 30 years; estimate for those age 35-44 years were potted at age 40 years; estimate for those age 45-54 years were plotted at age 55 years; estimate for those age ≥55 years was plotted at age 60 years. Access Atlas Plus here: <https://www.cdc.gov/nchhstp/atlas/index.htm>:

Further, the lack of awareness of these differential age distributions and subsequent differential prevalence of comorbidities and multimorbidity patterns is evidence that PEARL model’s subgroup output is important and necessary to inform preparations for meeting the healthcare needs of the heterogenous population of people aging with HIV in the United States.

We have modified the Discussion as follows:

*“*Subgroup stratification is essential due to the heterogeneity of PWH in the US. For example, Black/AA WWID are forecasted to have the oldest age distribution in 2030, which is a function of a reduction in new HIV diagnoses among people who inject drugs (which are more likely to occur in young people) and the older age at death in this subgroup (as demonstrated in the CDC’s HIV surveillance data);^a^ the age distribution estimated by PEARL reflected the distribution observe in the NA-ACCORD (**Supplement Figure S3**).

^a^ US Centers for Disease Control and Prevention. NCHHSTP AtlasPlus. https://www.cdc.gov/nchhstp/atlas/index.htm. Accessed October 2023.

- Fitting: The reviewer requested that the authors graph the age distributions from their modeled population vs. the observed population. Instead, the reviewer provided 8 pages of tables listing % differences between model and data in different strata. The requested graph would show the actual population by age group, graphed as a distribution, and overlaying these for model vs. data on the same graph. This visualization approach will be much more interpretable and revealing of the model's validity, versus the 8-page table.

We agree that a visual comparison of forecasted vs observed age distribution is easier to interpret. However, presenting such comparison for each calibration years (2009-2017) within each of 15-subgroups will similarly take several pages.

In response to reviewer’s request, we have added the following plot to the supplement (**Supplement Figure S3)**, showing a schematic comparison at selected years (2010, 2013, 2017) for all 15 subgroups:

We have also added the 8-page table (previously sent in response to reviewers ) to the Supplement (**Supplement Table S5**) because the figure above does not 1) include all years of calibration (2009-2017) and 2) does not reveal the important details of when the difference in the NA-ACCORD and PEARL estimates are greater than 5% percentage points.

Reviewer #3: The authors have addressed my points.

Michael Dewey

Thank you, Dr. Dewey.

ADDITIONAL COMMENTS/REQUESTS

FIGURES

Please consider avoiding the use of red and or green to improve accessibility of your figures to those with colour blindness.

The programmer who created these figures is color blind, and selected colors he felt were contrasting. However, your comment motivated us to look for color blindness accessibility standards in the published literature. We found *Wong B. Points of view: Color blindness. Nature Methods 2011;8:441* which included this helpful chart for color optimization for color blind individuals:

The programmer selected the color schemes for Figures 2a, 2b, 3a, 3b, and 3c from these options. Please also note that the markers of the yearly prevalence in figures 3a, 3b, and 3c differentiate the lines (in addition to the color).

Figures associated with your paper as individual TIF or EPS files with 300dpi resolution at resubmission

We believe we have met these requirements and have uploaded the figures to PACE (as instructed) and no problems were detected.

Figure 5 - you clearly define the meaning of the dots and the lines for the reader in the caption.

Thank you.

DISCUSSION

Please remove the sub-heading ‘conclusion’ such that it reads as continuous prose.

Completed.

REFERENCES

In the bibliography please list up to but no more than 6 author names followed by et al in the event than more than 6 authors contribute to an individual study.

Please ensure all web references include an ‘Accessed [date]’

Completed

SUPPORTING INFORMATION

In the published article, supporting information files are accessed only through a hyperlink attached to the captions. For this reason, you must list captions at the end of your manuscript file. You may include a caption within the supporting information file itself, as long as that caption is also provided in the manuscript file. Do not submit a separate caption file.

We have listed the titles for the Section, Table, and Figure numbers in the Supplement following the REFERENCES (the information provided matches what is within the text of the manuscript).

Supplementary table 6 – in the footnote, please revised CDK to read CKD

Completed. Please note: the number has been revised to Supplement Table S7 (not S6).

**Response to reviewers submitted August 22, 2023 (in response to comments from the editors sent via e-mail on July 11, August 4, and August 7)**

1.Please amend your detailed Financial Disclosure statement according to point “a” or point “b” below. This is published with the article. It must therefore be completed in full sentences and contain the exact wording you wish to be published.

a.If authors did not receive any funding for this study, please simply state: “The authors received no specific funding for this work.”

b.If authors received funding for this study, please state the initials, alongside each funding source, of each author to receive each grant. For example: "This work was supported by the National Institutes of Health (####### to AM; ###### to CJ) and the National Science Foundation (###### to AM)." Also, please state what role the funders took in the study. If the funders had no role in your study, please state: “The funders had no role in study design, data collection and analysis, decision to publish, or preparation of the manuscript.”

We have amended the Financial Disclosure statement following the instructions provided.

2. Please ensure that the funders and grant numbers match between the Financial Disclosure field and the Funding Information tab in your submission form. Note that the funders must be provided in the same order in both places as well. Currently, the Funding Information box in your submission form states “Office of AIDS Research (U01 AI069918) Keri N Althoff" whereas the same statement is not present at the Financial Disclosure field.

We requested the addition of “Jerome and Celia Reich Endowed Scholar Award” to the drop-down list of funders and it was added by a Olena Szabo, Journal Assistant.

Per the suggestion of Olena Szabo on August 7 (in response to our inquiry), our Financial Disclosure statement now reads: “The presented research was supported by the National Institutes of Health (R01 AG053100 to KNA; U01AI069918 to KNA and RDM; K01AI138853 to PK; R01AG069575 to EPH) and the Jerome and Celia Reich Endowed Scholar Award (to EPH). The funders had no role in study design, data collection and analysis, decision to publish, or preparation of the manuscript.”

We have moved the NA-ACCORD funding statement to the Acknowledgements (following the Discussion and before the References), at the direction of Olena Szabo.

The Financial Disclosure statement in the manuscript and the Financial Disclosure fields in the PLoS Medicine online submission system are now identical.

3.We have amended your Competing Interest statement to comply with journal style. We kindly ask that you double check the statement and let us know if anything is incorrect.

To my knowledge, this is correct.

4.We have amended your Competing Interest statement to include a competing interest declared by your co-author, Dr. KG, PFR, JT, VFM, HNK. Please review the added statement below and let us know if anything is incorrect.

“KG declares that his institution receives funding from U.S. Department of Defense’s (DOD) Joint Program Executive Office for Chemical, Biological, Radiological and Nuclear Defense (JPEO-CBRND), in collaboration with the Defense Health Agency (DHA) (contract number: W911QY2090012) Bloomberg Philanthropies, State of Maryland, NIH National Center for Advancing Translational Sciences (NCATS) U24TR001609, Division of Intramural Research NIAID NIH, Mental Wellness Foundation, Moriah Fund, Octapharma, HealthNetwork Foundation, and the Shear Family Foundation for his work. KG received royalties from UptoDate and served as a paid consultant to Aspen Institute, adn Teach for America. KG declares that none of these funding sources are related to this manuscript.

PFR declares consultation with Gilead & Janssen pharmaceuticals (money paid to individual); research grants from NIH/NIAID (money paid to institution).

JT declares to be Consultant for AbbVie, Canfield, Gilead, Roche and Tarsier and being Equity owner for Tarsier.

VFM has received support from the Emory CFAR (P30 AI050409) and received investigator-initiated research grants (to the institution) and consultation fees (both unrelated to the current work) from Eli Lilly, Bayer, Gilead Sciences, and ViiV.

HNK declares that Gilead Sciences program funding paid to the author’s institution.”

To my knowledge, this is correct; we corrected incorrect pronouns and typos.

5.We have noticed that your submission form mentions that an ethics statement is included in the manuscript. In the manuscript file we have not found an ethics statement under the subheading of the methods section. Please insert an Ethics Statement at the beginning of your Methods section, under a subheading 'Ethics Statement'. It must include:

a. The name(s) of the Institutional Review Board(s) or Ethics Committee(s)

b. The approval number(s), or a statement that approval was granted by the named board(s)

c.In case the study involved a human participants/donors please include: A statement that formal consent was obtained (must state whether verbal/written) OR the reason consent was not obtained (e.g. anonymity). NOTE: If child participants, the statement must declare that formal consent was obtained from the parent/guardian.

The following has been added:

**“Ethics Statement**

The PEARL model was classified as “Exempt under 45 CRF 46.101(b), Category (4)” by the Johns Hopkins Bloomberg School of Public Health Institutional Review Board.”

6. Please ensure that the Title in your manuscript file and the Title provided in your online submission form are the same. Currently the manuscript has the title as “The forecasted prevalence of comorbidities and multimorbidity in people with HIV in the United States through the year 2030: A modeling study” whereas the title in the submission is written as “The projected prevalence of comorbidities and multimorbidity in people with HIV in the United States through the year 2030”

The title “The forecasted prevalence of comorbidities and multimorbidity in people with HIV in the United States through the year 2030: A modeling study” is now in both places (online submission form and manuscript).

7.We have noticed that you have uploaded Supporting Information files, but you have not included a list of legends. Please add a full list of legends for your Supporting Information files after the references list.

We have included a list of legends on the first page of the supplement (i.e., Supporting Information document).

8.We have noticed that Supplementary / Supporting Figure S3 is not included in your supporting information files. Please amend the supporting information file in a way that Figure S3 file is included in it.

We have amended the Supplement to include Figure S3.

9.Some material included in your submission may be copyrighted. According to PLOS’s copyright policy, authors who use figures or other material (e.g., graphics, clipart, maps) from another author or copyright holder must demonstrate or obtain permission to publish this material under the Creative Commons Attribution 4.0 International (CC BY 4.0) License used by PLOS journals. Please closely review the details of PLOS’s copyright requirements here: PLOS Licenses and Copyright. If you need to request permissions from a copyright holder, you may use PLOS's Copyright Content Permission form.

Please respond directly to this email and provide any known details concerning your material's license terms and permissions required for reuse, even if you have not yet obtained copyright permissions or are unsure of your material's copyright compatibility. Once you have responded and addressed all other outstanding technical requirements, you may resubmit your manuscript within Editorial Manager.

Potential Copyright Issues:

‘Figure 1A’: Please confirm whether you drew the images / clip-art within the figure panels by hand. If you did not draw the images, please provide (a) a link to the source of the images or icons and their license / terms of use; or (b) written permission from the copyright holder to publish the images or icons under our CC BY 4.0 license. Alternatively, you may replace the images with open source alternatives. See these open source resources you may use to replace images / clip-art:

We have removed the CDC logo in Figure 1.

The image of the people within Figure 1A has been replaced with images from Wikimedia Commons (available at [https://commons.wikimedia.org/wiki/File:Person_icon_(based_on_Noun-Project-3797130).svg](https://nam02.safelinks.protection.outlook.com/?url=https%3A%2F%2Fcommons.wikimedia.org%2Fwiki%2FFile%3APerson_icon_(based_on_Noun-Project-3797130).svg&data=05%7C01%7Ckalthoff%40jhu.edu%7C656783b3d71d45ab117108dba285a9ec%7C9fa4f438b1e6473b803f86f8aedf0dec%7C0%7C0%7C638282467191203592%7CUnknown%7CTWFpbGZsb3d8eyJWIjoiMC4wLjAwMDAiLCJQIjoiV2luMzIiLCJBTiI6Ik1haWwiLCJXVCI6Mn0%3D%7C3000%7C%7C%7C&sdata=DyVZf%2FeehNuwsF0uEjzTuy40%2Bs4BQbHUuGSlAh8U5sQ%3D&reserved=0)). The source of the image is specified in the footnote as directed by Olena Szabo, Journal Assistant, on August 22, 2023. We believe the figure is now copyright compatible.

**Response to reviewers sent July 5, 2023**

**Reviewer #1:** Thanks for sharing your manuscript entitled: "The projected prevalence of comorbidities and multimorbidity in people with HIV in the United States through the year 2030". I really enjoy reading your work, I think this kind of modeling efforts are very important to define the potential burden of diseases in a population, and to plan the care and needs of HIV people further than their HIV traditional care, even more if they are focused on groups of specific and different risk.

We appreciate the reviewer’s enthusiasm and understanding of the population-level focus of the modeling output.

I have only minor comments related to the description of the model. I couldn´t understand in the manuscript, how new people incorporates in the follow-up, and neither I could open the link which authors mention that details are (Please double-check the link is really working). I would like to see some lines into the manuscript to understand how is the entrance in the model by group of risk year by year. If this is explained in previous papers, I think readers interested could be informed.

We are sorry about the difficulty that the reviewer experienced with accessing our website. We double checked the link to the methodological details of the model, it works when we click on it (<https://pearlhivmodel.org/method_details.html>). This page provides the methodological details on the “population initiation ART from 2010-2030”, including the procedures used to estimate the annual population sizes, age distribution and CD4 count at ART initiation.

We have clarified how the agents with HIV enter the model when they initiate ART (specifically, in the calendar year of ART initiation from 2010 to 2030) in the second paragraph of the Methods section. To further address the reviewer’s comment, we have added Section S2 to the Supplement, which details the approach to estimating the population size of people with HIV initiating ART.

I would also like to know why ART regimen was not incorporated in some of the comorbidities´ incidence estimations? Is not a current potential association of ART with some of the variables involved, such as changes in BMI?

We agree with the reviewer about the potential impact of ART regimen on comorbidity outcomes. However, we did not have enough data within all 15 subgroups to explicitly integrate ART regimen into the current version of the PEARL model. While the mathematical functions predicting comorbidity incidence do not explicitly include ART regimens, they are estimated among people who initiated ART in the NA-ACCORD from 2010-2017, and as such, potential associations are implicitly impacting the frequency of outcomes. In response to reviewer’s comment, we have added this limitation to the Discussion:

*“ART regimen type is not explicitly contained in the PEARL model, but the impact of various ART regimens on comorbidity incidence is implicitly contained in the mathematical functions from observed NA-ACCORD data.”*

Each risk group has their own probabilities of ART disengagement and re-engagement?

Correct, each subgroup has their own predicted probabilities of disengagement *(see Table 9 at* [*https://pearlhivmodel.org/method_details.html*](https://pearlhivmodel.org/method_details.html) *for more details*).

The sample size observed to re-engage (after disengagement) in the NA-ACCORD was insufficient in many of the 15 subgroups; therefore, we aggregated all sub-populations and assessed at the number of years spent out of care, estimated between 1 to 7 years. The probability of spending a certain number of years out of care was estimated via a normalized Poisson distribution such that the probability of staying disengaged for more than 7 years was zero (*see Figure 7 at* [*https://pearlhivmodel.org/method_details.html*](https://pearlhivmodel.org/method_details.html) *for more details*). Upon disengagement, we apply this distribution to generate the number of years that a simulated person will spend off ART before reengaging back with treatment.

In response to this comment, we have added the following description to the second paragraph of the Methods section:

*“All mathematical functions and parameters in PEARL are estimated separately for each of the 15 subgroups, or collapsed if a priori specifications of minimum sample size or number of events were not met (collapsing race/ethnicity groups first, followed by sex and then HIV-acquisition risk until the minimum is met).”*

Additionally, I´m curious about if this model could be used to predict the comorbidities by group by US state, Is this possible? Or the model calibration and other details to program it don´t allow it? I think this kind a state stratification could be useful for local policy makers and that other social factors associated with local resources, education level or income could be incorporated in the estimations. Is this an issue that authors could comment/discuss into their work? New potential policies to have a better care for some of the populations: those avoiding racism in black and Hispanic population, or those addressed on drug users, could have any impact on your estimations? How big could be this impact?

The PEARL model is well suited to address potential questions concerning the projected prevalence of comorbidities and multimorbidity among 15-subgroups of PLWH at a “national-level”, but as of now, it does not project outcomes by geography.

We agree with the reviewer on the importance of expanding our projections to local settings. Currently, we are conversing with Dr. James Tesoriero, Director of the Center for Program Development, Implementation, Research, and Evaluation at the New York State Department of Health AIDS Institute, and his team to review modeling assumptions needing further revisions to make simulations outputs applicable to the New York state. This will likely include a wide array of assumptions and parameters specific to population size, demographic characteristic, and ART initiation among people with HIV in the state of New York within the 15 subgroups. Potential assumptions regarding individual’s socioeconomic status can influence several parameters in our model (e.g., lower rates of engagement in care among certain vulnerable populations). Other efforts to prevent new infections (via PrEP or community outreach program for different groups) can be further impact the future projections of HIV incidence, and subsequently the population size and age of those initiating ART.

In response to reviewer’s comment, we have added this limitation to the Discussion section:

*“PEARL model forecasts are currently at the national-level, and we are examining the availability of data to forecast at the state-level.”*

I think authors could also mention some advantages of the agent-based models compared to other type of models in the discussion.

We have added a sentence to the last paragraph in the Discussion:

*“Agent-based simulation models are recommended for chronic disease forecasting because they capture the complex interactions among individual-level risk factors that determine the risk of comorbidities and mortality as well as feedback loops needed for disengagement and re-engagement in care.”*

**Reviewer #2:** This very interesting study aims to predict the rates of co-morbidities in different groups of US PLHIV.

Abstract and title are confusing because they do not indicate that this is a modeling study.

We have added “*A modeling study”* to the end of the title.

In the abstract: “***Design:*** *Agent-based simulation model”*

The Introduction discusses the criticality of SDoH, but as-written, it is not clear which SDoH are included in the model. Please incorporate these into Figure 1. For broader introduction, please also define SDoH and list which are the most important determinants for the populations modeled.

We have added the following to the second paragraph of the Introduction:

*“Key SDoH include race, ethnicity, sex, and HIV acquisition risk group.”*

Social determinants of health are included in the model by building the model as the sum of the experiences of 15 subgroups defined by the key SDoH. We have added the following to the first paragraph of the Methods section to clarify:

*“The ProjEcting Age, multimoRbidity, and poLypharmacy (PEARL) model is an agent-based computer simulation model of 15 subgroups of PWH who have initiated ART in the US, including those who disengage from HIV care (****Figure 1****). The 15 subgroups are defined at the intersections of key social determinants of health: (1 & 2) men and women with history of injection drug use as an HIV acquisition risk factor, including those who have injection drug use and any additional HIV acquisition risk category specified (MWID and WWID respectively); (3) men who have sex with men (MSM); and (4 & 5) heterosexual men and women. These five groups were further stratified into non-Hispanic White, non-Hispanic Black/AA, and Hispanic.”*

The last sentence of paragraph 2 of the Intro is difficult to parse: "Multimorbidity is a function of the comorbidities included in the definition and estimates among PWH in the US have a wide range." There seem to be two different points being made here. Make them separately and in sufficient detail, i.e., one sentence explaining the definition of multimorbidity, and the other stating the ranges on multimorbidity estimates from prior studies.

The definition of multimorbidity is in the second to last sentence of the second paragraph in the Introduction:

*“The disproportionate burdens of comorbidities and subsequent multimorbidity (i.e., ≥2 comorbidities not including HIV) pose persistent challenges in ensuring adequate healthcare for people with HIV.”*

We have modified the last sentence of the paragraph to clarify that the wide-ranging estimates of multimorbidity in PWH is due in part to the differences in comorbidities include in the definition of multimorbidity across studies:

*“Estimates among PWH in the US have ranged from 8.2% in 2000 to 65% in 2010-2011; the variability is in part due to modification to the definition of multimorbidity and differences in comorbidities included.^24-26^”*

Is it necessary to subdivide factors correlated with but not caused by HIV (such as smoking an injecting drugs), versus ones caused by HIV (cancer and diseases associated with chronic immune activation)?

Both correlated factors and causal factors related to the comorbidity outcomes are necessary for accurate probability estimates; sub-dividing factors correlated vs. caused (in part) by HIV within the model is not necessary.

The model appears to really be fifteen different models for each of 5 HIV transmission group (MSM, MWID, WWID, heterosexual men, heterosexual women) X 3 racial groups (white, black/AA, Hispanic). It does not consider that individuals can be multiracial or have overlapping risks. Does it include transmission between racial groups and risk groups? It is important to clarify these limitations and think about whether the results are still valid subject to these limitations.

The reviewer’s understanding is correct. PEARL includes 15 subgroups of sex, race and HIV acquisition risk, but does not consider individuals who may be multiracial or those with overlapping risks.

We do not simulate HIV transmissions within the PEARL model. The population size of persons starting ART in each calendar year is estimated from CDC HIV Surveillance data on number of new HIV diagnosis and the rate of linkage to care and ART initiation (as presented in *Supplement Section 2*). These limitations have been discussed in previous publications of the PEARL model, and we have now added them to the last paragraph of the Discussion section:

*“PEARL includes 15 subgroups defined by sex, HIV acquisition risk groups and race/ethnicity, however, it does not represent individuals who are multiracial or those with overlapping risks.”*

*“The PEARL model does not include HIV transmission. The forecasted annual number of new ART initiators relies on CDC's reported HIV diagnoses and linkage to care during the calibration period and does not consider changes in HIV transmission dynamics after the calibration period, including changes due to HIV prevention efforts (e.g., pre-exposure prophylaxis or PrEP) or during the COVID-19 pandemic (e.g., 2020-2021).”*

To the reviewer’s point, we are interested to understand how changes in HIV incidence impact the forecasted age distributions, comorbidity prevalence, and multimorbidity prevalence over the next decade. We are currently working with a modeling team to link their forecasted number of new HIV diagnosis as PEARL input parameters. This would allow us to toggle between two sources of estimated new HIV diagnosis: the observed CDC HIV surveillance data and model-generated data from various scenarios involving *Ending the HIV Epidemic* efforts. This work is ongoing.

The model appears to only include people from the time they first initiate ART. How is the rate of people newly initiating ART (i.e., new individuals entering the model) determined? Presumably this depends on future ART coverage as well as future rates of new HIV infections which give rise to people never before on ART - and HIV infections in turn depend on the populations with unsuppressed HIV viral load participating in different forms of behavioral risk - how is all of this handled?

The approach to estimating the number of new ART-initiators relies on CDC HIV Surveillance data of new HIV diagnoses, CDC estimates of receipt of HIV care 1-to-3 months after HIV diagnosis, and HIV treatment guideline estimates that estimates 97% of people with HIV are eligible to initiate ART at presentation for HIV care. The PEARL model includes the assumption that trends in ART initiation from 2010-2017 persist to 2030; the potential impact of emerging care technologies like oral or long-acting PrEP and ART, or potential changes in HIV transmission dynamics due to COVID-19 is unaccounted for.

We added a new section to the Supplement (Section S2) to detail the methodology for estimating the number of new ART initiators entering the PEARL model, and have expanded the Discussion on limitations of the PEARL model due to lack a transmission engine:

*“The PEARL model does not include HIV transmission. The forecasted annual number of new ART initiators relies on CDC's reported HIV diagnoses and linkage to care during the calibration period and does not consider changes in HIV transmission dynamics after the calibration period, including changes due to HIV prevention efforts (e.g., pre-exposure prophylaxis or PrEP) or during the COVID-19 pandemic (e.g., 2020-2021). Similarly, the calibration of mathematical functions in PEARL is based on NA-ACCORD data available during the calibration period. The PEARL model assumes that the trends observed during the calibration period will continue and does not account for influences occurring outside the calibration period (e.g., the impact of emerging care technologies like long-acting ART or the impact of COVID-19 on mortality); such factors influencing comorbidities incidence can be incorporated into PEARL by extending the calibration period when there are more current data available within the 15 subgroups.”*

Drug use and risk of drug overdose is a large driver of morbidity and mortality, in PWID, constituting six of the fifteen populations being modeled. How can the model accurately capture life expectancy without accounting for drug use morbidities and risk of overdose?

The reviewer raises an astute point that we have examined closely in another study under review that dives deeper into how the subgroup specific mortality rates used in PEARL are essential for predicting the future age distributions and size of the population aging with HIV through the year 2030.

Because mortality rates are subgroup specific, the higher mortality rate of people who use drugs is reflected in the mortality rates used to create the experience of the PEARL agents who are classified as people who use drugs. We have re-written the second paragraph of the Methods section to clarify this point:

*“The agents are followed after ART initiation and observed to experience aging, disengagement and re-engagement in care, changing CD4 counts, risk factors, comorbidities, and mortality via mathematical functions and parameters from the NA-ACCORD and CDC data. The mathematical functions forecast the agents’ experience in the future after observed data end. All mathematical functions and parameters in PEARL are estimated separately for each of the 15 subgroups, or collapsed if a priori specifications of minimum sample size or number of events were not met (collapsing race/ethnicity groups first, followed by sex and then HIV-acquisition risk until the minimum is met).”*

Additionally, the engaged and dis-engaged-specific mortality for the subgroups is in *Supplemental Table S3*, which we refer to in the “*Comorbidities parameterizations*” sub-section of the Methods:

*“Finally, mortality was estimated for simulated persons as a function of individual-level attributes, existing risk factors, and present comorbidities, and was estimated separately for those (1) engaged and (2) disengaged from care (≥2 years without CD4 or HIV RNA measurement) and in each of the 15 subgroups using observed NA-ACCORD data from 2009-2017 (Supplement Table S3).”*

The calibration plots in Supplementary Appendix are an excellent component of this study and very well-presented. There don't appear to be any incidence plots where the model fails to match the data, but there are a number of prevalence plots where the model fails to match the data. How can this be? Is it a problem with the initial conditions?

Thank you for reviewing the Supplementary Appendix. The prevalence of certain comorbidities that do not fit tightly to the observed NA-ACCORD data are noted with orange shading in *Supplemental Figure S2*. This less than desirable fit occurs among the Hispanic subgroups, white heterosexual men, and Black/AA and White women who injected drugs. These groups have smaller observed sample sizes in NA-ACCORD and were combined with other subgroups resulting in differences between the NA-ACCORD observed estimates and PEARL estimates using the *a priori* collapsing strategy of collapsing first by race/ethnicity and second by gender; we have not needed to collapse HIV transmission risk groups. This information is included in the title and footnotes of *Supplemental Figure S2.*

The model estimates the incidence of comorbidities (e.g., depression) as a function of calendar year, age, CD4 count at ART initiation, and time since ART initiation. These are shown as betas from a regression. Does that assume that incidence of comorbidities must always be a linear function of age? Many comorbidities have an age of maximum or minimum incidence (hump-shaped of bowl-shaped function of age) and many others have low incidence until older age (aging-related conditions like HTN, diabetes…) How are these common age patterns taken into account?

The reviewer is correct that age is an independent variable in the model estimating the probability of comorbidity incidence; the relationship is assumed to be linear (i.e., the change in probability per unit increase in age). We agree with the reviewer that the relationship between age and comorbidities are not always necessarily linear. When selecting the mathematical functions estimating the probability of the incident comorbidity, we modified the parameterization of age (using splines and other approaches) and found no appreciable benefit in the fit of the function. Conversely, the functions estimating the probabilities of HIV mortality while on ART and while disengaged from care that included cubic splines for age were superior in fit and were selected for PEARL. As the data from the NA-ACCORD more substantially reflects these nonlinear relationships with age, the updates to the PEARL model functions will incorporate the best fitting functions.

Why did Black WWID have the oldest median age in 2030? One would think their life expectancy would be shorter than heterosexual women due to many health risks and comorbidities. Could this possibly be an error in the model? If so, this could explain why WWID are predicted to have so many co-morbidities compared to other groups (they are simply older and risks of many chronic health conditions grow with age).

Survivor bias is inherent in PWH who reach older ages and may be strongest among those of older age in the subgroups that have the greatest threats to mortality. Black women classified with injection drug use as their HIV acquisition risk who survive to older ages are likely to have survivor bias and are more likely to accumulate age-related comorbidities with increasing age (as the reviewer suggests). Given that Black WWID agents in PEARL are populated with CDC HIV Surveillance estimates of new diagnoses and NA-ACCORD estimates specific to this subgroup, and experience mortality rates estimated among those who injected drugs as their HIV acquisition group, we believe these estimates are accurate. The same pattern was presented in the prior PEARL study of the forecasted age distributions of subgroups (Althoff KN et al., AIDS, 2022. doi: 10.1097/QAD.0000000000003128). We have included this point in the Discussion:

*“Subgroup stratification is essential due to the heterogeneity of PWH. For example, Black/AA WWID are forecasted to have the oldest age distribution in 2030, which is likely a function of gender differences in life expectancy, a survivor effect of these women who had injected drugs during the period they acquired HIV but could be in recovery from substance use disorder, and the national reduction in new HIV infections (which typically happen in younger adults) among people who use injection drugs.^47,48^”*

Could the authors provide calibration graphs showing how their modeled population age structure compares to observed age structure of the 15 groups in different years? I wonder if discrepancies there are contributing to the unintuitive median age findings in the model outputs.

The comparison of the age distributions estimated by PEARL and observed in the NA-ACCORD were presented for the years of 2010-2017 in the Supplement of Althoff KN et al., *AIDS*, 2022 (doi: 10.1097/QAD.00000000000031280) and are on the following pages:

Table S1: Comparing the age distributions of ART-users in PEARL to the observed data from NA-ACCORD, from 2010–2017 (Simulation validation “out-of-sample” approach). Values represent the difference in the age distribution in each subgroup [NA-ACCORD estimate - PEARL estimate]. A threshold of 5 percentage points (>5% or <-5%) is used to detect significant differences (highlighted in blue).

|  | **2010** | **2011** | **2012** | **2013** | **2014** | **2015** | **2016** | **2017** |
| --- | --- | --- | --- | --- | --- | --- | --- | --- |
| **White MSM** |  |  |  |  |  |  |  |  |
|  |  |  |  |  |  |  |  |  |
| <30 years | 0.7% | 0.8% | 0.6% | 0.8% | 0.9% | 0.6% | 0.7% | 0.8% |
|  | [4.9% vs. 4.2%] | [5.4% vs. 4.6%] | [5.7% vs. 5.1%] | [6.1% vs. 5.4%] | [6.4% vs. 5.5%] | [6.1% vs. 5.5%] | [6.1% vs. 5.4%] | [6.1% vs. 5.3%] |
| 30-39 years | -1.2% | -0.4% | 0.4% | 0.8% | 1.1% | 1.2% | 1.4% | 2.6% |
|  | [14.8% vs. 16.0%] | [14.2% vs. 14.7%] | [14.3% vs. 13.8%] | [14.1% vs. 13.3%] | [14.1% vs. 13.0%] | [14.0% vs. 12.8%] | [14.2% vs. 12.8%] | [15.5% vs. 13.0%] |
| 40-49 years | -0.9% | -0.7% | -1.5% | -1.8% | -2.5% | -2.4% | -2.1% | -1.4% |
|  | [41.0% vs. 41.9%] | [38.4% vs. 39.1%] | [34.7% vs. 36.2%] | [31.4% vs. 33.3%] | [28.0% vs. 30.5%] | [25.6% vs. 28.0%] | [23.4% vs. 25.6%] | [22.1% vs. 23.5%] |
| 50-59 years | 1.2% | 0.6% | 0.6% | 0.3% | 0.3% | 0.5% | -0.7% | -0.2% |
|  | [29.3% vs. 28.2%] | [31.1% vs. 30.6%] | [33.2% vs. 32.6%] | [34.7% vs. 34.4%] | [36.1% vs. 35.9%] | [37.5% vs. 37.0%] | [37.0% vs. 37.7%] | [37.7% vs. 37.9%] |
| 60-69 years | -0.1% | -0.5% | -0.3% | 0.0% | 0.3% | 0.2% | 0.7% | -1.1% |
|  | [8.4% vs. 8.5%] | [9.1% vs. 9.6%] | [10.2% vs. 10.5%] | [11.5% vs. 11.6%] | [12.9% vs. 12.6%] | [14.0% vs. 13.8%] | [15.8% vs. 15.1%] | [15.3% vs. 16.4%] |
| ≥70 years | 0.3% | 0.2% | 0.1% | 0.0% | 0.0% | -0.1% | 0.0% | -0.7% |
|  | [1.6% vs. 1.3%] | [1.7% vs. 1.5%] | [1.9% vs. 1.8%] | [2.2% vs. 2.1%] | [2.5% vs. 2.5%] | [2.8% vs. 2.9%] | [3.3% vs. 3.4%] | [3.2% vs. 3.9%] |
| **Black/AA MSM** |  |  |  |  |  |  |  |  |
|  |  |  |  |  |  |  |  |  |
| <30 years | 0.8% | 1.8% | 1.9% | 2.6% | 3.7% | 3.7% | 4.0% | 4.4% |
|  | [19.4% vs. 18.6%] | [21.8% vs. 20.0%] | [23.3% vs. 21.3%] | [24.4% vs. 21.8%] | [25.4% vs. 21.8%] | [25.0% vs. 21.3%] | [24.7% vs. 20.6%] | [24.2% vs. 19.8%] |
| 30-39 years | -1.5% | -0.8% | 0.5% | 1.1% | 2.1% | 2.9% | 0.8% | 1.7% |
|  | [21.2% vs. 22.8%] | [20.7% vs. 21.5%] | [21.6% vs. 21.1%] | [22.4% vs. 21.2%] | [24.0% vs. 21.9%] | [26.0% vs. 23.0%] | [25.2% vs. 24.5%] | [27.8% vs. 26.1%] |
| 40-49 years | 1.9% | 0.5% | -0.6% | -2.1% | -4.2% | -4.9% | -4.9% | -4.7% |
|  | [36.7% vs. 34.8%] | [33.5% vs. 33.1%] | [30.6% vs. 31.2%] | [27.3% vs. 29.4%] | [23.3% vs. 27.6%] | [20.9% vs. 25.8%] | [19.2% vs. 24.0%] | [17.6% vs. 22.4%] |
| 50-59 years | -1.8% | -1.5% | -1.6% | -1.2% | -0.7% | -0.3% | 0.9% | 0.3% |
|  | [18.1% vs. 19.9%] | [19.2% vs. 20.8%] | [19.7% vs. 21.3%] | [20.6% vs. 21.8%] | [21.5% vs. 22.2%] | [22.2% vs. 22.5%] | [23.6% vs. 22.7%] | [23.0% vs. 22.7%] |
| 60-69 years | 0.3% | -0.2% | -0.6% | -0.8% | -1.2% | -1.6% | -1.0% | -1.7% |
|  | [4.0% vs. 3.7%] | [4.1% vs. 4.3%] | [4.2% vs. 4.8%] | [4.6% vs. 5.4%] | [4.8% vs. 6.0%] | [5.1% vs. 6.7%] | [6.4% vs. 7.4%] | [6.4% vs. 8.1%] |
| ≥70 years | 0.3% | 0.3% | 0.4% | 0.4% | 0.4% | 0.1% | 0.3% | 0.0% |
|  | [0.6% vs. 0.2%] | [0.6% vs. 0.3%] | [0.7% vs. 0.4%] | [0.8% vs. 0.4%] | [1.0% vs. 0.5%] | [0.8% vs. 0.7%] | [1.1% vs. 0.8%] | [0.9% vs. 1.0%] |
| **Hispanic MSM** |  |  |  |  |  |  |  |  |
|  |  |  |  |  |  |  |  |  |
| <30 years | 1.5% | 1.4% | 1.1% | 1.6% | 1.0% | 0.3% | -0.4% | -0.3% |
|  | [11.5% vs. 10.0%] | [12.1% vs. 10.7%] | [12.7% vs. 11.6%] | [13.7% vs. 12.1%] | [13.4% vs. 12.4%] | [12.7% vs. 12.5%] | [12.1% vs. 12.5%] | [12.1% vs. 12.4%] |
| 30-39 years | -4.2% | -1.8% | -0.8% | -0.9% | 0.8% | 2.0% | 2.3% | 2.6% |
|  | [28.1% vs. 32.3%] | [27.9% vs. 29.6%] | [26.9% vs. 27.7%] | [25.4% vs. 26.3%] | [26.2% vs. 25.4%] | [26.9% vs. 24.9%] | [27.3% vs. 24.9%] | [27.9% vs. 25.4%] |
| 40-49 years | 3.4% | 1.7% | 0.9% | -0.3% | -2.4% | -3.3% | -4.0% | -2.5% |
|  | [42.3% vs. 38.9%] | [40.8% vs. 39.0%] | [39.5% vs. 38.6%] | [37.6% vs. 37.9%] | [34.5% vs. 36.9%] | [32.4% vs. 35.7%] | [30.2% vs. 34.2%] | [30.0% vs. 32.5%] |
| 50-59 years | -1.2% | -1.5% | -0.8% | -0.3% | 0.3% | 0.8% | 1.4% | 0.7% |
|  | [14.0% vs. 15.2%] | [15.3% vs. 16.8%] | [17.2% vs. 18.0%] | [19.0% vs. 19.3%] | [20.8% vs. 20.5%] | [22.4% vs. 21.6%] | [23.9% vs. 22.5%] | [24.0% vs. 23.3%] |
| 60-69 years | 0.6% | 0.4% | -0.3% | -0.1% | 0.4% | 0.4% | 0.5% | -0.6% |
|  | [3.3% vs. 2.7%] | [3.4% vs. 3.0%] | [2.9% vs. 3.3%] | [3.5% vs. 3.6%] | [4.4% vs. 4.0%] | [4.8% vs. 4.4%] | [5.4% vs. 4.9%] | [4.8% vs. 5.5%] |
| ≥70 years | -0.1% | -0.3% | -0.1% | -0.1% | -0.1% | -0.1% | 0.2% | 0.1% |
|  | [0.7% vs. 0.9%] | [0.6% vs. 0.9%] | [0.7% vs. 0.9%] | [0.7% vs. 0.9%] | [0.7% vs. 0.9%] | [0.8% vs. 0.9%] | [1.2% vs. 0.9%] | [1.1% vs. 1.0%] |
| **White IDU men** |  |  |  |  |  |  |  |  |
|  |  |  |  |  |  |  |  |  |
| <30 years | 0.2% | 0.1% | 0.4% | 0.6% | 0.6% | 0.3% | -0.2% | -0.2% |
|  | [2.2% vs. 2.0%] | [2.1% vs. 2.0%] | [2.6% vs. 2.2%] | [2.9% vs. 2.3%] | [3.1% vs. 2.5%] | [3.1% vs. 2.7%] | [2.9% vs. 3.1%] | [3.3% vs. 3.5%] |
| 30-39 years | 0.0% | -0.4% | -0.4% | -0.2% | 0.0% | -0.4% | 3.2% | 1.3% |
|  | [10.6% vs. 10.6%] | [9.3% vs. 9.7%] | [8.9% vs. 9.3%] | [8.9% vs. 9.1%] | [9.2% vs. 9.2%] | [8.9% vs. 9.4%] | [12.9% vs. 9.7%] | [11.5% vs. 10.2%] |
| 40-49 years | -1.1% | -0.5% | -0.9% | -2.0% | -3.2% | -2.7% | 4.0% | 5.5% |
|  | [36.0% vs. 37.1%] | [34.1% vs. 34.6%] | [31.2% vs. 32.1%] | [27.6% vs. 29.6%] | [24.0% vs. 27.2%] | [22.0% vs. 24.7%] | [26.3% vs. 22.3%] | [25.6% vs. 20.1%] |
| 50-59 years | 0.1% | 0.7% | 0.5% | 0.4% | 1.1% | 0.5% | 0.2% | 1.4% |
|  | [38.5% vs. 38.4%] | [40.5% vs. 39.8%] | [41.0% vs. 40.5%] | [41.3% vs. 40.9%] | [42.2% vs. 41.1%] | [41.3% vs. 40.9%] | [40.5% vs. 40.3%] | [40.8% vs. 39.4%] |
| 60-69 years | 0.7% | 0.1% | 0.4% | 1.3% | 1.6% | 2.2% | -5.6% | -5.7% |
|  | [11.7% vs. 11.0%] | [12.9% vs. 12.8%] | [14.8% vs. 14.5%] | [17.4% vs. 16.1%] | [19.4% vs. 17.8%] | [21.7% vs. 19.5%] | [15.5% vs. 21.1%] | [17.0% vs. 22.7%] |
| ≥70 years | 0.1% | 0.0% | 0.0% | 0.0% | -0.1% | 0.1% | -1.6% | -2.3% |
|  | [0.9% vs. 0.9%] | [1.1% vs. 1.2%] | [1.5% vs. 1.5%] | [1.8% vs. 1.9%] | [2.2% vs. 2.3%] | [2.9% vs. 2.8%] | [1.8% vs. 3.4%] | [1.8% vs. 4.1%] |
| **Black/AA IDU men** |  |  |  |  |  |  |  |  |
|  |  |  |  |  |  |  |  |  |
| <30 years | 0.1% | 0.1% | -0.1% | -0.3% | -0.4% | -0.5% | 4.5% | 7.2% |
|  | [0.6% vs. 0.5%] | [0.7% vs. 0.6%] | [0.7% vs. 0.8%] | [0.8% vs. 1.1%] | [1.1% vs. 1.5%] | [1.5% vs. 2.0%] | [7.1% vs. 2.6%] | [10.6% vs. 3.4%] |
| 30-39 years | 0.1% | 0.0% | -0.3% | -0.5% | -0.8% | -1.2% | 3.2% | 5.6% |
|  | [2.6% vs. 2.5%] | [2.6% vs. 2.5%] | [2.5% vs. 2.8%] | [2.7% vs. 3.1%] | [2.7% vs. 3.5%] | [2.6% vs. 3.8%] | [7.3% vs. 4.1%] | [9.9% vs. 4.3%] |
| 40-49 years | -1.3% | -1.1% | -1.0% | -1.2% | -1.2% | -0.8% | 3.4% | 3.2% |
|  | [20.4% vs. 21.7%] | [17.1% vs. 18.2%] | [14.3% vs. 15.3%] | [11.7% vs. 12.9%] | [9.7% vs. 10.9%] | [8.5% vs. 9.3%] | [11.5% vs. 8.1%] | [10.5% vs. 7.2%] |
| 50-59 years | 0.6% | 0.6% | 0.7% | 1.0% | 0.6% | -1.0% | -0.5% | -2.6% |
|  | [56.1% vs. 55.4%] | [55.8% vs. 55.2%] | [54.6% vs. 53.8%] | [52.7% vs. 51.6%] | [49.3% vs. 48.7%] | [44.2% vs. 45.3%] | [40.9% vs. 41.4%] | [34.6% vs. 37.3%] |
| 60-69 years | 0.0% | 0.0% | 0.6% | 0.9% | 1.9% | 3.8% | -9.1% | -10.1% |
|  | [19.1% vs. 19.1%] | [22.4% vs. 22.4%] | [26.3% vs. 25.7%] | [30.1% vs. 29.2%] | [34.5% vs. 32.6%] | [39.7% vs. 35.9%] | [29.8% vs. 38.9%] | [31.4% vs. 41.5%] |
| ≥70 years | 0.4% | 0.4% | 0.1% | 0.0% | -0.2% | -0.2% | -1.5% | -3.3% |
|  | [1.3% vs. 0.8%] | [1.5% vs. 1.1%] | [1.7% vs. 1.6%] | [2.1% vs. 2.1%] | [2.6% vs. 2.9%] | [3.6% vs. 3.8%] | [3.4% vs. 4.8%] | [2.9% vs. 6.2%] |
| **Hispanic IDU men** |  |  |  |  |  |  |  |  |
|  |  |  |  |  |  |  |  |  |
| <30 years | 0.1% | -0.6% | -0.2% | 0.2% | -0.2% | -0.1% | 3.1% | 3.6% |
|  | [1.5% vs. 1.4%] | [1.0% vs. 1.6%] | [1.7% vs. 1.9%] | [2.3% vs. 2.1%] | [2.2% vs. 2.4%] | [2.5% vs. 2.6%] | [6.0% vs. 2.9%] | [6.8% vs. 3.2%] |
| 30-39 years | -1.2% | -0.2% | 0.6% | 0.3% | 0.5% | 0.8% | 10.7% | 12.3% |
|  | [9.8% vs. 11.0%] | [10.3% vs. 10.5%] | [10.8% vs. 10.1%] | [10.0% vs. 9.8%] | [10.1% vs. 9.5%] | [10.2% vs. 9.4%] | [20.1% vs. 9.4%] | [21.8% vs. 9.5%] |
| 40-49 years | 0.4% | -0.1% | -0.8% | -2.0% | -2.1% | -0.8% | 17.3% | 13.0% |
|  | [27.5% vs. 27.2%] | [24.9% vs. 25.0%] | [22.7% vs. 23.4%] | [20.2% vs. 22.2%] | [19.1% vs. 21.2%] | [19.6% vs. 20.4%] | [37.0% vs. 19.6%] | [32.0% vs. 19.0%] |
| 50-59 years | -0.1% | -0.8% | -1.0% | -0.7% | -0.4% | -5.8% | -10.4% | -5.6% |
|  | [45.9% vs. 46.0%] | [44.7% vs. 45.5%] | [43.3% vs. 44.3%] | [42.0% vs. 42.7%] | [40.5% vs. 40.9%] | [33.0% vs. 38.8%] | [26.1% vs. 36.5%] | [28.6% vs. 34.2%] |
| 60-69 years | 0.5% | 1.3% | 0.9% | 2.1% | 2.3% | 6.0% | -19.1% | -20.6% |
|  | [14.3% vs. 13.8%] | [17.8% vs. 16.5%] | [19.9% vs. 19.0%] | [23.5% vs. 21.4%] | [26.0% vs. 23.7%] | [31.8% vs. 25.8%] | [8.7% vs. 27.8%] | [8.8% vs. 29.4%] |
| ≥70 years | 0.3% | 0.3% | 0.5% | 0.3% | -0.1% | 0.0% | -1.6% | -2.7% |
|  | [0.9% vs. 0.6%] | [1.2% vs. 0.9%] | [1.7% vs. 1.2%] | [1.9% vs. 1.7%] | [2.2% vs. 2.3%] | [2.9% vs. 2.9%] | [2.2% vs. 3.8%] | [2.0% vs. 4.8%] |
| **White IDU women** |  |  |  |  |  |  |  |  |
|  |  |  |  |  |  |  |  |  |
| <30 years | 0.6% | 1.6% | 0.7% | -0.4% | 0.3% | 0.8% | 2.1% | 0.6% |
|  | [3.9% vs. 3.3%] | [4.4% vs. 2.8%] | [3.3% vs. 2.6%] | [2.0% vs. 2.4%] | [2.5% vs. 2.2%] | [2.8% vs. 2.1%] | [4.0% vs. 2.0%] | [2.5% vs. 1.9%] |
| 30-39 years | -0.6% | 0.5% | 0.6% | 2.0% | 0.3% | -0.1% | -1.0% | 0.0% |
|  | [21.9% vs. 22.5%] | [20.7% vs. 20.2%] | [19.1% vs. 18.5%] | [19.2% vs. 17.2%] | [16.5% vs. 16.3%] | [15.4% vs. 15.4%] | [13.8% vs. 14.8%] | [14.2% vs. 14.2%] |
| 40-49 years | 0.6% | -1.8% | -2.3% | -2.0% | -2.6% | -2.0% | 2.3% | 3.0% |
|  | [41.9% vs. 41.2%] | [38.2% vs. 40.0%] | [36.0% vs. 38.3%] | [34.5% vs. 36.5%] | [31.9% vs. 34.5%] | [30.8% vs. 32.7%] | [33.3% vs. 31.0%] | [32.7% vs. 29.7%] |
| 50-59 years | 0.6% | 0.9% | 2.2% | 1.1% | 3.0% | 3.1% | -0.9% | -3.5% |
|  | [28.7% vs. 28.1%] | [31.7% vs. 30.9%] | [35.4% vs. 33.2%] | [36.0% vs. 34.9%] | [39.2% vs. 36.3%] | [40.3% vs. 37.2%] | [36.7% vs. 37.6%] | [34.2% vs. 37.6%] |
| 60-69 years | -1.6% | -1.1% | -0.9% | -0.3% | -0.2% | -0.9% | -1.6% | 0.9% |
|  | [3.1% vs. 4.7%] | [4.8% vs. 5.9%] | [6.3% vs. 7.2%] | [8.3% vs. 8.6%] | [9.9% vs. 10.1%] | [10.8% vs. 11.7%] | [11.8% vs. 13.4%] | [16.0% vs. 15.1%] |
| ≥70 years | 0.4% | -0.1% | -0.3% | -0.5% | -0.7% | -0.9% | -0.8% | -1.1% |
|  | [0.6% vs. 0.2%] | [0.2% vs. 0.3%] | [0.0% vs. 0.3%] | [0.0% vs. 0.5%] | [0.0% vs. 0.7%] | [0.0% vs. 0.9%] | [0.3% vs. 1.2%] | [0.4% vs. 1.5%] |
| **Black/AA IDU women** |  |  |  |  |  |  |  |  |
|  |  |  |  |  |  |  |  |  |
| <30 years | 0.2% | 0.3% | 0.0% | -0.4% | -0.4% | -0.4% | 1.5% | 1.9% |
|  | [0.6% vs. 0.4%] | [0.8% vs. 0.5%] | [0.5% vs. 0.5%] | [0.2% vs. 0.6%] | [0.2% vs. 0.6%] | [0.2% vs. 0.6%] | [2.2% vs. 0.7%] | [2.7% vs. 0.7%] |
| 30-39 years | -0.7% | -0.3% | 0.4% | 1.1% | -0.3% | 0.2% | 1.6% | 1.2% |
|  | [7.1% vs. 7.9%] | [6.0% vs. 6.3%] | [5.7% vs. 5.3%] | [5.8% vs. 4.8%] | [4.1% vs. 4.4%] | [4.4% vs. 4.3%] | [5.7% vs. 4.1%] | [5.3% vs. 4.1%] |
| 40-49 years | 0.4% | -3.1% | -2.9% | -2.7% | -0.8% | -2.6% | 3.8% | 3.6% |
|  | [37.6% vs. 37.1%] | [30.4% vs. 33.5%] | [27.1% vs. 30.0%] | [24.0% vs. 26.8%] | [22.9% vs. 23.7%] | [18.2% vs. 20.8%] | [22.0% vs. 18.2%] | [19.6% vs. 16.0%] |
| 50-59 years | -1.6% | 0.1% | -0.7% | -1.2% | -1.2% | 0.9% | -5.7% | -3.0% |
|  | [47.1% vs. 48.6%] | [51.6% vs. 51.5%] | [52.2% vs. 53.0%] | [52.1% vs. 53.3%] | [51.4% vs. 52.6%] | [52.0% vs. 51.1%] | [43.2% vs. 48.9%] | [43.1% vs. 46.2%] |
| 60-69 years | 1.7% | 2.9% | 2.8% | 2.5% | 2.4% | 1.9% | -1.3% | -3.8% |
|  | [7.4% vs. 5.8%] | [10.9% vs. 8.0%] | [13.6% vs. 10.8%] | [16.7% vs. 14.2%] | [20.4% vs. 18.0%] | [24.2% vs. 22.3%] | [25.6% vs. 26.8%] | [27.6% vs. 31.3%] |
| ≥70 years | 0.0% | 0.1% | 0.6% | 0.8% | 0.4% | 0.0% | 0.1% | 0.0% |
|  | [0.2% vs. 0.1%] | [0.3% vs. 0.2%] | [0.8% vs. 0.3%] | [1.2% vs. 0.4%] | [1.0% vs. 0.6%] | [0.9% vs. 0.9%] | [1.3% vs. 1.2%] | [1.8% vs. 1.8%] |
| **Hispanic IDU women** |  |  |  |  |  |  |  |  |
|  |  |  |  |  |  |  |  |  |
| <30 years | 1.2% | 0.3% | 0.1% | 0.6% | -0.3% | -0.3% | -0.2% | -0.2% |
|  | [3.5% vs. 2.3%] | [1.7% vs. 1.4%] | [1.0% vs. 0.9%] | [1.1% vs. 0.5%] | [0.0% vs. 0.3%] | [0.0% vs. 0.3%] | [0.0% vs. 0.2%] | [0.0% vs. 0.2%] |
| 30-39 years | -0.2% | 1.0% | 1.7% | 2.7% | -0.3% | -2.2% | -0.2% | 10.0% |
|  | [9.6% vs. 9.7%] | [10.4% vs. 9.4%] | [11.0% vs. 9.3%] | [12.0% vs. 9.2%] | [8.8% vs. 9.1%] | [6.7% vs. 8.9%] | [8.3% vs. 8.5%] | [18.2% vs. 8.1%] |
| 40-49 years | -2.6% | -1.7% | -6.0% | -7.2% | -4.1% | -1.3% | 5.9% | 0.6% |
|  | [35.7% vs. 38.3%] | [32.2% vs. 33.8%] | [24.0% vs. 30.0%] | [19.6% vs. 26.8%] | [19.8% vs. 23.8%] | [20.0% vs. 21.3%] | [25.0% vs. 19.1%] | [18.2% vs. 17.6%] |
| 50-59 years | 0.3% | -0.8% | -0.9% | 0.1% | -2.9% | 2.3% | -28.9% | -26.0% |
|  | [41.7% vs. 41.5%] | [43.5% vs. 44.3%] | [45.0% vs. 45.9%] | [46.7% vs. 46.7%] | [44.0% vs. 46.9%] | [48.9% vs. 46.6%] | [16.7% vs. 45.5%] | [18.2% vs. 44.1%] |
| 60-69 years | 0.7% | 0.6% | 4.7% | 2.6% | 6.7% | -1.3% | 9.3% | 0.5% |
|  | [8.7% vs. 8.0%] | [11.3% vs. 10.7%] | [18.0% vs. 13.3%] | [18.5% vs. 15.9%] | [25.3% vs. 18.6%] | [20.0% vs. 21.3%] | [33.3% vs. 24.0%] | [27.3% vs. 26.8%] |
| ≥70 years | 0.6% | 0.5% | 0.4% | 1.2% | 0.9% | 2.7% | 14.2% | 15.0% |
|  | [0.9% vs. 0.2%] | [0.9% vs. 0.4%] | [1.0% vs. 0.6%] | [2.2% vs. 0.9%] | [2.2% vs. 1.3%] | [4.4% vs. 1.8%] | [16.7% vs. 2.4%] | [18.2% vs. 3.2%] |
| **White heterosexual men** |  |  |  |  |  |  |  |  |
|  |  |  |  |  |  |  |  |  |
| <30 years | 0.4% | 0.2% | 0.0% | 1.0% | 1.7% | 1.1% | 0.2% | 0.5% |
|  | [3.0% vs. 2.6%] | [2.7% vs. 2.5%] | [2.5% vs. 2.5%] | [3.6% vs. 2.6%] | [4.4% vs. 2.6%] | [3.8% vs. 2.7%] | [3.0% vs. 2.9%] | [3.5% vs. 3.1%] |
| 30-39 years | -1.0% | -2.7% | -2.3% | -2.7% | -2.8% | -1.7% | -1.0% | -0.3% |
|  | [12.5% vs. 13.5%] | [11.1% vs. 13.8%] | [11.6% vs. 14.0%] | [11.2% vs. 13.8%] | [10.7% vs. 13.5%] | [11.2% vs. 12.9%] | [11.3% vs. 12.4%] | [11.5% vs. 11.8%] |
| 40-49 years | 0.0% | 0.4% | -0.6% | -0.7% | -1.8% | -0.1% | -2.8% | -1.4% |
|  | [37.5% vs. 37.5%] | [35.2% vs. 34.8%] | [31.8% vs. 32.4%] | [29.4% vs. 30.1%] | [26.2% vs. 28.0%] | [26.1% vs. 26.2%] | [21.6% vs. 24.4%] | [21.3% vs. 22.7%] |
| 50-59 years | -0.8% | 0.8% | 2.3% | 1.6% | 2.6% | 0.9% | 0.5% | 1.0% |
|  | [31.0% vs. 31.8%] | [34.1% vs. 33.2%] | [36.7% vs. 34.4%] | [37.1% vs. 35.5%] | [39.1% vs. 36.5%] | [37.9% vs. 37.0%] | [37.6% vs. 37.1%] | [37.8% vs. 36.9%] |
| 60-69 years | 0.5% | 0.5% | -0.1% | -0.4% | -0.2% | -0.2% | 1.6% | -1.1% |
|  | [12.3% vs. 11.8%] | [13.1% vs. 12.7%] | [13.4% vs. 13.5%] | [14.1% vs. 14.5%] | [15.5% vs. 15.6%] | [16.6% vs. 16.9%] | [19.9% vs. 18.3%] | [18.9% vs. 20.0%] |
| ≥70 years | 1.0% | 0.8% | 0.8% | 1.2% | 0.4% | -0.1% | 1.6% | 1.4% |
|  | [3.7% vs. 2.6%] | [3.8% vs. 2.9%] | [3.9% vs. 3.2%] | [4.6% vs. 3.5%] | [4.2% vs. 3.9%] | [4.3% vs. 4.3%] | [6.5% vs. 4.9%] | [6.9% vs. 5.6%] |
| **Black/AA heterosexual men** |  |  |  |  |  |  |  |  |
|  |  |  |  |  |  |  |  |  |
| <30 years | -0.2% | -0.4% | 0.0% | -0.1% | 0.1% | 0.9% | 0.7% | 0.9% |
|  | [3.7% vs. 3.9%] | [3.9% vs. 4.3%] | [4.7% vs. 4.7%] | [4.7% vs. 4.8%] | [4.9% vs. 4.8%] | [5.6% vs. 4.7%] | [5.3% vs. 4.6%] | [5.3% vs. 4.4%] |
| 30-39 years | 1.0% | 1.3% | 1.6% | 1.7% | 1.9% | 2.2% | 1.2% | 1.3% |
|  | [17.1% vs. 16.1%] | [16.0% vs. 14.7%] | [15.3% vs. 13.8%] | [14.8% vs. 13.1%] | [14.5% vs. 12.6%] | [14.5% vs. 12.4%] | [13.4% vs. 12.3%] | [13.6% vs. 12.4%] |
| 40-49 years | -1.5% | -2.0% | -2.8% | -2.6% | -2.3% | -1.4% | 0.4% | 1.6% |
|  | [40.1% vs. 41.6%] | [37.5% vs. 39.5%] | [34.5% vs. 37.2%] | [32.4% vs. 35.0%] | [30.4% vs. 32.7%] | [28.9% vs. 30.4%] | [28.5% vs. 28.1%] | [27.5% vs. 25.9%] |
| 50-59 years | 1.0% | 0.9% | 1.3% | 1.1% | 0.5% | -1.1% | -0.5% | -1.2% |
|  | [30.0% vs. 28.9%] | [31.8% vs. 30.9%] | [33.9% vs. 32.6%] | [35.3% vs. 34.2%] | [36.0% vs. 35.5%] | [35.5% vs. 36.6%] | [36.8% vs. 37.4%] | [36.5% vs. 37.7%] |
| 60-69 years | -1.1% | -0.6% | -0.5% | -0.4% | -0.4% | -0.5% | -1.1% | -1.5% |
|  | [6.8% vs. 7.9%] | [8.2% vs. 8.8%] | [9.2% vs. 9.7%] | [10.4% vs. 10.8%] | [11.5% vs. 11.9%] | [12.8% vs. 13.3%] | [13.6% vs. 14.7%] | [14.8% vs. 16.2%] |
| ≥70 years | 0.7% | 0.8% | 0.5% | 0.3% | 0.2% | -0.1% | -0.7% | -1.1% |
|  | [2.3% vs. 1.6%] | [2.5% vs. 1.8%] | [2.4% vs. 1.9%] | [2.5% vs. 2.2%] | [2.6% vs. 2.4%] | [2.6% vs. 2.7%] | [2.3% vs. 3.0%] | [2.3% vs. 3.4%] |
| **Hispanic heterosexual men** |  |  |  |  |  |  |  |  |
|  |  |  |  |  |  |  |  |  |
| <30 years | -0.1% | -0.6% | -0.2% | -0.2% | 0.1% | 0.5% | -0.6% | -0.9% |
|  | [6.0% vs. 6.0%] | [4.9% vs. 5.5%] | [4.8% vs. 5.0%] | [4.2% vs. 4.4%] | [4.1% vs. 3.9%] | [3.9% vs. 3.4%] | [2.5% vs. 3.1%] | [1.8% vs. 2.8%] |
| 30-39 years | -1.9% | -0.6% | -0.3% | -0.1% | -0.2% | 1.6% | -0.5% | -0.4% |
|  | [24.6% vs. 26.6%] | [23.6% vs. 24.2%] | [22.2% vs. 22.4%] | [20.8% vs. 20.9%] | [19.5% vs. 19.7%] | [20.1% vs. 18.5%] | [17.0% vs. 17.5%] | [16.2% vs. 16.6%] |
| 40-49 years | 0.0% | -1.2% | -2.1% | -1.0% | -1.2% | 0.4% | -1.3% | 1.5% |
|  | [36.7% vs. 36.7%] | [35.7% vs. 36.9%] | [34.3% vs. 36.4%] | [34.5% vs. 35.5%] | [33.1% vs. 34.3%] | [33.4% vs. 33.0%] | [30.5% vs. 31.8%] | [32.4% vs. 30.8%] |
| 50-59 years | 2.3% | 2.7% | 1.9% | 1.2% | 0.3% | -0.3% | 3.3% | 1.0% |
|  | [23.0% vs. 20.7%] | [25.2% vs. 22.5%] | [26.3% vs. 24.3%] | [27.2% vs. 26.1%] | [27.9% vs. 27.6%] | [28.4% vs. 28.7%] | [32.7% vs. 29.4%] | [30.9% vs. 29.9%] |
| 60-69 years | -0.5% | -0.8% | 0.1% | -0.4% | 0.6% | -1.9% | -0.5% | -0.3% |
|  | [7.5% vs. 8.0%] | [7.9% vs. 8.7%] | [9.6% vs. 9.4%] | [10.0% vs. 10.4%] | [12.2% vs. 11.6%] | [11.0% vs. 12.9%] | [13.7% vs. 14.2%] | [15.1% vs. 15.4%] |
| ≥70 years | 0.2% | 0.5% | 0.6% | 0.6% | 0.3% | -0.2% | -0.4% | -0.9% |
|  | [2.2% vs. 2.0%] | [2.7% vs. 2.2%] | [2.9% vs. 2.4%] | [3.3% vs. 2.6%] | [3.3% vs. 3.0%] | [3.2% vs. 3.4%] | [3.6% vs. 3.9%] | [3.7% vs. 4.6%] |
| **White heterosexual women** |  |  |  |  |  |  |  |  |
|  |  |  |  |  |  |  |  |  |
| <30 years | 0.8% | 1.5% | 1.0% | 1.3% | 1.3% | 1.0% | 1.3% | 1.9% |
|  | [6.4% vs. 5.6%] | [7.3% vs. 5.9%] | [7.2% vs. 6.2%] | [7.3% vs. 6.0%] | [6.8% vs. 5.6%] | [5.9% vs. 4.9%] | [5.5% vs. 4.2%] | [5.4% vs. 3.5%] |
| 30-39 years | -2.1% | -3.0% | -0.5% | 0.4% | -0.2% | 0.0% | -1.0% | -0.3% |
|  | [21.6% vs. 23.6%] | [17.7% vs. 20.7%] | [18.2% vs. 18.7%] | [17.9% vs. 17.5%] | [16.6% vs. 16.8%] | [16.3% vs. 16.3%] | [14.9% vs. 15.9%] | [15.2% vs. 15.5%] |
| 40-49 years | -1.3% | -0.2% | -1.2% | -3.0% | -2.4% | -3.1% | -5.2% | -1.6% |
|  | [37.6% vs. 38.9%] | [38.2% vs. 38.4%] | [36.0% vs. 37.2%] | [32.9% vs. 35.8%] | [31.8% vs. 34.2%] | [29.5% vs. 32.6%] | [25.8% vs. 31.0%] | [28.0% vs. 29.6%] |
| 50-59 years | 2.0% | 1.5% | 0.5% | 0.9% | 0.0% | 1.4% | 4.0% | 0.7% |
|  | [25.2% vs. 23.2%] | [26.8% vs. 25.3%] | [27.5% vs. 27.0%] | [29.4% vs. 28.6%] | [30.0% vs. 30.0%] | [32.6% vs. 31.2%] | [36.2% vs. 32.2%] | [33.6% vs. 32.9%] |
| 60-69 years | 0.6% | 0.4% | 0.4% | 0.3% | 0.8% | 0.2% | -0.3% | -1.4% |
|  | [7.6% vs. 7.1%] | [8.3% vs. 8.0%] | [9.2% vs. 8.8%] | [10.1% vs. 9.8%] | [11.6% vs. 10.8%] | [12.2% vs. 12.0%] | [12.9% vs. 13.2%] | [13.2% vs. 14.6%] |
| ≥70 years | 0.0% | -0.1% | -0.2% | 0.1% | 0.5% | 0.5% | 1.3% | 0.8% |
|  | [1.6% vs. 1.6%] | [1.7% vs. 1.9%] | [1.9% vs. 2.1%] | [2.4% vs. 2.3%] | [3.2% vs. 2.7%] | [3.5% vs. 3.0%] | [4.7% vs. 3.4%] | [4.6% vs. 3.8%] |
| **Black/AA heterosexual women** |  |  |  |  |  |  |  |  |
|  |  |  |  |  |  |  |  |  |
| <30 years | 0.0% | -0.1% | 0.2% | 0.3% | 0.4% | 0.5% | 0.3% | 0.9% |
|  | [8.9% vs. 8.8%] | [8.1% vs. 8.2%] | [7.9% vs. 7.8%] | [7.5% vs. 7.2%] | [7.0% vs. 6.6%] | [6.5% vs. 5.9%] | [5.6% vs. 5.2%] | [5.5% vs. 4.6%] |
| 30-39 years | -1.7% | -1.5% | -0.6% | 0.2% | 0.4% | 1.5% | -0.3% | -0.9% |
|  | [25.5% vs. 27.2%] | [23.9% vs. 25.3%] | [23.2% vs. 23.7%] | [22.6% vs. 22.4%] | [21.6% vs. 21.2%] | [21.8% vs. 20.2%] | [19.1% vs. 19.3%] | [17.7% vs. 18.6%] |
| 40-49 years | 1.2% | 1.8% | 0.7% | 0.3% | -0.1% | 0.1% | 1.2% | 0.8% |
|  | [36.5% vs. 35.3%] | [36.9% vs. 35.1%] | [35.3% vs. 34.6%] | [34.4% vs. 34.1%] | [33.3% vs. 33.4%] | [32.8% vs. 32.6%] | [32.9% vs. 31.7%] | [31.6% vs. 30.7%] |
| 50-59 years | 0.3% | -0.5% | -0.7% | -0.6% | -0.4% | -1.8% | -0.9% | 0.2% |
|  | [22.7% vs. 22.4%] | [23.6% vs. 24.1%] | [24.9% vs. 25.6%] | [26.3% vs. 26.8%] | [27.6% vs. 28.0%] | [27.3% vs. 29.1%] | [29.1% vs. 30.0%] | [31.0% vs. 30.8%] |
| 60-69 years | -0.3% | 0.0% | 0.2% | -0.5% | -0.7% | -1.1% | -0.8% | -1.3% |
|  | [5.3% vs. 5.6%] | [6.5% vs. 6.5%] | [7.7% vs. 7.5%] | [8.0% vs. 8.5%] | [8.8% vs. 9.5%] | [9.5% vs. 10.6%] | [11.0% vs. 11.9%] | [11.8% vs. 13.1%] |
| ≥70 years | 0.5% | 0.4% | 0.2% | 0.3% | 0.5% | 0.6% | 0.5% | 0.3% |
|  | [1.1% vs. 0.5%] | [1.1% vs. 0.7%] | [1.0% vs. 0.8%] | [1.3% vs. 1.0%] | [1.7% vs. 1.3%] | [2.2% vs. 1.5%] | [2.3% vs. 1.8%] | [2.5% vs. 2.2%] |
| **Hispanic heterosexual women** |  |  |  |  |  |  |  |  |
|  |  |  |  |  |  |  |  |  |
| <30 years | 0.5% | 0.7% | 0.9% | 0.8% | -0.2% | 0.5% | -1.2% | 1.0% |
|  | [8.7% vs. 8.2%] | [7.7% vs. 7.0%] | [7.0% vs. 6.1%] | [6.1% vs. 5.3%] | [4.6% vs. 4.7%] | [4.8% vs. 4.3%] | [2.9% vs. 4.0%] | [4.8% vs. 3.9%] |
| 30-39 years | -0.3% | -0.5% | -0.8% | -0.7% | 0.1% | 1.3% | 4.2% | 4.1% |
|  | [28.5% vs. 28.8%] | [27.4% vs. 27.9%] | [26.2% vs. 27.0%] | [25.2% vs. 25.9%] | [24.7% vs. 24.7%] | [24.7% vs. 23.4%] | [26.3% vs. 22.1%] | [25.0% vs. 20.9%] |
| 40-49 years | 1.1% | 0.8% | 0.5% | 0.9% | 1.0% | 1.2% | 2.2% | 5.0% |
|  | [34.6% vs. 33.5%] | [33.5% vs. 32.7%] | [32.4% vs. 32.0%] | [32.2% vs. 31.3%] | [31.7% vs. 30.7%] | [31.3% vs. 30.1%] | [31.7% vs. 29.5%] | [33.9% vs. 28.9%] |
| 50-59 years | -2.9% | -2.2% | -1.7% | -1.2% | -0.6% | -1.0% | -3.5% | -4.2% |
|  | [19.9% vs. 22.8%] | [22.1% vs. 24.3%] | [23.7% vs. 25.4%] | [25.1% vs. 26.3%] | [26.6% vs. 27.1%] | [26.6% vs. 27.6%] | [24.4% vs. 27.9%] | [23.8% vs. 28.0%] |
| 60-69 years | 1.4% | 0.4% | 0.6% | -0.2% | -0.8% | -2.1% | -1.9% | -5.8% |
|  | [7.5% vs. 6.1%] | [7.7% vs. 7.2%] | [9.1% vs. 8.5%] | [9.6% vs. 9.7%] | [10.2% vs. 11.0%] | [10.2% vs. 12.4%] | [11.7% vs. 13.7%] | [9.3% vs. 15.0%] |
| ≥70 years | 0.3% | 0.8% | 0.6% | 0.4% | 0.4% | 0.1% | 0.2% | 0.0% |
|  | [0.9% vs. 0.6%] | [1.6% vs. 0.8%] | [1.7% vs. 1.1%] | [1.8% vs. 1.3%] | [2.1% vs. 1.7%] | [2.3% vs. 2.2%] | [2.9% vs. 2.7%] | [3.2% vs. 3.3%] |

The discrepancies were <10% difference in all subgroups and years (15 subgroups * 8 calendar years = 120 subgroups and years) with the exception of 12 (10%) subgroup and year cells. The biggest differences were in Hispanic males and females who injected drugs, the subgroups with smallest observed sample size (and therefore largest estimate variances) in the NA-ACCORD.

As a benchmark, could the authors show what co-morbidities the model would predict in HIV-negative people, and whether the model does a reasonable job of those predictions? This could help validate the comorbidity predictions in a broader population and troubleshoot issues like linear relationships with age.

This agent-based simulation model is composed of mathematical functions from observed data of people with HIV. To forecast comorbidity in people without HIV would require building a separate model with mathematical functions from observed data of people without HIV; this is outside the scope of the PEARL model. We note the scope of the model in the Discussion:

*“We did not compare the comorbidities and multimorbidity forecasts to similar people without HIV. Although this comparison would be useful, our goal was to provide future morbidity predictions to inform HIV clinical directors’ planning and direct HIV policy decision-making.”*

Overall, a very interesting study, but the model may require further debugging and validation to ensure the reported predictions are accurate.

PEARL is a dynamic model. We are always improving the model to ensure the best calibration possible and have re-run all estimates for this re-submission to incorporate improvements made to PEARL in the last 6 months (since our Jan 5, 2023 submission).

To document improvements to the PEARL model, we are diligent about updating the methods section of our website (<https://pearlhivmodel.org/method_details.html>). Additionally, we have posted the code for the PEARL model that produced these results (<https://github.com/PearlHivModelingTeam/comorbidityPaper>) and added this link to the second paragraph of the Methods section.

**Statistical Reviewer**

This paper describes a series of projections of comorbidity in people living with HIV in the USA. I was asked for a statistical report and I interpret that to include all aspects of the design and conduct of the study. The version I received did not have page numbers. My numbering has the abstract starting on page 6.

The .pdf file of the submitted manuscript includes the abstract on page 17.

*Points of detail*

Page 6 The results here are presented as facts in the past (`increased', `de-creased') rather than projections (`would increase') which seems potentially misleading in the abstract. In the main text it is fine.

We begin the reporting of results by stating “PEARL forecasts showed” to clarify that these are future forecasts produced from a model.

Page 10 I am afraid I did not understand the paragraph on validation and Figure S2 did not enlighten me. I suspect a few more words would clarify it.

Although there is ambiguity about which concepts the reviewer comments are unclear in the “*Validation*” sub-section of the Methods section, we have attempted to re-write the paragraph for clarity:

*“We compared the estimated annual incidence of each comorbidity from the NA-ACCORD data with the forecasts from PEARL during the calibration period (where both observed NA-ACCORD estimates and PEARL forecasts were available, i.e., 2009-2017). Within each subgroup, we noted the comorbidities with <75% of PEARL forecasts falling within 5% of the NA-ACCORD observed incidence prevalence or within the NA-ACCORD observed 95% confidence (whichever interval was larger,* ***Supplement Figure S1****). We repeated this validation approach for the annual prevalence of each comorbidity (****Supplement Figure S2****).”*

Page 11 The sentence starting `The number of Black/AA MWID and WWID' would make more sense to me if `but' was replaced by `and' as the two clauses do not seem to contradict one another. Have I failed to under-

stand the meaning here? I assume decreased is a typo for decrease.

We have replaced “but” with “and.” “Decreased” is a typo for decrease; this has been corrected.

Page 15 Some of the speculation about limitations seems to me to go beyond what the data shows. It would be equally plausible that the selective mortality from COVID would have removed the people with most morbidities resulting in fewer surviving to 2030.

We have added this to the limitations paragraph in the Discussion:

*“The PEARL model assumes that the trends observed during the calibration period will continue and does not account for influences occurring outside the calibration period (e.g., the impact of emerging care technologies like long-acting ART or the impact of COVID-19 on mortality); such factors influencing comorbidities incidence can be incorporated into PEARL by extending the calibration period when there are more current data available within the 15 subgroups.”*

Page 15 I am not an HIV researcher but I wonder whether the widespread adoption of pre-exposure prophylaxis should be mentioned too. Obviously it cannot yet be in the model since its long-term effects are as yet unknown.”

We appreciate this insight. We added the following statement to the last paragraph of the Discussion: *“The PEARL model does not include HIV transmission. The forecasted annual number of new ART initiators relies on CDC's reported HIV diagnoses and linkage to care during the calibration period and does not consider changes in HIV transmission dynamics after the calibration period, including changes due to HIV prevention efforts (e.g., pre-exposure prophylaxis or PrEP) or during the COVID-19 pandemic (e.g., 2020-2021).“*

Table 1 What are the numbers in parentheses or brackets?

The brackets are the interquartile range (IQR) of the median age for each of the subgroups noted. This is conveyed in the row header “*Subgroups, n % (median age) [IQR*].”

Figure 3 The advertised shading did not appear in the version I received although I suspect it would make the plots unreadable if it did. The different comorbidities are quite hard to distinguish here. Different line type might help to clear up which is which. I had to blow up the PDF to 200% on my ancient monitor to even see there were different symbols. For some reason Figures S3a-o do have shading and more distinguishable lines.

Shaded uncertainty ranges are present around line in *Figure 3*, but the uncertainty range is so narrow due to the large sample size of all agents. You may notice the shading around the estimates close to the year 2030 in *Figure 3. Figures S3a-*o have more visible shading because the sample size of agents is small in each subgroup, so the shaded uncertainty ranges are more visible.

There are different line types for each comorbidity, visible on more recent computer monitors. Additionally, green is used to predict the mental comorbidities with higher prevalence at younger ages, yellow represents the comorbidities more prevalent in middle age, and red comorbidities represent late-stage comorbidities.

Figure 5 It looks Cam did sort the plot but then forgot to delete the comment.

The comment has been omitted.

Mostly for clarification. I will leave it to the clinical reviewers to comment on the plausibility of the projections.

Michael Dewey

**COMMENTS FROM THE ACADEMIC EDITOR**

I agree that this is potentially an interesting paper, for the US readers and policy-makers. It projects the potential burden of multimorbidity in people on ART by ethnic, sex and risk group, in the USA.

However, what I think is missing is the information that a clinician would like to have (rather than a policy/programme person) in understanding whether there is a greater or lesser likelihood that the person with HIV on ART under his/her care will develop multimorbidities, and which ones. With that knowledge some preventive action may be possible. Would it be possible to use the output from the model to give an example of what change there would be from 2020 to 2030 for such a clinician? The projected prevalences of multimorbidities are not that high, and although this is important to know about a population-level, it is not entirely clear for the clinician.

The PEARL model is a population-based simulation of people living with HIV, with the capacity to forecast trends in aging with HIV and burden of comorbidities through the year 2030 among 15 key subgroups in the US. As the academic editor correctly alludes to, the PEARL model can act as **a decision support tool** for HIV policymakers to inform future HIV care policies in the US: for example, the PEARL projections suggest an alarming increase in burden of CKD among PWH over the next decade which will assert high financial burdens, particularly if renal replacement therapy is needed. Black women with injection drug use as their HIV acquisition risk group have a high burden of CKD, and this group may experience additional barriers to accessing care. Such forecasts can provide information to plan for CKD healthcare costs and lower barriers to nephrology care in anticipation of this growing healthcare need.

Following this, the take-home message for clinicians is that comorbidity care needs of their patient populations (as a mix of the 15 subgroups) is likely to change from 2020-2030, potentially forcing the need for clinicians to stay up-to-date on care guidelines for the most burdensome comorbidities, or build/strengthen connections with subspecialty clinicians who care for the most burdensome comorbidities.

The PEARL model can also act as an experimental tool to forecast the impact of preventive interventions for different comorbidities. We are currently working on a new study focusing on impact of a weight management program in the first 24 months after ART initiation on future reductions in incident diabetes diagnosis and other comorbidities among persons aging with HIV. Such results can inform the design of new clinical trials to assess the impact of such programs and to inform clinicians about the potential effectiveness of such interventions at the patient- and population-level.

Finally, it is important to keep in mind that the PEARL model is programmed as a series of mathematical functions that assign incident comorbidities to the agents as they age with HIV, ultimately producing a **population-level perspective** on the changing burdens of comorbidities and multimorbidity through 2030. This is a different methodologic and conceptual approach than developing prediction models for risk of disease among patients, as has been done via other models such as the *Framingham Risk Score for Hard Coronary Heart Disease* (<https://www.framinghamheartstudy.org/fhs-risk-functions/cardiovascular-disease-10-year-risk/>). While such models can predict an individual’s risk for certain comorbidities, they cannot forecast the population-level burden of these comorbidities in a defined population; this is the main aim of the PEARL model.

Also, the age distribution in PEARL 2020 and projected PEARL 2030 is somewhat different. How much of the increase is associated with changes in the projected age distribution of people on ART?

The age distributions within the 15 subgroups and overall change from 2020 to 2030, and are fully described in Althoff KN et al., AIDS, 2022 (doi: 10.1097/QAD.00000000000031280). It is unclear which outcome the editors are referring to when they inquire “How much of the increase,” but age is playing a role in the mathematical functions estimating the probability of each comorbidity (see the coefficients for age [βage] in *Supplemental Table S2*).

The model allows for age, but is it possible to clarify whether some (or all) of the projected multimorbidity increase is related to increases in particular age groups (as opposed to a general increase overall)? Would there be changes in the distribution of multi-morbidity by age in 2030, compared to 2020? What drives this increase?

The following Figure shows the forecasted burden of multimorbidity among people with HIV in the US by age group from 2010 to 2030. Along with the increase in population size of people with HIV and the aging phenomena, the older age groups show an increasing number of people with HIV in 2030 compared to 2010. Moreover, among those surviving to older ages, the proportion of persons living with 2+ physical comorbidities is also increasing (69% of persons age 70+ living with 2+ comorbidities in 2020 [n= 28,568 persons], and 80% of persons age 70+ living with 2+ comorbidities in 2030 [n=113,291 persons]).

This forecasted trend in aging and the burden of multimorbidity is heterogeneous among the 15 subgroups. In response to this comment, we have now included the set of plots showing the trend in multimorbidity burden by age groups among all people with HIV and 15-subgroups in the Supplement (**Figure S3a-p**) and noted it in the Results:

*“Multimorbidity burden by age group overall and within each of the 15 subgroups is available in* ***Supplement Figure S3a-p****.”*

Further, why is there no allowance for ART regimen? And ART history? Both regimen and previous history could bear on prevalence/incidence of morbidity. This is something that should at least be discussed, and if the data are not available then it should be noted under limitations.

Please see our more elaborate response to this question posed by Reviewer #1 (above).

Briefly, the mathematical functions predicting comorbidity incidence do not explicitly include ART regimens. Because the functions were estimated among people who initiated ART observed in the NA-ACCORD, these associations are implicitly impacting the frequency of outcomes. We have added the following limitation to the Discussion:

*“ART regimen type is not explicitly contained in the PEARL model, but the impact of various ART regimens on comorbidity incidence is implicitly contained in the mathematical functions from observed NA-ACCORD data.”*

It is not clear to me how specific to the PEARL model and available data this model is - can it be generalized across the USA (assuming not all HIV infected people start ART and likelihood of starting may change over time). This discussion could be informed by providing some understanding of what drives the increases in multimorbidity.

We have added the following sentence to the second paragraph of the Methods section:

*“Due to the structure of PEARL and the data sources, findings are generalizable to PWH using ART in the US who identify with the 15 subgroups included in PEARL.”*

The Discussion includes the following statements of what is driving the increases in multimorbidity:

*“**Among the comorbidities included in the PEARL model, the forecasts suggest that two of the greatest contributors to multimorbidity are two common mental health diagnoses that occur throughout the lifespan: depression and anxiety. The prevalence of anxiety was forecasted to increase in all the subgroups by the year 2030 and by 10 ppc overall. In 2030, the prevalence of mental or physical multimorbidity is forecasted to be 70% and nearly 1 in 3 (31%) PWH using ART will have mental comorbidity and physical multimorbidity by 2030; these estimates are a lower limit due to the inclusion of only 2 mental and 7 physical comorbidities when forecasting multimorbidity.”*

*“Over the next decade, these metabolic and vascular diseases may drive the PEARL-forecasted increases in dyslipidemia, CKD, diabetes, and MIs. Importantly, the prevalence of hypertension is forecasted to decline slightly by 4 ppc to 33% in 2030, perhaps due to increases in younger, healthier adults entering HIV care through successful test-and-treat (without delay) initiatives and the “Treat All” clinical guidelines, the reduced use of protease inhibitors (which have been linked to metabolic disorders), or the greater likelihood of a plateau or decrease when prevalence is high.^18,45^ Although hypertension prevalence may decline slightly, the MI burden is forecasted to increase, which is influenced by the increasing prevalence of other comorbidities and the increasing number of PWH at older ages by 2030.^14,46^”*

*“Implementing clinical program interventions that are tailored to Black/AA women and focused on prevention and management of diabetes, hypertension, and anxiety may prove beneficial in slowing future multimorbidity growth among Black/AA WWID. The PEARL modeling team is pursuing estimates of forecasted comorbidities in scenarios where a prominent risk factor is reduced by a specified amount.”*

1. Panel on Antiretroviral Guidelines for Adults and Adolescents. Guidelines for the Use of Antiretroviral Agents in Adults and Adolescents with HIV. Available at <https://clinicalinfo.hiv.gov/en/guidelines/adult-and-adoescent-arv>. Accessed October 3, 2023. Page J-42.
2. US Preventive Services Tas Force. Screening for Anxiety Disorders in Adults: US Preventive Services Task Force Recommendation Statement. JAMA 2023;329(24):2163-2170.
3. Grinspoon SK, et al. Pitavastatin to prevent cardiovascular disease in HIV infection. NEJM 2023;389(8):687-699.
4. US Centers for Disease Control and Prevention AtlasPlus. Available at <https://www.cdc.gov/nchhstp/atlas/index.htm>. Accessed October 11, 2023.
5. US Centers for Disease Control and Prevention AtlasPlus. Available at <https://www.cdc.gov/nchhstp/atlas/index.htm>. Accessed October 11, 2023.
6. US Centers for Disease Control and Prevention AtlasPlus. Available at <https://www.cdc.gov/nchhstp/atlas/index.htm>. Accessed October 11, 2023.
7. US Centers for Disease Control and Prevention AtlasPlus. Available at <https://www.cdc.gov/nchhstp/atlas/index.htm>. Accessed October 11, 2023.
